# Supplementary material for: Discovery and application of insertion-deletion (INDEL) polymorphisms for QTL mapping of early life-history traits in Atlantic salmon
Source: BMC Genomics. 2010 Mar 8;11:156. doi: 10.1186/1471-2164-11-156 (PMC2838853; doi:10.1186/1471-2164-11-156)
Supplement: Additional file 2 — Information on developed 76 locus single-run INDEL panel in Atlantic salmon. Information on fluorescence labeling, primer concentrations, PCR pooling and links to alignments, INDEL motifs and GENESCAN (Burge and Karlin 1997) predictions of genes/exons are available in html format. [file 1471-2164-11-156-S2.ZIP › Additionalfile2/table228.html]

Contig Alignment

```
Base	A B C D E F G H I J K L M N O P Q R S T U V W X Y Z a b c d e f g h i j k l m n o p q r s t u v w x y z A B C D E F G H I J K L M N O P Q R S T U V W X Y Z a b c d e f g h i j k l m n o p q r s t u v w x y z A B C D E F G H I J K L M N O P Q R S T U V W X Y Z a b c d e f g h i   
1	. . . . . . . . . . . . . . . . . . . . . G . . . . . . . G . . . . . . . . . . . . . . . . . . . . . . . . . . . . . . . . . . . . . . . . . . . . . . . . . . . . . . . . . . . . . . . . . . . . . . . . . . . . . . . . . . . . . . . . . . . . . . . . . . . . . . . . . . . . .    
2	. . . . . . . . . . . . . . . . . G G G G G . G . G . . . G . . . . . . . . . . . . . . . . . . . . . . . . . . . . . . . . . . . . . . . . . . . . . . . . . . . . . . . . . . . . . . . . . . . . . . . . . . . . . . . . . . . . . . . . . . . . . . . . . . . . . . . . . . . . .    
3	. . . . . . . . . . . . . . . . . G G G G G . G . G . . . G . . . . . . . . . . . . . . . . . . . . . . . . . . . . . . . . . . . . . . . . . . . . . . . . . . . . . . . . . . . . . . . . . . . . . . . . . . . . . . . . . . . . . . . . . . . . . . . . . . . . . . . . . . . . .    
4	. . . . . . . . . . . . . . . . . G G G G G . G . G . . . G . . . . . . . . . . . . . . . . . . . . . . . . . . . . . . . . . . . . . . . . . . . . . . . . . . . . . . . . . . . . . . . . . . . . . . . . . . . . . . . . . . . . . . . . . . . . . . . . . . . . . . . . . . . . .    
5	. . . . . . . . . . . . . . . . . G G G G G . G . G . . . G . . . . . . . . . . . . . . . . . . . . . . . . . . . . . . . . . . . . . . . . . . . . . . . . . . . . . . . . . . . . . . . . . . . . . . . . . . . . . . . . . . . . . . . . . . . . . . . . . . . . . . . . . . . . .    
6	. . . . . . . . . . . . . . . . . G G G G G . G . G . . . G . . . . . . . . . . . . . . . . . . . . . . . . . . . . . . . . . . . . . . . . . . . . . . . . . . . . . . . . . . . . . . . . . . . . . . . . . . . . . . . . . . . . . . . . . . . . . . . . . . . . . . . . . . . . .    
7	. . . . . . . . . . . . . . . . . A A A A A . A . A . . . A . . . . . . . . . . . . . . . . . . . . . . . . . . . . . . . . . . . . . . . . . . . . . . . . . . . . . . . . . . . . . . . . . . . . . . . . . . . . . . . . . . . . . . . . . . . . . . . . . . . . . . . . . . . . .    
8	. . . . . . . . . . . . . . . . . G G G G G . G . G . . . G . . . . . . . . . . . . . . . . . . . . . . . . . . . . . . . . . . . . . . . . . . . . . . . . . . . . . . . . . . . . . . . . . . . . . . . . . . . . . . . . . . . . . . . . . . . . . . . . . . . . . . . . . . . . .    
9	. . . . . . . . . . . . . . . . . T T T T T . T . T . . . T . . . . . . . . . . . . . . . . . . . . . . . . . . . . . . . . . . . . . . . . . . . . . . . . . . . . . . . . . . . . . . . . . . . . . . . . . . . . . . . . . . . . . . . . . . . . . . . . . . . . . . . . . . . . .    
10	. . . . . T . . . . . . . . . . . T T T T T . T . T . . . T . . . . . . . . . . . . . . . . . . . . . . . . . . . . . . . . . . . . . . . . . . . . . . . . . . . . . . . . . . . . . . . . . . . . . . . . . . . . . . . . . . . . . . . . . . . . . . . . . . . . . . . . . . . . .    
11	. . . . . C . . . . . . . . . . . C C C C C . C . C . . . C . . . . . . . . . . . . . . . . . . . . . . . . . . . . . . . . . . . . . . . . . . . . . . . . . . . . . . . . . . . . . . . . . . . . . . . . . . . . . . . . . . . . . . . . . . . . . . . . . . . . . . . . . . . . .    
12	. . . . . A . . . . . . . . . . . A A A A A . A . A . A . A . . . . . . . . . . . . . . . . . . . . . . . . . . . . . . . . . . . . . . . . . . . . . . . . . . . . . . . . . . . . . . . . . . . . . . . . . . . . . . . . . . . . . . . . . . . . . . . . . . . . . . . . . . . . .    
13	. . . . . G . . . . . . . . . . . G G G G G . G . G . G . G . . . . . . . . . . . . . . . . . . . . . . . . . . . . . . . . . . . . . . . . . . . . . . . . . . . . . . . . . . . . . . . . . . . . . . . . . . . . . . . . . . . . . . . . . . . . . . . . . . . . . . . . . . . . .    
14	. . . . T T . . . . . . . . . . . T T T T T . T . T . T . T . . . . . . . . . . . . . . . . . . . . . . . . . . . . . . . . . . . . . . . . . . . . . . . . . . . . . . . . . . . . . . . . . . . . . . . . . . . . . . . . . . . . . . . . . . . . . . . . . . . . . . . . . . . . .    
15	. . . . C C . . . . . . . . . . . C C C C C . C . C . C . C . . . . . . . . . . . . . . . . . . . . . . . . . . . . . . . . . . . . . . . . . . . . . . . . . . . . . . . . . . . . . . . . . . . . . . . . . . . . . . . . . . . . . . . . . . . . . . . . . . . . . . . . . . . . .    
16	. . . . T T . . . . . . . . . . . T T T T T . T . T . T . T . . . . . . . . . . . . . . . . . . . . . . . . . . . . . . . . . . . . . . . . . . . . . . . . . . . . . . . . . . . . . . . . . . . . . . . . . . . . . . . . . . . . . . . . . . . . . . . . . . . . . . . . . . . . .    
17	. . . . C C . . . . . . . . . . . C C C C C . C . C . C . C . . . . . . . . . . . . . . . . . . . . . . . . . . . . . . . . . . . . . . . . . . . . . . . . . . . . . . . . . . . . . . . . . . . . . . . . . . . . . . . . . . . . . . . . . . . . . . . . . . . . . . . . . . . . .    
18	. . . . A A . . . . . . . . . . . A A A A A . A . A . A . A . . . . . . . . . . . . . . . . . . . . . . . . . . . . . . . . . . . . . . . . . . . . . . . . . . . . . . . . . . . . . . . . . . . . . . . . . . . . . . . . . . . . . . . . . . . . . . . . . . . . . . . . . . . . .    
19	. . . . C C . . . . . . . . . . . C C C C C . C . C . C . C . . . . . . . . . . . . . . . . . . . . . . . . . . . . . . . . . . . . . . . . . . . . . . . . . . . . . . . . . . . . . . . . . . . . . . . . . . . . . . . . . . . . . . . . . . . . . . . . . . . . . . . . . . . . .    
20	. . . . C C . . . . . . . . . . . C C C C C . C . C . C . C . . . . . . . . . . . . . . . . . . . . . . . . . . . . . . . . . . . . . . . . . . . . . . . . . . . . . . . . . . . . . . . . . . . . . . . . . . . . . . . . . . . . . . . . . . . . . . . . . . . . . . . . . . . . .    
21	. . . . C C . . . . . . . . . . C C C C C C . C . C . C . C . . . . . . . . . . . . . . . . . . . . . . . . . . . . . . . . . . . . . . . . . . . . . . . . . . . . . . . . . . . . . . . . . . . . . . . . . . . . . . . . . . . . . . . . . . . . . . . . . . . . . . . . . . . . .    
22	. . . . A A . . . . . . . . . . A A A A A A . A . A . A . A . . . . . . . . . . . . . . . . . . . . . . . . . . . . . . . . . . . . . . . . . . . . . . . . . . . . . . . . . . . . . . . . . . . . . . . . . . . . . . . . . . . . . . . . . . . . . . . . . . . . . . . . . . . . .    
23	. . . . G G . . . . . . . . . . G G G G G G . G . G . G . G . . . . . . . . . . . . . . . . . . . . . . . . . . . . . . . . . . . . . . . . . . . . . . . . . . . . . . . . . . . . . . . . . . . . . . . . . . . . . . . . . . . . . . . . . . . . . . . . . . . . . . . . . . . . .    
24	. . . . C C . . . . . . . . . . C C C C C C . C . C . C . C . . . . . . . . . . . . . . . . . . . . . . . . . . . . . . . . . . . . . . . . . . . . . . . . . . . . . . . . . . . . . . . . . . . . . . . . . . . . . . . . . . . . . . . . . . . . . . . . . . . . . . . . . . . . .    
25	. . . . A A . . . . . . . . . . A A A A A A . A . A . A . A . . . . . . . . . . . . . . . . . . . . . . . . . . . . . . . . . . . . . . . . . . . . . . . . . . . . . . . . . . . . . . . . . . . . . . . . . . . . . . . . . . . . . . . . . . . . . . . . . . . . . . . . . . . . .    
26	. . . G G G G . . . . . . . . . G G G G G G . G . G . G . G . . . . . . . . . . . . . . . . . . . . . . . . . . . . . . . . . . . . . . . . . . . . . . . . . . . . . . . . . . . . . . . . . . . . . . . . . . . . . . . . . . . . . . . . . . . . . . . . . . . . . . . . . . . . .    
27	. . . A A A A . . . . . . . . . A A A A A A . A . A . A . A . . . . . . . . . . . . . . . . . . . . . . . . . . . . . . . . . . . . . . . . . . . . . . . . . . . . . . . . . . . . . . . . . . . . . . . . . . . . . . . . . . . . . . . . . . . . . . . . . . . . . . . . . . . . .    
28	G . . G G G G G G G G . . G . . G G G G G G . G . G . G . G . . . . . . . . . . . . . . . . . . . . . . . . . . . . . . . . . . . . . . . . . . . . . . . . . . . . . . . . . . . . . . . . . . . . . . . . . . . . . . . . . . . . . . . . . . . . . . . . . . . . . . . . . . . . .    
29	G . . G G G G G G G G . . G . . G G G G G G . G . G . G . G . . . . . . . . . . . . . . . . . . . . . . . . . . . . . . . . . . . . . . . . . . . . . . . . . . . . . . . . . . . . . . . . . . . . . . . . . . . . . . . . . . . . . . . . . . . . . . . . . . . . . . . . . . . . .    
30	A . . A A A A A A A A . . A . . A A A A A A . A . A . A . A . . . . . . . . . . . . . . . . . . . . . . . . . . . . . . . . . . . . . . . . . . . . . . . . . . . . . . . . . . . . . . . . . . . . . . . . . . . . . . . . . . . . . . . . . . . . . . . . . . . . . . . . . . . . .    
31	A . . A A A A A A A A . . A . . A A A A A A . A . A . A . A . . . . . . . . . . . . . . . . . . . . . . . . . . . . . . . . . . . . . . . . . . . . . . . . . . . . . . . . . . . . . . . . . . . . . . . . . . . . . . . . . . . . . . . . . . . . . . . . . . . . . . . . . . . . .    
32	C . . C C C C C C C C . . C . . C C C C C C . C . C . C . C . . . . . . . . . . . . . . . . . . . . . . . . . . . . . . . . . . . . . . . . . . . . . . . . . . . . . . . . . . . . . . . . . . . . . . . . . . . . . . . . . . . . . . . . . . . . . . . . . . . . . . . . . . . . .    
33	A . . A A A A A A A A . . A . . A A A A A A . A . A . A . A . . . . . . . . . . . . . . . . . . . . . . . . . . . . . . . . . . . . . . . . . . . . . . . . . . . . . . . . . . . . . . . . . . . . . . . . . . . . . . . . . . . . . . . . . . . . . . . . . . . . . . . . . . . . .    
34	T . . T T T T T T T T . . T . . T T T T T T . T . T . T . T . . . . . . . . . . . . . . . . . . . . . . . . . . . . . . . . . . . . . . . . . . . . . . . . . . . . . . . . . . . . . . . . . . . . . . . . . . . . . . . . . . . . . . . . . . . . . . . . . . . . . . . . . . . . .    
35	G . . G G G G G G G G . . G . . G G G G G G . G . G . G . G . . . . . . . . . . . . . . . . . . . . . . . . . . . . . . . . . . . . . . . . . . . . . . . . . . . . . . . . . . . . . . . . . . . . . . . . . . . . . . . . . . . . . . . . . . . . . . . . . . . . . . . . . . . . .    
36	T . . T T T T T T T T . . T . . T T T T T T . T . T . T . T . . . . . . . . . . . . . . . . . . . . . . . . . . . . . . . . . . . . . . . . . . . . . . . . . . . . . . . . . . . . . . . . . . . . . . . . . . . . . . . . . . . . . . . . . . . . . . . . . . . . . . . . . . . . .    
37	C . . C C C C C C C C . . C . . C C C C C C . C . C . C . C . . . . . . . . . . . . . . . . . . . . . . . . . . . . . . . . . . . . . . . . . . . . . . . . . . . . . . . . . . . . . . . . . . . . . . . . . . . . . . . . . . . . . . . . . . . . . . . . . . . . . . . . . . . . .    
38	G . . G G G G G G G G . . G . . G G G G G G . G . G . G . G . . . . . . . . . . . . . . . . . . . . . . . . . . . . . . . . . . . . . . . . . . . . . . . . . . . . . . . . . . . . . . . . . . . . . . . . . . . . . . . . . . . . . . . . . . . . . . . . . . . . . . . . . . . . .    
39	A . . A A A A A A A A . . A A . A A A A A A . A . A . A . A . . . . . . . . . . . . . . . . . . . . . . . . . . . . . . . . . . . . . . . . . . . . . . . . . . . . . . . . . . . . . . . . . . . . . . . . . . . . . . . . . . . . . . . . . . . . . . . . . . . . . . . . . . . . .    
40	T . . T T T T T T T T . . T T . T T T T T T . T . T . T . T . . . . . . . . . . . . . . . . . . . . . . . . . . . . . . . . . . . . . . . . . . . . . . . . . . . . . . . . . . . . . . . . . . . . . . . . . . . . . . . . . . . . . . . . . . . . . . . . . . . . . . . . . . . . .    
41	G . . G G G G G G G G . . G G . G G G G G G . G . G . G . G . . . . . . . . . . . . . . . . . . . . . . . . . . . . . . . . . . . . . . . . . . . . . . . . . . . . . . . . . . . . . . . . . . . . . . . . . . . . . . . . . . . . . . . . . . . . . . . . . . . . . . . . . . . . .    
42	T . . T T T T T T T T . . T T . T T T T T T . T . T . T . T . . . . . . . . . . . . . . . . . . . . . . . . . . . . . . . . . . . . . . . . . . . . . . . . . . . . . . . . . . . . . . . . . . . . . . . . . . . . . . . . . . . . . . . . . . . . . . . . . . . . . . . . . . . . .    
43	C . . C C C C C C C C . . C C C C C C C C C . C . C . C . C . . . . . . . . . . . . . . . . . . . . . . . . . . . . . . . . . . . . . . . . . . . . . . . . . . . . . . . . . . . . . . . . . . . . . . . . . . . . . . . . . . . . . . . . . . . . . . . . . . . . . . . . . . . . .    
44	T . . T T T T T T T T . . T T T T T T T T T . T . T . T . T . . . . . . . . . . . . . . . . . . . . . . . . . . . . . . . . . . . . . . . . . . . . . . . . . . . . . . . . . . . . . . . . . . . . . . . . . . . . . . . . . . . . . . . . . . . . . . . . . . . . . . . . . . . . .    
45	A . . A A A A A A A A . . A A A A A A A A A . A . A . A . A . . . . . . . . . . . . . . . . . . . . . . . . . . . . . . . . . . . . . . . . . . . . . . . . . . . . . . . . . . . . . . . . . . . . . . . . . . . . . . . . . . . . . . . . . . . . . . . . . . . . . . . . . . . . .    
46	T . . T T T T T T T T . . T T T T T T T T T . T . T . T . T . . . . . . . . . . . . . . . . . . . . . . . . . . . . . . . . . . . . . . . . . . . . . . . . . . . . . . . . . . . . . . . . . . . . . . . . . . . . . . . . . . . . . . . . . . . . . . . . . . . . . . . . . . . . .    
47	C . . C C C C C C C C . . C C C C C C C C C . C C C . C . C . . . . . . . . . . . . . . . . . . . . . . . . . . . . . . . . . . . . . . . . . . . . . . . . . . . . . . . . . . . . . . . . . . . . . . . . . . . . . . . . . . . . . . . . . . . . . . . . . . . . . . . . . . . . .    
48	T . . T T T T T T T T . . T T T T T T T T T . T T T . T . T . . . . . . . . . . . . . . . . . . . . . . . . . . . . . . . . . . . . . . . . . . . . . . . . . . . . . . . . . . . . . . . . . . . . . . . . . . . . . . . . . . . . . . . . . . . . . . . . . . . . . . . . . . . . .    
49	T . . T T T T T T T T . . T T T T T T T T T . T T T . T . T . . . . . . . . . . . . . . . . . . . . . . . . . . . . . . . . . . . . . . . . . . . . . . . . . . . . . . . . . . . . . . . . . . . . . . . . . . . . . . . . . . . . . . . . . . . . . . . . . . . . . . . . . . . . .    
50	C . . C C C C C C C C . . C C C C C C C C C . C C C . C . C . . . . . . . . . . . . . . . . . . . . . . . . . . . . . . . . . . . . . . . . . . . . . . . . . . . . . . . . . . . . . . . . . . . . . . . . . . . . . . . . . . . . . . . . . . . . . . . . . . . . . . . . . . . . .    
51	T . . T T T T T T T T . . T T T T T T T T T . T T T . T . T . . . . . . . . . . . . . . . . . . . . . . . . . . . . . . . . . . . . . . . . . . . . . . . . . . . . . . . . . . . . . . . . . . . . . . . . . . . . . . . . . . . . . . . . . . . . . . . . . . . . . . . . . . . . .    
52	G . . G G G G G G G G . . G G G G G G G G G . G G G . G . G . . . . . . . . . . . . . . . . . . . . . . . . . . . . . . . . . . . . . . . . . . . . . . . . . . . . . . . . . . . . . . . . . . . . . . . . . . . . . . . . . . . . . . . . . . . . . . . . . . . . . . . . . . . . .    
53	C . . C C C C C C C C . . C C C C C C C C C . C C C . C . C . . . . . . . . . . . . . . . . . . . . . . . . . . . . . . . . . . . . . . . . . . . . . . . . . . . . . . . . . . . . . . . . . . . . . . . . . . . . . . . . . . . . . . . . . . . . . . . . . . . . . . . . . . . . .    
54	G . . G G G G G G G G . . G G G G G G G G G . G G G . G . G . . . . . . . . . . . . . . . . . . . . . . . . . . . . . . . . . . . . . . . . . . . . . . . . . . . . . . . . . . . . . . . . . . . . . . . . . . . . . . . . . . . . . . . . . . . . . . . . . . . . . . . . . . . . .    
55	T . . T T T T T T T T . . T T T T T T T T T . T T T . T . T . . . . . . . . . . . . . . . . . . . . . . . . . . . . . . . . . . . . . . . . . . . . . . . . . . . . . . . . . . . . . . . . . . . . . . . . . . . . . . . . . . . . . . . . . . . . . . . . . . . . . . . . . . . . .    
56	T . . T T T T T T T T . . T T T T T T T T T . T T T . T . T . . . . . . . . . . . . . . . . . . . . . . . . . . . . . . . . . . . . . . . . . . . . . . . . . . . . . . . . . . . . . . . . . . . . . . . . . . . . . . . . . . . . . . . . . . . . . . . . . . . . . . . . . . . . .    
57	- . . T T T T T - T T . . - T T T T T T T T . T T T . T . T . . . . . . . . . . . . . . . . . . . . . . . . . . . . . . . . . . . . . . . . . . . . . . . . . . . . . . . . . . . . . . . . . . . . . . . . . . . . . . . . . . . . . . . . . . . . . . . . . . . . . . . . . . . . .    
58	C . . C C C C C C C C . . C C C C C C C C C . C C C . C . C . . . . . . . . . . . . . . . . . . . . . . . . . . . . . . . . . . . . . . . . . . . . . . . . . . . . . . . . . . . . . . . . . . . . . . . . . . . . . . . . . . . . . . . . . . . . . . . . . . . . . . . . . . . . .    
59	C . . C C C C C C C C . . C C C C C C C C C . C C C . C . C . . . . . . . . . . . . . . . . . . . . . . . . . . . . . . . . . . . . . . . . . . . . . . . . . . . . . . . . . . . . . . . . . . . . . . . . . . . . . . . . . . . . . . . . . . . . . . . . . . . . . . . . . . . . .    
60	C . . C C C C C C C C . . C C C C C C C C C . C C C . C . C . . . . . . . . . . . . . . . . . . . . . . . . . . . . . . . . . . . . . . . . . . . . . . . . . . . . . . . . . . . . . . . . . . . . . . . . . . . . . . . . . . . . . . . . . . . . . . . . . . . . . . . . . . . . .    
61	T . . T T T T T T T T T . T T T T T T T T T . T T T . T . T . . . . . . . . . . . . . . . . . . . . . . . . . . . . . . . . . . . . . . . . . . . . . . . . . . . . . . . . . . . . . . . . . . . . . . . . . . . . . . . . . . . . . . . . . . . . . . . . . . . . . . . . . . . . .    
62	G . . G G G G G G G G G . G G G G G G G G G . G G G . G . G . . . . . . . . . . . . . . . . . . . . . . . . . . . . . . . . . . . . . . . . . . . . . . . . . . . . . . . . . . . . . . . . . . . . . . . . . . . . . . . . . . . . . . . . . . . . . . . . . . . . . . . . . . . . .    
63	A . . A A A A A A A A A . A A A A A A A A A . A A A . A . A . . . . . . . . . . . . . . . . . . . . . . . . . . . . . . . . . . . . . . . . . . . . . . . . . . . . . . . . . . . . . . . . . . . . . . . . . . . . . . . . . . . . . . . . . . . . . . . . . . . . . . . . . . . . .    
64	C . . C C C C C C C C C . C C C C C C C C C . C C C . C . C . . . . . . . . . . . . . . . . . . . . . . . . . . . . . . . . . . . . . . . . . . . . . . . . . . . . . . . . . . . . . . . . . . . . . . . . . . . . . . . . . . . . . . . . . . . . . . . . . . . . . . . . . . . . .    
65	C . . C C C C C C C C C . C C C C C C C C C . C C C . C . C . . . . . . . . . . . . . . . . . . . . . . . . . . . . . . . . . . . . . . . . . . . . . . . . . . . . . . . . . . . . . . . . . . . . . . . . . . . . . . . . . . . . . . . . . . . . . . . . . . . . . . . . . . . . .    
66	C . . C C C C C C C C C . C C C C C C C C C . C C C . C . C . . . . . . . . . . . . . . . . . . . . . . . . . . . . . . . . . . . . . . . . . . . . . . . . . . . . . . . . . . . . . . . . . . . . . . . . . . . . . . . . . . . . . . . . . . . . . . . . . . . . . . . . . . . . .    
67	T . . T T T T T T T T T . T T T T T T T T T . T T T . T . T . . . . . . . . . . . . . . . . . . . . . . . . . . . . . . . . . . . . . . . . . . . . . . . . . . . . . . . . . . . . . . . . . . . . . . . . . . . . . . . . . . . . . . . . . . . . . . . . . . . . . . . . . . . . .    
68	G . . G G G G G G G G G . G G G G G G G G G . G G G . G . G . . . . . . . . . . . . . . . . . . . . . . . . . . . . . . . . . . . . . . . . . . . . . . . . . . . . . . . . . . . . . . . . . . . . . . . . . . . . . . . . . . . . . . . . . . . . . . . . . . . . . . . . . . . . .    
69	G . . G G G G G G G G G . G G G G G G G G G . G G G . G . G . . . . . . . . . . . . . . . . . . . . . . . . . . . . . . . . . . . . . . . . . . . . . . . . . . . . . . . . . . . . . . . . . . . . . . . . . . . . . . . . . . . . . . . . . . . . . . . . . . . . . . . . . . . . .    
70	T . . T T T T T T T T T . T T T T T T T T T . T T T . T . T . . . . . . . . . . . . . . . . . . . . . . . . . . . . . . . . . . . . . . . . . . . . . . . . . . . . . . . . . . . . . . . . . . . . . . . . . . . . . . . . . . . . . . . . . . . . . . . . . . . . . . . . . . . . .    
71	T . . T T T T T T T T T . T T T T T T T T T . T T T . T . T . . . . . . . . . . . . . . . . . . . . . . . . . . . . . . . . . . . . . . . . . . . . . . . . . . . . . . . . . . . . . . . . . . . . . . . . . . . . . . . . . . . . . . . . . . . . . . . . . . . . . . . . . . . . .    
72	T . . T T T T T T T T T . T T T T T T T T T . T T T . T . T . . . . . . . . . . . . . . . . . . . . . . . . . . . . . . . . . . . . . . . . . . . . . . . . . . . . . . . . . . . . . . . . . . . . . . . . . . . . . . . . . . . . . . . . . . . . . . . . . . . . . . . . . . . . .    
73	T . . T T T T T T T T T . T T T T T T T T T . T T T . T . T . . . . . . . . . . . . . . . . . . . . . . . . . . . . . . . . . . . . . . . . . . . . . . . . . . . . . . . . . . . . . . . . . . . . . . . . . . . . . . . . . . . . . . . . . . . . . . . . . . . . . . . . . . . . .    
74	G . . G G G G G G G G G . G G G G G G G G G . G G G . G . G . . . . . . . . . . . . . . . . . . . . . . . . . . . . . . . . . . . . . . . . . . . . . . . . . . . . . . . . . . . . . . . . . . . . . . . . . . . . . . . . . . . . . . . . . . . . . . . . . . . . . . . . . . . . .    
75	T . . T T T T T T T T T . T T T T T T T T T . T T T . T . T . . . . . . . . . . . . . . . . . . . . . . . . . . . . . . . . . . . . . . . . . . . . . . . . . . . . . . . . . . . . . . . . . . . . . . . . . . . . . . . . . . . . . . . . . . . . . . . . . . . . . . . . . . . . .    
76	C . . C C C C C C C C C . C C C C C C C C C . C C C . C . C . . . . . . . . . . . . . . . . . . . . . . . . . . . . . . . . . . . . . . . . . . . . . . . . . . . . . . . . . . . . . . . . . . . . . . . . . . . . . . . . . . . . . . . . . . . . . . . . . . . . . . . . . . . . .    
77	C C . C C C C C C C C C . C C C C C C C C C . C C C . C . C . . . . . . . . . . . . . . . . . . . . . . . . . . . . . . . . . . . . . . . . . . . . . . . . . . . . . . . . . . . . . . . . . . . . . . . . . . . . . . . . . . . . . . . . . . . . . . . . . . . . . . . . . . . . .    
78	A A A A A A A A A A A A . A A A A A A A A A . A A A . A . A . . . . . . . . . . . . . . . . . . . . . . . . . . . . . . . . . . . . . . . . . . . . . . . . . . . . . . . . . . . . . . . . . . . . . . . . . . . . . . . . . . . . . . . . . . . . . . . . . . . . . . . . . . . . .    
79	T T T T T T T T T T T T . T T T T T T T T T . T T T . T . T . . . . . . . . . . . . . . . . . . . . . . . . . . . . . . . . . . . . . . . . . . . . . . . . . . . . . . . . . . . . . . . . . . . . . . . . . . . . . . . . . . . . . . . . . . . . . . . . . . . . . . . . . . . . .    
80	A A A A A A A A A A A A . A A A A A A A A A . A A A . A . A . . . . . . . . . . . . . . . . . . . . . . . . . . . . . . . . . . . . . . . . . . . . . . . . . . . . . . . . . . . . . . . . . . . . . . . . . . . . . . . . . . . . . . . . . . . . . . . . . . . . . . . . . . . . .    
81	T T T T T T T T T T T T . T T T T T T T T T . T T T . T . T . . . . . . . . . . . . . . . . . . . . . . . . . . . . . . . . . . . . . . . . . . . . . . . . . . . . . . . . . . . . . . . . . . . . . . . . . . . . . . . . . . . . . . . . . . . . . . . . . . . . . . . . . . . . .    
82	T T T T T T T T T T T T . T T T T T T T T T . T T T . T . T . . . . . . . . . . . . . . . . . . . . . . . . . . . . . . . . . . . . . . . . . . . . . . . . . . . . . . . . . . . . . . . . . . . . . . . . . . . . . . . . . . . . . . . . . . . . . . . . . . . . . . . . . . . . .    
83	C C C C C C C C C C C C . C C C C C C C C C . C C C . C . C . . . . . . . . . . . . . . . . . . . . . . . . . . . . . . . . . . . . . . . . . . . . . . . . . . . . . . . . . . . . . . . . . . . . . . . . . . . . . . . . . . . . . . . . . . . . . . . . . . . . . . . . . . . . .    
84	T T T T T T T T T T T T . T T T T T T T T T . T T T . T . T . . . . . . . . . . . . . . . . . . . . . . . . . . . . . . . . . . . . . . . . . . . . . . . . . . . . . . . . . . . . . . . . . . . . . . . . . . . . . . . . . . . . . . . . . . . . . . . . . . . . . . . . . . . . .    
85	C C C C C C C C C C C C . C C C C C C C C C . C C C . C . C . . . . . . . . . . . . . . . . . . . . . . . . . . . . . . . . . . . . . . . . . . . . . . . . . . . . . . . . . . . . . . . . . . . . . . . . . . . . . . . . . . . . . . . . . . . . . . . . . . . . . . . . . . . . .    
86	T T T T T T T T T T T T . T T T T T T T T T . T T T . T . T . . . . . . . . . . . . . . . . . . . . . . . . . . . . . . . . . . . . . . . . . . . . . . . . . . . . . . . . . . . . . . . . . . . . . . . . . . . . . . . . . . . . . . . . . . . . . . . . . . . . . . . . . . . . .    
87	G G G G G G G G G G G G . G G G G G G G G G . G G G . G . G . . . . . . . . . . . . . . . . . . . . . . . . . . . . . . . . . . . . . . . . . . . . . . . . . . . . . . . . . . . . . . . . . . . . . . . . . . . . . . . . . . . . . . . . . . . . . . . . . . . . . . . . . . . . .    
88	G G G G G G G G G G G G . G G G G G G G G G . G G G . G . G . . . . . . . . . . . . . . . . . . . . . . . . . . . . . . . . . . . . . . . . . . . . . . . . . . . . . . . . . . . . . . . . . . . . . . . . . . . . . . . . . . . . . . . . . . . . . . . . . . . . . . . . . . . . .    
89	A A A A A A A A A A A A . A A A A A A A A A . A A A . A . A . . . . . . . . . . . . . . . . . . . . . . . . . . . . . . . . . . . . . . . . . . . . . . . . . . . . . . . . . . . . . . . . . . . . . . . . . . . . . . . . . . . . . . . . . . . . . . . . . . . . . . . . . . . . .    
90	A A A A A A A A A A A A . A A A A A A A A A . A A A . A . A . . . . . . . . . . . . . . . . . . . . . . . . . . . . . . . . . . . . . . . . . . . . . . . . . . . . . . . . . . . . . . . . . . . . . . . . . . . . . . . . . . . . . . . . . . . . . . . . . . . . . . . . . . . . .    
91	C C C C C C C C C C C C . C C C C C C C C C . C C C . C . C . . . . . . . . . . . . . . . . . . . . . . . . . . . . . . . . . . . . . . . . . . . . . . . . . . . . . . . . . . . . . . . . . . . . . . . . . . . . . . . . . . . . . . . . . . . . . . . . . . . . . . . . . . . . .    
92	A A A A A A A A A A A A . A A A A A A A A A . A A A . A . A . . . . . . . . . . . . . . . . . . . . . . . . . . . . . . . . . . . . . . . . . . . . . . . . . . . . . . . . . . . . . . . . . . . . . . . . . . . . . . . . . . . . . . . . . . . . . . . . . . . . . . . . . . . . .    
93	G G G G G G G G G G G G . G G G G G G G G G . G G G . G . G . . . . . . . . . . . . . . . . . . . . . . . . . . . . . . . . . . . . . . . . . . . . . . . . . . . . . . . . . . . . . . . . . . . . . . . . . . . . . . . . . . . . . . . . . . . . . . . . . . . . . . . . . . . . .    
94	A A A A A A A A A A A A . A A A A A A A A A . A A A . A . A . . . . . . . . . . . . . . . . . . . . . . . . . . . . . . . . . . . . . . . . . . . . . . . . . . . . . . . . . . . . . . . . . . . . . . . . . . . . . . . . . . . . . . . . . . . . . . . . . . . . . . . . . . . . .    
95	T T T T T T T T T T T T . T T T T T T T T T . T T T . T . T . . . . . . . . . . . . . . . . . . . . . . . . . . . . . . . . . . . . . . . . . . . . . . . . . . . . . . . . . . . . . . . . . . . . . . . . . . . . . . . . . . . . . . . . . . . . . . . . . . . . . . . . . . . . .    
96	G G G G G G G G G G G G . G G G G G G G G G . G G G . G . G . . . . . . . . . . . . . . . . . . . . . . . . . . . . . . . . . . . . . . . . . . . . . . . . . . . . . . . . . . . . . . . . . . . . . . . . . . . . . . . . . . . . . . . . . . . . . . . . . . . . . . . . . . . . .    
97	G G G G G G G G G G G G . G G G G G G G G G . G G G . G . G . . . . . . . . . . . . . . . . . . . . . . . . . . . . . . . . . . . . . . . . . . . . . . . . . . . . . . . . . . . . . . . . . . . . . . . . . . . . . . . . . . . . . . . . . . . . . . . . . . . . . . . . . . . . .    
98	A A A A A A A A A A A A . A A A A A A A A A . A A A . A . A . . . . . . . . . . . . . . . . . . . . . . . . . . . . . . . . . . . . . . . . . . . . . . . . . . . . . . . . . . . . . . . . . . . . . . . . . . . . . . . . . . . . . . . . . . . . . . . . . . . . . . . . . . . . .    
99	T T T T T T T T T T T T . T T T T T T T T T . T T T . T . T . . . . . . . . . . . . . . . . . . . . . . . . . . . . . . . . . . . . . . . . . . . . . . . . . . . . . . . . . . . . . . . . . . . . . . . . . . . . . . . . . . . . . . . . . . . . . . . . . . . . . . . . . . . . .    
100	A A A A A A A A A A A A . A A A A A A A A A . A A A . A . A . . . . . . . . . . . . . . . . . . . . . . . . . . . . . . . . . . . . . . . . . . . . . . . . . . . . . . . . . . . . . . . . . . . . . . . . . . . . . . . . . . . . . . . . . . . . . . . . . . . . . . . . . . . . .    
101	T T T T T T T T T T T T . T T T T T T T T T . T T T . T . T . . . . . . . . . . . . . . . . . . . . . . . . . . . . . . . . . . . . . . . . . . . . . . . . . . . . . . . . . . . . . . . . . . . . . . . . . . . . . . . . . . . . . . . . . . . . . . . . . . . . . . . . . . . . .    
102	T T T T T T T T T T T T . T T T T T T T T T . T T T . T . T . . . . . . . . . . . . . . . . . . . . . . . . . . . . . . . . . . . . . . . . . . . . . . . . . . . . . . . . . . . . . . . . . . . . . . . . . . . . . . . . . . . . . . . . . . . . . . . . . . . . . . . . . . . . .    
103	T T T T T T T T T T T T . T T T T T T T T T . T T T . T . T . . . . . . . . . . . . . . . . . . . . . . . . . . . . . . . . . . . . . . . . . . . . . . . . . . . . . . . . . . . . . . . . . . . . . . . . . . . . . . . . . . . . . . . . . . . . . . . . . . . . . . . . . . . . .    
104	T T T T T T T T T T T T . T T T T T T T T T . T T T . T . T . . . . . . . . . . . . . . . . . . . . . . . . . . . . . . . . . . . . . . . . . . . . . . . . . . . . . . . . . . . . . . . . . . . . . . . . . . . . . . . . . . . . . . . . . . . . . . . . . . . . . . . . . . . . .    
105	G G G G G G G G G G G G . G G G G G G G G G . G G G . G . G . . . . . . . . . . . . . . . . . . . . . . . . . . . . . . . . . . . . . . . . . . . . . . . . . . . . . . . . . . . . . . . . . . . . . . . . . . . . . . . . . . . . . . . . . . . . . . . . . . . . . . . . . . . . .    
106	A A A A A A A A A A A A . A A A A A A A A A . A A A . A . A . . . . . . . . . . . . . . . . . . . . . . . . . . . . . . . . . . . . . . . . . . . . . . . . . . . . . . . . . . . . . . . . . . . . . . . . . . . . . . . . . . . . . . . . . . . . . . . . . . . . . . . . . . . . .    
107	A A A A A A A A A A A A . A A A A A A A A A . A A A . A . A . . . . . . . . . . . . . . . . . . . . . . . . . . . . . . . . . . . . . . . . . . . . . . . . . . . . . . . . . . . . . . . . . . . . . . . . . . . . . . . . . . . . . . . . . . . . . . . . . . . . . . . . . . . . .    
108	C C C C C C C C C C C C . C C C C C C C C C . C C C . C . C . . . . . . . . . . . . . . . . . . . . . . . . . . . . . . . . . . . . . . . . . . . . . . . . . . . . . . . . . . . . . . . . . . . . . . . . . . . . . . . . . . . . . . . . . . . . . . . . . . . . . . . . . . . . .    
109	A A A A A A A A A A A A . A A A A A A A A A . A A A . A . A . . . . . . . . . . . . . . . . . . . . . . . . . . . . . . . . . . . . . . . . . . . . . . . . . . . . . . . . . . . . . . . . . . . . . . . . . . . . . . . . . . . . . . . . . . . . . . . . . . . . . . . . . . . . .    
110	G G G G G G G G G G G G . G G G G G G G G G . G G G . G . G . . . . . . . . . . . . . . . . . . . . . . . . . . . . . . . . . . . . . . . . . . . . . . . . . . . . . . . . . . . . . . . . . . . . . . . . . . . . . . . . . . . . . . . . . . . . . . . . . . . . . . . . . . . . .    
111	G G G G G G G G G G G G . G G G G G G G G G . G G G . G . G . . . . . . . . . . . . . . . . . . . . . . . . . . . . . . . . . . . . . . . . . . . . . . . . . . . . . . . . . . . . . . . . . . . . . . . . . . . . . . . . . . . . . . . . . . . . . . . . . . . . . . . . . . . . .    
112	T T T T T T T T T T T T . T T T T T T T T T . T T T . T . T . . . . . . . . . . . . . . . . . . . . . . . . . . . . . . . . . . . . . . . . . . . . . . . . . . . . . . . . . . . . . . . . . . . . . . . . . . . . . . . . . . . . . . . . . . . . . . . . . . . . . . . . . . . . .    
113	T T T T T T T T T T T T . T T T T T T T T T . T T T . T . T . . . . . . . . . . . . . . . . . . . . . . . . . . . . . . . . . . . . . . . . . . . . . . . . . . . . . . . . . . . . . . . . . . . . . . . . . . . . . . . . . . . . . . . . . . . . . . . . . . . . . . . . . . . . .    
114	G G G G G G G G G G G G . G G G G G G G G G . G G G . G . G . . . . . . . . . . . . . . . . . . . . . . . . . . . . . . . . . . . . . . . . . . . . . . . . . . . . . . . . . . . . . . . . . . . . . . . . . . . . . . . . . . . . . . . . . . . . . . . . . . . . . . . . . . . . .    
115	T T T T T T T T T T T T . T T T T T T T T T . T T T . T . T . . . . . . . . . . . . . . . . . . . . . . . . . . . . . . . . . . . . . . . . . . . . . . . . . . . . . . . . . . . . . . . . . . . . . . . . . . . . . . . . . . . . . . . . . . . . . . . . . . . . . . . . . . . . .    
116	G G G G G G G G G G G G . G G G G G G G G G . G G G . G . G . . . . . . . . . . . . . . . . . . . . . . . . . . . . . . . . . . . . . . . . . . . . . . . . . . . . . . . . . . . . . . . . . . . . . . . . . . . . . . . . . . . . . . . . . . . . . . . . . . . . . . . . . . . . .    
117	A A A A A A A A A A A A . A A A A A A A A A . A A A . A . A . . . . . . . . . . . . . . . . . . . . . . . . . . . . . . . . . . . . . . . . . . . . . . . . . . . . . . . . . . . . . . . . . . . . . . . . . . . . . . . . . . . . . . . . . . . . . . . . . . . . . . . . . . . . .    
118	G G G G G G G G G G G G . G G G G G G G G G . G G G . G . G . . . . . . . . . . . . . . . . . . . . . . . . . . . . . . . . . . . . . . . . . . . . . . . . . . . . . . . . . . . . . . . . . . . . . . . . . . . . . . . . . . . . . . . . . . . . . . . . . . . . . . . . . . . . .    
119	A A A A A A A A A A A A . A A A A A A A A A . A A A . A . A . . . . . . . . . . . . . . . . . . . . . . . . . . . . . . . . . . . . . . . . . . . . . . . . . . . . . . . . . . . . . . . . . . . . . . . . . . . . . . . . . . . . . . . . . . . . . . . . . . . . . . . . . . . . .    
120	C C C C C C C C C C C C . C C C C C C C C C . C C C . C . C . . . . . . . . . . . . . . . . . . . . . . . . . . . . . . . . . . . . . . . . . . . . . . . . . . . . . . . . . . . . . . . . . . . . . . . . . . . . . . . . . . . . . . . . . . . . . . . . . . . . . . . . . . . . .    
121	A A A A A A A A A A A A . A A A A A A A A A . A A A . A . A . . . . . . . . . . . . . . . . . . . . . . . . . . . . . . . . . . . . . . . . . . . . . . . . . . . . . . . . . . . . . . . . . . . . . . . . . . . . . . . . . . . . . . . . . . . . . . . . . . . . . . . . . . . . .    
122	G G G G G G G G G G G G . G G G G G G G G G . G G G . G . G . . . . . . . . . . . . . . . . . . . . . . . . . . . . . . . . . . . . . . . . . . . . . . . . . . . . . . . . . . . . . . . . . . . . . . . . . . . . . . . . . . . . . . . . . . . . . . . . . . . . . . . . . . . . .    
123	T T T T T T T T T T T T . T T T T T T T T T . T T T . T . T . . . . . . . . . . . . . . . . . . . . . . . . . . . . . . . . . . . . . . . . . . . . . . . . . . . . . . . . . . . . . . . . . . . . . . . . . . . . . . . . . . . . . . . . . . . . . . . . . . . . . . . . . . . . .    
124	G G G G G G G G G G G G . G G G G G G G G G . G G G . G . G . . . . . . . . . . . . . . . . . . . . . . . . . . . . . . . . . . . . . . . . . . . . . . . . . . . . . . . . . . . . . . . . . . . . . . . . . . . . . . . . . . . . . . . . . . . . . . . . . . . . . . . . . . . . .    
125	C C C C C C C C C C C C . C C C C C C C C C . C C C . C . C . . . . . . . . . . . . . . . . . . . . . . . . . . . . . . . . . . . . . . . . . . . . . . . . . . . . . . . . . . . . . . . . . . . . . . . . . . . . . . . . . . . . . . . . . . . . . . . . . . . . . . . . . . . . .    
126	C C C C C C C C C C C C . C C C C C C C C C . C C C . C . C . . . . . . . . . . . . . . . . . . . . . . . . . . . . . . . . . . . . . . . . . . . . . . . . . . . . . . . . . . . . . . . . . . . . . . . . . . . . . . . . . . . . . . . . . . . . . . . . . . . . . . . . . . . . .    
127	G G G G G G G G G G G G . G G G G G G G G G . G G G . G . G . . . . . . . . . . . . . . . . . . . . . . . . . . . . . . . . . . . . . . . . . . . . . . . . . . . . . . . . . . . . . . . . . . . . . . . . . . . . . . . . . . . . . . . . . . . . . . . . . . . . . . . . . . . . .    
128	A A A A A A A A A A A A . A A A A A A A A A . A A A . A . A . . . . . . . . . . . . . . . . . . . . . . . . . . . . . . . . . . . . . . . . . . . . . . . . . . . . . . . . . . . . . . . . . . . . . . . . . . . . . . . . . . . . . . . . . . . . . . . . . . . . . . . . . . . . .    
129	T T T T T T T T T T T T . T T T T T T T T T . T T T . T . T . . . . . . . . . . . . . . . . . . . . . . . . . . . . . . . . . . . . . . . . . . . . . . . . . . . . . . . . . . . . . . . . . . . . . . . . . . . . . . . . . . . . . . . . . . . . . . . . . . . . . . . . . . . . .    
130	A A A A A A A A A A A A . A A A A A A A A A . A A A . A . A . . . . . . . . . . . . . . . . . . . . . . . . . . . . . . . . . . . . . . . . . . . . . . . . . . . . . . . . . . . . . . . . . . . . . . . . . . . . . . . . . . . . . . . . . . . . . . . . . . . . . . . . . . . . .    
131	C C C C C C C C C C C C . C C C C C C C C C . C C C . C . C . . . . . . . . . . . . . . . . . . . . . . . . . . . . . . . . . . . . . . . . . . . . . . . . . . . . . . . . . . . . . . . . . . . . . . . . . . . . . . . . . . . . . . . . . . . . . . . . . . . . . . . . . . . . .    
132	T T T T T T T T T T T T . T T T T T T T T T . T T T . T . T . . . . . . . . . . . . . . . . . . . . . . . . . . . . . . . . . . . . . . . . . . . . . . . . . . . . . . . . . . . . . . . . . . . . . . . . . . . . . . . . . . . . . . . . . . . . . . . . . . . . . . . . . . . . .    
133	C C C C C C C C C C C C . C C C C C C C C C . C C C . C . C . . . . . . . . . . . . . . . . . . . . . . . . . . . . . . . . . . . . . . . . . . . . . . . . . . . . . . . . . . . . . . . . . . . . . . . . . . . . . . . . . . . . . . . . . . . . . . . . . . . . . . . . . . . . .    
134	C C C C C C C C C C C C . C C C C C C C C C . C C C . C . C . . . . . . . . . . . . . . . . . . . . . . . . . . . . . . . . . . . . . . . . . . . . . . . . . . . . . . . . . . . . . . . . . . . . . . . . . . . . . . . . . . . . . . . . . . . . . . . . . . . . . . . . . . . . .    
135	T T T T T T T T T T T T . T T T T T T T T T . T T T . T . T . . . . . . . . . . . . . . . . . . . . . . . . . . . . . . . . . . . . . . . . . . . . . . . . . . . . . . . . . . . . . . . . . . . . . . . . . . . . . . . . . . . . . . . . . . . . . . . . . . . . . . . . . . . . .    
136	C C C C C C C C C C C C . C C C C C C C C C . C C C . C . C . . . . . . . . . . . . . . . . . . . . . . . . . . . . . . . . . . . . . . . . . . . . . . . . . . . . . . . . . . . . . . . . . . . . . . . . . . . . . . . . . . . . . . . . . . . . . . . . . . . . . . . . . . . . .    
137	A A A A A A A A A A A A . A A A A A A A A A . A A A . A . A . . . . . . . . . . . . . . . . . . . . . . . . . . . . . . . . . . . . . . . . . . . . . . . . . . . . . . . . . . . . . . . . . . . . . . . . . . . . . . . . . . . . . . . . . . . . . . . . . . . . . . . . . . . . .    
138	A A A A A A A A A A A A . A A A A A A A A A . A A A . A . A . . . . . . . . . . . . . . . . . . . . . . . . . . . . . . . . . . . . . . . . . . . . . . . . . . . . . . . . . . . . . . . . . . . . . . . . . . . . . . . . . . . . . . . . . . . . . . . . . . . . . . . . . . . . .    
139	A A A A A A A A A A A A . A A A A A A A A A . A A A . A . A . . . . . . . . . . . . . . . . . . . . . . . . . . . . . . . . . . . . . . . . . . . . . . . . . . . . . . . . . . . . . . . . . . . . . . . . . . . . . . . . . . . . . . . . . . . . . . . . . . . . . . . . . . . . .    
140	G G G G G G G G G G G G . G G G G G G G G G . G G G . G . G . . . . . . . . . . . . . . . . . . . . . . . . . . . . . . . . . . . . . . . . . . . . . . . . . . . . . . . . . . . . . . . . . . . . . . . . . . . . . . . . . . . . . . . . . . . . . . . . . . . . . . . . . . . . .    
141	G G G G G G G G G G G G . G G G G G G G G G . G G G . G . G . . . . . . . . . . . . . . . . . . . . . . . . . . . . . . . . . . . . . . . . . . . . . . . . . . . . . . . . . . . . . . . . . . . . . . . . . . . . . . . . . . . . . . . . . . . . . . . . . . . . . . . . . . . . .    
142	A A A A A A A A A A A A . A A A A A A A A A . A A A . A . A . . . . . . . . . . . . . . . . . . . . . . . . . . . . . . . . . . . . . . . . . . . . . . . . . . . . . . . . . . . . . . . . . . . . . . . . . . . . . . . . . . . . . . . . . . . . . . . . . . . . . . . . . . . . .    
143	C C C C C C C C C C C C . C C C C C C C C C . C C C . C . C . . . . . . . . . . . . . . . . . . . . . . . . . . . . . . . . . . . . . . . . . . . . . . . . . . . . . . . . . . . . . . . . . . . . . . . . . . . . . . . . . . . . . . . . . . . . . . . . . . . . . . . . . . . . .    
144	C C C C C C C C C C C C . C C C C C C C C C . C C C . C . C . . . . . . . . . . . . . . . . . . . . . . . . . . . . . . . . . . . . . . . . . . . . . . . . . . . . . . . . . . . . . . . . . . . . . . . . . . . . . . . . . . . . . . . . . . . . . . . . . . . . . . . . . . . . .    
145	T T T T T T T T T T T T . T T T T T T T T T . T T T . T . T . . . . . . . . . . . . . . . . . . . . . . . . . . . . . . . . . . . . . . . . . . . . . . . . . . . . . . . . . . . . . . . . . . . . . . . . . . . . . . . . . . . . . . . . . . . . . . . . . . . . . . . . . . . . .    
146	G G G G G G G G G G G G . G G G G G G G G G . G G G . G . G . . . . . . . . . . . . . . . . . . . . . . . . . . . . . . . . . . . . . . . . . . . . . . . . . . . . . . . . . . . . . . . . . . . . . . . . . . . . . . . . . . . . . . . . . . . . . . . . . . . . . . . . . . . . .    
147	C C C C C C C C C C C C . C C C C C C C C C . C C C . C . C . . . . . . . . . . . . . . . . . . . . . . . . . . . . . . . . . . . . . . . . . . . . . . . . . . . . . . . . . . . . . . . . . . . . . . . . . . . . . . . . . . . . . . . . . . . . . . . . . . . . . . . . . . . . .    
148	A C A A A A A A A A A A . A A A A A A A A A . A A A . A . A . . . . . . . . . . . . . . . . . . . . . . . . . . . . . . . . . . . . . . . . . . . . . . . . . . . . . . . . . . . . . . . . . . . . . . . . . . . . . . . . . . . . . . . . . . . . . . . . . . . . . . . . . . . . .    
149	G A G G G G G G G G G G . G G G G G G G G G . G G G . G . G . . . . . . . . . . . . . . . . . . . . . . . . . . . . . . . . . . . . . . . . . . . . . . . . . . . . . . . . . . . . . . . . . . . . . . . . . . . . . . . . . . . . . . . . . . . . . . . . . . . . . . . . . . . . .    
150	G G G G G G G G G G G G . G G G G G G G G G . G G G . G . G . . . . . . . . . . . . . . . . . . . . . . . . . . . . . . . . . . . . . . . . . . . . . . . . . . . . . . . . . . . . . . . . . . . . . . . . . . . . . . . . . . . . . . . . . . . . . . . . . . . . . . . . . . . . .    
151	G G G G G G G G G G G G . G G G G G G G G G . G G G . G . G . . . . . . . . . . . . . . . . . . . . . . . . . . . . . . . . . . . . . . . . . . . . . . . . . . . . . . . . . . . . . . . . . . . . . . . . . . . . . . . . . . . . . . . . . . . . . . . . . . . . . . . . . . . . .    
152	T T T T T T T T T T T T . T T T T T T T T T . T T T . T . T . . . . . . . . . . . . . . . . . . . . . . . . . . . . . . . . . . . . . . . . . . . . . . . . . . . . . . . . . . . . . . . . . . . . . . . . . . . . . . . . . . . . . . . . . . . . . . . . . . . . . . . . . . . . .    
153	A A A A A A A A A A A A . A A A A A A A A A . A A A . A . A . . . . . . . . . . . . . . . . . . . . . . . . . . . . . . . . . . . . . . . . . . . . . . . . . . . . . . . . . . . . . . . . . . . . . . . . . . . . . . . . . . . . . . . . . . . . . . . . . . . . . . . . . . . . .    
154	T T T T T T T T T T T T . T T T T T T T T T . T T T . T . T . . . . . . . . . . . . . . . . . . . . . . . . . . . . . . . . . . . . . . . . . . . . . . . . . . . . . . . . . . . . . . . . . . . . . . . . . . . . . . . . . . . . . . . . . . . . . . . . . . . . . . . . . . . . .    
155	A A A A A A A A A A A A . A A A A A A A A A . A A A . A . A . . . . . . . . . . . . . . . . . . . . . . . . . . . . . . . . . . . . . . . . . . . . . . . . . . . . . . . . . . . . . . . . . . . . . . . . . . . . . . . . . . . . . . . . . . . . . . . . . . . . . . . . . . . . .    
156	G G G G G G G G G G G G . G G G G G G G G G . G G G . G . G . . . . . . . . . . . . . . . . . . . . . . . . . . . . . . . . . . . . . . . . . . . . . . . . . . . . . . . . . . . . . . . . . . . . . . . . . . . . . . . . . . . . . . . . . . . . . . . . . . . . . . . . . . . . .    
157	A A A A A A A A A A A A . A A A A A A A A A . A A A . A . A . . . . . . . . . . . . . . . . . . . . . . . . . . . . . . . . . . . . . . . . . . . . . . . . . . . . . . . . . . . . . . . . . . . . . . . . . . . . . . . . . . . . . . . . . . . . . . . . . . . . . . . . . . . . .    
158	G G G G G G G G G G G G . G G G G G G G G G . G G G . G . G . . . . . . . . . . . . . . . . . . . . . . . . . . . . . . . . . . . . . . . . . . . . . . . . . . . . . . . . . . . . . . . . . . . . . . . . . . . . . . . . . . . . . . . . . . . . . . . . . . . . . . . . . . . . .    
159	T T T T T T T T T T T T . T T T T T T T T T . T T T . T . T . . . . . . . . . . . . . . . . . . . . . . . . . . . . . . . . . . . . . . . . . . . . . . . . . . . . . . . . . . . . . . . . . . . . . . . . . . . . . . . . . . . . . . . . . . . . . . . . . . . . . . . . . . . . .    
160	T T T T T T T T T T T T . T T T T T T T T T . T T T . T . T . . . . . . . . . . . . . . . . . . . . . . . . . . . . . . . . . . . . . . . . . . . . . . . . . . . . . . . . . . . . . . . . . . . . . . . . . . . . . . . . . . . . . . . . . . . . . . . . . . . . . . . . . . . . .    
161	T T T T T T T T T T T T . T T T T T T T T T . T T T . T . T . . . . . . . . . . . . . . . . . . . . . . . . . . . . . . . . . . . . . . . . . . . . . . . . . . . . . . . . . . . . . . . . . . . . . . . . . . . . . . . . . . . . . . . . . . . . . . . . . . . . . . . . . . . . .    
162	A A A A A A A A A A A A . A A A A A A A A A . A A A . A . A . . . . . . . . . . . . . . . . . . . . . . . . . . . . . . . . . . . . . . . . . . . . . . . . . . . . . . . . . . . . . . . . . . . . . . . . . . . . . . . . . . . . . . . . . . . . . . . . . . . . . . . . . . . . .    
163	T T T T T T T T T T T T . T T T T T T T T T . T T T . T . T . . . . . . . . . . . . . . . . . . . . . . . . . . . . . . . . . . . . . . . . . . . . . . . . . . . . . . . . . . . . . . . . . . . . . . . . . . . . . . . . . . . . . . . . . . . . . . . . . . . . . . . . . . . . .    
164	A A A A A A A A A A A A . A A A A A A A A A . A A A . A . A . . . . . . . . . . . . . . . . . . . . . . . . . . . . . . . . . . . . . . . . . . . . . . . . . . . . . . . . . . . . . . . . . . . . . . . . . . . . . . . . . . . . . . . . . . . . . . . . . . . . . . . . . . . . .    
165	G G G G G G G G G G G G . G G G G G G G G G . G G G . G . G . . . . . . . . . . . . . . . . . . . . . . . . . . . . . . . . . . . . . . . . . . . . . . . . . . . . . . . . . . . . . . . . . . . . . . . . . . . . . . . . . . . . . . . . . . . . . . . . . . . . . . . . . . . . .    
166	A A A A A A A A A A A A . A A A A A A A A A . A A A . A . A . . . . . . . . . . . . . . . . . . . . . . . . . . . . . . . . . . . . . . . . . . . . . . . . . . . . . . . . . . . . . . . . . . . . . . . . . . . . . . . . . . . . . . . . . . . . . . . . . . . . . . . . . . . . .    
167	C C C C C C C C C C C C . C C C C C C C C C . C C C . C . C . . . . . . . . . . . . . . . . . . . . . . . . . . . . . . . . . . . . . . . . . . . . . . . . . . . . . . . . . . . . . . . . . . . . . . . . . . . . . . . . . . . . . . . . . . . . . . . . . . . . . . . . . . . . .    
168	T T T T T T T T T T T T . T T T T T T T T T . T T T . T . T . . . . . . . . . . . . . . . . . . . . . . . . . . . . . . . . . . . . . . . . . . . . . . . . . . . . . . . . . . . . . . . . . . . . . . . . . . . . . . . . . . . . . . . . . . . . . . . . . . . . . . . . . . . . .    
169	C C C C C C C C C C C C . C C C C C C C C C . C C C . C . C . . . . . . . . . . . . . . . . . . . . . . . . . . . . . . . . . . . . . . . . . . . . . . . . . . . . . . . . . . . . . . . . . . . . . . . . . . . . . . . . . . . . . . . . . . . . . . . . . . . . . . . . . . . . .    
170	T T T T T T T T T T T T . T T T T T T T T T . T T T . T . T . . . . . . . . . . . . . . . . . . . . . . . . . . . . . . . . . . . . . . . . . . . . . . . . . . . . . . . . . . . . . . . . . . . . . . . . . . . . . . . . . . . . . . . . . . . . . . . . . . . . . . . . . . . . .    
171	T T T T T T T T T T T T . T T T T T T T T T . T T T . T . T . . . . . . . . . . . . . . . . . . . . . . . . . . . . . . . . . . . . . . . . . . . . . . . . . . . . . . . . . . . . . . . . . . . . . . . . . . . . . . . . . . . . . . . . . . . . . . . . . . . . . . . . . . . . .    
172	A A A A A A A A A A A A . A A A A A A A A A . A A A . A . A . . . . . . . . . . . . . . . . . . . . . . . . . . . . . . . . . . . . . . . . . . . . . . . . . . . . . . . . . . . . . . . . . . . . . . . . . . . . . . . . . . . . . . . . . . . . . . . . . . . . . . . . . . . . .    
173	T T T T T T T T T T T T . T T T T T T T T T . T T T . T . T . . . . . . . . . . . . . . . . . . . . . . . . . . . . . . . . . . . . . . . . . . . . . . . . . . . . . . . . . . . . . . . . . . . . . . . . . . . . . . . . . . . . . . . . . . . . . . . . . . . . . . . . . . . . .    
174	G G G G G G G G G G G G . G G G G G G G G G . G G G . G . G . . . . . . . . . . . . . . . . . . . . . . . . . . . . . . . . . . . . . . . . . . . . . . . . . . . . . . . . . . . . . . . . . . . . . . . . . . . . . . . . . . . . . . . . . . . . . . . . . . . . . . . . . . . . .    
175	T T T T T T T T T T T T . T T T T T T T T T . T T T . T . T . . . . . . . . . . . . . . . . . . . . . . . . . . . . . . . . . . . . . . . . . . . . . . . . . . . . . . . . . . . . . . . . . . . . . . . . . . . . . . . . . . . . . . . . . . . . . . . . . . . . . . . . . . . . .    
176	T T T T T T T T T T T T . T T T T T T T T T . T T T . T . T . . . . . . . . . . . . . . . . . . . . . . . . . . . . . . . . . . . . . . . . . . . . . . . . . . . . . . . . . . . . . . . . . . . . . . . . . . . . . . . . . . . . . . . . . . . . . . . . . . . . . . . . . . . . .    
177	T T T T T T T T T T T T . T T T T T T T T T . T T T . T . T . . . . . . . . . . . . . . . . . . . . . . . . . . . . . . . . . . . . . . . . . . . . . . . . . . . . . . . . . . . . . . . . . . . . . . . . . . . . . . . . . . . . . . . . . . . . . . . . . . . . . . . . . . . . .    
178	T T T T T T T T T T T T . T T T T T T T T T . T T T . T . T . . . . . . . . . . . . . . . . . . . . . . . . . . . . . . . . . . . . . . . . . . . . . . . . . . . . . . . . . . . . . . . . . . . . . . . . . . . . . . . . . . . . . . . . . . . . . . . . . . . . . . . . . . . . .    
179	C C C C C C C C C C C C . C C C C C C C C C . C C C . C . C C . . . . . . . . . . . . . . . . . . . . . . . . . . . . . . . . . . . . . . . . . . . . . . . . . . . . . . . . . . . . . . . . . . . . . . . . . . . . . . . . . . . . . . . . . . . . . . . . . . . . . . . . . . . .    
180	A A A A A A A A A A A A . A A A A A A A A A . A A A . A . A A . . . . . . . . . . . . . . . . . . . . . . . . . . . . . . . . . . . . . . . . . . . . . . . . . . . . . . . . . . . . . . . . . . . . . . . . . . . . . . . . . . . . . . . . . . . . . . . . . . . . . . . . . . . .    
181	A A A A A A A A A A A A . A A A A A A A A A . A A A . A . A A . . . . . . . . . . . . . . . . . . . . . . . . . . . . . . . . . . . . . . . . . . . . . . . . . . . . . . . . . . . . . . . . . . . . . . . . . . . . . . . . . . . . . . . . . . . . . . . . . . . . . . . . . . . .    
182	T T T T T T T T T T T T . T T T T T T T T T . T T T . T . T T . . . . . . . . . . . . . . . . . . . . . . . . . . . . . . . . . . . . . . . . . . . . . . . . . . . . . . . . . . . . . . . . . . . . . . . . . . . . . . . . . . . . . . . . . . . . . . . . . . . . . . . . . . . .    
183	A A A A A A A A A A A A . A A A A A A A A A . A A A . A . A A . . . . . . . . . . . . . . . . . . . . . . . . . . . . . . . . . . . . . . . . . . . . . . . . . . . . . . . . . . . . . . . . . . . . . . . . . . . . . . . . . . . . . . . . . . . . . . . . . . . . . . . . . . . .    
184	A A A A A A A A A A A A . A A A A A A A A A . A A A . A . A A . . . . . . . . . . . . . . . . . . . . . . . . . . . . . . . . . . . . . . . . . . . . . . . . . . . . . . . . . . . . . . . . . . . . . . . . . . . . . . . . . . . . . . . . . . . . . . . . . . . . . . . . . . . .    
185	G G G G G G G G G G G G . G G G G G G G G G . G G G . G . G G . . . . . . . . . . . . . . . . . . . . . . . . . . . . . . . . . . . . . . . . . . . . . . . . . . . . . . . . . . . . . . . . . . . . . . . . . . . . . . . . . . . . . . . . . . . . . . . . . . . . . . . . . . . .    
186	G G G G G G G G G G G G . G G G G G G G G G . G G G . G . G G . . . . . . . . . . . . . . . . . . . . . . . . . . . . . . . . . . . . . . . . . . . . . . . . . . . . . . . . . . . . . . . . . . . . . . . . . . . . . . . . . . . . . . . . . . . . . . . . . . . . . . . . . . . .    
187	C C C C C C C C C C C C . C C C C C C C C C C C C C . C . C C . . . . . . . . . . . . . . . . . . . . . . . . . . . . . . . . . . . . . . . . . . . . . . . . . . . . . . . . . . . . . . . . . . . . . . . . . . . . . . . . . . . . . . . . . . . . . . . . . . . . . . . . . . . .    
188	T T T T T T T T T T T T . T T T T T T T T T T T T T . T . T T . . . . . . . . . . . . . . . . . . . . . . . . . . . . . . . . . . . . . . . . . . . . . . . . . . . . . . . . . . . . . . . . . . . . . . . . . . . . . . . . . . . . . . . . . . . . . . . . . . . . . . . . . . . .    
189	G G G G G G G G G G G G . G G G G G G G G G G G G G . G . G G . . . . . . . . . . . . . . . . . . . . . . . . . . . . . . . . . . . . . . . . . . . . . . . . . . . . . . . . . . . . . . . . . . . . . . . . . . . . . . . . . . . . . . . . . . . . . . . . . . . . . . . . . . . .    
190	. A A A A A A A A A A A . A A A A A A A A A A A A A . A . A A . . . . . . . . . . . . . . . . . . . . . . . . . . . . . . . . . . . . . . . . . . . . . . . . . . . . . . . . . . . . . . . . . . . . . . . . . . . . . . . . . . . . . . . . . . . . . . . . . . . . . . . . . . . .    
191	. A A A A A A A A A A A . A A A A A A A A A A A A A . A . A A . . . . . . . . . . . . . . . . . . . . . . . . . . . . . . . . . . . . . . . . . . . . . . . . . . . . . . . . . . . . . . . . . . . . . . . . . . . . . . . . . . . . . . . . . . . . . . . . . . . . . . . . . . . .    
192	. T T T T T T T T T T T . T T T T T T T T T T T T T . T . T T . . . . . . . . . . . . . . . . . . . . . . . . . . . . . . . . . . . . . . . . . . . . . . . . . . . . . . . . . . . . . . . . . . . . . . . . . . . . . . . . . . . . . . . . . . . . . . . . . . . . . . . . . . . .    
193	. A A A A A A A A A A A . A A A A A A A A A A A A A . A . A A . . . . . . . . . . . . . . . . . . . . . . . . . . . . . . . . . . . . . . . . . . . . . . . . . . . . . . . . . . . . . . . . . . . . . . . . . . . . . . . . . . . . . . . . . . . . . . . . . . . . . . . . . . . .    
194	. T T T T T T T T T T T . T T T T T T T T T T T T T . T . T T . . . . . . . . . . . . . . . . . . . . . . . . . . . . . . . . . . . . . . . . . . . . . . . . . . . . . . . . . . . . . . . . . . . . . . . . . . . . . . . . . . . . . . . . . . . . . . . . . . . . . . . . . . . .    
195	. G G G G G G G G G G G . G G G G G G G G G G G G G . G . G G . . . . . . . . . . . . . . . . . . . . . . . . . . . . . . . . . . . . . . . . . . . . . . . . . . . . . . . . . . . . . . . . . . . . . . . . . . . . . . . . . . . . . . . . . . . . . . . . . . . . . . . . . . . .    
196	. T T T T T T T T T T T . T T T T T T T T T T T T T . T . T T . . . . . . . . . . . . . . . . . . . . . . . . . . . . . . . . . . . . . . . . . . . . . . . . . . . . . . . . . . . . . . . . . . . . . . . . . . . . . . . . . . . . . . . . . . . . . . . . . . . . . . . . . . . .    
197	. C C C C C C C C C C C . C C C C C C C C C C C C C . C . C C . . . . . . . . . . . . . . . . . . . . . . . . . . . . . . . . . . . . . . . . . . . . . . . . . . . . . . . . . . . . . . . . . . . . . . . . . . . . . . . . . . . . . . . . . . . . . . . . . . . . . . . . . . . .    
198	. A A A A A A A A A A A . A A A A A A A A A A A A A . A . A A . . . . . . . . . . . . . . . . . . . . . . . . . . . . . . . . . . . . . . . . . . . . . . . . . . . . . . . . . . . . . . . . . . . . . . . . . . . . . . . . . . . . . . . . . . . . . . . . . . . . . . . . . . . .    
199	. G G G G G G G G G G G . G G G G G G G G G G G G G . G . G G . . . . . . . . . . . . . . . . . . . . . . . . . . . . . . . . . . . . . . . . . . . . . . . . . . . . . . . . . . . . . . . . . . . . . . . . . . . . . . . . . . . . . . . . . . . . . . . . . . . . . . . . . . . .    
200	. A A A A A A A A A A A . A A A A A A A A A A A A A . A . A A . . . . . . . . . . . . . . . . . . . . . . . . . . . . . . . . . . . . . . . . . . . . . . . . . . . . . . . . . . . . . . . . . . . . . . . . . . . . . . . . . . . . . . . . . . . . . . . . . . . . . . . . . . . .    
201	. T T T T T T T T T T T . T T T T T T T T T T T T T . T . T T . . . . . . . . . . . . . . . . . . . . . . . . . . . . . . . . . . . . . . . . . . . . . . . . . . . . . . . . . . . . . . . . . . . . . . . . . . . . . . . . . . . . . . . . . . . . . . . . . . . . . . . . . . . .    
202	. T T T T T T T T T T T . T T T T T T T T T T T T T . T . T T . . . . . . . . . . . . . . . . . . . . . . . . . . . . . . . . . . . . . . . . . . . . . . . . . . . . . . . . . . . . . . . . . . . . . . . . . . . . . . . . . . . . . . . . . . . . . . . . . . . . . . . . . . . .    
203	. C C C C C C C C C C C . C C C C C C C C C C C C C . C . C C . . . . . . . . . . . . . . . . . . . . . . . . . . . . . . . . . . . . . . . . . . . . . . . . . . . . . . . . . . . . . . . . . . . . . . . . . . . . . . . . . . . . . . . . . . . . . . . . . . . . . . . . . . . .    
204	. A A A A A A A A A A A . A A A A A A A A A A A A A . A . A A . . . . . . . . . . . . . . . . . . . . . . . . . . . . . . . . . . . . . . . . . . . . . . . . . . . . . . . . . . . . . . . . . . . . . . . . . . . . . . . . . . . . . . . . . . . . . . . . . . . . . . . . . . . .    
205	. A A A A A A A A A A A . A A A A A A A A A A A A A . A . A A . . . . . . . . . . . . . . . . . . . . . . . . . . . . . . . . . . . . . . . . . . . . . . . . . . . . . . . . . . . . . . . . . . . . . . . . . . . . . . . . . . . . . . . . . . . . . . . . . . . . . . . . . . . .    
206	. C C C C C C C C C C C . C C C C C C C C C C C C C . C . C C . . . . . . . . . . . . . . . . . . . . . . . . . . . . . . . . . . . . . . . . . . . . . . . . . . . . . . . . . . . . . . . . . . . . . . . . . . . . . . . . . . . . . . . . . . . . . . . . . . . . . . . . . . . .    
207	. A A A A A A A A A A A . A A A A A A A A A A A A A . A . A A . . . . . . . . . . . . . . . . . . . . . . . . . . . . . . . . . . . . . . . . . . . . . . . . . . . . . . . . . . . . . . . . . . . . . . . . . . . . . . . . . . . . . . . . . . . . . . . . . . . . . . . . . . . .    
208	. G G G G G G G G G G G . G G G G G G G G G G G G G . G . G G . . . . . . . . . . . . . . . . . . . . . . . . . . . . . . . . . . . . . . . . . . . . . . . . . . . . . . . . . . . . . . . . . . . . . . . . . . . . . . . . . . . . . . . . . . . . . . . . . . . . . . . . . . . .    
209	. C C C C C C C C C C C . C C C C C C C C C C C C C . C . C C . . . . . . . . . . . . . . . . . . . . . . . . . . . . . . . . . . . . . . . . . . . . . . . . . . . . . . . . . . . . . . . . . . . . . . . . . . . . . . . . . . . . . . . . . . . . . . . . . . . . . . . . . . . .    
210	. A A A A A A A A A A A . A A A A A A A A A A A A A . A . A A . . . . . . . . . . . . . . . . . . . . . . . . . . . . . . . . . . . . . . . . . . . . . . . . . . . . . . . . . . . . . . . . . . . . . . . . . . . . . . . . . . . . . . . . . . . . . . . . . . . . . . . . . . . .    
211	. C C C C C C C C C C C . C C C C C C C C C C C C C . C . C C . . . . . . . . . . . . . . . . . . . . . . . . . . . . . . . . . . . . . . . . . . . . . . . . . . . . . . . . . . . . . . . . . . . . . . . . . . . . . . . . . . . . . . . . . . . . . . . . . . . . . . . . . . . .    
212	. T T T T T T T T T T T . T T T T T T T T T T T T T . T . T T . . . . . . . . . . . . . . . . . . . . . . . . . . . . . . . . . . . . . . . . . . . . . . . . . . . . . . . . . . . . . . . . . . . . . . . . . . . . . . . . . . . . . . . . . . . . . . . . . . . . . . . . . . . .    
213	. G G G G G G G G G G G . G G G G G G G G G G G G G . G . G G . . . . . . . . . . . . . . . . . . . . . . . . . . . . . . . . . . . . . . . . . . . . . . . . . . . . . . . . . . . . . . . . . . . . . . . . . . . . . . . . . . . . . . . . . . . . . . . . . . . . . . . . . . . .    
214	. T T T T T T T T T T T . T T T T T T T T T T T T T . T . T T . . . . . . . . . . . . . . . . . . . . . . . . . . . . . . . . . . . . . . . . . . . . . . . . . . . . . . . . . . . . . . . . . . . . . . . . . . . . . . . . . . . . . . . . . . . . . . . . . . . . . . . . . . . .    
215	. G G G G G G G G G G G . G G G G G G G G G G G G G . G . G G . . . . . . . . . . . . . . . . . . . . . . . . . . . . . . . . . . . . . . . . . . . . . . . . . . . . . . . . . . . . . . . . . . . . . . . . . . . . . . . . . . . . . . . . . . . . . . . . . . . . . . . . . . . .    
216	. G G G G G G G G G G G . G G G G G G G G G G G G G . G . G G . . . . . . . . . . . . . . . . . . . . . . . . . . . . . . . . . . . . . . . . . . . . . . . . . . . . . . . . . . . . . . . . . . . . . . . . . . . . . . . . . . . . . . . . . . . . . . . . . . . . . . . . . . . .    
217	. G G G G G G G G G G G . G G G G G G G G G G G G G . G . G G . . . . . . . . . . . . . . . . . . . . . . . . . . . . . . . . . . . . . . . . . . . . . . . . . . . . . . . . . . . . . . . . . . . . . . . . . . . . . . . . . . . . . . . . . . . . . . . . . . . . . . . . . . . .    
218	. G G G G G G G G G G G . G G G G G G G G G G G G G . G . G G . . . . . . . . . . . . . . . . . . . . . . . . . . . . . . . . . . . . . . . . . . . . . . . . . . . . . . . . . . . . . . . . . . . . . . . . . . . . . . . . . . . . . . . . . . . . . . . . . . . . . . . . . . . .    
219	. A A A A A A A A A A A . A A A A A A A A A A A A A . A . A A . . . . . . . . . . . . . . . . . . . . . . . . . . . . . . . . . . . . . . . . . . . . . . . . . . . . . . . . . . . . . . . . . . . . . . . . . . . . . . . . . . . . . . . . . . . . . . . . . . . . . . . . . . . .    
220	. A A A A A A A A A A A . A A A A A A A A A A A A A . A . A A . . . . . . . . . . . . . . . . . . . . . . . . . . . . . . . . . . . . . . . . . . . . . . . . . . . . . . . . . . . . . . . . . . . . . . . . . . . . . . . . . . . . . . . . . . . . . . . . . . . . . . . . . . . .    
221	. G G G G G G G G G G G . G G G G G G G G G G G G G . G . G G . . . . . . . . . . . . . . . . . . . . . . . . . . . . . . . . . . . . . . . . . . . . . . . . . . . . . . . . . . . . . . . . . . . . . . . . . . . . . . . . . . . . . . . . . . . . . . . . . . . . . . . . . . . .    
222	. T T T T T T T T T T T . T T T T T T T T T T T T T . T . T T . . . . . . . . . . . . . . . . . . . . . . . . . . . . . . . . . . . . . . . . . . . . . . . . . . . . . . . . . . . . . . . . . . . . . . . . . . . . . . . . . . . . . . . . . . . . . . . . . . . . . . . . . . . .    
223	. A A A A A A A A A A A . A A A A A A A A A A A A A . A . A A . . . . . . . . . . . . . . . . . . . . . . . . . . . . . . . . . . . . . . . . . . . . . . . . . . . . . . . . . . . . . . . . . . . . . . . . . . . . . . . . . . . . . . . . . . . . . . . . . . . . . . . . . . . .    
224	. T T T T T T T T T T T . T T T T T T T T T T T T T . T . T T . . . . . . . . . . . . . . . . . . . . . . . . . . . . . . . . . . . . . . . . . . . . . . . . . . . . . . . . . . . . . . . . . . . . . . . . . . . . . . . . . . . . . . . . . . . . . . . . . . . . . . . . . . . .    
225	. G G G G G G G G G G G . G G G G G G G G G G G G G . G . G G . . . . . . . . . . . . . . . . . . . . . . . . . . . . . . . . . . . . . . . . . . . . . . . . . . . . . . . . . . . . . . . . . . . . . . . . . . . . . . . . . . . . . . . . . . . . . . . . . . . . . . . . . . . .    
226	. T T T T T T T T T T T . T T T T T T T T T T T T T . T . T T . . . . . . . . . . . . . . . . . . . . . . . . . . . . . . . . . . . . . . . . . . . . . . . . . . . . . . . . . . . . . . . . . . . . . . . . . . . . . . . . . . . . . . . . . . . . . . . . . . . . . . . . . . . .    
227	. T T T T T T T T T T T . T T T T T T T T T T T T T . T . T T . . . . . . . . . . . . . . . . . . . . . . . . . . . . . . . . . . . . . . . . . . . . . . . . . . . . . . . . . . . . . . . . . . . . . . . . . . . . . . . . . . . . . . . . . . . . . . . . . . . . . . . . . . . .    
228	. G G G G G G G G G G G . G G G G G G G G G G G G G . G . G G . . . . . . . . . . . . . . . . . . . . . . . . . . . . . . . . . . . . . . . . . . . . . . . . . . . . . . . . . . . . . . . . . . . . . . . . . . . . . . . . . . . . . . . . . . . . . . . . . . . . . . . . . . . .    
229	. G G G G G G G G G G G . G G G G G G G G G G G G G . G . G G . . . . . . . . . . . . . . . . . . . . . . . . . . . . . . . . . . . . . . . . . . . . . . . . . . . . . . . . . . . . . . . . . . . . . . . . . . . . . . . . . . . . . . . . . . . . . . . . . . . . . . . . . . . .    
230	. A A A A A A A A A A A . A A A A A A A A A A A A A . A . A A . . . . . . . . . . . . . . . . . . . . . . . . . . . . . . . . . . . . . . . . . . . . . . . . . . . . . . . . . . . . . . . . . . . . . . . . . . . . . . . . . . . . . . . . . . . . . . . . . . . . . . . . . . . .    
231	. T T T T T T T T T T T . T T T T T T T T T T T T T . T . T T . . . . . . . . . . . . . . . . . . . . . . . . . . . . . . . . . . . . . . . . . . . . . . . . . . . . . . . . . . . . . . . . . . . . . . . . . . . . . . . . . . . . . . . . . . . . . . . . . . . . . . . . . . . .    
232	. A A A A A A A A A A A . A A A A A A A A A A A A A . A . A A . . . . . . . . . . . . . . . . . . . . . . . . . . . . . . . . . . . . . . . . . . . . . . . . . . . . . . . . . . . . . . . . . . . . . . . . . . . . . . . . . . . . . . . . . . . . . . . . . . . . . . . . . . . .    
233	. C C C C C C C C C C C . C C C C C C C C C C C C C . C . C C . . . . . . . . . . . . . . . . . . . . . . . . . . . . . . . . . . . . . . . . . . . . . . . . . . . . . . . . . . . . . . . . . . . . . . . . . . . . . . . . . . . . . . . . . . . . . . . . . . . . . . . . . . . .    
234	. A A A A A A A A A A A . A A A A A A A A A A A A A . A . A A . . . . . . . . . . . . . . . . . . . . . . . . . . . . . . . . . . . . . . . . . . . . . . . . . . . . . . . . . . . . . . . . . . . . . . . . . . . . . . . . . . . . . . . . . . . . . . . . . . . . . . . . . . . .    
235	. C C C C C C C C C C C . C C C C C C C C C C C C C . C . C C . . . . . . . . . . . . . . . . . . . . . . . . . . . . . . . . . . . . . . . . . . . . . . . . . . . . . . . . . . . . . . . . . . . . . . . . . . . . . . . . . . . . . . . . . . . . . . . . . . . . . . . . . . . .    
236	. T T T T T T T T T T T . T T T T T T T T T T T T T . T . T T . . . . . . . . . . . . . . . . . . . . . . . . . . . . . . . . . . . . . . . . . . . . . . . . . . . . . . . . . . . . . . . . . . . . . . . . . . . . . . . . . . . . . . . . . . . . . . . . . . . . . . . . . . . .    
237	. G G G G G G G G G G G . G G G G G G G G G G G G G . G . G G . . . . . . . . . . . . . . . . . . . . . . . . . . . . . . . . . . . . . . . . . . . . . . . . . . . . . . . . . . . . . . . . . . . . . . . . . . . . . . . . . . . . . . . . . . . . . . . . . . . . . . . . . . . .    
238	. A A A A A A A A A A A . A A A A A A A A A A A A A . A . A A . . . . . . . . . . . . . . . . . . . . . . . . . . . . . . . . . . . . . . . . . . . . . . . . . . . . . . . . . . . . . . . . . . . . . . . . . . . . . . . . . . . . . . . . . . . . . . . . . . . . . . . . . . . .    
239	. G G G G G G G G G G G . G G G G G G G G G G G G G . G . G G . . . . . . . . . . . . . . . . . . . . . . . . . . . . . . . . . . . . . . . . . . . . . . . . . . . . . . . . . . . . . . . . . . . . . . . . . . . . . . . . . . . . . . . . . . . . . . . . . . . . . . . . . . . .    
240	. C C C C C C C C C C C . C C C C C C C C C C C C C . C . C C . . . . . . . . . . . . . . . . . . . . . . . . . . . . . . . . . . . . . . . . . . . . . . . . . . . . . . . . . . . . . . . . . . . . . . . . . . . . . . . . . . . . . . . . . . . . . . . . . . . . . . . . . . . .    
241	. T T T T T T T T T T T . T T T T T T T T T T T T T . T . T T . . . . . . . . . . . . . . . . . . . . . . . . . . . . . . . . . . . . . . . . . . . . . . . . . . . . . . . . . . . . . . . . . . . . . . . . . . . . . . . . . . . . . . . . . . . . . . . . . . . . . . . . . . . .    
242	. G G G G G G G G G G G . G G G G G G G G G G G G G . G . G G . . . . . . . . . . . . . . . . . . . . . . . . . . . . . . . . . . . . . . . . . . . . . . . . . . . . . . . . . . . . . . . . . . . . . . . . . . . . . . . . . . . . . . . . . . . . . . . . . . . . . . . . . . . .    
243	. G G G G G G G G G G G . G G G G G G G G G G G G G . G . G G . . . . . . . . . . . . . . . . . . . . . . . . . . . . . . . . . . . . . . . . . . . . . . . . . . . . . . . . . . . . . . . . . . . . . . . . . . . . . . . . . . . . . . . . . . . . . . . . . . . . . . . . . . . .    
244	. G G G G G G G G G G G . G G G G G G G G G G G G G . G . G G . . . . . . . . . . . . . . . . . . . . . . . . . . . . . . . . . . . . . . . . . . . . . . . . . . . . . . . . . . . . . . . . . . . . . . . . . . . . . . . . . . . . . . . . . . . . . . . . . . . . . . . . . . . .    
245	. A A A A A A A A A A A . A A A A A A A A A A A A A . A . A A . . . . . . . . . . . . . . . . . . . . . . . . . . . . . . . . . . . . . . . . . . . . . . . . . . . . . . . . . . . . . . . . . . . . . . . . . . . . . . . . . . . . . . . . . . . . . . . . . . . . . . . . . . . .    
246	. G G G G G G G G G G G . G G G G G G G G G G G G G . G . G G . . . . . . . . . . . . . . . . . . . . . . . . . . . . . . . . . . . . . . . . . . . . . . . . . . . . . . . . . . . . . . . . . . . . . . . . . . . . . . . . . . . . . . . . . . . . . . . . . . . . . . . . . . . .    
247	. T T T T T T T T T T T . T T T T T T T T T T T T T . T . T T . . . . . . . . . . . . . . . . . . . . . . . . . . . . . . . . . . . . . . . . . . . . . . . . . . . . . . . . . . . . . . . . . . . . . . . . . . . . . . . . . . . . . . . . . . . . . . . . . . . . . . . . . . . .    
248	. . G G G G G G G G G G . G G G G G G G G G G G G G . G . G G . . . . . . . . . . . . . . . . . . . . . . . . . . . . . . . . . . . . . . . . . . . . . . . . . . . . . . . . . . . . . . . . . . . . . . . . . . . . . . . . . . . . . . . . . . . . . . . . . . . . . . . . . . . .    
249	. . A A A A A A A A A A . A A A A A A A A A A A A A . A . A A . . . . . . . . . . . . . . . . . . . . . . . . . . . . . . . . . . . . . . . . . . . . . . . . . . . . . . . . . . . . . . . . . . . . . . . . . . . . . . . . . . . . . . . . . . . . . . . . . . . . . . . . . . . .    
250	. . A A A A A A A A A A . A A A A A A A A A A A A A . A . A A . . . . . . . . . . . . . . . . . . . . . . . . . . . . . . . . . . . . . . . . . . . . . . . . . . . . . . . . . . . . . . . . . . . . . . . . . . . . . . . . . . . . . . . . . . . . . . . . . . . . . . . . . . . .    
251	. . G G G G G G G G G G . G G G G G G G G G G G G G . G . G G . . . . . . . . . . . . . . . . . . . . . . . . . . . . . . . . . . . . . . . . . . . . . . . . . . . . . . . . . . . . . . . . . . . . . . . . . . . . . . . . . . . . . . . . . . . . . . . . . . . . . . . . . . . .    
252	. . A A A A A A A A A A . A A A A A A A A A A A A A . A . A A . . . . . . . . . . . . . . . . . . . . . . . . . . . . . . . . . . . . . . . . . . . . . . . . . . . . . . . . . . . . . . . . . . . . . . . . . . . . . . . . . . . . . . . . . . . . . . . . . . . . . . . . . . . .    
253	. . A A A A A A A A A A . A A A A A A A A A A A A A . A . A A . . . . . . . . . . . . . . . . . . . . . . . . . . . . . . . . . . . . . . . . . . . . . . . . . . . . . . . . . . . . . . . . . . . . . . . . . . . . . . . . . . . . . . . . . . . . . . . . . . . . . . . . . . . .    
254	. . T T T T T T T T T T . T T T T T T T T T T T T T . T . T T . . . . . . . . . . . . . . . . . . . . . . . . . . . . . . . . . . . . . . . . . . . . . . . . . . . . . . . . . . . . . . . . . . . . . . . . . . . . . . . . . . . . . . . . . . . . . . . . . . . . . . . . . . . .    
255	. . G G G G G G G G G G . G G G G G G G G G G G G G . G . G G . . . . . . . . . . . . . . . . . . . . . . . . . . . . . . . . . . . . . . . . . . . . . . . . . . . . . . . . . . . . . . . . . . . . . . . . . . . . . . . . . . . . . . . . . . . . . . . . . . . . . . . . . . . .    
256	. . C C C C C C C C C C . C C C C C C C C C C C C C . C . C C . . . . . . . . . . . . . . . . . . . . . . . . . . . . . . . . . . . . . . . . . . . . . . . . . . . . . . . . . . . . . . . . . . . . . . . . . . . . . . . . . . . . . . . . . . . . . . . . . . . . . . . . . . . .    
257	. . A A A A A A A A A A . A A A A A A A A A A A A A . A . A A . . . . . . . . . . . . . . . . . . . . . . . . . . . . . . . . . . . . . . . . . . . . . . . . . . . . . . . . . . . . . . . . . . . . . . . . . . . . . . . . . . . . . . . . . . . . . . . . . . . . . . . . . . . .    
258	. . G G G G G G G G G G . G G G G G G G G G G G G G . G . G G . . . . . . . . . . . . . . . . . . . . . . . . . . . . . . . . . . . . . . . . . . . . . . . . . . . . . . . . . . . . . . . . . . . . . . . . . . . . . . . . . . . . . . . . . . . . . . . . . . . . . . . . . . . .    
259	. . A A A A A A A A A A . A A A A A A A A A A A A A . A . A A . . . . . . . . . . . . . . . . . . . . . . . . . . . . . . . . . . . . . . . . . . . . . . . . . . . . . . . . . . . . . . . . . . . . . . . . . . . . . . . . . . . . . . . . . . . . . . . . . . . . . . . . . . . .    
260	. . A A A A A A A A A A . A A A A A A A A A A A A A . A . A A . . . . . . . . . . . . . . . . . . . . . . . . . . . . . . . . . . . . . . . . . . . . . . . . . . . . . . . . . . . . . . . . . . . . . . . . . . . . . . . . . . . . . . . . . . . . . . . . . . . . . . . . . . . .    
261	. . G G G G G G G G G G . G G G G G G G G G G G G G . G . G G . . . . . . . . . . . . . . . . . . . . . . . . . . . . . . . . . . . . . . . . . . . . . . . . . . . . . . . . . . . . . . . . . . . . . . . . . . . . . . . . . . . . . . . . . . . . . . . . . . . . . . . . . . . .    
262	. . C C C C C C C C C C . C C C C C C C C C C C C C . C . C C . . . . . . . . . . . . . . . . . . . . . . . . . . . . . . . . . . . . . . . . . . . . . . . . . . . . . . . . . . . . . . . . . . . . . . . . . . . . . . . . . . . . . . . . . . . . . . . . . . . . . . . . . . . .    
263	. . C C C C C C C C C C . C C C C C C C C C C C C C . C . C C . . . . . . . . . . . . . . . . . . . . . . . . . . . . . . . . . . . . . . . . . . . . . . . . . . . . . . . . . . . . . . . . . . . . . . . . . . . . . . . . . . . . . . . . . . . . . . . . . . . . . . . . . . . .    
264	. . T T T T T T T T T T . T T T T T T T T T T T T T . T . T T . . . T . . . . . . . . . . . . . . . . . . . . . . . . . . . . . . . . . . . . . . . . . . . . . . . . . . . . . . . . . . . . . . . . . . . . . . . . . . . . . . . . . . . . . . . . . . . . . . . . . . . . . . . .    
265	. . G G G G G G G G G G . G G G G G G G G G G G G G . G . G G . . . G . . . . . . . . . . . . . . . . . . . . . . . . . . . . . . . . . . . . . . . . . . . . . . . . . . . . . . . . . . . . . . . . . . . . . . . . . . . . . . . . . . . . . . . . . . . . . . . . . . . . . . . .    
266	. . G G G G G G G G G G . G G G G G G G G G G G G G . G . G G . . . G . . . . . . . . . . . . . . . . . . . . . . . . . . . . . . . . . . . . . . . . . . . . . . . . . . . . . . . . . . . . . . . . . . . . . . . . . . . . . . . . . . . . . . . . . . . . . . . . . . . . . . . .    
267	. . A A A A A A A A A A . A A A A A A A A A A A A A . A . A A . . . A . . . . . . . . . . . . . . . . . . . . . . . . . . . . . . . . . . . . . . . . . . . . . . . . . . . . . . . . . . . . . . . . . . . . . . . . . . . . . . . . . . . . . . . . . . . . . . . . . . . . . . . .    
268	. . A A A A A A A A A A . A A A A A A A A A A A A A . A . A A . . . A . . . . . . . . . . . . . . . . . . . . . . . . . . . . . . . . . . . . . . . . . . . . . . . . . . . . . . . . . . . . . . . . . . . . . . . . . . . . . . . . . . . . . . . . . . . . . . . . . . . . . . . .    
269	. . C C C C C C C C C C . C C C C C C C C C C C C C . C . C C . . . C . . . . . . . . . . . . . . . . . . . . . . . . . . . . . . . . . . . . . . . . . . . . . . . . . . . . . . . . . . . . . . . . . . . . . . . . . . . . . . . . . . . . . . . . . . . . . . . . . . . . . . . .    
270	. . . A A A A A A A A A . A A A A A A A A A A A A A . A . A A . . . A . . . . . . . . . . . . . . . . . . . . . . . . . . . . . . . . . . . . . . . . . . . . . . . . . . . . . . . . . . . . . . . . . . . . . . . . . . . . . . . . . . . . . . . . . . . . . . . . . . . . . . . .    
271	. . . A A A A A A A A A . A A A A A A A A A A A A A . A . A A . . . A . . . . . . . . . . . . . . . . . . . . . . . . . . . . . . . . . . . . . . . . . . . . . . . . . . . . . . . . . . . . . . . . . . . . . . . . . . . . . . . . . . . . . . . . . . . . . . . . . . . . . . . .    
272	. . . A A A A A A A A A . A A A A A A A A A A A A A . A . A A . . . A . . . . . . . . . . . . . . . . . . . . . . . . . . . . . . . . . . . . . . . . . . . . . . . . . . . . . . . . . . . . . . . . . . . . . . . . . . . . . . . . . . . . . . . . . . . . . . . . . . . . . . . .    
273	. . . G G G G G G G G G . G G G G G G G G G G G G G . G . G G . . . G . . . . . . . . . . . . . . . . . . . . . . . . . . . . . . . . . . . . . . . . . . . . . . . . . . . . . . . . . . . . . . . . . . . . . . . . . . . . . . . . . . . . . . . . . . . . . . . . . . . . . . . .    
274	. . . G G G G G G G G G . G G G G G G G G G G G G G . G . G G . . . G . . . . . . . . . . . . . . . . . . . . . . . . . . . . . . . . . . . . . . . . . . . . . . . . . . . . . . . . . . . . . . . . . . . . . . . . . . . . . . . . . . . . . . . . . . . . . . . . . . . . . . . .    
275	. . . T T T T T T T T T . T T T T T T T T T T T T T . T . T T . . . T . . . . . . . . . . . . . . . . . . . . . . . . . . . . . . . . . . . . . . . . . . . . . . . . . . . . . . . . . . . . . . . . . . . . . . . . . . . . . . . . . . . . . . . . . . . . . . . . . . . . . . . .    
276	. . . C C C C C C C C C . C C C C C C C C C C C C C . C . C C . . . C . . . . . . . . . . . . . . . . . . . . . . . . . . . . . . . . . . . . . . . . . . . . . . . . . . . . . . . . . . . . . . . . . . . . . . . . . . . . . . . . . . . . . . . . . . . . . . . . . . . . . . . .    
277	. . . C C C C C C C C C . C C C C C C C C C C C C C . C . C C . . . C . . . . . . . . . . . . . . . . . . . . . . . . . . . . . . . . . . . . . . . . . . . . . . . . . . . . . . . . . . . . . . . . . . . . . . . . . . . . . . . . . . . . . . . . . . . . . . . . . . . . . . . .    
278	. . . T T T T T T T T T . T T T T T T T T T T T T T . T . T T . . . T . . . . . . . . . . . . . . . . . . . . . . . . . . . . . . . . . . . . . . . . . . . . . . . . . . . . . . . . . . . . . . . . . . . . . . . . . . . . . . . . . . . . . . . . . . . . . . . . . . . . . . . .    
279	. . . G G G G G G G G G . G G G G G G G G G G G G G . G . G G . . . G . . . . . . . . . . . . . . . . . . . . . . . . . . . . . . . . . . . . . . . . . . . . . . . . . . . . . . . . . . . . . . . . . . . . . . . . . . . . . . . . . . . . . . . . . . . . . . . . . . . . . . . .    
280	. . . A A A A A A A A A . A A A A A A A A A A A A A . A . A A . . . A . . . . . . . . . . . . . . . . . . . . . . . . . . . . . . . . . . . . . . . . . . . . . . . . . . . . . . . . . . . . . . . . . . . . . . . . . . . . . . . . . . . . . . . . . . . . . . . . . . . . . . . .    
281	. . . G G G G G G G G G . G G G G G G G G G G G G G . G . G G . . . G . . . . . . . . . . . . . . . . . . . . . . . . . . . . . . . . . . . . . . . . . . . . . . . . . . . . . . . . . . . . . . . . . . . . . . . . . . . . . . . . . . . . . . . . . . . . . . . . . . . . . . . .    
282	. . . C C C C C C C C C . C C C C C C C C C C C C C . C . C C . . . C . . . . . . . . . . . . . . . . . . . . . . . . . . . . . . . . . . . . . . . . . . . . . . . . . . . . . . . . . . . . . . . . . . . . . . . . . . . . . . . . . . . . . . . . . . . . . . . . . . . . . . . .    
283	. . . T T T T T T T T T . T T T T T T T T T T T T T . T . T T . . . T . . . . . . . . . . . . . . . . . . . . . . . . . . . . . . . . . . . . . . . . . . . . . . . . . . . . . . . . . . . . . . . . . . . . . . . . . . . . . . . . . . . . . . . . . . . . . . . . . . . . . . . .    
284	. . . G G G G G G G G G . G G G G G G G G G G G G G . G . G G . . . G . . . . . . . . . . . . . . . . . . . . . . . . . . . . . . . . . . . . . . . . . . . . . . . . . . . . . . . . . . . . . . . . . . . . . . . . . . . . . . . . . . . . . . . . . . . . . . . . . . . . . . . .    
285	. . . G G G G G G G G G . G G G G G G G G G G G G G . G . G G . . . G . . . . . . . . . . . . . . . . . . . . . . . . . . . . . . . . . . . . . . . . . . . . . . . . . . . . . . . . . . . . . . . . . . . . . . . . . . . . . . . . . . . . . . . . . . . . . . . . . . . . . . . .    
286	. . . C C C C C C C C C . C C C C C C C C C C C C C . C . C C . . . C . . . . . . . . . . . . . . . . . . . . . . . . . . . . . . . . . . . . . . . . . . . . . . . . . . . . . . . . . . . . . . . . . . . . . . . . . . . . . . . . . . . . . . . . . . . . . . . . . . . . . . . .    
287	. . . T T T T T T T T T . T T T T T T T T T T T T T . T . T T . . . T . . . . . . . . . . . . . . . . . . . . . . . . . . . . . . . . . . . . . . . . . . . . . . . . . . . . . . . . . . . . . . . . . . . . . . . . . . . . . . . . . . . . . . . . . . . . . . . . . . . . . . . .    
288	. . . G G G G G G G G G . G G G G G G G G G G G G G . G . G G . . . G . . . . . . . . . . . . . . . . . . . . . . . . . . . . . . . . . . . . . . . . . . . . . . . . . . . . . . . . . . . . . . . . . . . . . . . . . . . . . . . . . . . . . . . . . . . . . . . . . . . . . . . .    
289	. . . T T T T C T T T T . T T T T T T T T T T T T T . T . T T . . . T . . . . . . . . . . . . . . . . . . . . . . . . . . . . . . . . . . . . . . . . . . . . . . . . . . . . . . . . . . . . . . . . . . . . . . . . . . . . . . . . . . . . . . . . . . . . . . . . . . . . . . . .    
290	. . . A A A A A A A A A . A A A A A A A A A A A A A . A . A A . . . A . . . . . . . . . . . . . . . . . . . . . . . . . . . . . . . . . . . . . . . . . . . . . . . . . . . . . . . . . . . . . . . . . . . . . . . . . . . . . . . . . . . . . . . . . . . . . . . . . . . . . . . .    
291	. . . G G G G G G G G G . G G G G G G G G G G G G G . G . G G . . . G . . . . . . . . . . . . . . . . . . . . . . . . . . . . . . . . . . . . . . . . . . . . . . . . . . . . . . . . . . . . . . . . . . . . . . . . . . . . . . . . . . . . . . . . . . . . . . . . . . . . . . . .    
292	. . . A A A A A A A A A . A A A A A A A A A A A A A . A . A A . . . A . . . . . . . . . . . . . . . . . . . . . . . . . . . . . . . . . . . . . . . . . . . . . . . . . . . . . . . . . . . . . . . . . . . . . . . . . . . . . . . . . . . . . . . . . . . . . . . . . . . . . . . .    
293	. . . G G G G G G G G G . G G G G G G G G G G G G G . G . G G . . . G . . . . . . . . . . . . . . . . . . . . . . . . . . . . . . . . . . . . . . . . . . . . . . . . . . . . . . . . . . . . . . . . . . . . . . . . . . . . . . . . . . . . . . . . . . . . . . . . . . . . . . . .    
294	. . . C C C C C C C C C . C C C C C C C C C C C C C . C . C C . . . C . . . . . . . . . . . . . . . . . . . . . . . . . . . . . . . . . . . . . . . . . . . . . . . . . . . . . . . . . . . . . . . . . . . . . . . . . . . . . . . . . . . . . . . . . . . . . . . . . . . . . . . .    
295	. . . T T T T T T T T T . T T T T T T T T T T T T T . T . T T . . . T . . . . . . . . . . . . . . . . . . . . . . . . . . . . . . . . . . . . . . . . . . . . . . . . . . . . . . . . . . . . . . . . . . . . . . . . . . . . . . . . . . . . . . . . . . . . . . . . . . . . . . . .    
296	. . . A A A A A A A A A . A A A A A A A A A A A A A . A . A A . . . A . . . . . . . . . . . . . . . . . . . . . . . . . . . . . . . . . . . . . . . . . . . . . . . . . . . . . . . . . . . . . . . . . . . . . . . . . . . . . . . . . . . . . . . . . . . . . . . . . . . . . . . .    
297	. . . G G G G G G G G G . G G G G G G G G G G G G G . G . G G . . . G . . . . . . . . . . . . . . . . . . . . . . . . . . . . . . . . . . . . . . . . . . . . . . . . . . . . . . . . . . . . . . . . . . . . . . . . . . . . . . . . . . . . . . . . . . . . . . . . . . . . . . . .    
298	. . . G G G G G G G G G . G G G G G G G G G G G G G . G . G G . . . G . . . . . . . . . . . . . . . . . . . . . . . . . . . . . . . . . . . . . . . . . . . . . . . . . . . . . . . . . . . . . . . . . . . . . . . . . . . . . . . . . . . . . . . . . . . . . . . . . . . . . . . .    
299	. . . G G G G G G G G G . G G G G G G G G G G G G G . G . G G . . . G . . . . . . . . . . . . . . . . . . . . . . . . . . . . . . . . . . . . . . . . . . . . . . . . . . . . . . . . . . . . . . . . . . . . . . . . . . . . . . . . . . . . . . . . . . . . . . . . . . . . . . . .    
300	. . . G G G G G G G G G . G G G G G G G G G G G G G . G . G G . . . G . . . . . . . . . . . . . . . . . . . . . . . . . . . . . . . . . . . . . . . . . . . . . . . . . . . . . . . . . . . . . . . . . . . . . . . . . . . . . . . . . . . . . . . . . . . . . . . . . . . . . . . .    
301	. . . A A A A A A A A A . A A A A A A A A A A A A A . A . A A . . . A . . . . . . . . . . . . . . . . . . . . . . . . . . . . . . . . . . . . . . . . . . . . . . . . . . . . . . . . . . . . . . . . . . . . . . . . . . . . . . . . . . . . . . . . . . . . . . . . . . . . . . . .    
302	. . . G G G G G G G G G . G G G G G G G G G G G G G . G . G G . . . G . . . . . . . . . . . . . . . . . . . . . . . . . . . . . . . . . . . . . . . . . . . . . . . . . . . . . . . . . . . . . . . . . . . . . . . . . . . . . . . . . . . . . . . . . . . . . . . . . . . . . . . .    
303	. . . C C C C C C C C C . C C C C C C C C C C C C C . C . C C . . . C . . . . . . . . . . . . . . . . . . . . . . . . . . . . . . . . . . . . . . . . . . . . . . . . . . . . . . . . . . . . . . . . . . . . . . . . . . . . . . . . . . . . . . . . . . . . . . . . . . . . . . . .    
304	. . . T T T T T T T T T . T T T T T T T T T T T T T . T . T T . . . T . . . . . . . . . . . . . . . . . . . . . . . . . . . . . . . . . . . . . . . . . . . . . . . . . . . . . . . . . . . . . . . . . . . . . . . . . . . . . . . . . . . . . . . . . . . . . . . . . . . . . . . .    
305	. . . G G G G G G G G G . G G G G G G G G G G G G G . G . G G . . . G . . . G . . . . . . . . . . . . . . . . . . . . . . . . . . . . . . . . . . . . . . . . . . . . . . . . . . . . . . . . . . . . . . . . . . . . . . . . . . . . . . . . . . . . . . . . . . . . . . . . . . . .    
306	. . . G G G G G G G G G . G G G G G G G G G G G G G . G . G G . . . G . . . G . . . . . . . . . . . . . . . . . . . . . . . . . . . . . . . . . . . . . . . . . . . . . . . . . . . . . . . . . . . . . . . . . . . . . . . . . . . . . . . . . . . . . . . . . . . . . . . . . . . .    
307	. . . A A A A A A A A A . A A A A A A A A A A A A A . A . A A . . . A . . . A . . . . . . . . . . . . . . . . . . . . . . . . . . . . . . . . . . . . . . . . . . . . . . . . . . . . . . . . . . . . . . . . . . . . . . . . . . . . . . . . . . . . . . . . . . . . . . . . . . . .    
308	. . . G G G G G G G G G . G G G G G G G G G G G G G . G . G G . . . G . . . G . . . . . . . . . . . . . . . . . . . . . . . . . . . . . . . . . . . . . . . . . . . . . . . . . . . . . . . . . . . . . . . . . . . . . . . . . . . . . . . . . . . . . . . . . . . . . . . . . . . .    
309	. . . C C C C C C C C C . C C C C C C C C C C C C C . C . C C . . . C . . . C . . . . . . . . . . . . . . . . . . . . . . . . . . . . . . . . . . . . . . . . . . . . . . . . . . . . . . . . . . . . . . . . . . . . . . . . . . . . . . . . . . . . . . . . . . . . . . . . . . . .    
310	. . . G G G G G G G G G . G G G G G G G G G G G G G . G . G G . . . G . . . G . . . . . . . . . . . . . . . . . . . . . . . . . . . . . . . . . . . . . . . . . . . . . . . . . . . . . . . . . . . . . . . . . . . . . . . . . . . . . . . . . . . . . . . . . . . . . . . . . . . .    
311	. . . T T T T T T T T T . T T T T T T T T T T T T T . T . T T . . . T . . . T . . . . . . . . . . . . . . . . . . . . . . . . . . . . . . . . . . . . . . . . . . . . . . . . . . . . . . . . . . . . . . . . . . . . . . . . . . . . . . . . . . . . . . . . . . . . . . . . . . . .    
312	. . . T T T T T T T T T . T T T T T T T T T T T T T . T . T T . . . T . . . T . . . . . . . . . . . . . . . . . . . . . . . . . . . . . . . . . . . . . . . . . . . . . . . . . . . . . . . . . . . . . . . . . . . . . . . . . . . . . . . . . . . . . . . . . . . . . . . . . . . .    
313	. . . T T T T T T T T T . T T T T T T T T T T T T T . T . T T . . . T . . . T . . . . . . . . . . . . . . . . . . . . . . . . . . . . . . . . . . . . . . . . . . . . . . . . . . . . . . . . . . . . . . . . . . . . . . . . . . . . . . . . . . . . . . . . . . . . . . . . . . . .    
314	. . . C C C C C C C C C . C C C C C C C C C C C C C . C . C C . . . C . . . C . . . . . . . . . . . . . . . . . . . . . . . . . . . . . . . . . . . . . . . . . . . . . . . . . . . . . . . . . . . . . . . . . . . . . . . . . . . . . . . . . . . . . . . . . . . . . . . . . . . .    
315	. . . T T T T T T T T T . T T T T T T T T T T T T T . T . T T . . . T . . . T . . . . . . . . . . . . . . . . . . . . . . . . . . . . . . . . . . . . . . . . . . . . . . . . . . . . . . . . . . . . . . . . . . . . . . . . . . . . . . . . . . . . . . . . . . . . . . . . . . . .    
316	. . . G G G G G G G G G . G G G G G G G G G G G G G . G . G G . . . G . . . G . . . . . . . . . . . . . . . . . . . . . . . . . . . . . . . . . . . . . . . . . . . . . . . . . . . . . . . . . . . . . . . . . . . . . . . . . . . . . . . . . . . . . . . . . . . . . . . . . . . .    
317	. . . T T T T T T T T T . T T T T T T T T T T T T T . T . T T . . . T . . . T . . . . . . . . . . . . . . . . . . . . . . . . . . . . . . . . . . . . . . . . . . . . . . . . . . . . . . . . . . . . . . . . . . . . . . . . . . . . . . . . . . . . . . . . . . . . . . . . . . . .    
318	. . . A A A A A A A A A . A A A A A A A A A A A A A . A . A A . . . A . . . A . . . . . . . . . . . . . . . . . . . . . . . . . . . . . . . . . . . . . . . . . . . . . . . . . . . . . . . . . . . . . . . . . . . . . . . . . . . . . . . . . . . . . . . . . . . . . . . . . . . .    
319	. . . A A A A A A A A A . A A A A A A A A A A A A A . A . A A . . . A . . . A . . . . . . . . . . . . . . . . . . . . . . . . . . . . . . . . . . . . . . . . . . . . . . . . . . . . . . . . . . . . . . . . . . . . . . . . . . . . . . . . . . . . . . . . . . . . . . . . . . . .    
320	. . . G G G G G G G G G . G G G G G G G G G G G G G . G . G G . . . G . . . G . . . . . . . . . . . . . . . . . . . . . . . . . . . . . . . . . . . . . . . . . . . . . . . . . . . . . . . . . . . . . . . . . . . . . . . . . . . . . . . . . . . . . . . . . . . . . . . . . . . .    
321	. . . C C C C C C C C C . C C C C C C C C C C C C C . C . C C . . . C . . . C . . . . . . . . . . . . . . . . . . . . . . . . . . . . . . . . . . . . . . . . . . . . . . . . . . . . . . . . . . . . . . . . . . . . . . . . . . . . . . . . . . . . . . . . . . . . . . . . . . . .    
322	. . . A A A A A A A A A . A A A A A A A A A A A A A . A . A A . . . A . . . A . . . . . . . . . . . . . . . . . . . . . . . . . . . . . . . . . . . . . . . . . . . . . . . . . . . . . . . . . . . . . . . . . . . . . . . . . . . . . . . . . . . . . . . . . . . . . . . . . . . .    
323	. . . T T T T T T T T T . T T T T T T T T T T T T T . T . T T . . . T . . . T . . . . . . . . . . . . . . . . . . . . . . . . . . . . . . . . . . . . . . . . . . . . . . . . . . . . . . . . . . . . . . . . . . . . . . . . . . . . . . . . . . . . . . . . . . . . . . . . . . . .    
324	. . . A A A A A A A A A . A A A A A A A A A A A A A . A . A A . . . A . . . A . . . . . . . . . . . . . . . . . . . . . . . . . . . . . . . . . . . . . . . . . . . . . . . . . . . . . . . . . . . . . . . . . . . . . . . . . . . . . . . . . . . . . . . . . . . . . . . . . . . .    
325	. . . A A A A A A A A A . A A A A A A A A A A A A A . A . A A . . . A . . . A . . . . . . . . . . . . . . . . . . . . . . . . . . . . . . . . . . . . . . . . . . . . . . . . . . . . . . . . . . . . . . . . . . . . . . . . . . . . . . . . . . . . . . . . . . . . . . . . . . . .    
326	. . . C C C C C C C C C . C C C C C C C C C C C C C . C . C C . . . C . . . C . . . . . . . . . . . . . . . . . . . . . . . . . . . . . . . . . . . . . . . . . . . . . . . . . . . . . . . . . . . . . . . . . . . . . . . . . . . . . . . . . . . . . . . . . . . . . . . . . . . .    
327	. . . G G G G G G G G G . G G G G G G G G G G G G G . G . G G . . . G . . . G . . . . . . . . . . . . . . . . . . . . . . . . . . . . . . . . . . . . . . . . . . . . . . . . . . . . . . . . . . . . . . . . . . . . . . . . . . . . . . . . . . . . . . . . . . . . . . . . . . . .    
328	. . . C C C C C C C C C . C C C C C C C C C C C C C . C . C C . . . C . . . C . . . . . . . . . . . . . . . . . . . . . . . . . . . . . . . . . . . . . . . . . . . . . . . . . . . . . . . . . . . . . . . . . . . . . . . . . . . . . . . . . . . . . . . . . . . . . . . . . . . .    
329	. . . T T T T T T T T T . T T T T T T T T T T T T T . T . T T . . . T . . . T . . . . . . . . . . . . . . . . . . . . . . . . . . . . . . . . . . . . . . . . . . . . . . . . . . . . . . . . . . . . . . . . . . . . . . . . . . . . . . . . . . . . . . . . . . . . . . . . . . . .    
330	. . . G G G G G G G G G . G G G G G G G G G G G G G . G . G G . . . G . . . G . . . . . . . . . . . . . . . . . . . . . . . . . . . . . . . . . . . . . . . . . . . . . . . . . . . . . . . . . . . . . . . . . . . . . . . . . . . . . . . . . . . . . . . . . . . . . . . . . . . .    
331	. . . A A A A C A A A C . C A A A A A A A A C A A A . A . A A . . . A . . . A . . . . . . . . . . . . . . . . . . . . . . . . . . . . . . . . . . . . . . . . . . . . . . . . . . . . . . . . . . . . . . . . . . . . . . . . . . . . . . . . . . . . . . . . . . . . . . . . . . . .    
332	. . . T T T T T T T T T . T T T T T T T T T T T T T . T . T T . . . T . . . T . . . . . . . . . . . . . . . . . . . . . . . . . . . . . . . . . . . . . . . . . . . . . . . . . . . . . . . . . . . . . . . . . . . . . . . . . . . . . . . . . . . . . . . . . . . . . . . . . . . .    
333	. . . C C C C A C C C A . A C C C C C C C C A C C C . C . C C . . . C . . . C . . . . . . . . . . . . . . . . . . . . . . . . . . . . . . . . . . . . . . . . . . . . . . . . . . . . . . . . . . . . . . . . . . . . . . . . . . . . . . . . . . . . . . . . . . . . . . . . . . . .    
334	. . . T T T T T T T T T . T T T T T T C T T T T T T . T . T T . . . T . . . T . . . . . . . . . . . . . . . . . . . . . . . . . . . . . . . . . . . . . . . . . . . . . . . . . . . . . . . . . . . . . . . . . . . . . . . . . . . . . . . . . . . . . . . . . . . . . . . . . . . .    
335	. . . C C C C C C C C C . C C C C C C C C C C C C C . C . C C . . . C . . . C . . . . . . . . . . . . . . . . . . . . . . . . . . . . . . . . . . . . . . . . . . . . . . . . . . . . . . . . . . . . . . . . . . . . . . . . . . . . . . . . . . . . . . . . . . . . . . . . . . . .    
336	. . . C C C C T C C C T . T C C C C C C C C T C C C . C . C C . . . C . . . C . . . . . . . . . . . . . . . . . . . . . . . . . . . . . . . . . . . . . . . . . . . . . . . . . . . . . . . . . . . . . . . . . . . . . . . . . . . . . . . . . . . . . . . . . . . . . . . . . . . .    
337	. . . A A A A A A A A A . A A A A A A A A A A A A A . A . A A . . . A . . . A . . . . . . . . . . . . . . . . . . . . . . . . . . . . . . . . . . . . . . . . . . . . . . . . . . . . . . . . . . . . . . . . . . . . . . . . . . . . . . . . . . . . . . . . . . . . . . . . . . . .    
338	. . . C C C C C C C C C . C C C C C C C C C C C C C . C . C C . C . C . . . C . . . . . . . . . . . . . . . . . . . . . . . . . . . . . . . . . . . . . . . . . . . . . . . . . . . . . . . . . . . . . . . . . . . . . . . . . . . . . . . . . . . . . . . . . . . . . . . . . . . .    
339	. . . T T T T T T T T T . T T T T T T T T T T T T T . T . T T . T . T . . . T . . . . . . . . . . . . . . . . . . . . . . . . . . . . . . . . . . . . . . . . . . . . . . . . . . . . . . . . . . . . . . . . . . . . . . . . . . . . . . . . . . . . . . . . . . . . . . . . . . . .    
340	. . . A A A A A A A A A . A A A A A A A A A A A A A . A . A A . A . A . . . A . . . . . . . . . . . . . . . . . . . . . . . . . . . . . . . . . . . . . . . . . . . . . . . . . . . . . . . . . . . . . . . . . . . . . . . . . . . . . . . . . . . . . . . . . . . . . . . . . . . .    
341	. . . C C C C C C C C C . C C C C C C C C C C C C C . C . C C . C . C . . . C . . . . . . . . . . . . . . . . . . . . . . . . . . . . . . . . . . . . . . . . . . . . . . . . . . . . . . . . . . . . . . . . . . . . . . . . . . . . . . . . . . . . . . . . . . . . . . . . . . . .    
342	. . . C C C C A C C C A . A C C C C C C C C A C C C . C . C C . C . C . . . C . . . . . . . . . . . . . . . . . . . . . . . . . . . . . . . . . . . . . . . . . . . . . . . . . . . . . . . . . . . . . . . . . . . . . . . . . . . . . . . . . . . . . . . . . . . . . . . . . . . .    
343	. . . G G G G G G G G G . G G G G G G G G G G G G G . G . G G . G . G . . . G . . . . . . . . . . . . . . . . . . . . . . . . . . . . . . . . . . . . . . . . . . . . . . . . . . . . . . . . . . . . . . . . . . . . . . . . . . . . . . . . . . . . . . . . . . . . . . . . . . . .    
344	. . . C C C C G C C C T . T C C C C C C C C T C C C . C . C C . C . C . . . C . . . . . . . . . . . . . . . . . . . . . . . . . . . . . . . . . . . . . . . . . . . . . . . . . . . . . . . . . . . . . . . . . . . . . . . . . . . . . . . . . . . . . . . . . . . . . . . . . . . .    
345	. . . G G G G G G G G G . G G G G G G G G G G G G G . G . G G . G . G . . . G . . . . . . . . . . . . . . . . . . . . . . . . . . . . . . . . . . . . . . . . . . . . . . . . . . . . . . . . . . . . . . . . . . . . . . . . . . . . . . . . . . . . . . . . . . . . . . . . . . . .    
346	. . . C C C C C C C C C . C C C C C C C C C C C C C . C . C C . C C C . . . C . . . . . . . . . . . . . . . . . . . . . . . . . . . . . . . . . . . . . . . . . . . . . . . . . . . . . . . . . . . . . . . . . . . . . . . . . . . . . . . . . . . . . . . . . . . . . . . . . . . .    
347	. . . C C C C C C C C C . C C C C C C C C C C C C C . C . C C . C C C . . . C . . . . . . . . . . . . . . . . . . . . . . . . . . . . . . . . . . . . . . . . . . . . . . . . . . . . . . . . . . . . . . . . . . . . . . . . . . . . . . . . . . . . . . . . . . . . . . . . . . . .    
348	. . . A A A A A A A A A . A A A A A A A A A A A A A . A . A A . A A A . . . A . . . . . . . . . . . . . . . . . . . . . . . . . . . . . . . . . . . . . . . . . . . . . . . . . . . . . . . . . . . . . . . . . . . . . . . . . . . . . . . . . . . . . . . . . . . . . . . . . . . .    
349	. . . T T T T T T T T T . T T T T T T T T T T T T T . T . T T . T T T . . . T . . . . . . . . . . . . . . . . . . . . . . . . . . . . . . . . . . . . . . . . . . . . . . . . . . . . . . . . . . . . . . . . . . . . . . . . . . . . . . . . . . . . . . . . . . . . . . . . . . . .    
350	. . . A A A A A A A A A . A A A A A A A A A A A A A . A . A A . A A A . . . A . . . . . . . . . . . . . . . . . . . . . . . . . . . . . . . . . . . . . . . . . . . . . . . . . . . . . . . . . . . . . . . . . . . . . . . . . . . . . . . . . . . . . . . . . . . . . . . . . . . .    
351	. . . C C C C C C C C C . C C C C C C C C C C C C C . C . C C . C C C . . . C . . . . . . . . . . . . . . . . . . . . . . . . . . . . . . . . . . . . . . . . . . . . . . . . . . . . . . . . . . . . . . . . . . . . . . . . . . . . . . . . . . . . . . . . . . . . . . . . . . . .    
352	. . . T T T T T T T T T . T T T T T T T T T T T T T . T . T T . T T T . . . T . . . . . . . . . . . . . . . . . . . . . . . . . . . . . . . . . . . . . . . . . . . . . . . . . . . . . . . . . . . . . . . . . . . . . . . . . . . . . . . . . . . . . . . . . . . . . . . . . . . .    
353	. . . G G G G G G G G G . G G G G G G G G G G G G G . G . G G . G G G . . . G . . . . . . . . . . . . . . . . . . . . . . . . . . . . . . . . . . . . . . . . . . . . . . . . . . . . . . . . . . . . . . . . . . . . . . . . . . . . . . . . . . . . . . . . . . . . . . . . . . . .    
354	. . . G G G G G G G G G . G G G G G G G G G G G G G . G . G G . G G G . . . G . . . . . . . . . . . . . . . . . . . . . . . . . . . . . . . . . . . . . . . . . . . . . . . . . . . . . . . . . . . . . . . . . . . . . . . . . . . . . . . . . . . . . . . . . . . . . . . . . . . .    
355	. . . A A A A A A A A A . A A A A A A A A A A A A A . A . A A . A A A . . . A . . . . . . . . . . . . . . . . . . . . . . . . . . . . . . . . . . . . . . . . . . . . . . . . . . . . . . . . . . . . . . . . . . . . . . . . . . . . . . . . . . . . . . . . . . . . . . . . . . . .    
356	. . . C C C C C C C C C . C C C C C C C C C C C C C . C . C C . C C C . . . C . . . . . . . . . . . . . . . . . . . . . . . . . . . . . . . . . . . . . . . . . . . . . . . . . . . . . . . . . . . . . . . . . . . . . . . . . . . . . . . . . . . . . . . . . . . . . . . . . . . .    
357	. . . A A A A A A A A A . A A A A A A A A A A A A A . A . A A . A A A . . . A . . . . . . . . . . . . . . . . . . . . . . . . . . . . . . . . . . . . . . . . . . . . . . . . . . . . . . . . . . . . . . . . . . . . . . . . . . . . . . . . . . . . . . . . . . . . . . . . . . . .    
358	. . . A A A A A A A A A . A A A A A A A A A A A A A . A . A A . A A A . . . A . . . . . . . . . . . . . . . . . . . . . . . . . . . . . . . . . . . . . . . . . . . . . . . . . . . . . . . . . . . . . . . . . . . . . . . . . . . . . . . . . . . . . . . . . . . . . . . . . . . .    
359	. . . G G G G G G G G G . G G G G G G G G G G G G G . G . G G . G G G . . . G . . . . . . . . . . . . . . . . . . . . . . . . . . . . . . . . . . . . . . . . . . . . . . . . . . . . . . . . . . . . . . . . . . . . . . . . . . . . . . . . . . . . . . . . . . . . . . . . . . . .    
360	. . . A A A A A A A A A . A A A A A A A A A A A A A . A . A A . A A A . . . A . . . . . . . . . . . . . . . . . . . . . . . . . . . . . . . . . . . . . . . . . . . . . . . . . . . . . . . . . . . . . . . . . . . . . . . . . . . . . . . . . . . . . . . . . . . . . . . . . . . .    
361	. . . C C C C C C C C C . C C C C C C C C C C C C C . C . C C . C C C . . . C . . . . . . . . . . . . . . . . . . . . . . . . . . . . . . . . . . . . . . . . . . . . . . . . . . . . . . . . . . . . . . . . . . . . . . . . . . . . . . . . . . . . . . . . . . . . . . . . . . . .    
362	. . . A A A A A A A A A . A A A A A A A A A A A A A . A . A A . A A A . . . A . . . . . . . . . . . . . . . . . . . . . . . . . . . . . . . . . . . . . . . . . . . . . . . . . . . . . . . . . . . . . . . . . . . . . . . . . . . . . . . . . . . . . . . . . . . . . . . . . . . .    
363	. . . G G G G G G G G G . G G G G G G G G G G G G G . G . G G . G G G . . . G . . . . . . . . . . . . . . . . . . . . . . . . . . . . . . . . . . . . . . . . . . . . . . . . . . . . . . . . . . . . . . . . . . . . . . . . . . . . . . . . . . . . . . . . . . . . . . . . . . . .    
364	. . . T T T T T T T T T . T T T T T T T T T T T T T . T . T T . T T T . . . T . . . . . . . . . . . . . . . . . . . . . . . . . . . . . . . . . . . . . . . . . . . . . . . . . . . . . . . . . . . . . . . . . . . . . . . . . . . . . . . . . . . . . . . . . . . . . . . . . . . .    
365	. . . T T T T T T T T T . T T T T T T T T T T T T T . T . T T . T T T . . . T . . . . . . . . . . . . . . . . . . . . . . . . . . . . . . . . . . . . . . . . . . . . . . . . . . . . . . . . . . . . . . . . . . . . . . . . . . . . . . . . . . . . . . . . . . . . . . . . . . . .    
366	. . . G G G G G G G G G . G G G G G G G G G G G G G . G . G G . G G G . . . G . . . . . . . . . . . . . . . . . . . . . . . . . . . . . . . . . . . . . . . . . . . . . . . . . . . . . . . . . . . . . . . . . . . . . . . . . . . . . . . . . . . . . . . . . . . . . . . . . . . .    
367	. . . A A A A A A A A A . A A A A A A A A A A A A A . A . A A . A A A . . . A . . . . . . . . . . . . . . . . . . . . . . . . . . . . . . . . . . . . . . . . . . . . . . . . . . . . . . . . . . . . . . . . . . . . . . . . . . . . . . . . . . . . . . . . . . . . . . . . . . . .    
368	. . . G G G G G G G G G . G G G G G G G G G G G G G . G . G G . G G G . . . G . . . . . . . . . . . . . . . . . . . . . . . . . . . . . . . . . . . . . . . . . . . . . . . . . . . . . . . . . . . . . . . . . . . . . . . . . . . . . . . . . . . . . . . . . . . . . . . . . . . .    
369	. . . C C C C C C C C C . C C C C C C C C C C C C C . C . C C . C C C . . . C . . . . . . . . . . . . . . . . . . . . . . . . . . . . . . . . . . . . . . . . . . . . . . . . . . . . . . . . . . . . . . . . . . . . . . . . . . . . . . . . . . . . . . . . . . . . . . . . . . . .    
370	. . . C C C C C C C C C . C C C C C C C C C C C C C . C . C C . C C C . . . C . . . . . . . . . . . . . . . . . . . . . . . . . . . . . . . . . . . . . . . . . . . . . . . . . . . . . . . . . . . . . . . . . . . . . . . . . . . . . . . . . . . . . . . . . . . . . . . . . . . .    
371	. . . C C C C C C C C C . C C C C C C C C C C C C C . C . C C . C C C . . . C . . . . . . . . . . . . . . . . . . . . . . . . . . . . . . . . . . . . . . . . . . . . . . . . . . . . . . . . . . . . . . . . . . . . . . . . . . . . . . . . . . . . . . . . . . . . . . . . . . . .    
372	. . . C C C C C C C C C . C C C C C C C C C C C C C . C . C C . C C C . . . C . . . . . . . . . . . . . . . . . . . . . . . . . . . . . . . . . . . . . . . . . . . . . . . . . . . . . . . . . . . . . . . . . . . . . . . . . . . . . . . . . . . . . . . . . . . . . . . . . . . .    
373	. . . A A A A A A A A A . A A A A A A A A A A A A A . A . A A . A A A . . . A . . . . . . . . . . . . . . . . . . . . . . . . . . . . . . . . . . . . . . . . . . . . . . . . . . . . . . . . . . . . . . . . . . . . . . . . . . . . . . . . . . . . . . . . . . . . . . . . . . . .    
374	. . . T T T T T T T T T . T T T T T T T T T T T T T . T . T T . T T T . . . T . . . . . . . . . . . . . . . . . . . . . . . . . . . . . . . . . . . . . . . . . . . . . . . . . . . . . . . . . . . . . . . . . . . . . . . . . . . . . . . . . . . . . . . . . . . . . . . . . . . .    
375	. . . G G G G G G G G G . G G G G G G G G G G G G G . G . G G . G G G . . . G . . . . . . . . . . . . . . . . . . . . . . . . . . . . . . . . . . . . . . . . . . . . . . . . . . . . . . . . . . . . . . . . . . . . . . . . . . . . . . . . . . . . . . . . . . . . . . . . . . . .    
376	. . . T T T . T T T T T . T T T T T T T T T T T T T . T . T T . T T T . . . T . . . . . . . . . . . . . . . . . . . . . . . . . . . . . . . . . . . . . . . . . . . . . . . . . . . . . . . . . . . . . . . . . . . . . . . . . . . . . . . . . . . . . . . . . . . . . . . . . . . .    
377	. . . C C C . C C C C C . C C C C C C C C C C C C C . C . C C . C C C . . . C . . . . . . . . . . . . . . . . . . . . . . . . . . . . . . . . . . . . . . . . . . . . . . . . . . . . . . . . . . . . . . . . . . . . . . . . . . . . . . . . . . . . . . . . . . . . . . . . . . . .    
378	. . . A A A . A A A A A . A A A A A A A A A A A A A . A . A A . A A A . . . A . . . . . . . . . . . . . . . . . . . . . . . . . . . . . . . . . . . . . . . . . . . . . . . . . . . . . . . . . . . . . . . . . . . . . . . . . . . . . . . . . . . . . . . . . . . . . . . . . . . .    
379	. . . G G G . G G G G G . G G G G G G G G G G G G G . G . G G . G G G . . . G . . . . . . . . . . . . . . . . . . . . . . . . . . . . . . . . . . . . . . . . . . . . . . . . . . . . . . . . . . . . . . . . . . . . . . . . . . . . . . . . . . . . . . . . . . . . . . . . . . . .    
380	. . . A A A . A A A A A . A A A A A A A A A A A A A . A . A A . A A A . . . A . . . . . . . . . . . . . . . . . . . . . . . . . . . . . . . . . . . . . . . . . . . . . . . . . . . . . . . . . . . . . . . . . . . . . . . . . . . . . . . . . . . . . . . . . . . . . . . . . . . .    
381	. . . C C C . C C C C C . C C C C C C C C C C C C C . C . C C . C C C . . . C . . . . . . . . . . . . . . . . . . . . . . . . . . . . . . . . . . . . . . . . . . . . . . . . . . . . . . . . . . . . . . . . . . . . . . . . . . . . . . . . . . . . . . . . . . . . . . . . . . . .    
382	. . . T T T . T T T T T . T T T T T T T T T T T T T . T . T T . T T T . . . T . . . . . . . . . . . . . . . . . . . . . . . . . . . . . . . . . . . . . . . . . . . . . . . . . . . . . . . . . . . . . . . . . . . . . . . . . . . . . . . . . . . . . . . . . . . . . . . . . . . .    
383	. . . G G G . G G G G G . G G G G G G G G G G G G G . G . G G . G G G . . . G . . . . . . . . . . . . . . . . . . . . . . . . . . . . . . . . . . . . . . . . . . . . . . . . . . . . . . . . . . . . . . . . . . . . . . . . . . . . . . . . . . . . . . . . . . . . . . . . . . . .    
384	. . . A A A . A A A A A . A A A A A A A A A A A A A . A . A A . A A A . . . A . . . . . . . . . . . . . . . . . . . . . . . . . . . . . . . . . . . . . . . . . . . . . . . . . . . . . . . . . . . . . . . . . . . . . . . . . . . . . . . . . . . . . . . . . . . . . . . . . . . .    
385	. . . G G G . G G G G G . G G G G G G G G G G G G G . G . G G . G G G . . . G . . . . . . . . . . . . . . . . . . . . . . . . . . . . . . . . . . . . . . . . . . . . . . . . . . . . . . . . . . . . . . . . . . . . . . . . . . . . . . . . . . . . . . . . . . . . . . . . . . . .    
386	. . . C C C . C C C C C . C C C C C C C C C C C C C . C . C C . C C C . . . C . . . . . . . . . . . . . . . . . . . . . . . . . . . . . . . . . . . . . . . . . . . . . . . . . . . . . . . . . . . . . . . . . . . . . . . . . . . . . . . . . . . . . . . . . . . . . . . . . . . .    
387	. . . T T T . T T T T T . T T T T T T T T T T T T T . T . T T . T T T . . . T . . . . . . . . . . . . . . . . . . . . . . . . . . . . . . . . . . . . . . . . . . . . . . . . . . . . . . . . . . . . . . . . . . . . . . . . . . . . . . . . . . . . . . . . . . . . . . . . . . . .    
388	. . . C C C . C C C C C . C C C C C C C C C C C C C . C . C C . C C C . . . C . . . . . . . . . . . . . . . . . . . . . . . . . . . . . . . . . . . . . . . . . . . . . . . . . . . . . . . . . . . . . . . . . . . . . . . . . . . . . . . . . . . . . . . . . . . . . . . . . . . .    
389	. . . A A A . A A A A A . A A A A A A A A A A A A A . A . A A . A A A . . . A . . . . . . . . . . . . . . . . . . . . . . . . . . . . . . . . . . . . . . . . . . . . . . . . . . . . . . . . . . . . . . . . . . . . . . . . . . . . . . . . . . . . . . . . . . . . . . . . . . . .    
390	. . . G G G . G G G G G . G G G G G G G G G G G G G . G . G G . G G G . . . G . . . . . . . . . . . . . . . . . . . . . . . . . . . . . . . . . . . . . . . . . . . . . . . . . . . . . . . . . . . . . . . . . . . . . . . . . . . . . . . . . . . . . . . . . . . . . . . . . . . .    
391	. . . T T T . A T T T T . T T T T T T T T T T T T T . T . T T . T T T . . . T . . . . . . . . . . . . . . . . . . . . . . . . . . . . . . . . . . . . . . . . . . . . . . . . . . . . . . . . . . . . . . . . . . . . . . . . . . . . . . . . . . . . . . . . . . . . . . . . . . . .    
392	. . . G G G . G G G G G . G G G G G G G G G G G G G . G . G G . G G G . . . G . . . . . . . . . . . . . . . . . . . . . . . . . . . . . . . . . . . . . . . . . . . . . . . . . . . . . . . . . . . . . . . . . . . . . . . . . . . . . . . . . . . . . . . . . . . . . . . . . . . .    
393	. . . G G G . G G G G G . G G G G G G G G G G G G G . G . G G . G G G . . . G . . . . . . . . . . . . . . . . . . . . . . . . . . . . . . . . . . . . . . . . . . . . . . . . . . . . . . . . . . . . . . . . . . . . . . . . . . . . . . . . . . . . . . . . . . . . . . . . . . . .    
394	. . . C C C . C C C C C . C C C C C C C C C C C C C . C . C C . C C C . . . C . . . . . . . . . . . . . . . . . . . . . . . . . . . . . . . . . . . . . . . . . . . . . . . . . . . . . . . . . . . . . . . . . . . . . . . . . . . . . . . . . . . . . . . . . . . . . . . . . . . .    
395	. . . T T T . T T T T T . T T T T T T T T T T T T T . T . T T . T T T . . . T . . . . . . . . . . . . . . . . . . . . . . . . . . . . . . . . . . . . . . . . . . . . . . . . . . . . . . . . . . . . . . . . . . . . . . . . . . . . . . . . . . . . . . . . . . . . . . . . . . . .    
396	. . . C C C . C C C C C . C C C C C C C C C C C C C . C . C C . C C C . . . C . . . . . . . . . . . . . . . . . . . . . . . . . . . . . . . . . . . . . . . . . . . . . . . . . . . . . . . . . . . . . . . . . . . . . . . . . . . . . . . . . . . . . . . . . . . . . . . . . . . .    
397	. . . C C C . C C C C C . C C C C C C C C C C C C C . C . C C . C C C . . . C . . . . . . . . . . . . . . . . . . . . . . . . . . . . . . . . . . . . . . . . . . . . . . . . . . . . . . . . . . . . . . . . . . . . . . . . . . . . . . . . . . . . . . . . . . . . . . . . . . . .    
398	. . . C C C . C C C C C . C C C C C C C C C C C C C . C . C C . C C C . . . C . . . . . . . . . . . . . . . . . . . . . . . . . . . . . . . . . . . . . . . . . . . . . . . . . . . . . . . . . . . . . . . . . . . . . . . . . . . . . . . . . . . . . . . . . . . . . . . . . . . .    
399	. . . C C C . C C C C C . C C C C C C C C C C C C C . C . C C . C C C . . . C . . . . . . . . . . . . . . . . . . . . . . . . . . . . . . . . . . . . . . . . . . . . . . . . . . . . . . . . . . . . . . . . . . . . . . . . . . . . . . . . . . . . . . . . . . . . . . . . . . . .    
400	. . . C . C . C C C C C . C C C C C C C C C C C C C . C . C C . C C C . . . C . . . . . . . . . . . . . . . . . . . . . . . . . . . . . . . . . . . . . . . . . . . . . . . . . . . . . . . . . . . . . . . . . . . . . . . . . . . . . . . . . . . . . . . . . . . . . . . . . . . .    
401	. . . C . C . C C C C C . C C C C C C C C C C C C C . C . C C . C C C . . . C . . . . . . . . . . . . . . . . . . . . . . . . . . . . . . . . . . . . . . . . . . . . . . . . . . . . . . . . . . . . . . . . . . . . . . . . . . . . . . . . . . . . . . . . . . . . . . . . . . . .    
402	. . . A . A . A A A A A . A A A A A A A A A A A A A . A . A A . A A A . . . A . . . . . . . . . . . . . . . . . . . . . . . . . . . . . . . . . . . . . . . . . . . . . . . . . . . . . . . . . . . . . . . . . . . . . . . . . . . . . . . . . . . . . . . . . . . . . . . . . . . .    
403	. . . . . G . G G G G G . G G G G G G G G G G G G G . G . G G . G G G . . . G . . . . . . . . . . . . . . . . . . . . . . . . . . . . . . . . . . . . . . . . . . . . . . . . . . . . . . . . . . . . . . . . . . . . . . . . . . . . . . . . . . . . . . . . . . . . . . . . . . . .    
404	. . . . . T . T T T T T . T T T T T T T T T T T T T . T . T T . T T T . . . T . . . . . . . . . . . . . . . . . . . . . . . . . . . . . . . . . . . . . . . . . . . . . . . . . . . . . . . . . . . . . . . . . . . . . . . . . . . . . . . . . . . . . . . . . . . . . . . . . . . .    
405	. . . . . G . G G G G G . G G G G G G G G G G G G G . G . G G . G G G . . . G . . . . . . . . . . . . . . . . . . . . . . . . . . . . . . . . . . . . . . . . . . . . . . . . . . . . . . . . . . . . . . . . . . . . . . . . . . . . . . . . . . . . . . . . . . . . . . . . . . . .    
406	. . . . . G . G G G G G . G G G G G G G G G G G G G . G . G G . G G G . . . G . . . . . . . . . . . . . . . . . . . . . . . . . . . . . . . . . . . . . . . . . . . . . . . . . . . . . . . . . . . . . . . . . . . . . . . . . . . . . . . . . . . . . . . . . . . . . . . . . . . .    
407	. . . . . C . C C C C C . C C C C C C C C C C C C C . C . C C . C C C . . . C . . . . . . . . . . . . . . . . . . . . . . . . . . . . . . . . . . . . . . . . . . . . . . . . . . . . . . . . . . . . . . . . . . . . . . . . . . . . . . . . . . . . . . . . . . . . . . . . . . . .    
408	. . . . . A . A A A A A . A A A A A A A A A A A A A . A . A A . A A A . . . A . . . . . . . . . . . . . . . . . . . . . . . . . . . . . . . . . . . . . . . . . . . . . . . . . . . . . . . . . . . . . . . . . . . . . . . . . . . . . . . . . . . . . . . . . . . . . . . . . . . .    
409	. . . . . G . G G G G G . G G G G G G G G G G G G G . G . G G . G G G . . . G . . . . . . . . . . . . . . . . . . . . . . . . . . . . . . . . . . . . . . . . . . . . . . . . . . . . . . . . . . . . . . . . . . . . . . . . . . . . . . . . . . . . . . . . . . . . . . . . . . . .    
410	. . . . . A . A A A A A . A A A A A A A A A A A A A . A . A A . A A A . . . A . . . . . . . . . . . . . . . . . . . . . . . . . . . . . . . . . . . . . . . . . . . . . . . . . . . . . . . . . . . . . . . . . . . . . . . . . . . . . . . . . . . . . . . . . . . . . . . . . . . .    
411	. . . . . C . C C C C C . C C C C C C C C C C C C C . C . C C C C C C . . . C . . . . . . . . . . . . . . . . . . . . . . . . . . . . . . . . . . . . . . . . . . . . . . . . . . . . . . . . . . . . . . . . . . . . . . . . . . . . . . . . . . . . . . . . . . . . . . . . . . . .    
412	. . . . . A . A A A A A . A A A A A A A A A A A A A . A . A A A A A A . . . A . . . . . . . . . . . . . . . . . . . . . . . . . . . . . . . . . . . . . . . . . . . . . . . . . . . . . . . . . . . . . . . . . . . . . . . . . . . . . . . . . . . . . . . . . . . . . . . . . . . .    
413	. . . . . C . C C C C C C C C C C C C C C C C C C C . C . C C C C C C . . . C . . . . . . . . . . . . . . . . . . . . . . . . . . . . . . . . . . . . . . . . . . . . . . . . . . . . . . . . . . . . . . . . . . . . . . . . . . . . . . . . . . . . . . . . . . . . . . . . . . . .    
414	. . . . . C . C C C C C C C C C C C C C C C C C C C . C . C C C C C C . . . C . . . . . . . . . . . . . . . . . . . . . . . . . . . . . . . . . . . . . . . . . . . . . . . . . . . . . . . . . . . . . . . . . . . . . . . . . . . . . . . . . . . . . . . . . . . . . . . . . . . .    
415	. . . . . C . C C C C C C C C C C C C C C C C C C C . C . C C C C C C . . . C . . . . . . . . . . . . . . . . . . . . . . . . . . . . . . . . . . . . . . . . . . . . . . . . . . . . . . . . . . . . . . . . . . . . . . . . . . . . . . . . . . . . . . . . . . . . . . . . . . . .    
416	. . . . . T . T T T T T T T T T T T T T T T T T T T . T . T T T T T T . . . T . . . . . . . . . . . . . . . . . . . . . . . . . . . . . . . . . . . . . . . . . . . . . . . . . . . . . . . . . . . . . . . . . . . . . . . . . . . . . . . . . . . . . . . . . . . . . . . . . . . .    
417	. . . . . G . G G G G G G G G G G G G G G G G G G G . G . G G G G G G . . . G . . . . . . . . . . . . . . . . . . . . . . . . . . . . . . . . . . . . . . . . . . . . . . . . . . . . . . . . . . . . . . . . . . . . . . . . . . . . . . . . . . . . . . . . . . . . . . . . . . . .    
418	. . . . . G . C C C C C C C C C C C C C C C C C C C . C . C C C C C C . . . C . . . . . . . . . . . . . . . . . . . . . . . . . . . . . . . . . . . . . . . . . . . . . . . . . . . . . . . . . . . . . . . . . . . . . . . . . . . . . . . . . . . . . . . . . . . . . . . . . . . .    
419	. . . . . . . C C C C C C C C C C C C C C C C C C C . C . C C C C C C . . . C . . . . . . . . . . . . . . . . . . . . . . . . . . . . . . . . . . . . . . . . . . . . . . . . . . . . . . . . . . . . . . . . . . . . . . . . . . . . . . . . . . . . . . . . . . . . . . . . . . . .    
420	. . . . . . . A A A A A A A A A A A A A A A A A A A . A . A A A A A A . . . A . . . . . . . . . . . . . . . . . . . . . . . . . . . . . . . . . . . . . . . . . . . . . . . . . . . . . . . . . . . . . . . . . . . . . . . . . . . . . . . . . . . . . . . . . . . . . . . . . . . .    
421	. . . . . . . T T T T T T T T T T T T T T T T T T T . T . T T T T T T . . . T . . . . . . . . . . . . . . . . . . . . . . . . . . . . . . . . . . . . . . . . . . . . . . . . . . . . . . . . . . . . . . . . . . . . . . . . . . . . . . . . . . . . . . . . . . . . . . . . . . . .    
422	. . . . . . . G G G G G G G G G G G G G G G G G G G . G . G G G G G G . . . G . . . . . . . . . . . . . . . . . . . . . . . . . . . . . . . . . . . . . . . . . . . . . . . . . . . . . . . . . . . . . . . . . . . . . . . . . . . . . . . . . . . . . . . . . . . . . . . . . . . .    
423	. . . . . . . C C C C C C C C C C C C C C C C C C C . C . C C C C C C . . . C . . . . . . . . . . . . . . . . . . . . . . . . . . . . . . . . . . . . . . . . . . . . . . . . . . . . . . . . . . . . . . . . . . . . . . . . . . . . . . . . . . . . . . . . . . . . . . . . . . . .    
424	. . . . . . . T C T T T T T T T T T T T T T T T T T . T . T T T T T T . . . T . . . . . . . . . . . . . . . . . . . . . . . . . . . . . . . . . . . . . . . . . . . . . . . . . . . . . . . . . . . . . . . . . . . . . . . . . . . . . . . . . . . . . . . . . . . . . . . . . . . .    
425	. . . . . . . G G G G G G G G G G G G G G G G G G G . G . G G G G G G . . . G . . . . . . . . . . . . . . . . . . . . . . . . . . . . . . . . . . . . . . . . . . . . . . . . . . . . . . . . . . . . . . . . . . . . . . . . . . . . . . . . . . . . . . . . . . . . . . . . . . . .    
426	. . . . . . . A A A A A A A A A A A A A A A A A A A . A . A A A A A A . . . A . . . . . . . . . . . . . . . . . . . . . . . . . . . . . . . . . . . . . . . . . . . . . . . . . . . . . . . . . . . . . . . . . . . . . . . . . . . . . . . . . . . . . . . . . . . . . . . . . . . .    
427	. . . . . . . T T T T T T T T T T T T T T T T T T T . T . T T T T T T . . . T . . . . . . . . . . . . . . . . . . . . . . . . . . . . . . . . . . . . . . . . . . . . . . . . . . . . . . . . . . . . . . . . . . . . . . . . . . . . . . . . . . . . . . . . . . . . . . . . . . . .    
428	. . . . . . . G G G G G G G G G G G G G G G G G G G . G . G G G G G G . . . G . . . . . . . . . . . . . . . . . . . . . . . . . . . . . . . . . . . . . . . . . . . . . . . . . . . . . . . . . . . . . . . . . . . . . . . . . . . . . . . . . . . . . . . . . . . . . . . . . . . .    
429	. . . . . . . T T T T T T T T T T T T T T T T T T T . T . T T T T T T . . . T . . . . . . . . . . . . . . . . . . . . . . . . . . . . . . . . . . . . . . . . . . . . . . . . . . . . . . . . . . . . . . . . . . . . . . . . . . . . . . . . . . . . . . . . . . . . . . . . . . . .    
430	. . . . . . . G G G G G G G G G G G G G G G G G G G . G . G G G G G G . . . G . . . . . . . . . . . . . . . . . . . . . . . . . . . . . . . . . . . . . . . . . . . . . . . . . . . . . . . . . . . . . . . . . . . . . . . . . . . . . . . . . . . . . . . . . . . . . . . . . . . .    
431	. . . . . . . C C C C C C C C C C C C C C C C C C C . C . C C C C C C . . . C . . . . . . . . . . . . . . . . . . . . . . . . . . . . . . . . . . . . . . . . . . . . . . . . . . . . . . . . . . . . . . . . . . . . . . . . . . . . . . . . . . . . . . . . . . . . . . . . . . . .    
432	. . . . . . . A A A A A A A A A A A A A A A A A A A . A . A A A A A A . . . A . . . . . . . . . . . . . . . . . . . . . . . . . . . . . . . . . . . . . . . . . . . . . . . . . . . . . . . . . . . . . . . . . . . . . . . . . . . . . . . . . . . . . . . . . . . . . . . . . . . .    
433	. . . . . . . G G G G G G G G G G G G G G G G G G G . G . G G G G G G . . . G . . . . . . . . . . . . . . . . . . . . . . . . . . . . . . . . . . . . . . . . . . . . . . . . . . . . . . . . . . . . . . . . . . . . . . . . . . . . . . . . . . . . . . . . . . . . . . . . . . . .    
434	. . . . . . . C A C C C C C C C C C C C C C C C C C . C . C C C T C C . . . C . . . . . . . . . . . . . . . . . . . . . . . . . . . . . . . . . . . . . . . . . . . . . . . . . . . . . . . . . . . . . . . . . . . . . . . . . . . . . . . . . . . . . . . . . . . . . . . . . . . .    
435	. . . . . . . G G G G G G G G G G G G G G G G G G G . G . G G G G G G . . . G . . . . . . . . . . . . . . . . . . . . . . . . . . . . . . . . . . . . . . . . . . . . . . . . . . . . . . . . . . . . . . . . . . . . . . . . . . . . . . . . . . . . . . . . . . . . . . . . . . . .    
436	. . . . . . . C C C C C C C C C C C C C C C C C C C . C . C C C C C C . . . C . . . . . . . . . . . . . . . . . . . . . . . . . . . . . . . . . . . . . . . . . . . . . . . . . . . . . . . . . . . . . . . . . . . . . . . . . . . . . . . . . . . . . . . . . . . . . . . . . . . .    
437	. . . . . . . C C C C C C C C C C C C C C C C C C C . C . C C C C C C . . . C . . C . . . . . . . . . . . . . . . . . . . . . . . . . . . . . . . . . . . . . . . . . . . . . . . . . . . . . . . . . . . . . . . . . . . . . . . . . . . . . . . . . . . . . . . . . . . . . . . . .    
438	. . . . . . . T T T T T T T T T T T T T T T T T T T . T . T T T T T T . . . T . . T . . . . . . . . . . . . . . . . . . . . . . . . . . . . . . . . . . . . . . . . . . . . . . . . . . . . . . . . . . . . . . . . . . . . . . . . . . . . . . . . . . . . . . . . . . . . . . . . .    
439	. . . . . . . A A A A A A A A A A A A A A A A A A A . A . A A A A A A . . . A . . A . . . . . . . . . . . . . . . . . . . . . . . . . . . . . . . . . . . . . . . . . . . . . . . . . . . . . . . . . . . . . . . . . . . . . . . . . . . . . . . . . . . . . . . . . . . . . . . . .    
440	. . . . . . . C C C C C C C C C C C C C C C C C C C . C . C C C C C C . . . C . . C . . . . . . . . . . . . . . . . . . . . . . . . . . . . . . . . . . . . . . . . . . . . . . . . . . . . . . . . . . . . . . . . . . . . . . . . . . . . . . . . . . . . . . . . . . . . . . . . .    
441	. . . . . . . G G G G G G G G G G G G G G G G G G G . G . G G G G G G . . . G . . G . . . . . . . . . . . . . . . . . . . . . . . . . . . . . . . . . . . . . . . . . . . . . . . . . . . . . . . . . . . . . . . . . . . . . . . . . . . . . . . . . . . . . . . . . . . . . . . . .    
442	. . . . . . . A A A A A A A A A A A A A A A A A A A . A . A A A A A A . . . A . . A . . . . . . . . . . . . . . . . . . . . . . . . . . . . . . . . . . . . . . . . . . . . . . . . . . . . . . . . . . . . . . . . . . . . . . . . . . . . . . . . . . . . . . . . . . . . . . . . .    
443	. . . . . . . C C C C C C C C C C C C C C C C C C C . C . C C C C C C . . . C . . C . . . . . . . . . . . . . . . . . . . . . . . . . . . . . . . . . . . . . . . . . . . . . . . . . . . . . . . . . . . . . . . . . . . . . . . . . . . . . . . . . . . . . . . . . . . . . . . . .    
444	. . . . . . . T T T T T T T T T T T T T T T T T T T . T . T T T T T T . . . T . . T . . . . . . . . . . . . . . . . . . . . . . . . . . . . . . . . . . . . . . . . . . . . . . . . . . . . . . . . . . . . . . . . . . . . . . . . . . . . . . . . . . . . . . . . . . . . . . . . .    
445	. . . . . . . T T T T T T T T T T T T T T T T T T T . T . T T T T T T . . . T . . T . . . . . . . . . . . . . . . . . . . . . . . . . . . . . . . . . . . . . . . . . . . . . . . . . . . . . . . . . . . . . . . . . . . . . . . . . . . . . . . . . . . . . . . . . . . . . . . . .    
446	. . . . . . . C C C C C C C C C C C C C C C C C C C . C . C C C C C C . . . C . . C . . . . . . . . . . . . . . . . . . . . . . . . . . . . . . . . . . . . . . . . . . . . . . . . . . . . . . . . . . . . . . . . . . . . . . . . . . . . . . . . . . . . . . . . . . . . . . . . .    
447	. . . . . . . T T T T T T T T T T T T T T T T T T T . T . T T T T T T . . . T . . T . . . . . . . . . . . . . . . . . . . . . . . . . . . . . . . . . . . . . . . . . . . . . . . . . . . . . . . . . . . . . . . . . . . . . . . . . . . . . . . . . . . . . . . . . . . . . . . . .    
448	. . . . . . . A A A A A A A A A A A A A A A A A A A . A . A A A A A A . . . A . . A . . . . . . . . . . . . . . . . . . . . . . . . . . . . . . . . . . . . . . . . . . . . . . . . . . . . . . . . . . . . . . . . . . . . . . . . . . . . . . . . . . . . . . . . . . . . . . . . .    
449	. . . . . . . C G C C C C C C C C C C C C C C C C C . C . C C C C C C . . . C . . C . . . . . . . . . . . . . . . . . . . . . . . . . . . . . . . . . . . . . . . . . . . . . . . . . . . . . . . . . . . . . . . . . . . . . . . . . . . . . . . . . . . . . . . . . . . . . . . . .    
450	. . . . . . . C C C C C C C C C C C C C C C C C C C . C . C C C C C C . . . C . . C . . . . . . . . . . . . . . . . . . . . . . . . . . . . . . . . . . . . . . . . . . . . . . . . . . . . . . . . . . . . . . . . . . . . . . . . . . . . . . . . . . . . . . . . . . . . . . . . .    
451	. . . . . . . C C C C C C C C C C C C C C C C C C C . C . C C C C C C . . . C . . C . . . . . . . . . . . . . . . . . . . . . . . . . . . . . . . . . . . . . . . . . . . . . . . . . . . . . . . . . . . . . . . . . . . . . . . . . . . . . . . . . . . . . . . . . . . . . . . . .    
452	. . . . . . . C C C C C C C C C C C C C C C C C C C . C . C C C C C C . . . C . . C . . . . . . . . . . . . . . . . . . . . . . . . . . . . . . . . . . . . . . . . . . . . . . . . . . . . . . . . . . . . . . . . . . . . . . . . . . . . . . . . . . . . . . . . . . . . . . . . .    
453	. . . . . . . A A A A A A A A A A A A A A A A A A A . A . A A A A A A . . . A . . A . . . . . . . . . . . . . . . . . . . . . . . . . . . . . . . . . . . . . . . . . . . . . . . . . . . . . . . . . . . . . . . . . . . . . . . . . . . . . . . . . . . . . . . . . . . . . . . . .    
454	. . . . . . . A A A A A A A A A A A A A A A A A A A . A . A A A A A A . . . A . . A . . . . . . . . . . . . . . . . . . . . . . . . . . . . . . . . . . . . . . . . . . . . . . . . . . . . . . . . . . . . . . . . . . . . . . . . . . . . . . . . . . . . . . . . . . . . . . . . .    
455	. . . . . . . A A A A A A A A A A A A A A A A A A A . A . A A A A A A . . . A . . A . . . . . . . . . . . . . . . . . . . . . . . . . . . . . . . . . . . . . . . . . . . . . . . . . . . . . . . . . . . . . . . . . . . . . . . . . . . . . . . . . . . . . . . . . . . . . . . . .    
456	. . . . . . . C C C C C C C C C C C C C C C C C C C . C . C C C C C C . . . C . . C . . . . . . . . . . . . . . . . . . . . . . . . . . . . . . . . . . . . . . . . . . . . . . . . . . . . . . . . . . . . . . . . . . . . . . . . . . . . . . . . . . . . . . . . . . . . . . . . .    
457	. . . . . . . C C C C C C C C C C C C C C C C C C C . C . C C C C C C . . . C . . C . . . . . . . . . . . . . . . . . . . . . . . . . . . . . . . . . . . . . . . . . . . . . . . . . . . . . . . . . . . . . . . . . . . . . . . . . . . . . . . . . . . . . . . . . . . . . . . . .    
458	. . . . . . . A A A A A A A A A A A A A A A A A A A . A . A A A A A A . . . A . . A . . . . . . . . . . . . . . . . . . . . . . . . . . . . . . . . . . . . . . . . . . . . . . . . . . . . . . . . . . . . . . . . . . . . . . . . . . . . . . . . . . . . . . . . . . . . . . . . .    
459	. . . . . . . A A A A A A A A A A A A A A A A A A A . A . A A A A A A . . . A . . A . . . . . . . . . . . . . . . . . . . . . . . . . . . . . . . . . . . . . . . . . . . . . . . . . . . . . . . . . . . . . . . . . . . . . . . . . . . . . . . . . . . . . . . . . . . . . . . . .    
460	. . . . . . . T T T T T T T T T T T T T T T T T T T . T . T T T T T T . . . T . . T . . . . . . . . . . . . . . . . . . . . . . . . . . . . . . . . . . . . . . . . . . . . . . . . . . . . . . . . . . . . . . . . . . . . . . . . . . . . . . . . . . . . . . . . . . . . . . . . .    
461	. . . . . . . C C C C C C C C C C C C C C C C C C C . C . C C C C C C . . . C . . C . . . . . . . . . . . . . . . . . . . . . . . . . . . . . . . . . . . . . . . . . . . . . . . . . . . . . . . . . . . . . . . . . . . . . . . . . . . . . . . . . . . . . . . . . . . . . . . . .    
462	. . . . . . . A A A A A A A A A A A A A A A A A A A . A . A A A A A A . . . A . . A . . . . . . . . . . . . . . . . . . . . . . . . . . . . . . . . . . . . . . . . . . . . . . . . . . . . . . . . . . . . . . . . . . . . . . . . . . . . . . . . . . . . . . . . . . . . . . . . .    
463	. . . . . . . G G G G G G G G G G G G G G G G G G G . G . G G G G G G . . . G . . G . . . . . . . . . . . . . . . . . . . . . . . . . . . . . . . . . . . . . . . . . . . . . . . . . . . . . . . . . . . . . . . . . . . . . . . . . . . . . . . . . . . . . . . . . . . . . . . . .    
464	. . . . . . . A A A A A A A A A A A A A A A A A A A . A . A A A A A A . . . A . . A . . . . . . . . . . . . . . . . . . . . . . . . . . . . . . . . . . . . . . . . . . . . . . . . . . . . . . . . . . . . . . . . . . . . . . . . . . . . . . . . . . . . . . . . . . . . . . . . .    
465	. . . . . . . G G G G G G G G G G G G G G G G G G G . G . G G G G G G . . . G . . G . . . . . . . . . . . . . . . . . . . . . . . . . . . . . . . . . . . . . . . . . . . . . . . . . . . . . . . . . . . . . . . . . . . . . . . . . . . . . . . . . . . . . . . . . . . . . . . . .    
466	. . . . . . . T T T T T T T T T T T T T T T T T T T . T . T T T T T T . . . T . . T . . . . . . . . . . . . . . . . . . . . . . . . . . . . . . . . . . . . . . . . . . . . . . . . . . . . . . . . . . . . . . . . . . . . . . . . . . . . . . . . . . . . . . . . . . . . . . . . .    
467	. . . . . . . G G G G G G G G G G G G G G G G G G G . G . G G G G G G . . . G . . G . G . . . . . . . . . . . . . . . . . . . . . . . . . . . . . . . . . . . . . . . . . . . . . . . . . . . . . . . . . . . . . . . . . . . . . . . . . . . . . . . . . . . . . . . . . . . . . . .    
468	. . . . . . . A A A A A A A A A A A A A G A A A A A . A . A A A A A A . . . A . . A . A . . . . . . . . . . . . . . . . . . . . . . . . . . . . . . . . . . . . . . . . . . . . . . . . . . . . . . . . . . . . . . . . . . . . . . . . . . . . . . . . . . . . . . . . . . . . . . .    
469	. . . . . . . C C C C C C C C C C C C C C C C C C C . C . C C C C C C . . . C . . C . C . . . . . . . . . . . . . . . . . . . . . . . . . . . . . . . . . . . . . . . . . . . . . . . . . . . . . . . . . . . . . . . . . . . . . . . . . . . . . . . . . . . . . . . . . . . . . . .    
470	. . . . . . . C C C C C C C C C C C C C C C C C C C . C . C C C C C C . . . C . . C . C . . . . . . . . . . . . . . . . . . . . . . . . . . . . . . . . . . . . . . . . . . . . . . . . . . . . . . . . . . . . . . . . . . . . . . . . . . . . . . . . . . . . . . . . . . . . . . .    
471	. . . . . . . T T T T T T T T T T T T T T T T T T T . T . T T T T T T . . . T . . T . T . . . . . . . . . . . . . . . . . . . . . . . . . . . . . . . . . . . . . . . . . . . . . . . . . . . . . . . . . . . . . . . . . . . . . . . . . . . . . . . . . . . . . . . . . . . . . . .    
472	. . . . . . . G G G G G G G G G G G G G G G G G G G . G . G G G G G G . . . G . . G . G . . . . . . . . . . . . . . . . . . . . . . . . . . . . . . . . . . . . . . . . . . . . . . . . . . . . . . . . . . . . . . . . . . . . . . . . . . . . . . . . . . . . . . . . . . . . . . .    
473	. . . . . . . G G G G G G G G G G G G G G G G G G G . G . G G G G G G . . . G . . G . G . . . . . . . . . . . . . . . . . . . . . . . . . . . . . . . . . . . . . . . . . . . . . . . . . . . . . . . . . . . . . . . . . . . . . . . . . . . . . . . . . . . . . . . . . . . . . . .    
474	. . . . . . . C C C C C C C C C C C C C C C C C C C . C . C C C C C C . . . C . . C . C . . . . . . . . . . . . . . . . . . . . . . . . . . . . . . . . . . . . . . . . . . . . . . . . . . . . . . . . . . . . . . . . . . . . . . . . . . . . . . . . . . . . . . . . . . . . . . .    
475	. . . . . . . T T T T T T T T T T T T T T T T T T T . T . T T T T T T . . . T . . T . T . . . . . . . . . . . . . . . . . . . . . . . . . . . . . . . . . . . . . . . . . . . . . . . . . . . . . . . . . . . . . . . . . . . . . . . . . . . . . . . . . . . . . . . . . . . . . . .    
476	. . . . . . . G G G G G G G G G G G G G G G G G G G . G . G G G G G G . . . G . . G . G . . . . . . . . . . . . . . . . . . . . . . . . . . . . . . . . . . . . . . . . . . . . . . . . . . . . . . . . . . . . . . . . . . . . . . . . . . . . . . . . . . . . . . . . . . . . . . .    
477	. . . . . . . A A A A A A A A A A A A A A A A A A A . A . A A A A A A . . . A . . A . A . . . . . . . . . . . . . . . . . . . . . . . . . . . . . . . . . . . . . . . . . . . . . . . . . . . . . . . . . . . . . . . . . . . . . . . . . . . . . . . . . . . . . . . . . . . . . . .    
478	. . . . . . . G G G G G G G G G G G G G G G G G G G . G . G G G G G G . . . G . . G . G . . . . . . . . . . . . . . . . . . . . . . . . . . . . . . . . . . . . . . . . . . . . . . . . . . . . . . . . . . . . . . . . . . . . . . . . . . . . . . . . . . . . . . . . . . . . . . .    
479	. . . . . . . G G G G G G G G G G G G G G G G G G G . G . G G G G G G . . . G . . G . G . . . . . . . . . . . . . . . . . . . . . . . . . . . . . . . . . . . . . . . . . . . . . . . . . . . . . . . . . . . . . . . . . . . . . . . . . . . . . . . . . . . . . . . . . . . . . . .    
480	. . . . . . . G G G G G G G G G G G G G G G G G G G . G . G G G G G G . . . G . . G . G . . . . . . . . . . . . . . . . . . . . . . . . . . . . . . . . . . . . . . . . . . . . . . . . . . . . . . . . . . . . . . . . . . . . . . . . . . . . . . . . . . . . . . . . . . . . . . .    
481	. . . . . . . . A A A A A A A A A A A A A A A A A A . A . A A A A A A . . . A . . A . A . . . . . . . . . . . . . . . . . . . . . . . . . . . . . . . . . . . . . . . . . . . . . . . . . . . . . . . . . . . . . . . . . . . . . . . . . . . . . . . . . . . . . . . . . . . . . . .    
482	. . . . . . . . T T T C C C T T T T T T T T C T T T . T . T T C T C T . . . T . . T . T . . . . . . . . . . . . . . . . . . . . . . . . . . . . . . . . . . . . . . . . . . . . . . . . . . . . . . . . . . . . . . . . . . . . . . . . . . . . . . . . . . . . . . . . . . . . . . .  6	 transition  
483	. . . . . . . . G G G G G G G G G G G G G G G G G G . G . G G G G G G . . . G . . G . G . . . . . . . . . . . . . . . . . . . . . . . . . . . . . . . . . . . . . . . . . . . . . . . . . . . . . . . . . . . . . . . . . . . . . . . . . . . . . . . . . . . . . . . . . . . . . . .    
484	. . . . . . . . G G G G G G G G G G G G G G G G G G . G . G G G G G G . . . G . . G . G . . . . . . . . . . . . . . . . . . . . . . . . . . . . . . . . . . . . . . . . . . . . . . . . . . . . . . . . . . . . . . . . . . . . . . . . . . . . . . . . . . . . . . . . . . . . . . .    
485	. . . . . . . . A A A A A A A A A A A A A A A A A A . A . A A A A A A . . . A . . A . A . . . . . . . . . . . . . . . . . . . . . . . . . . . . . . . . . . . . . . . . . . . . . . . . . . . . . . . . . . . . . . . . . . . . . . . . . . . . . . . . . . . . . . . . . . . . . . .    
486	. . . . . . . . C C C C C C C C C C C C C C C C C C . C . C C C C C C . . . C . . C . C . . . . . . . . . . . . . . . . . . . . . . . . . . . . . . . . . . . . . . . . . . . . . . . . . . . . . . . . . . . . . . . . . . . . . . . . . . . . . . . . . . . . . . . . . . . . . . .    
487	. . . . . . . . G G G C G G G G G G G G G G G G G G . G . G G G G G G . . . G . . G . G . . . . . . . . . . . . . . . . . . . . . . . . . . . . . . . . . . . . . . . . . . . . . . . . . . . . . . . . . . . . . . . . . . . . . . . . . . . . . . . . . . . . . . . . . . . . . . .    
488	. . . . . . . . T T T T T T T T T T T T T T T T T T . T . T T T T T T . . . T . . T . T . . . . . . . . . . . . . . . . . . . . . . . . . . . . . . . . . . . . . . . . . . . . . . . . . . . . . . . . . . . . . . . . . . . . . . . . . . . . . . . . . . . . . . . . . . . . . . .    
489	. . . . . . . . - - - T - - - - - - - - - - - - - - . - . - - - - - - . . . - . . - . - . . . . . . . . . . . . . . . . . . . . . . . . . . . . . . . . . . . . . . . . . . . . . . . . . . . . . . . . . . . . . . . . . . . . . . . . . . . . . . . . . . . . . . . . . . . . . . .    
490	. . . . . . . . G G G G G G G G G G G G G G G G G G . G . G G G G G G . . . G . . G . G . . . . . . . . . . . . . . . . . . . . . . . . . . . . . . . . . . . . . . . . . . . . . . . . . . . . . . . . . . . . . . . . . . . . . . . . . . . . . . . . . . . . . . . . . . . . . . .    
491	. . . . . . . . A A A A A A A A A A A A A A A A A A . A . A A A A A A . . . A . . A . A . . . . . . . . . . . . . . . . . . . . . . . . . . . . . . . . . . . . . . . . . . . . . . . . . . . . . . . . . . . . . . . . . . . . . . . . . . . . . . . . . . . . . . . . . . . . . . .    
492	. . . . . . . . G G G A G G G G G G G G G G G G G G . G . G G G G G G . . . G . . G . G . . . . . . . . . . . . . . . . . . . . . . . . . . . . . . . . . . . . . . . . . . . . . . . . . . . . . . . . . . . . . . . . . . . . . . . . . . . . . . . . . . . . . . . . . . . . . . .    
493	. . . . . . . . G G G G G G G G G G G G G G G G G G . G . G G G G G G . . . G . . G . G . . . . . . . . . . . . . . . . . . . . . . . . . . . . . . . . . . . . . . . . . . . . . . . . . . . . . . . . . . . . . . . . . . . . . . . . . . . . . . . . . . . . . . . . . . . . . . .    
494	. . . . . . . . T T T T T T T T T T T T T T T T T T . T . T T T T T T . . . T . . T . T . . . . . . . . . . . . . . . . . . . . . . . . . . . . . . . . . . . . . . . . . . . . . . . . . . . . . . . . . . . . . . . . . . . . . . . . . . . . . . . . . . . . . . . . . . . . . . .    
495	. . . . . . . . G G G G G G G G G G G G G G G G G G . G . G G G G G G . . . G . . G . G . . . . . . . . . . . . . . . . . . . . . . . . . . . . . . . . . . . . . . . . . . . . . . . . . . . . . . . . . . . . . . . . . . . . . . . . . . . . . . . . . . . . . . . . . . . . . . .    
496	. . . . . . . . A A A A A A A A A A A A A A A A A A . A . A A A A A A . . . A . . A . A . . . . . . . . . . . . . . . . . . . . . . . . . . . . . . . . . . . . . . . . . . . . . . . . . . . . . . . . . . . . . . . . . . . . . . . . . . . . . . . . . . . . . . . . . . . . . . .    
497	. . . . . . . . A A A A A A A A A A A A A A A A A A . A . A A A A A A . . . A . . A . A . . . . . . . . . . . . . . . . . . . . . . . . . . . . . . . . . . . . . . . . . . . . . . . . . . . . . . . . . . . . . . . . . . . . . . . . . . . . . . . . . . . . . . . . . . . . . . .    
498	. . . . . . . . . G G G G G G G G G G G G G G G G G . G . G G G G G G . . . G . . G . G . . . . . . . . . . . . . . . . . . . . . . . . . . . . . . . . . . . . . . . . . . . . . . . . . . . . . . . . . . . . . . . . . . . . . . . . . . . . . . . . . . . . . . . . . . . . . . .    
499	. . . . . . . . . T T T T T T T T T T T T T T T T T . T . T T T T T T . . . T . . T . T . . . . . . . . . . . . . . . . . . . . . . . . . . . . . . . . . . . . . . . . . . . . . . . . . . . . . . . . . . . . . . . . . . . . . . . . . . . . . . . . . . . . . . . . . . . . . . .    
500	. . . . . . . . . C C C C C C C C C C C C C C C C C . C . C C C C C C . . . C . . C . C . . . . . . . . . . . . . . . . . . . . . . . . . . . . . . . . . . . . . . . . . . . . . . . . . . . . . . . . . . . . . . . . . . . . . . . . . . . . . . . . . . . . . . . . . . . . . . .    
501	. . . . . . . . . T T T T T T T T T T T T T T T T T . T . T T T T T T . . . T . . T . T . . . . . . . . . . . . . . . . . . . . . . . . . . . . . . . . . . . . . . . . . . . . . . . . . . . . . . . . . . . . . . . . . . . . . . . . . . . . . . . . . . . . . . . . . . . . . . .    
502	. . . . . . . . . G G G G G G G G G G G G G G G G G . G . G G G G G G . . . G . . G . G . . . . . . . . . . . . . . . . . . . . . . . . . . . . . . . . . . . . . . . . . . . . . . . . . . . . . . . . . . . . . . . . . . . . . . . . . . . . . . . . . . . . . . . . . . . . . . .    
503	. . . . . . . . . A A A A A A A A A A A A A A G A A . A . A A A A A A . . . A . . A . A . . . . . . . . . . . . . . . . . . . . . . . . . . . . . . . . . . . . . . . . . . . . . . . . . . . . . . . . . . . . . . . . . . . . . . . . . . . . . . . . . . . . . . . . . . . . . . .    
504	. . . . . . . . . T T T T T T T T T T T T T T T T T . T . T T T T T T . . . T . . T . T . . . . . . . . . . . . . . . . . . . . . . . . . . . . . . . . . . . . . . . . . . . . . . . . . . . . . . . . . . . . . . . . . . . . . . . . . . . . . . . . . . . . . . . . . . . . . . .    
505	. . . . . . . . . G G G G G G G G G G G G G G G G G . G . G G G G G G . . . G . . G . G G . . . . . . . . . . . . . . . . . . . . . . . . . . . . . . . . . . . . . . . . . . . . . . . . . . . . . . . . . . . . . . . . . . . . . . . . . . . . . . . . . . . . . . . . . . . . . .    
506	. . . . . . . . . T T T T T T T T T T T T T T T T T . T . T T T T T T . . . T . . T . T T . . . . . . . . . . . . . . . . . . . . . . . . . . . . . . . . . . . . . . . . . . . . . . . . . . . . . . . . . . . . . . . . . . . . . . . . . . . . . . . . . . . . . . . . . . . . . .    
507	. . . . . . . . . G G G G G G G G G G G G G G G G G . G . G G G G G G . . . G . . G . G G . . . . . . . . . . . . . . . . . . . . . . . . . . . . . . . . . . . . . . . . . . . . . . . . . . . . . . . . . . . . . . . . . . . . . . . . . . . . . . . . . . . . . . . . . . . . . .    
508	. . . . . . . . . A A A A A A A A A A A A A A A A A . A . A A A A A A . . . A . . A . A A . . . . . . . . . . . . . . . . . . . . . . . . . . . . . . . . . . . . . . . . . . . . . . . . . . . . . . . . . . . . . . . . . . . . . . . . . . . . . . . . . . . . . . . . . . . . . .    
509	. . . . . . . . . C C C C C C C C C C C C C C C C C . C . C C C C C C . . . C . . C . C C . . . . . . . . . . . . . . . . . . . . . . . . . . . . . . . . . . . . . . . . . . . . . . . . . . . . . . . . . . . . . . . . . . . . . . . . . . . . . . . . . . . . . . . . . . . . . .    
510	. . . . . . . . . C C C C C C C C C C C C C C C C C . C . C C C C C C . . . C . . C . C C C . . . . . . . . . . . . . . . . . . . . . . . . . . . . . . . . . . . . . . . . . . . . . . . . . . . . . . . . . . . . . . . . . . . . . . . . . . . . . . . . . . . . . . . . . . . . .    
511	. . . . . . . . . T T T T T T T T T T T T T T T T T . T . T T T T T T . . . T . . T . T T T . . . . . . . . . . . . . . . . . . . . . . . . . . . . . . . . . . . . . . . . . . . . . . . . . . . . . . . . . . . . . . . . . . . . . . . . . . . . . . . . . . . . . . . . . . . . .    
512	. . . . . . . . . C C C C C C C C C C C C C C C C C . C . C C C C C C . . . C . . C . C C C . . . . . . . . . . . . . . . . . . . . . . . . . . . . . . . . . . . . . . . . . . . . . . . . . . . . . . . . . . . . . . . . . . . . . . . . . . . . . . . . . . . . . . . . . . . . .    
513	. . . . . . . . . C C C C C C C C C C C C C C C C C . C . C C C C C C . . . C . . C . C C C . . . . . . . . . . . . . . . . . . . . . . . . . . . . . . . . . . . . . . . . . . . . . . . . . . . . . . . . . . . . . . . . . . . . . . . . . . . . . . . . . . . . . . . . . . . . .    
514	. . . . . . . . . A A A A A A A A A A A A A A A A A . A . A A A A A A . . . A . . A . A A A . . . . . . . . . . . . . . . . . . . . . . . . . . . . . . . . . . . . . . . . . . . . . . . . . . . . . . . . . . . . . . . . . . . . . . . . . . . . . . . . . . . . . . . . . . . . .    
515	. . . . . . . . . C C C C C C C C C C C C C C C C C . C . C C C C C C . . . C . . C . C C C . . . . . . . . . . . . . . . . . . . . . . . . . . . . . . . . . . . . . . . . . . . . . . . . . . . . . . . . . . . . . . . . . . . . . . . . . . . . . . . . . . . . . . . . . . . . .    
516	. . . . . . . . . T T T T T T T T T T T T T T T T T . T . T T T T T T . . . T . . T . T T T . . . . . . . . . . . . . . . . . . . . . . . . . . . . . . . . . . . . . . . . . . . . . . . . . . . . . . . . . . . . . . . . . . . . . . . . . . . . . . . . . . . . . . . . . . . . .    
517	. . . . . . . . . G G G G G G G G G G G G G G G G G . G . G G G G G G . . . G . . G . G G G . . . . . . . . . . . . . . . . . . . . . . . . . . . . . . . . . . . . . . . . . . . . . . . . . . . . . . . . . . . . . . . . . . . . . . . . . . . . . . . . . . . . . . . . . . . . .    
518	. . . . . . . . . A A A A A A A A A A A A A A A A A . A . A A A A A A . . . A . . A . A A A . . . . . . . . . . . . . . . . . . . . . . . . . . . . . . . . . . . . . . . . . . . . . . . . . . . . . . . . . . . . . . . . . . . . . . . . . . . . . . . . . . . . . . . . . . . . .    
519	. . . . . . . . . G G G G G G G G G G G G G G G G G . G . G G G G G G . . . G . . G . G G G . . . . . . . . . . . . . . . . . . . . . . . . . . . . . . . . . . . . . . . . . . . . . . . . . . . . . . . . . . . . . . . . . . . . . . . . . . . . . . . . . . . . . . . . . . . . .    
520	. . . . . . . . . G G G G G G G G G G G G G G G G G . G . G G G G G G . . . G . . G . G G G . . . . . . . . . . . . . . . . . . . . . . . . . . . . . . . . . . . . . . . . . . . . . . . . . . . . . . . . . . . . . . . . . . . . . . . . . . . . . . . . . . . . . . . . . . . . .    
521	. . . . . . . . . A A A A A A A A A A A A A A A A A . A . A A A A A A . . . A . . A . A A A . . . . . . . . . . . . . . . . . . . . . . . . . . . . . . . . . . . . . . . . . . . . . . . . . . . . . . . . . . . . . . . . . . . . . . . . . . . . . . . . . . . . . . . . . . . . .    
522	. . . . . . . . . G G G G G G G G G G G G G G G G G . G . G G G G G G . . . G . . G . G G G . . . . . . . . . . . . . . . . . . . . . . . . . . . . . . . . . . . . . . . . . . . . . . . . . . . . . . . . . . . . . . . . . . . . . . . . . . . . . . . . . . . . . . . . . . . . .    
523	. . . . . . . . . C C C C C C C C C C C C C C C C C . C C C C C C C C . . . C . . C . C C C . . . . . . . . . . . . . . . . . . . . . . . . . . . . . . . . . . . . . . . . . . . . . . . . . . . . . . . . . . . . . . . . . . . . . . . . . . . . . . . . . . . . . . . . . . . . .    
524	. . . . . . . . . T T T T T T T T T T T T T T T T T . T T T T T T T T . . . T . . T . T T T . . . . . . . . . . . . . . . . . . . . . . . . . . . . . . . . . . . . . . . . . . . . . . . . . . . . . . . . . . . . . . . . . . . . . . . . . . . . . . . . . . . . . . . . . . . . .    
525	. . . . . . . . . . G G G G G G G G G G G G G G G G . G G G G G G G G . . . G . . G . G G G . . . . . . . . . . . . . . . . . . . . . . . . . . . . . . . . . . . . . . . . . . . . . . . . . . . . . . . . . . . . . . . . . . . . . . . . . . . . . . . . . . . . . . . . . . . . .    
526	. . . . . . . . . . G G G G G G G G G G G G G G G G . G G G G G G G G . . . G . . G . G G G . . . . . . . . . . . . . . . . . . . . . . . . . . . . . . . . . . . . . . . . . . . . . . . . . . . . . . . . . . . . . . . . . . . . . . . . . . . . . . . . . . . . . . . . . . . . .    
527	. . . . . . . . . . C C C C C C C C C C C C C C C C . C C C C C C C C . . . C . . C . C C C . . . . . . . . . . . . . . . . . . . . . . . . . . . . . . . . . . . . . . . . . . . . . . . . . . . . . . . . . . . . . . . . . . . . . . . . . . . . . . . . . . . . . . . . . . . . .    
528	. . . . . . . . . . T T T T T T T T T T T T T T T T . T T T T T T T T . . . T . . T . T T T . . . . . . . . . . . . . . . . . . . . . . . . . . . . . . . . . . . . . . . . . . . . . . . . . . . . . . . . . . . . . . . . . . . . . . . . . . . . . . . . . . . . . . . . . . . . .    
529	. . . . . . . . . . A A A A A A A A A A A A A A A A . A A A A A A A A . . . A . . A . A A A A . . . . . . . . . . . . . . . . . . . . . . . . . . . . . . . . . . . . . . . . . . . . . . . . . . . . . . . . . . . . . . . . . . . . . . . . . . . . . . . . . . . . . . . . . . . .    
530	. . . . . . . . . . A A A A A A A A A A A A A A A A . A A A A A A A A . . . A . . A . A A A A . . . . . . . . . . . . . . . . . . . . . . . . . . . . . . . . . . . . . . . . . . . . . . . . . . . . . . . . . . . . . . . . . . . . . . . . . . . . . . . . . . . . . . . . . . . .    
531	. . . . . . . . . . C C C C C C C C C C C C C C C C . C C C C C C C C . . . C . . C . C C C C . . . . . . . . . . . . . . . . . . . . . . . . . . . . . . . . . . . . . . . . . . . . . . . . . . . . . . . . . . . . . . . . . . . . . . . . . . . . . . . . . . . . . . . . . . . .    
532	. . . . . . . . . . G G G G G G G G G G G G G G G G . G G G G G G G G . . . G . . G . G G G G . . . . . . . . . . . . . . . . . . . . . . . . . . . . . . . . . . . . . . . . . . . . . . . . . . . . . . . . . . . . . . . . . . . . . . . . . . . . . . . . . . . . . . . . . . . .    
533	. . . . . . . . . . G G G G G G G G G G G G G G G G . G G G G G G G G . . . G . . G . G G G G . . . . . . . . . . . . . . . . . . . . . . . . . . . . . . . . . . . . . . . . . . . . . . . . . . . . . . . . . . . . . . . . . . . . . . . . . . . . . . . . . . . . . . . . . . . .    
534	. . . . . . . . . . G N G G G G G G G G G G G G G G . G G G G G G G G . . . G . . G . G G G G . . . . . . . . . . . . . . . . . . . . . . . . . . . . . . . . . . . . . . . . . . . . . . . . . . . . . . . . . . . . . . . . . . . . . . . . . . . . . . . . . . . . . . . . . . . .    
535	. . . . . . . . . . G G G G G G G G G G G G G G G G . G G G G G G G G . . . G . . G . G G G G . . . . . . . . . . . . . . . . . . . . . . . . . . . . . . . . . . . . . . . . . . . . . . . . . . . . . . . . . . . . . . . . . . . . . . . . . . . . . . . . . . . . . . . . . . . .    
536	. . . . . . . . . . A A A A A A A A A A A A A A A A . A A A A A A A A . . . A . . A . A A A A . . . . . . . . . . . . . . . . . . . . . . . . . . . . . . . . . . . . . . . . . . . . . . . . . . . . . . . . . . . . . . . . . . . . . . . . . . . . . . . . . . . . . . . . . . . .    
537	. . . . . . . . . . C C C C C C C C C C C C C C C C . C C C C C C C C . . . C . . C . C C C C . . . . . . . . . . . . . . . . . . . . . . . . . . . . . . . . . . . . . . . . . . . . . . . . . . . . . . . . . . . . . . . . . . . . . . . . . . . . . . . . . . . . . . . . . . . .    
538	. . . . . . . . . . T T T T T T T T T T T T T T T T . T T T T T T T T . . . T . . T . T T T T . . . . . . . . . . . . . . . . . . . . . . . . . . . . . . . . . . . . . . . . . . . . . . . . . . . . . . . . . . . . . . . . . . . . . . . . . . . . . . . . . . . . . . . . . . . .    
539	. . . . . . . . . . G G G G G G G G G G G G G G G G . G G G G G G G G . . . G . . G . G G G G . . . . . . . . . . . . . . . . . . . . . . . . . . . . . . . . . . . . . . . . . . . . . . . . . . . . . . . . . . . . . . . . . . . . . . . . . . . . . . . . . . . . . . . . . . . .    
540	. . . . . . . . . . G G G G G G G G G G G G G G G G . G G G G G G G G . . . G . . G . G G G G . . . . . . . . . . . . . . . . . . . . . . . . . . . . . . . . . . . . . . . . . . . . . . . . . . . . . . . . . . . . . . . . . . . . . . . . . . . . . . . . . . . . . . . . . . . .    
541	. . . . . . . . . . T T T T T T T T T T T T T T T T . T T T T T T T T . . . T . . T . T T T T . . . . . . . . . . . . . . . . . . . . . . . . . . . . . . . . . . . . . . . . . . . . . . . . . . . . . . . . . . . . . . . . . . . . . . . . . . . . . . . . . . . . . . . . . . . .    
542	. . . . . . . . . . A A A A A A A A A A A A A A A A . A A A A A A A A . . . A . . A . A A A A . . . . . . . . . . . . . . . . . . . . . . . . . . . . . . . . . . . . . . . . . . . . . . . . . . . . . . . . . . . . . . . . . . . . . . . . . . . . . . . . . . . . . . . . . . . .    
543	. . . . . . . . . . C C C C C C C C C C C C C C C C . C C C C C C C C . . . C . . C . C C C C . . . . . . . . . . . . . . . . . . . . . . . . . . . . . . . . . . . . . . . . . . . . . . . . . . . . . . . . . . . . . . . . . . . . . . . . . . . . . . . . . . . . . . . . . . . .    
544	. . . . . . . . . . T T T T T T T T T T T T T T T T . T T T T T T T T . . . T . . T . T T T T . . . . . . . . . . . . . . . . . . . . . . . . . . . . . . . . . . . . . . . . . . . . . . . . . . . . . . . . . . . . . . . . . . . . . . . . . . . . . . . . . . . . . . . . . . . .    
545	. . . . . . . . . . A A A A A A A A A A A A A A A A . A A A A A A A A . . . A . . A . A A A A . . . . . . . . . . . . . . . . . . . . . . . . . . . . . . . . . . . . . . . . . . . . . . . . . . . . . . . . . . . . . . . . . . . . . . . . . . . . . . . . . . . . . . . . . . . .    
546	. . . . . . . . . . C C C C C C C C C C C C C C C C . C C C C C C C C . . . C . . C . C C C C . . . . . . . . . . . . . . . . . . . . . . . . . . . . . . . . . . . . . . . . . . . . . . . . . . . . . . . . . . . . . . . . . . . . . . . . . . . . . . . . . . . . . . . . . . . .    
547	. . . . . . . . . . C C C C C C C C C C C C C C C C . C C C C C C C C . . . C . . C . C C C C . . . . . . . . . . . . . . . . . . . . . . . . . . . . . . . . . . . . . . . . . . . . . . . . . . . . . . . . . . . . . . . . . . . . . . . . . . . . . . . . . . . . . . . . . . . .    
548	. . . . . . . . . . A A A A A A A A A A A A A A A A . A A A A A A A A . . . A . . A . A A A A . . . . . . . . . . . . . . . . . . . . . . . . . . . . . . . . . . . . . . . . . . . . . . . . . . . . . . . . . . . . . . . . . . . . . . . . . . . . . . . . . . . . . . . . . . . .    
549	. . . . . . . . . . G G G G G G G G G G G G G G G G . G G G G G G G G . . . G . . G . G G G G . . . . . . . . . . . . . . . . . . . . . . . . . . . . . . . . . . . . . . . . . . . . . . . . . . . . . . . . . . . . . . . . . . . . . . . . . . . . . . . . . . . . . . . . . . . .    
550	. . . . . . . . . . A A A A A A A A A A A A A A A A . A A A A A A A A . . . A . . A . A A A A . . . . . . . . . . . . . . . . . . . . . . . . . . . . . . . . . . . . . . . . . . . . . . . . . . . . . . . . . . . . . . . . . . . . . . . . . . . . . . . . . . . . . . . . . . . .    
551	. . . . . . . . . . T T T T T T T T T T T T T T T T . T T T T T T T T . . . T . . T . T T T T . . . . . . . . . . . . . . . . . . . . . . . . . . . . . . . . . . . . . . . . . . . . . . . . . . . . . . . . . . . . . . . . . . . . . . . . . . . . . . . . . . . . . . . . . . . .    
552	. . . . . . . . . . C C C C C C C C C C C C C C C C . C C C C C C C C . . . C . . C . C C C C . . . . . . . . . . . . . . . . . . . . . . . . . . . . . . . . . . . . . . . . . . . . . . . . . . . . . . . . . . . . . . . . . . . . . . . . . . . . . . . . . . . . . . . . . . . .    
553	. . . . . . . . . . C C C C C C C C C C C C C C C C . C C C C C C C C C . . C . . C . C C C C . . . . . . . . . . . . . . . . . . . . . . . . . . . . . . . . . . . . . . . . . . . . . . . . . . . . . . . . . . . . . . . . . . . . . . . . . . . . . . . . . . . . . . . . . . . .    
554	. . . . . . . . . . A A A A A A A A A A A A A A A A . A A A A A A A A A . . A . . A . A A A A . . . . . . . . . . . . . . . . . . . . . . . . . . . . . . . . . . . . . . . . . . . . . . . . . . . . . . . . . . . . . . . . . . . . . . . . . . . . . . . . . . . . . . . . . . . .    
555	. . . . . . . . . . C C C C C C C C C C C C C C C C . C C C C C C C C C . . C . . C . C C C C . . . . . . . . . . . . . . . . . . . . . . . . . . . . . . . . . . . . . . . . . . . . . . . . . . . . . . . . . . . . . . . . . . . . . . . . . . . . . . . . . . . . . . . . . . . .    
556	. . . . . . . . . . T T T T T T T T T T T T T T T T . T T T T T T T T T . . T . . T . T T T T . . . . . . . . . . . . . . . . . . . . . . . . . . . . . . . . . . . . . . . . . . . . . . . . . . . . . . . . . . . . . . . . . . . . . . . . . . . . . . . . . . . . . . . . . . . .    
557	. . . . . . . . . . C C C C C C C C C C C C C C C C . C C C C C C C C C . . C . . C . C C C C . . . . . . . . . . . . . . . . . . . . . . . . . . . . . . . . . . . . . . . . . . . . . . . . . . . . . . . . . . . . . . . . . . . . . . . . . . . . . . . . . . . . . . . . . . . .    
558	. . . . . . . . . . C C C C C C C C C C C C C C C C . C C C C C C C C C . . C . . C . C C C C . . . . . . . . . . . . . . . . . . . . . . . . . . . . . . . . . . . . . . . . . . . . . . . . . . . . . . . . . . . . . . . . . . . . . . . . . . . . . . . . . . . . . . . . . . . .    
559	. . . . . . . . . . C C C C C C C C C C C C C C C C . C C C C C C C C C . . C . . C . C C C C . . . . . . . . . . . . . . . . . . . . . . . . . . . . . . . . . . . . . . . . . . . . . . . . . . . . . . . . . . . . . . . . . . . . . . . . . . . . . . . . . . . . . . . . . . . .    
560	. . . . . . . . . . A A A A A A A A A A A A A A A A . A A A A A A A A A . . A . . A . A A A A . . . . . . . . . . . . . . . . . . . . . . . . . . . . . . . . . . . . . . . . . . . . . . . . . . . . . . . . . . . . . . . . . . . . . . . . . . . . . . . . . . . . . . . . . . . .    
561	. . . . . . . . . . C C C C C C C C C C C C C C C C . C C C C C C C C C . . C . . C . C C C C . . . . . . . . . . . . . . . . . . . . . . . . . . . . . . . . . . . . . . . . . . . . . . . . . . . . . . . . . . . . . . . . . . . . . . . . . . . . . . . . . . . . . . . . . . . .    
562	. . . . . . . . . . C C C C C C C C C C C C C C C C . C C C C C C C C C . . C . . C . C C C C . . . . . . . . . . . . . . . . . . . . . . . . . . . . . . . . . . . . . . . . . . . . . . . . . . . . . . . . . . . . . . . . . . . . . . . . . . . . . . . . . . . . . . . . . . . .    
563	. . . . . . . . . . T T T T T T T T T T T T T T T T . T T T T T T T T T . . T . . T . T T T T . . . . . . . . . . . . . . . . . . . . . . . . . . . . . . . . . . . . . . . . . . . . . . . . . . . . . . . . . . . . . . . . . . . . . . . . . . . . . . . . . . . . . . . . . . . .    
564	. . . . . . . . . . G G G G G G G G G G G G G G G G . G G G G G G G G G . . G . . G . G G G G . . . . . . G . . . . . . . . . . . . . . . . . . . . . . . . . . . . . . . . . . . . . . . . . . . . . . . . . . . . . . . . . . . . . . . . . . . . . . . . . . . . . . . . . . . . .    
565	. . . . . . . . . . G G G G G G G G G G G G G G G G . G G G G G G G G G . . G . . G . G G G G . . . . . . G . . . . . . . . . . . . . . . . . . . . . . . . . . . . . . . . . . . . . . . . . . . . . . . . . . . . . . . . . . . . . . . . . . . . . . . . . . . . . . . . . . . . .    
566	. . . . . . . . . . A A A A A A A A A A A A A A A A . A A A A A A A A A . . A . . A . A A A A . . . . . . A . . . . . . . . . . . . . . . . . . . . . . . . . . . . . . . . . . . . . . . . . . . . . . . . . . . . . . . . . . . . . . . . . . . . . . . . . . . . . . . . . . . . .    
567	. . . . . . . . . . G G G G G G G G G G G G G G G G . G G G G G G G G G . . G . . G . G G G G . . . . . . G . . . . . . . . . . . . . . . . . . . . . . . . . . . . . . . . . . . . . . . . . . . . . . . . . . . . . . . . . . . . . . . . . . . . . . . . . . . . . . . . . . . . .    
568	. . . . . . . . . . T T T T T T T T T T T T T T T T . T T T T T T T T T . . T . . T . T T T T . . . . . . T . . . . . . . . . . . . . . . . . . . . . . . . . . . . . . . . . . . . . . . . . . . . . . . . . . . . . . . . . . . . . . . . . . . . . . . . . . . . . . . . . . . . .    
569	. . . . . . . . . . A A A A A A A A A A A A A A A A . A A A A A A A A A . . A . . A . A A A A . . . . . . A . . . . . . . . . . . . . . . . . . . . . . . . . . . . . . . . . . . . . . . . . . . . . . . . . . . . . . . . . . . . . . . . . . . . . . . . . . . . . . . . . . . . .    
570	. . . . . . . . . . C C C C C C C C C C C C C C C C . C C C C C C C C C . . C . . C . C C C C . . . . . . C . . . . . . . . . . . . . . . . . . . . . . . . . . . . . . . . . . . . . . . . . . . . . . . . . . . . . . . . . . . . . . . . . . . . . . . . . . . . . . . . . . . . .    
571	. . . . . . . . . . A A A A A A A A A A A A A A A A . A A A A A A A A A . . A . . A . A A A A . . . . . . A . . . . . . . . . . . . . . . . . . . . . . . . . . . . . . . . . . . . . . . . . . . . . . . . . . . . . . . . . . . . . . . . . . . . . . . . . . . . . . . . . . . . .    
572	. . . . . . . . . . C C C C C C C C C C C C C C C C . C C C C C C C C C . . C . . C . C C C C . . . . . . C C . . . . . . . . . . . . . . . . . . . . . . . . . . . . . . . . . . . . . . . . . . . . . . . . . . . . . . . . . . . . . . . . . . . . . . . . . . . . . . . . . . . .    
573	. . . . . . . . . . A A A A A A A A A A A A A A A A . A A A A A A A A A . . A . . A . A A A A . . . . . . A A . . . . . . . . . . . . . . . . . . . . . . . . . . . . . . . . . . . . . . . . . . . . . . . . . . . . . . . . . . . . . . . . . . . . . . . . . . . . . . . . . . . .    
574	. . . . . . . . . . C C C C C C C C C C C C C C C C . C C C C C C C C C . . C . . C . C C C C . . . . . . C C . . . . . . . . . . . . . . . . . . . . . . . . . . . . . . . . . . . . . . . . . . . . . . . . . . . . . . . . . . . . . . . . . . . . . . . . . . . . . . . . . . . .    
575	. . . . . . . . . . C C C C C C C C C C C C C C C C . C C C C C C C C C . . C . . C . C C C C . . . . . . C C . C . . . . . . . . . . . . . . . . . . . . . . . . . . . . . . . . . . . . . . . . . . . . . . . . . . . . . . . . . . . . . . . . . . . . . . . . . . . . . . . . . .    
576	. . . . . . . . . . C C C C C C C C C C C C C C C C . C C C C C C C C C . . C . . C . C C C C . . . . . . C C . C . . . . . . . . . . . . . . . . . . . . . . . . . . . . . . . . . . . . . . . . . . . . . . . . . . . . . . . . . . . . . . . . . . . . . . . . . . . . . . . . . .    
577	. . . . . . . . . . A . C A A A A A A A A A A A A A . A A A A A A A A A . . A . . A . A A A A . . . . . . A A . A . . . . . . . . . . . . . . . . . . . . . . . . . . . . . . . . . . . . . . . . . . . . . . . . . . . . . . . . . . . . . . . . . . . . . . . . . . . . . . . . . .    
578	. . . . . . . . . . G . . G G G G G G G G G G G G G . G G G G G G G G G . . G . . G . G G G G . . . . . . G G . G . . . . . . . . . . . . . . . . . . . . . . . . . . . . . . . . . . . . . . . . . . . . . . . . . . . . . . . . . . . . . . . . . . . . . . . . . . . . . . . . . .    
579	. . . . . . . . . . G . . G G G G G G G G G G G G G . G G G G G G G G G . . G . . G . G G G G . . . . . . G G . G . . . . . . . . . . . . . . . . . . . . . . . . . . . . . . . . . . . . . . . . . . . . . . . . . . . . . . . . . . . . . . . . . . . . . . . . . . . . . . . . . .    
580	. . . . . . . . . . T . . T T T T T T T T T T T T T . T T T T T T T T T . . T . . T . T T T T . . . . . . T T . T . . . . . . . . . . . . . . . . . . . . . . . . . . . . . . . . . . . . . . . . . . . . . . . . . . . . . . . . . . . . . . . . . . . . . . . . . . . . . . . . . .    
581	. . . . . . . . . . C . . C C C C C C C C C C C C C . C C C C C C C C C . . C . . C . C C C C . . . . . . C C . C . . . . . . . . . . . . . . . . . . . . . . . . . . . . . . . . . . . . . . . . . . . . . . . . . . . . . . . . . . . . . . . . . . . . . . . . . . . . . . . . . .    
582	. . . . . . . . . . T . . T T T T T T T T T T T T T . T T T T T T T T T . . T . . T . T T T T . . . . . . T T . T . . . . . . . . . . . . . . . . . . . . . . . . . . . . . . . . . . . . . . . . . . . . . . . . . . . . . . . . . . . . . . . . . . . . . . . . . . . . . . . . . .    
583	. . . . . . . . . . . . . G G G G G G G G G G G G G . G G G G G G G G G . . G . . G . G G G G . . . . . . G G . G . . . . . . . . . . . . . . . . . . . . . . . . . . . . . . . . . . . . . . . . . . . . . . . . . . . . . . . . . . . . . . . . . . . . . . . . . . . . . . . . . .    
584	. . . . . . . . . . . . . G G G G G G G G G G G G G . G G G G G G G G G . . G . . G . G G G G . . . . . . G G . G . . . . . . . . . . . . . . . . . . . . . . . . . . . . . . . . . . . . . . . . . . . . . . . . . . . . . . . . . . . . . . . . . . . . . . . . . . . . . . . . . .    
585	. . . . . . . . . . . . . A A A A A A A A A A A A A . A A A A A A A A A . . A . . A . A A A A . . . . . . A A . A . . . . . . . . . . . . . . . . . . . . . . . . . . . . . . . . . . . . . . . . . . . . . . . . . . . . . . . . . . . . . . . . . . . . . . . . . . . . . . . . . .    
586	. . . . . . . . . . . . . G G G G G G G G G G G G G . G G G G G G G G G . . G . . G . G G G G . . . . . . G G . G . . . . . . . . . . . . . . . . . . . . . . . . . . . . . . . . . . . . . . . . . . . . . . . . . . . . . . . . . . . . . . . . . . . . . . . . . . . . . . . . . .    
587	. . . . . . . . . . . . . A A A A A A A A A A A A A . A A A A A A A A A . . A . . A . A A A A . . . . . . A A . A . . . . . . . . . . . . . . . . . . . . . . . . . . . . . . . . . . . . . . . . . . . . . . . . . . . . . . . . . . . . . . . . . . . . . . . . . . . . . . . . . .    
588	. . . . . . . . . . . . . G G G G G G G G G G G G G . G G G G G G G G G . . G . . G . G G G G . . . . . . G G . G . . . . . . . . . . . . . . . . . . . . . . . . . . . . . . . . . . . . . . . . . . . . . . . . . . . . . . . . . . . . . . . . . . . . . . . . . . . . . . . . . .    
589	. . . . . . . . . . . . . A A A A A A A A A A A A A . A A A A A A A A A . . A . . A . A A A A . . . . . . A A . A . . . . . . . . . . . . . . . . . . . . . . . . . . . . . . . . . . . . . . . . . . . . . . . . . . . . . . . . . . . . . . . . . . . . . . . . . . . . . . . . . .    
590	. . . . . . . . . . . . . A A A A A A A A A A A A A . A A A A A A A A A . . A . . A . A A A A . . . . . . A A . A . . . . . . . . . . . . . . . . . . . . . . . . . . . . . . . . . . . . . . . . . . . . . . . . . . . . . . . . . . . . . . . . . . . . . . . . . . . . . . . . . .    
591	. . . . . . . . . . . . . G G G G G G G G G G G G G . G G G G G G G G G . . G . . G . G G G G . . . . . . G G . G . . . . . . . . . . . . . . . . . . . . . . . . . . . . . . . . . . . . . . . . . . . . . . . . . . . . . . . . . . . . . . . . . . . . . . . . . . . . . . . . . .    
592	. . . . . . . . . . . . . A A A A A A A A A A A A A . A A A A A A A A A . . A . . A . A A A A . . . . . . A A . A . . . . . . . . . . . . . . . . . . . . . . . . . . . . . . . . . . . . . . . . . . . . . . . . . . . . . . . . . . . . . . . . . . . . . . . . . . . . . . . . . .    
593	. . . . . . . . . . . . . T T T T T T T T T T T T T . T T T T T T T T T . . T . . T . T T T T . . . . . . T T . T . . . . . . . . . . . . . . . . . . . . . . . . . . . . . . . . . . . . . . . . . . . . . . . . . . . . . . . . . . . . . . . . . . . . . . . . . . . . . . . . . .    
594	. . . . . . . . . . . . . C C C C C C C C C C C C C . C C C C C C C C C . . C . . C . C C C C . . . . . . C C . C . . . . . . . . . . . . . . . . . . . . . . . . . . . . . . . . . . . . . . . . . . . . . . . . . . . . . . . . . . . . . . . . . . . . . . . . . . . . . . . . . .    
595	. . . . . . . . . . . . . T T T T T T T T T T T T T . T T T T T T T T T . . T . . T . T T T T . . . . . . T T . T . . . . . . . . . . . . . . . . . . . . . . . . . . . . . . . . . . . . . . . . . . . . . . . . . . . . . . . . . . . . . . . . . . . . . . . . . . . . . . . . . .    
596	. . . . . . . . . . . . . C C C C C C C C C C C C C . C C C C C C C C C . . C . . C . C C C C . . . . . . C C . C . . . . . . . . . . . . . . . . . . . . . . . . . . . . . . . . . . . . . . . . . . . . . . . . . . . . . . . . . . . . . . . . . . . . . . . . . . . . . . . . . .    
597	. . . . . . . . . . . . . C C C C C C C C C C C C C . C C C C C C C C C . . C . . C . C C C C . . . . . . C C . C . . . . . . . . . . . . . . . . . . . . . . . . . . . . . . . . . . . . . . . . . . . . . . . . . . . . . . . . . . . . . . . . . . . . . . . . . . . . . . . . . .    
598	. . . . . . . . . . . . . T T T T T T T T T T T T T . T T T T T T T T T . . T . . T . T T T T . . . . . . T T . T . . . . . . . . . . . . . . . . . . . . . . . . . . . . . . . . . . . . . . . . . . . . . . . . . . . . . . . . . . . . . . . . . . . . . . . . . . . . . . . . . .    
599	. . . . . . . . . . . . . G G G G G G G G G G G G G . G G G G G G G G G . . G . . G . G G G G . . . . . . G G . G . . . . . . . . . . . . . . . . . . . . . . . . . . . . . . . . . . . . . . . . . . . . . . . . . . . . . . . . . . . . . . . . . . . . . . . . . . . . . . . . . .    
600	. . . . . . . . . . . . . C C C C C C C C C C C C C . C C C C C C C C C C . C . . C . C C C C . . . . . . C C . C . . . . . . . . . . . . . . . . . . . . . . . . . . . . . . . . . . . . . . . . . . . . . . . . . . . . . . . . . . . . . . . . . . . . . . . . . . . . . . . . . .    
601	. . . . . . . . . . . . . A A A A A A A A A A A A A . A A A A A A A A A A . A . . A . A A A A . . . . . . A A . A . . . . . . . . . . . . . . . . . . . . . . . . . . . . . . . . . . . . . . . . . . . . . . . . . . . . . . . . . . . . . . . . . . . . . . . . . . . . . . . . . .    
602	. . . . . . . . . . . . . T T T T T T T T T T T T T . T T C T T T T T T T . T . . T . T T T T . . . . . . T T . T . . . . . . . . . . . . . . . . . . . . . . . . . . . . . . . . . . . . . . . . . . . . . . . . . . . . . . . . . . . . . . . . . . . . . . . . . . . . . . . . . .    
603	. . . . . . . . . . . . . G G G G G G G G G G G G G . G G G G G G G G G G . G . . G . G G G G . . . . . . G G . G . . . . . . . . . . . . . . . . . . . . . . . . . . . . . . . . . . . . . . . . . . . . . . . . . . . . . . . . . . . . . . . . . . . . . . . . . . . . . . . . . .    
604	. . . . . . . . . . . . . G G G G G G G G G G G G G . G G G G G G G G G G . G . . G . G G G G . . . . . . G G . G . . . . . . . . . . . . . . . . . . . . . . . . . . . . . . . . . . . . . . . . . . . . . . . . . . . . . . . . . . . . . . . . . . . . . . . . . . . . . . . . . .    
605	. . . . . . . . . . . . . T T T T T T T T T T T T T . T T T T T T T T T T . T . . T . T T T T . . . . . . T T . T . . . . . . . . . . . . . . . . . . . . . . . . . . . . . . . . . . . . . . . . . . . . . . . . . . . . . . . . . . . . . . . . . . . . . . . . . . . . . . . . . .    
606	. . . . . . . . . . . . . G G G G G G G G G G G G G . G G G G G G G G G G . G . . G . G G G G . . . . . . G G . G . . . . . . . . . . . . . . . . . . . . . . . . . . . . . . . . . . . . . . . . . . . . . . . . . . . . . . . . . . . . . . . . . . . . . . . . . . . . . . . . . .    
607	. . . . . . . . . . . . . G G G G G G G G G G G G G . G G G G G G G G G G . G . . G . G G G G . . . . . . G G . G . . . . . . . . . . . . . . . . . . . . . . . . . . . . . . . . . . . . . . . . . . . . . . . . . . . . . . . . . . . . . . . . . . . . . . . . . . . . . . . . . .    
608	. . . . . . . . . . . . . A A A A A A A A A A A A A . A A A A A A A A A A . A . . A . A A A A . . . . . . A A . A . . . . . . . . . . . . . . . . . . . . . . . . . . . . . . . . . . . . . . . . . . . . . . . . . . . . . . . . . . . . . . . . . . . . . . . . . . . . . . . . . .    
609	. . . . . . . . . . . . . G G G G G G G G G G G G G . G G G G G G G G G G . G . . G . G G G G G . . . . . G G G G . . . . . . . . . . . . . . . . . . . . . . . . . . . . . . . . . . . . . . . . . . . . . . . . . . . . . . . . . . . . . . . . . . . . . . . . . . . . . . . . . .    
610	. . . . . . . . . . . . . C C C C C C C C C C C C C . C C C C C C C C C C . C . . C . C C C C C . . . . . C C C C . . . . . . . . . . . . . . . . . . . . . . . . . . . . . . . . . . . . . . . . . . . . . . . . . . . . . . . . . . . . . . . . . . . . . . . . . . . . . . . . . .    
611	. . . . . . . . . . . . . A A A A A A A A A A A A A . A A A A A A A A A A . A . . A . A A A A A . . . . . A A A A . . . . . . . . . . . . . . . . . . . . . . . . . . . . . . . . . . . . . . . . . . . . . . . . . . . . . . . . . . . . . . . . . . . . . . . . . . . . . . . . . .    
612	. . . . . . . . . . . . . C C C C C C C C C C C C C . C C C C C C C C C C . C . . C . C C C C C . . . . . C C C C . . . . . . . . . . . . . . . . . . . . . . . . . . . . . . . . . . . . . . . . . . . . . . . . . . . . . . . . . . . . . . . . . . . . . . . . . . . . . . . . . .    
613	. . . . . . . . . . . . . A A A A A A A A A A A A A . A A A A A A A A A A . A . . A . A A A A A . . A . . A A A A . . . . . . . . . . . . . . . . . . . A . . . . . . . . . . . . . . . . . . . . . . . . . . . . . . . . . . . . . . . . . . . . . . . . . . . . . . . . . . . . . .    
614	. . . . . . . . . . . . . T T T T T T T T T T T T T . T T T T T T T T T T . T . . T . T T T T T . . T . . T T T T . . . . . . . . . . . . . . . . . . . T . . . . . . . . . . . . . . . . . . . . . . . . . . . . . . . . . . . . . . . . . . . . . . . . . . . . . . . . . . . . . .    
615	. . . . . . . . . . . . . C C C C C C C C C C C C C . C C C C C C C C C C . C . . C . C C C C C . . C . . C C C C . . . . . . . . . . . . . . . . . . . C . . . . . . . . . . . . . . . . . . . . . . . . . . . . . . . . . . . . . . . . . . . . . . . . . . . . . . . . . . . . . .    
616	. . . . . . . . . . . . . A A A A A A A A A A A A A . A A A A A A A A A A . A . . A . A A A A A . . A . . A A A A . . . . . . . . . . . . . . . . . . . A . . . . . . . . . . . . . . . . . . . . . . . . . . . . . . . . . . . . . . . . . . . . . . . . . . . . . . . . . . . . . .    
617	. . . . . . . . . . . . . . G G G G G G G G G G G G . G G G G G G G G G G . G . . G . G G G G G . . G . . G G G G . . . . . . . . . . . . . . . . . . . G . . . . . . . . . . . . . . . . . . . . . . . . . . . . . . . . . . . . . . . . . . . . . . . . . . . . . . . . . . . . . .    
618	. . . . . . . . . . . . . . C C C C C C C C C C C C . C C C C C C C C C C . C . . C . C C C C C . . C . . C C C C . . . . . . . . . . . . . . . . . . . C . . . . . . . . . . . . . . . . . . . . . . . . . . . . . . . . . . . . . . . . . . . . . . . . . . . . . . . . . . . . . .    
619	. . . . . . . . . . . . . . C C C C C C C C C C C C . C C C C C C C C C C . C . . C . C C C C C . . C . . C C C C . . . . . . . . . . . . . . . . . . . C . . . . . . . . . . . . . . . . . . . . . . . . . . . . . . . . . . . . . . . . . . . . . . . . . . . . . . . . . . . . . .    
620	. . . . . . . . . . . . . . T T T T T T T T T T T T . T T T T T T T T T T . T . . T . T T T T T . . T . . T T T T . . . . . . . . . . . . . . . . . . . T . . . . . . . . . . . . . . . . . . . . . . . . . . . . . . . . . . . . . . . . . . . . . . . . . . . . . . . . . . . . . .    
621	. . . . . . . . . . . . . . C C C C C C C C C C C C . C C C C C C C T C C . C . . C . C C C C C . . C . C C C C C . . . . . . . . . . . . . . . . . . . C . . . . . . . . . . . . . . . . . . . . . . . . . . . . . . . . . . . . . . . . . . . . . . . . . . . . . . . . . . . . . .    
622	. . . . . . . . . . . . . . A A A A A A A A A A A A . A A A A A A A A A A . A . . A . A A A A A . . A . A A A A A . . . . . . . . . . . . . . . . . . . A . . . . . . . . . . . . . . . . . . . . . . . . . . . . . . . . . . . . . . . . . . . . . . . . . . . . . . . . . . . . . .    
623	. . . . . . . . . . . . . . C C C C C C C C C C C C . C C C C C C C C C C . C . . C . C C C C C . . C . C C C C C . . . . . . . . . . . . . . . . . . . C . . . . . . . . . . . . . . . . . . . . . . . . . . . . . . . . . . . . . . . . . . . . . . . . . . . . . . . . . . . . . .    
624	. . . . . . . . . . . . . . T T T T T T T T T T T T . T T T T T T T T T T . T . . T . T T T T T . . T . T T T T T . . . . . . . . . . . . . . . . . . . T . . . . . . . . . . . . . . . . . . . . . . . . . . . . . . . . . . . . . . . . . . . . . . . . . . . . . . . . . . . . . .    
625	. . . . . . . . . . . . . . G G G G G G G G G G G G . G G G G G G G G G G . G . . G . G G G G G . . G . G G G G G . . . . . . . . . . . . . . . . . . . G . . . . . . . . . . . . . . . . . . . . . . . . . . . . . . . . . . . . . . . . . . . . . . . . . . . . . . . . . . . . . .    
626	. . . . . . . . . . . . . . A A A A A A A A A A A A . A A A A A A A A A A . A . . A . A A A A A . . A . A A A A A . . . . . . . . . . . . . . . . . . . A . . . . . . . . . . . . . . . . . . . . . . . . . . . . . . . . . . . . . . . . . . . . . . . . . . . . . . . . . . . . . .    
627	. . . . . . . . . . . . . . G G G G G G G G G G G G . G G G G G G G G G G . G . . G . G G G G G . . G . G G G G G . . . . . . . . . . . . . . . . . . . G . . . . . . . . . . . . . . . . . . . . . . . . . . . G . . . . . . . . . . . . . . . . . . . . . . . . . . . . . . . . . .    
628	. . . . . . . . . . . . . . C C C C C C C C C C C C . C C C C C C C C C C . C . . C . C C C C C . . C . C C C C C . . . . . . . . . . . . . . . . . . . C . . . . . . . . . . . . . . . . . . . . . . . . . . . C . . . . . . . . . . . . . . . . . . . . . . . . . . . . . . . . . .    
629	. . . . . . . . . . . . . . C C C C C C C C C C C C . C C C C C C C C C C . C . . C . C C C C C . . C . C C C C C . . . . . . . . . . . . . . . . . . . C . . . . . . . . . . . . . . . . . . . . . . . . . . . C . . . . . . . . . . . . . . . . . . . . . . . . . . . . . . . . . .    
630	. . . . . . . . . . . . . . C C C C C C C C C C C C . C C C C C C C C C C . C . . C . C C C C C . . C . C C C C C . . . . . . . . . . . . . . . . . . . C . . . . . . . . . . . . . . . . . . . . . . . . . . . C . . . . . . . . . . . . . . . . . . . . . . . . . . . . . . . . . .    
631	. . . . . . . . . . . . . . A A A A A A A A A A A A . A A A A A A A A A A . A . . A . A A A A A . . A . A A A A A . . . . . . . . . . . . . . . . . . . A . . . . . . . . . . . . . . . . . . . . . . . . . . . A . . . . . . . . . . . . . . . . . . . . . . . . . . . . . . . . . .    
632	. . . . . . . . . . . . . . T T T T T T T T T T T T . T T T T T T T T T T . T . . T . T T T T T . . T . T T T T T . . . . . . . . . . . . . . . . . . . T . . . . . . . . . . . . . . . . . . . . . . . . . . . T . . . . . . . . . . . . . . . . . . . . . . . . . . . . . . . . . .    
633	. . . . . . . . . . . . . . G G G G G G G G G G G G . G G G G G G G G G G . G . . G . G G G G G . . G . G G G G G . . . . . . . . . . . . . . . . . . . G . . . . . . . . . . . . . . . . . . . . . . . . . . . G . . . . . . . . . . . . . . . . . . . . . . . . . . . . . . . . . .    
634	. . . . . . . . . . . . . . G G G G G G G G G G G G . G G G G G G G G G G . G . . G . G G G G G . . G . G G G G G . . . . . . . . . . . . . . . . . . . G . . . . . . . . . . . . . . . . . . . . . . . . . . . G . . . . . . . . . . . . . . . . . . . . . . . . . . . . . . . . . .    
635	. . . . . . . . . . . . . . T T T T T T T T T T T T . T T T T T T T T T T . T . . T . T T T T T . . T . T T T T T . . . . . . . . . . . . . . . . . . . T . . . . . . . . . . . . . . . . . . . . . . . . . . . T . . . . . . . . . . . . . . . . . . . . . . . . . . . . . . . . . .    
636	. . . . . . . . . . . . . . G G G G G G G G G G G G . G G G G G G G G G G . G . . G . G G G G G . . G . G G G G G . . . . . . . . . . . . . . . . . G G G . . . . . . . . . . . . . . . . . . . . . . . . . . . G . . . . . . . . . . . . . . . . . . . . . . . . . . . . . . . . . .    
637	. . . . . . . . . . . . . . T T T T T T T T T T T T . T T T T T T T T T T . T . . T . T T T T T . . T . T T T T T . . . . . . . . . . . . . . . . . T T T . . . . . . . . . . . . . . . . . . . . . . . . . . . T . . . . . . . . . . . . . . . . . . . . . . . . . . . . . . . . . .    
638	. . . . . . . . . . . . . . A A A A A A A A A A A A . A A A A A A A A A A . A . . A . A A A A A . . A . A A A A A . . . . . . . . . . . . . . . . . A A A . . . . . . . . . . . . . . . . . . . . . . . . . . . A . . . . . . . . . . . . . . . . . . . . . . . . . . . . . . . . . .    
639	. . . . . . . . . . . . . . T T T T T T T T T T T T . T T T T T T T T T T . T . . T . T T T T T . . T . T T T T T . . . . . . . . . . . . . . . . . T T T . . . . . . . . . . . . . . . . . . . . . . . . . . T T . . . . . . . . . . . . . . . . . . . . . . . . . . . . . . . . . .    
640	. . . . . . . . . . . . . . C C C C C C C C C C C C . C C C C C C C C C C . C . . C . C C C C C C . C . C C C C C . . . . . . . . . . . . . . . . . C C C . . . . . . . . . . . . . . . . . . . . . . C . . . C C . . . . . . . . . . . . . . . . . . . . . . . . . . . . . . . . . .    
641	. . . . . . . . . . . . . . A A A A A A A A A A A A . A A A A A A A A A A . A . . A . A A A A A A . A . A A A A A . . . . . . . . . . . . . . . . . A A A . . . . . . . . . . . . . . . . . . . . . . A . . . A A . . . . . . . . . . . . . . . . . . . . . . . . . . . . . . . . . .    
642	. . . . . . . . . . . . . . C C C C C C C C C C C C . C C C C C C C C C C . C . . C . C C C C C C . C . C C C C C . . . . . . . . . . . . . . . . . C C C . . . . . . . . . . . . . . . . . . . . . . C . . . C C . . . . . . . . . . . . . . . . . . . . . . . . . . . . . . . . . .    
643	. . . . . . . . . . . . . . T T T T T T T T T T T T . T T T T T T T T T T . T . . T . T T T T T T . T . T T T T T . . . . . . . . . . . . . . . . . T T T . . . . . . . . . . . . . . . . . . . . . . T . . . T T . . . . . . . . . . . . . . . . . . . . . . . . . . . . . . . . . .    
644	. . . . . . . . . . . . . . . G G G G G G G G G G G . G G G G G G G G G G . G . . G . G G G G G G . G . G G G G G . . . . . . . . . . . . . . . . . G G G . . . . . . . . . . . . . . . . . . . . . . G . . . G G . . . . . . . . . . . . . . . . . . . . . . . . . . . . . . . . . .    
645	. . . . . . . . . . . . . . . G G G G G G G G G N G . G G G G G G G G G G . G . . G . G G G G G G . G . G G G G G . . . . . . . . . . . . . . . . . G G G . . . . . . . . . . . . . . . . . . . . . . G . . . G G . . . . . . . . . . . . . . . . . . . . . . . . . . . . . . . . . .    
646	. . . . . . . . . . . . . . . G G G G G G G G G G G . G G G G G G G G G G . G . . G . G G G G G G . G . G G G G G . . . . . . . . . . . . . . . . . G G G . . . . . . . . . . . . . . . . . . . . . . G . . . G G . . . . . . . . . . . . . . . . . . . . . . . . . . . . . . . . . .    
647	. . . . . . . . . . . . . . . A A A A A A A A A A A . A A A A A A A A A A . A . . A . A A A A A A . A . A A A A A . . . . . . . . . . . . . . . . . A A A . . . . . . . . . . . . . . . . . . . . . . A . . . A A . A . . . . . . . . . . . . . . . . . . . . . . . . . . . . . . . .    
648	. . . . . . . . . . . . . . . C C C C C C C C C C C . C C C C C C C C C C . C . . C . C C C C C C . C . C C C C C . . . . . . . . . . . . . . . . . C C C . . . . . . . . . . . . . . . . . . . . . . C . . . C C . C . . . . . . . . . . . . . . . . . . . . . . . . . . . . . . . .    
649	. . . . . . . . . . . . . . . C C C C C C C C C C C . C C C C C C C C C C . C . . C . C C C C C C . C . C C C C C . . . . . . . . . . . . . . . . . C C C . . . . . . . . . . . . . . . . . . . . . . C . . . C C . C . . . . . . . . . . . . . . . . . . . . . . . . . . . . . . . .    
650	. . . . . . . . . . . . . . . C C C C C C C C C C C . C C C C C C C C C C . C . . C . C C C C C C . C . C C C C C . . . . . . . . . . . . . . . . . C C C . . . . . . . . . . . . . . . . . . . . . . C . . . C C . C . . . . . . . . . . . . . . . . . . . . . . . . . . . . . . . .    
651	. . . . . . . . . . . . . . . G G G G G G G G G G G . G G G G G G G G G G . G . . G . G G G G G G . G . G G G G G . . . . . . . . . . . . . . . . . G G G . . . . . . . . . . . . . . . . . . . . . . G . . . G G . G . . . . . . . . . . . . . . . . . . . . . . . . . . . . . . . .    
652	. . . . . . . . . . . . . . . T T T T T T T T C T T . T T T T T T T T T T . T . . T . T T T T T T . T . T T T T T . . . . . . . . . . . . . . . . . T T T . . . . . . . . . . . . . . . . . . . . . . T . . . T T . T . . . . . . . . . . . . . . . . . . . . . . . . . . . . . . . .    
653	. . . . . . . . . . . . . . . C C C C C C C C C C C . C C C C C C C C C C . C . . C . C C C C C C . C . C C C C C . . . . . . . . . . . . . . . . . C C C . . . . . . . . . . . . . . . . . . . . . . C . . . C C . C . . . . . . . . . . . . . . . . . . . . . . . . . . . . . . . .    
654	. . . . . . . . . . . . . . . C C C C C C C C C C C . C C C C C C C C C C . C . . C . C C C C C C . C . C C C C C . . . . . . . . . . . . . . . . . C C C . . . . . . . . . . . . . . . . . . . . . . C . . . C C . C . . . . . . . . . . . . . . . . . . . . . . . . . . . . . . . .    
655	. . . . . . . . . . . . . . . T C C C C C C C C C C . C C C C C C C C C C . C . . C . C C C C C C . C . C C C C C . . . . . . . . . . . . . . . . . C C C . . . . . . . . . . . . . . . . . . . . . . C . . . C C . C . . . . . . . . . . . . . . . . . . . . . . . . . . . . . . . .    
656	. . . . . . . . . . . . . . . . T T T T T T T T T T . T T T T T T T T T T . T . . T . T T T T T T . T . T T T T T . . . . . . . . . . . . . . . . . T T T . . . . . . . . . . . . . . . . . . . . . . T . . . T T . T . . . . . . . . . . . . . . . . . . . . . . . . . . . . . . . .    
657	. . . . . . . . . . . . . . . . G G G G G G G G G G . G G G G G G G G G G . G . . G . G G G G G G . G . G G G G G . . . . . . . . . . . . . . . . . G G G . . . . . . . . . . . . . . . . . . . . . . G . . . G G . G . . . . . . . . . . . . . . . . . . . . . . . . . . . . . . . .    
658	. . . . . . . . . . . . . . . . C C C C C C C C C C . C C C C C C C C C C . C . . C . C C C C C C . C . C C C C C . . . . . . . . . . . . . . . . . C C C . . . . . . . . . . . . . . . . . . . . . . C . . . C C . C . . . . . . . . . . . . . . . . . . . . . . . . . . . . . . . .    
659	. . . . . . . . . . . . . . . . C C C C C C C C C C . C C C C C C C C C C . C . . C . C C C C C C . C . C C C C C . . . . . . . . . . . . . . . . . C C C . . . . . . . . . . . . . . . . . . . . . . C . . . C C . C . . . . . . . . . . . . . . . . . . . . . . . . . . . . . . . .    
660	. . . . . . . . . . . . . . . . T T T T T T T T T T . T T T T T T T T T T . T . . T . T T T T T T . T . T T T T T . . . . . . . . . . . . . . . . . T T T . . . . . . . . . . . . . . . . . . . . . . T . . . T T . T . . . . . . . . . . . . . . . . . . . . . . . . . . . . . . . .    
661	. . . . . . . . . . . . . . . . G G G G G G G G G G . G G G G G G G G G G . G . . G . G G G G G G . G . G G G G G . . . . . . . . . . . . . . . . . G G G . . . . . . . . . . . . . . . . . . . . . . G . . . G G . G . . . . . . . . . . . . . . . . . . . . . . . . . . . . . . . .    
662	. . . . . . . . . . . . . . . . A A A A A A A A A A . A A A A A A A A A A . A . . A . A A A A A A . A . A A A A A . . . . . . . . . . . . . . . . . A A A . . . . . . . . . . . . . . . . . . . . . . A . . . A A . A . . . . . . . . . . . . . . . . . . . . . . . . . . . . . . . .    
663	. . . . . . . . . . . . . . . . G G G G G G G G G G . G G G G G G G G G G . G . . G . G G G G G G . G . G G G G G . . . . . . . . . . . . . . . . . G G G . . . . . . . . . . . . . . . . . . . . . . G . . . G G . G . . . . . . . . . . . . . . . . . . . . . . . . . . . . . . . .    
664	. . . . . . . . . . . . . . . . G G G G G G G G G G . G G G G G G G G G G . G . . G . G G G G G G . G . G G G G G . . . . . . . . . . . . . . . . . G G G . . . . . . . . . . . . . . . . . . . . . . G . . . G G . G . . . . . . . . . . . . . . . . . . . . . . . . . . . . . . . .    
665	. . . . . . . . . . . . . . . . C C C C C C C C C C . C C C C C C C C C C . C . . C . C C C C C C . C . C C C C C . . . . . . . . . . . . . . . . . C C C . . . . . . . . . . . . . . . . . . . . . . C . . . C C . C . . . . . . . . . . . . . . . . . . . . . . . . . . . . . . . .    
666	. . . . . . . . . . . . . . . . T T T T T T T C T T . T T T T T T T T T T . T . . T . T T T T T T . T . T T T T T . . . . . . . . . . . . . . . . . T T T . . . . . . . . . . . . . . . . . . . . . . T . . . T T . T . . . . . . . . . . . . T . . . . . . . . . . . . . . . . . . .    
667	. . . . . . . . . . . . . . . . G G G G G G G G G G . G G G G G G G G G G . G . . G . G G G G G G . G . G G G G G . . . . . . . . . . . . . . . . . G G G . . . . . . . . . . . . . . . . . . . . . . G . . . G G . G . . . . . . . . . . . . G . . . . . . . . . . . . . . . . . . .    
668	. . . . . . . . . . . . . . . . A A A A A A A A A A . A A A A A A A A A A . A . . A . A A A A A A . A . A A A A A . . . . . . . . . . . . . . . A . A A A . . . . . . . . . . . . . . . . . . . . . . A . . . A A . A . . . . . . . . . . . . A A . . . . . . . . . . . . . . . . . .    
669	. . . . . . . . . . . . . . . . G A G G G G G G G G . G G G G G G G G G G . G . . G . G G G G G G . G . G G G G G . . . . . . . . . . . . . . . G . G G G . . . . . . . . . . . . . . . . . . . . . . G . . . G G . G . . . . . . . . . . . . G G . . . . . . . . . . . . . . . . . .    
670	. . . . . . . . . . . . . . . . A . A A A A A A A A A A A A A A A A A A A . A . . A . A A A A A A . A . A A A A A . . . . . . . . . . . . . . . A . A A A . . . . . . . . . . . . . . . . . . . . . . A A . . A A . A . . . . . . . . . . . . A A . . . . . . . . . . . . . . . . . .    
671	. . . . . . . . . . . . . . . . G . G G G G G G G G G G G G G G G G G G G . G . . G . G G G G G G . G . G G G G G . . . . . . . . . . . . . . . G . G G G . . . . . . . . . . . . . . . . . . . . . . G G . . G G . G . . . . . . . . . . . . G G . . . . . . . . . . . . . . . . . .    
672	. . . . . . . . . . . . . . . . G . G G G G G G G G G G G G G G G G G G G . G . . G . G G G G G G . G . G G G G G . . . . . . . . . . . . . . . G . G G G . . . . . . . . . . . . . . . . . . . . . . G G . . G G . G . . . . . . . . . . . . G G . . . . . . . . . . . . . . . . . .    
673	. . . . . . . . . . . . . . . . A . A A A A A A A A A A A A A A A A A A A . A . . A . A A A A A A . A . A A A A A . . . . . . . . . . . . . . . A . A A A . . . . . . . . . . . . . . . . . . . . A . A A . . A A . A . . . . . . . . . . . . A A . . . . . . . . . . . . . . . . . .    
674	. . . . . . . . . . . . . . . . A . A A A A A A A A A A A A A A A A A A A . A . . A . A A A A A A . A . A A A A A . . . . . . . . . . . . . . . A . A A A . . . . . . . . . . . . . . . . . . . . A . A A A . A A . A . . . . . . . . . . . . A A . . . . . . . . . . . . . . . . . .    
675	. . . . . . . . . . . . . . . . T . T T T T T T T T T T T T T T T T T T T . T . . T . T T T T T T . T . T T T T T . . . . . . . . . . . . . . . T T T T T . . . . . . . . . . . . . . . . . . . . T . T T T . T T T T . . . . . . . . . . . T T T . . . . . . . . . . . . . . . . . .    
676	. . . . . . . . . . . . . . . . A . A A A A A A A A A A A A A A A A A A A . A . . A . A A A A A A . A . A A A A A . . . . . . . . . . . . . . . A A A A A . . . . . . . . . . . . . . . . . . . . A . A A A . A A A A . . . . . . . . . . . A A A . . . . . . . . . . . . . . . . . .    
677	. . . . . . . . . . . . . . . . A . A A A A A A A A A A A A A A A A A A A . A . . A . A A A A A A . A . A A A A A . . . . . . . . . . . . . . . A A A A A . . . . . . . . . . . . . . . . . . . . A . A A A . A A A A . . . . . . . . . . . A A A . . . . . . . . . . . . . . . . . .    
678	. . . . . . . . . . . . . . . . G . G G G G G G G G G G G G G G G G G G G . G . . G . G G G G G G . G . G G G G G . . . . . . . . . . . . . . . G G G G G . . . . . . . . . . . . . . . . . . . . G . G G G . G G G G . . . . . . . . . . . G G G . . . . . . . . . . . . . . . . . .    
679	. . . . . . . . . . . . . . . . A . A A A A A A A A A A A A A A A A A A A . A . . A . A A A A A A . A . A A A A A . . . . . . . . . . . . . . . A A A A A . . . . . . . . . . . . . . . . . . . . A . A A A . A A A A . . . . . . . . . . . A A A . . . . . . . . . . . . . . . . . .    
680	. . . . . . . . . . . . . . . . T . T T T T T T T T T T T T T T T T T T T . T . . T . T T T T T T . T . T T T T T . . . . . . . . . . . . . . . T T T T T . . . . . . . . . . . . . . . . . . . . T . T T T . T T T T . . . . . . . . . . . T T T . . . . . . . . . . . . . . . . . .    
681	. . . . . . . . . . . . . . . . A . A A A A A A A A A A A A A A A A A A A . A . . A . A A A A A A . A . A A A A A . . . . . . . . . . . . . . . A A A A A . . . . . . . . . . . . . . . . . . . . A . A A A . A A A A . . . . . . . . . . . A A A . . . . . . . . . . . . . . . . . .    
682	. . . . . . . . . . . . . . . . G . G G G G G G G G G G G G G G G G G G G . G . . G . G G G G G G . G . G G G G G . . . . . . . . . . . . . . . G G G G G . . . . . . . . . . . . . . . . . . . . G . G G G . G G G G . . . . . . . . . . . G G G . . . . . . . . . . . . . . . . . .    
683	. . . . . . . . . . . . . . . . C . C C C C C C C C C C C C C C C C C C C . C . . C . C C C C C C . C . C C C C C . . . . . . . . . . . . . . . C C C C C . . . . . . . . . . . . . . . . . . . . C . C C C . C C C C . . . . . . . . . . . C C C . . . . . . . . . . . . . . . . . .    
684	. . . . . . . . . . . . . . . . C . C C C C C C C C C C C C C C C C C C C . C . . C . C C C C C C . C . C C C C C . . . . . . . . . . . . . . . C C C C C . . . . . . . . . . . . . . . . . . . . C . C C C . C C C C . . . . . . . . . . . C C C . . . . . . . . . . . . . . . . . .    
685	. . . . . . . . . . . . . . . . A . A A A A A A A A A A A A A A A A A A A . A . . A . G A A A A A . A . A A A A A . . . . . . . . . . . . . . . A A A A A . . . . . . . . . . . . . . . . . . . . A . A A A . A A A G . . . . . . . . . . . A A A . . . . . . . . . . . . . . . . . .    
686	. . . . . . . . . . . . . . . . T . T T T T T T T T T T T T T T T T T T T . T . . T . T T T T T T . T . T T T T T . . . . . . . . . . . . . . . T T T T T . . . . . . . . . . . . . . . . . . . . T . T T T . T T T T . . . . . . . . . . . T T T . . . . . . . . . . . . . . . . . .    
687	. . . . . . . . . . . . . . . . C . C C C C C C C C C C C C C C C C C C C . C . . C . C C C C C C . C . C C C C C . . . . . . . . . . . . . . . C C C C C . . . . . . . . . . . . . . . . . . . . C . C C C . C C C C . . . . . . . . . . . C C C . . . . . . . . . . . . . . . . . .    
688	. . . . . . . . . . . . . . . . G . G G G G G G G G G G G G G G G G G G G . G . . G . G G G G G G . G . G G G G G . . . . . . . . . . . . . . . G G G G G . . . . . . . . . . . . . . . . . . . . G . G G G . G G G G . . . . . . . . . . . G G G . . . . . . . . . . . . . . . . . .    
689	. . . . . . . . . . . . . . . . G . G G G G G G G G G G G G G G G G G G G . G . . G . G G G G G G . G . G G G G G . . . . . . . . . . . . . . . G G G G G . . . . . . . . . . . . . . . . . . . . G . G G G . G G G G . . . . . . . . . . . G G G . . . . . . . . . . . . . . . . . .    
690	. . . . . . . . . . . . . . . . T . T T T T T T T T T T T T T T T T T T T . T . . T . T T T T T T . T . T T T T T . . . . . . . . . . . . . . . T T T T T . . . . . . . . . . . . . . . . . . . T T . T T T . T T T T . . . . . . . . . . . T T T . . . . . . . . . . . . . . . . . .    
691	. . . . . . . . . . . . . . . . G . G G G G G G G G G G G G G G G G G G G . G . . G . G G G G G G . G . G G G G G . . . . . . . . . . . . . . . G G G G G . . . . . . . . . . . . . . . . . . . G G . G G G . G G G G . . . . . . . . . . . G G G . . . . . . . . . . . . . . . . . .    
692	. . . . . . . . . . . . . . . . C . C C C C C C C C C C C C C C C C C C C . C . . C . C C C C C C . C . C C C C C . . . . . . . . . . . . . . . C C C C C . . . . . . . . . . . . . . . . . . . C C . C C C . C C C C . . . . . . . . . . . C C C . . . . . . . . . . . . . . . . . .    
693	. . . . . . . . . . . . . . . . G . G G G G G G G G G G G G G G G G G G G . G . . G . G G G G G G . G . G G G G G . . . . . . . . . . . . . . . G G G G G . . . . . . . . . . . . . . . . . . . G G . G G G . G G G G . . . . . . . . . . . G G G . . . . . . . . . . . . . . . . . .    
694	. . . . . . . . . . . . . . . . T . T T T T T T T T T T T T T T T T T T T . T . . T . T T T T T T . T . T T T T T . . . . . . . . . . . . . . . T T T T T . . . . . . . . . . . . . . . . . . . T T . T T T . T T T T . . . . . . . . . . . T T T . . . . . . . . . . . . . . . . . .    
695	. . . . . . . . . . . . . . . . C . C C C C C C C C C C C C C C C C C C C . C . . C . C C C C C C . C . C C C C C . . . . . . . . . . . . . . . C C C C C . . . . . . . . . . . . . . . . . . . C C . C C C . C C C C . . . . . . . . . . C C C C . . . . . . . . . . . . . . . . . .    
696	. . . . . . . . . . . . . . . . T . T T T T T T T T T T T T T T T T T T T . T . . T . T T T T T T . T . T T T T T . . . . . . . . . . . . . . . T T T T T . . . . . . . . . . . . . . . . . . . T T . T T T . T T T T . . . . . . . . . . T T T T . . . . . . . . . . . . . . . . . .    
697	. . . . . . . . . . . . . . . . G . . G G G G G G G G G G G G G G G G G G . G . . G . G G G G G G . G . G G G G G . . . . . . . . . . . . . . . G G G G G . . . . . . . . . . . . . . . . . . . G G . G G G . G G G G . . . . . . . . . . G G G G . . . . . . . . . . . . . . . . . .    
698	. . . . . . . . . . . . . . . . G . . G G G G G G G G G G G G G G G G G G . G . . G . G G G G G G . G . G G G G G . . . . . . . . . . . . . . . G G G G G . . . . . . . . . . . . . . . . . . . G G . G G G . G G G G . . . . . . . . . . G G G G G . . . . . . . . . . . . . . . . .    
699	. . . . . . . . . . . . . . . . T . . T T T T T T T T T T T T T T T T T T . T . . T . T T T T T T . T . T T T T T . . . . . . . . . . . . . . . T T T T T . . . . . . . . . . . . . . . . . . . T T . T T T . T T T T . . . . . . . . . . T T T T T . . . . . . . . . . . . . . . . .    
700	. . . . . . . . . . . . . . . . C . . C C C C C C C C C C C C C C C C C C . C . . C . C C C C T C . C . C C C C C . . . . . . . . . . . . . . . C C C C C . . . . . . . . . . . . . . . . . . . C C . C C C . C C C C . . . . . . . . . . C C C C C . . . . . . . . . . . . . . . . .    
701	. . . . . . . . . . . . . . . . T . . T T T T T T T T T T T T T T T T T T . T . . T . T T T T T T . T . T T T T T . . . . . . . . . . . . . . . T T T T T . . . . . . . . . . . . . . . . . . . T T . T T T . T T T T . . . . . . . . . . T T T T T . . . . . . . . . . . . . . . . .    
702	. . . . . . . . . . . . . . . . G . . G G G G G G G G G G G G G G G G G G . G . . G . G G G G G G . G . G G G G G . . . . . . . . . . . . . . . G G G G G . . . . . . . . . . . . . . . . . . . G G . G G G . G G G G . . . . . . . . . . G G G G G . . . . . . . . . . . . . . . . .    
703	. . . . . . . . . . . . . . . . G . . G G G G G G G G G G G G G G G G G G . G . . G . G G G G G G . G . G G G G G . . . . . . . . . . . . . . . G G G G G . . . . . . . . . . . . . . . . . . . G G . G G G . G G G G . . . . . . . . . . G G G G G . . . . . . . . . . . . . . . . .    
704	. . . . . . . . . . . . . . . . T . . T T T T T T T T T T T T T T T T T T . T . . T . T T T T T T . T . T T T T T . . . . . . . . . . . . . . . T T T T T . . . . . . . . . . . . . . . . . . . T T . T T T . T T T T . . . . . . . . . . T T T T T . . . . . . . . . . . . . . . . .    
705	. . . . . . . . . . . . . . . . G . . G G G G G G G G G G G G G G G G G G . G . . G . G G G G G G . G . G G G G G . . . . . . . . . . . . . . . G G G G G . . . . . . . . . . . . . . . . . G G G G . G G G . G G G G . . . . . . . . . . G G G G G . . . . . . . . . . . . . . . . .    
706	. . . . . . . . . . . . . . . . C . . C C C C C C C C C C C C C C C C C C . C . . C . C C C C C C . C . C C C C C . . . . . . . . . . . . . . . C C C C C . . . . . . . . . . . . . . . . . C C C C . C C C . C C C C . . . . . . . . . . C C C C C . . . . . . . . . . . . . . . . .    
707	. . . . . . . . . . . . . . . . T . . T T T T T T T T T T T T T T T T T T . T . . T . T T T T T T . T . T T T T T . . . . . . . . . . . . . . . T T T T T . . . . . . . . . . . . . . . . . T T T T . T T T . T T T T . . . . . . . . . . T T T T T . . . . . . . . . . . . . . . . .    
708	. . . . . . . . . . . . . . . . . . . G G G G G G G G G G G G G G G G G G . G . . G . G G G G G G . G . G G G G G . . . . . . . . . . . . . . . G G G G G . . . . . . . . . . . . . . . G . G G G G . G G G . G G G G . . . . . . . . . . G G G G G . . . . . . . . . . . . . . . . .    
709	. . . . . . . . . . . . . . . . . . . G G G G G G G G G G G G G G G G G G . G . . G . G G G G G G . G . G G G G G . . . . . . . . . . . . . . . G G G G G . . . . . . . . . . . . . . . G . G G G G G G G G . G G G G . . . . . . . . . . G G G G G . . . . . . . . . . . . . . . . .    
710	. . . . . . . . . . . . . . . . . . . G G G G G G G G G G G G G G G G G G . G . . G . G G G G G G . G . G G G G G . . . . . . . . . . . . . . . G G G G G . . . . . . . . . . . . . . . G . G G G G G G G G . G G G G . . . . . . . . . . G G G G G . . . . . . . . . . . . . . . . .    
711	. . . . . . . . . . . . . . . . . . . . A A A A A A A A A A A A A A A A A . A . . A . A A A A A A . A . A A A A A . . . . . . . . . . . . . . . A A A A A . . . . . . . . . . . . . . . A . A A A A A A A A . A A A A . . . . . . . . . . A A A A A . . . . . . . . . . . . . . . . .    
712	. . . . . . . . . . . . . . . . . . . . G G G G G G G G G G G G G G G G G . G . . G . G G G G G G . G . G G G G G . . . . . . . . . . . . . . . G G G G G . . . . . . . . . . . . . . . G . G G G G G G G G . G G G G . . . . . . . . . . G G G G G . . . . . . . . . . . . . . . . .    
713	. . . . . . . . . . . . . . . . . . . . C C C C C C C C C C C C C C C C C . C . . C . C C C C C C . C . C C C C C . . . . . . . . . . . . . . . C C C C C . . . . . . . . . . . . . . . C . C C C C C C C C . C C C C . . . . . . . . . . C C C C C . . . . . . . . . . . . . . . . .    
714	. . . . . . . . . . . . . . . . . . . . C C C C C C C C C C C C C C C C C . C . . C . C C C C C C . C . C C C C C . . . . . . . . . . . . . . . C C C C C . . . . . . . . . . . . . . . C C C C C C C C C C . C C C C . . . . . . . . . . C C C C C . . . . . . . . . . . . . . . . .    
715	. . . . . . . . . . . . . . . . . . . . A A A A A A A A A A A A A A A A A . A . . A . A A A A A A . A . A A A A A . . . . . . . . . . . . . . . A A A A A . . . . . . . . . . . . . . . A A A A A A A A A A . A A A A . . . . . . . . . . A A A A A . . . . . . . . . . . . . . . . .    
716	. . . . . . . . . . . . . . . . . . . . T T T T T T T T T T T T T T T T T . T . . T . T T T T T T . T . T T T T T . . . . . . . . . . . . . . . T T T T T . . . . . . . . . . . . . . . T T T T T T T T T T . T T T T . . . . . . . . . . T T T T T . . . . . . . . . . . . . . . . .    
717	. . . . . . . . . . . . . . . . . . . . C C C C C C C C C C C C C C C C C . C . . C . C C C C C C . C . C C C C C . . . . . . . . . . . . . . . C C C C C . . . . . . . . . . . . . . . C C C C C C C C C C . C C C C . . . . . . . . . . C C C C C . . . . . . . . . . . . . . . . .    
718	. . . . . . . . . . . . . . . . . . . . T T T T T T T T T T T T T T T T T . T . . T . T T T T T T . T . T T T T T . . . . . . . . . . . . . . . T T T T T . . . . . . . . . . . . . . . T T T T T T T T T T . T T T T . . . . . . . . . T T T T T T . . . . . . . . . . . . . . . . .    
719	. . . . . . . . . . . . . . . . . . . . T T T T T T T T T T T T T T T T T . T . . T . T T T T T T . T . T T T T T . . . . . . . . . . . . . . . T T T T T . . . . . . . . . . . . . . . T T T T T T T T T T . T T T T . . . . . . . . . T T T T T T . . . . . . . . . . . . . . . . .    
720	. . . . . . . . . . . . . . . . . . . . T A A A A A A A A A A A A A A A A . A . . A . A A A A A A . A . A A A A A . . . . . . . . . . . . . . . A A A A A . . . . . . . . . . . . . . . A A A A A A A A A A . A A A A . . . . . . . . . A A A A A A . . . . . . . . . . . . . . . . .    
721	. . . . . . . . . . . . . . . . . . . . . G G G G G G G G G G G G G G G G . G . . G . G G G G G G . G . G G G G G . . . . . . . . . . . . . . . G G G G G . . . . . . . . . . . . . . . G G G G G G G G G G . G G G G . . . . . . . . . G G G G G G . . . . . . . . . . . . . . . . .    
722	. . . . . . . . . . . . . . . . . . . . . C C C C C C C C C C C C C C C C . C . . C . C C C C C C . C . C C C C C . . . . . . . . . . . . . . . C C C C C . . . . . . . . . . . . . . . C C C C C C C C C C . C C C C . . . . . . . . . C C C C C C . . . . . . . . . . . . . . . . .    
723	. . . . . . . . . . . . . . . . . . . . . C C C C C C C C C C C C C C C C . C . . C . C C C C C C . C . C C C C C . . . . . . . . . . . . . . . C C C C C . . . . . . . . . . . . . . . C C C C C C C C C C . C C C C . . . . . . . . . C C C C C C . . . . . . . . . . . . . . . . .    
724	. . . . . . . . . . . . . . . . . . . . . T T C T T T T T T T T T T T T T . T . . T . T T T T T T . T . T T T T T . . . . . . . . . . . . . . . T T T T T . . . . . . . . . . . . . . . T T T T T T T T T T . T T T T . . . . . . . . . T T T T T T . . . . . . . . . . . . . . . . .    
725	. . . . . . . . . . . . . . . . . . . . . T T . T T T T T T T T T T T T T . T . . T . T T T T T T . T . T T T T T . . . . . . . . . . . . . . . T T T T T . . . . . . . . . . . . . . . T T T T T G G T T T . T T T T . . . . . . . . . T T T T T T . . . . . . . . . . . . . . . . .    
726	. . . . . . . . . . . . . . . . . . . . . G G . G G G G G G G G G G G G G . G . . G . G G G G G G . G . G G G G G . . . . . . . . . . . . . . . G G G G G . . . . . . . . . . . . . . . G G G G G G G G G G . G G G G . . . . . . . . . G G G G G G . . . . . . . . . . . . . . . . .    
727	. . . . . . . . . . . . . . . . . . . . . G G . G G G G G G G G G G G G G . G . . G . G G G G G G . G . G G G G G . . . . . . . . . . . . . . . G G G G G . . . . . . . . . . . . . . . G G G G G G G G G G . G G G G . . . . . . . . . G G G G G G . . . . . . . . . . . . . . . G .    
728	. . . . . . . . . . . . . . . . . . . . . C C . C C C C C C C C C C C C C . C . . C . C C C C C C . C . C C C C C . . . . . . . . . . . . . . . C C C C C . . . . . . . . . . . . . . . C C C C C C C C C C C C C C C . . . . . . . . . C C C C C C . . . . . . . . . . . . . . . C .    
729	. . . . . . . . . . . . . . . . . . . . . A A . A A A A A A A A A A A A A . A . . A . A A A A A A . A . A A A A A . . . . . . . . . . . . . . . A A A A A . . . . . . . . . . . . . . A A A A A A A A A A A A A A A A . . . . . . . . . A A A A A A . . . . . . . . . . . . . . . A .    
730	. . . . . . . . . . . . . . . . . . . . . G G . G G G G G G G G G G G G G . G . . G . G G G G G G . G . G G G G G . . . . . . . . . . . . . . . G G G G G . . . . . . . . . . . . . . G G G G G G G G G G G G G G G G . . . . . . . . . G G G G G G . . . . . . . . . . . . . . . G .    
731	. . . . . . . . . . . . . . . . . . . . . G G . G G G G G G G G G G G G G . G . . G . G G G G G G . G G G G G G G . . . . . . . . . . . . . . . G G G G G . . . . . . . . . . . . . . G G G G G G G G G G G G G G G G . . . . . . . . . G G G G G G . . . . . . . . . . . . . . . G .    
732	. . . . . . . . . . . . . . . . . . . . . A A . A A A A A A A A A A A A A . A . . A . A A A A A A . A A A A A A A . . . . . . . . . . . . . . . A A A A A . . . . . . . . . . . . . . A A A A A A A A A A A A A A A A . . . . . . . . . A A A A A A . . . . . . . . . . . . . . . A .    
733	. . . . . . . . . . . . . . . . . . . . . C C . C C C C C C C C C C C C C . C . . C . C C C C C C . C C C C C C C . . . . . . . . . . . . . . . C C C C C . . . . . . . . . . . . . . C C C C C C C C C C C C C C C C . . . . . . . . . C C C C C C . . . . . . . . . . . . . . . C .    
734	. . . . . . . . . . . . . . . . . . . . . T T . T T T T T T T T T T T T T . T . . T . T T T T T T . T T T T T T T . . . . . . . . . . . . . . . T T T T T . . . . . . . . . . . . . . T T T T T T T T T T T T T T T T . . . . . . . . . T T T T T T . . . . . . . . . . . . . . . T .    
735	. . . . . . . . . . . . . . . . . . . . . C C . C C C C C C C C C C C C C . C . . C . C C C C C C . C C C C C C C . . . . . . . . . . . . . . . C C C C C . . . . . . . . . . . . . . C C C C C C C C C C C C C C C C . . . . . . . . . C C C C C C . . . . . . . . . . . . . . . C .    
736	. . . . . . . . . . . . . . . . . . . . . A A . A A A A A A A A A A A A A . A . . A . A A A A A A . A A A A A A A . . . . . . . . . . . . . . . A A A A A . . . . . . . . . . . . . . A A A A A A A A A A A A A A A A . . . . . . . . . A A A A A A . . . . . . . . . . . . . . . A .    
737	. . . . . . . . . . . . . . . . . . . . . T T . T T T T T T T T T T T T T . T . . T . T T T T T T . T T T T T T T . . . . . . . . . . . . . . . T T T T T . . . . . . . . . . . . . . T T T T T T T T T T T T T T T T . . . . . . . . . T T T T T T . . . . . . . . . . . . . . . T .    
738	. . . . . . . . . . . . . . . . . . . . . C C . C C C C C C C C C C C C C . C . . C . C C C C C C . C C C C C C C . . . . . . . . . . . . . . . C C C C C . . . . . . . . . . . . . . C C C C C C C C C C C C C C C C . . . . . . . . . C C C C C C . . . . . . . . . . . . . . . C .    
739	. . . . . . . . . . . . . . . . . . . . . T T . T T T T T T T T T T T T T . T . . T . T T T T T T . T T T T T T T . . . . . . . . . . . . . . . T T T T T . . . . . . . . . . . . . . T T T T T T T T T T T T T T T T . . . . . . . . . T T T T T T . . . . . . . . . . . . . . . T .    
740	. . . . . . . . . . . . . . . . . . . . . A A . A A A A A A A A A A A A A . A . . A . A A A A A A . A A A A A A A . . . . . . . . . . . . . . . A A A A A . . . . . . . . . . . . . . A A A A A A A A A A A A A A A A . . . . . . . . . A A A A A A . . . . . . . . . . . . . . . A .    
741	. . . . . . . . . . . . . . . . . . . . . C C . C C C C C C C C C C C C C . C . . C . C C C C C C . C C C C C C C . . . . . . . . . . . . . . . C T C C C . . . . . . . . . . . . . . C C C C C C C C C C C C C C C C . . . . . . . . . C C C C C C . . . . . . . . . . . . . . . C .    
742	. . . . . . . . . . . . . . . . . . . . . T T . T T T T T T T T T T T T T . T . . T . T T T T T T . T T T T T T T . . . . . . . . . . . . . . . T T T T T . . . . . . . . . . . . . . T T T T T T T T T T T T T T T T . . . . . . . . . T T T T T T . . . . . . . . . . . . . . . T .    
743	. . . . . . . . . . . . . . . . . . . . . A A . A A A A A A A A A A A A A . A . . A . A A A A A A . A A A A A A A . . . . . . . . . . . . . . . A A A A A . . . . . . . . . . . . . . A A A A A A A A A A A A A A A A . . . . . . . . . A A A A A A . . . . . . . . . . . . . . . A .    
744	. . . . . . . . . . . . . . . . . . . . . C C . C C C C C C C C C C C C C . C . . C . C C C C C C . C C C C C C C . . . . . . . . . . . . . . . C C C C C . . . . . . . . . . . . . C C C C C C C C C C C C C C C C C . . . . . . . . . C C C C C C . . . . . . . . . . . . . . . C .    
745	. . . . . . . . . . . . . . . . . . . . . A A . A A A A A A A A A A A A A . A . . A . A A A A A A . A A A A A A A . . . . . . . . . . . . . . . A A A A A . . . . . . . . . . . . . A A A A A A A A A A A A A A A A A . . . . . . . . . A A A A A A . . . . . . . . . . . . . . . A .    
746	. . . . . . . . . . . . . . . . . . . . . A A . A . A A A A A A A A A A A . A . . A . A A A A A A . A A A A A A A . . . . . . . . . . . . . . . A A A A A . . . . . . . . . . . . . A A A A A A A A A A A A A A A A A . . . . . . . . . A A A A A A . . . . . . . . . . . . . . . A .    
747	. . . . . . . . . . . . . . . . . . . . . G G . G . G G G G G G G G G G G . G . . G . G G G G G G . G G G G G G G . . . . . . . . . . . . . . . G G G G G . . . . . . . . . . . . . G G G G G G G G G G G G G G G G G . . . . . . . . . G G G G G G . . . . . . . . . . . . . . . G .    
748	. . . . . . . . . . . . . . . . . . . . . A A . A . A A A A A A A A A A A . A . . A . A A A A A A . A A A A A A A . . . . . . . . . . . . . . . A A A A A . . . . . . . . . . . . . A A A A A A A A A A A A A A A A A . . . . . . . . . A A A A A A . . . . . . . . . . . . . . . A .    
749	. . . . . . . . . . . . . . . . . . . . . A A . A . A A A A A A A A A A A . A . . A . A A A A A A . A A A A A A A . . . . . . . . . . . . . . . A A A A A . . . . . . . . . . . . . A A A A A A A A A A A A A A A A A . . . . . . . . . A A A A A A . . . . . . . . . . . . . . . A .    
750	. . . . . . . . . . . . . . . . . . . . . G G . G . G G G G G G G G G G G . G . . G . G G G G G G . G G G G G G G . . . . . . . . . . . . . . . G G G G G . . . . . . . . . . . . . G G G G G G G G G G G G G G G G G . . . . . . . . . G G G G G G . . . . . . . . . . . . . . . G .    
751	. . . . . . . . . . . . . . . . . . . . . A A . A . A A A A A A A A A A A . A . . A . A A A A A A . A A A A A A A . . . . . . . . . . . . . . . A A A A A . . . . . . . . . . . . . A A A A A A A A A A A A A A A A A . . . . . . . . . A A A A A A . . . . . . . . . . . . . . . A .    
752	. . . . . . . . . . . . . . . . . . . . . A A . A . A A A A A A A A A A A . A . . A . A A A A A A . A A A A A A A . . . . . . . . . . . . . . . A A A A A . . . . . . . . . . . . . A A A A A A A A A A A A A A A A A . . . . . . . . . A A A A A A . . . . . . . . . . . . . . . A .    
753	. . . . . . . . . . . . . . . . . . . . . G G . G . G G G G G G G G G G G . G . . G . G G G G G G . G G G G G G G . . . . . . . . . . . . . . . G G G G G . . . . . . . . . . . . . G G G G G G G G G G G G G G G G G . . . . . . . . . G G G G G G . . . . . . . . . . . . . . . G .    
754	. . . . . . . . . . . . . . . . . . . . . T T . T . T T T T T T T T T T T . T . . T . T T T T T T . T T T T T T T . . . . . . . . . . . . . . . T T T T T . . . . . . . . . . . . . T T T T T T T T T T T T T T T T T . . . . . . . . . T T T T T T . . . . . . . . . . . . . . . T .    
755	. . . . . . . . . . . . . . . . . . . . . C C . C . C C C C C C C C C C C . C . . C . C C C C C C . C C C C C C C . . . . . . . . . . . . . . . C C C C C . . . . . . . . . . . . . C C C C C C C C C C C C C C C C C . . . . . . . . . C C C C C C . . . . . . . . . . . . . . . C .    
756	. . . . . . . . . . . . . . . . . . . . . T T . T . T T T T T T T T T T T . T . . T . T T T T T T . T T T T T T T . . . . . . . . . . . . . . . T T T T T . . . . . . . . . . . . . T T T T T T T T T T T T T T T T T . . . . . . . . . T T T T T T . . . . . . . . . . . . . . . T .    
757	. . . . . . . . . . . . . . . . . . . . . T T . T . T T T T T T T T T T T . T . . T . T T T T T T . T T T T T T T . . . . . . . . . . . . . . . T T T T T . . . . . . . . . . . . . T T T T T T T T T T T T T T T T T . . . . . . . . . T T T T T T . . . . . . . . . . . . . . . T .    
758	. . . . . . . . . . . . . . . . . . . . . C C . C . C C C C C C C C C C C . C . . C . C C C C C C . C C C C C C C . . . . . . . . . . . . . . . C C C C C . . . . . . . . . . . . . C C C C C C C C C C C C C C C C C . . . . . . . . . C C C C C C . . . . . . . . . . . . . . . C .    
759	. . . . . . . . . . . . . . . . . . . . . T T . T . T T T T T T T T T T T . T . . T . T T T T T T . T T T T T T T . . . . . . . . . . . . . . . T T T T T . . . . . . . . . . . . . T T T T T T T T T T T T T T T T T . . . . . . . . . T T T T T T . . . . . . . . . . . . . . . T .    
760	. . . . . . . . . . . . . . . . . . . . . G G . . . G G G G G G G G G G G . G . . G . G G G G G G . G G G G G G G . . . . . . . . . . . G G G G G G G G G . . . . . . . . . . . G . G G G G G G G G G G G G G G G G G . . . . . . . G . G G G G G G . . . . . . . . . . . . . . . G G    
761	. . . . . . . . . . . . . . . . . . . . . G G . . . G G G G G G G G G G G . G . . G . G G G G G G . G G G G G G G . . . . . . . . . . . G G G G G G G G G . . . . . . . . . . . G . G G G G G G G G G G G G G G G G G . . . . . . . G G G G G G G G . . . . . . . . . . . G . . G G G    
762	. . . . . . . . . . . . . . . . . . . . . G G . . . G G G G G G G G G G G . G . . G . G G G G G G . G G G G G G G . . . . . . . . . . . G G G G G G G G G . . . . . . . . . . . G . G G G G G G G G G G G G G G G G G . . . . . . . G G G G G G G G . . . . . . . . . . . G . . G G G    
763	. . . . . . . . . . . . . . . . . . . . . G G . . . G G G G G G G G G G G . G . . G . G G G G G G . G G G G G G G . . . . . . . . . . . G G G G G G G G G . . . . . . . . . . . G . G G G G G G G G G G G G G G G G G . . . . . . . G G G G G G G G . . . . . . . . . . . G . . G G G    
764	. . . . . . . . . . . . . . . . . . . . . T T . . . T T T T T T T T T T T . T . . T . T T T T T T . T T T T T T T . . . . . . . . . . . T T T T T T T T T . . . . . . . . . . . T . T T T T T T T T T T T T T T T T T . . . . . . . T T T T T T T T . . . . . . . . . . . T . . T T T    
765	. . . . . . . . . . . . . . . . . . . . . G G . . . G G G G G G G G G G G . G . . G . G G G G G G . G G G G G G G . . . . . . . . . . . G G G G G G G G G . . . . . . . . . . . G . G G G G G G G G G G G G G G G G G . . . . . . . G G G G G G G G . . . . . . . . . . . G . . G G G    
766	. . . . . . . . . . . . . . . . . . . . . C C . . . C C C C C C C C C C C . C . . C . C C C C C C . C C C C C C C . . . . . . . . . . C C C C C C C C C C . . . . . . . . . . C C . C C C C C C C C C C C C C C C C C . . . . . . . C C C C C C C C . . . . . . . . . . . C . . C C C    
767	. . . . . . . . . . . . . . . . . . . . . T T . . . T T T T T T T T T T T . T . . T . T T T T T T . T T T T T T T . . . . . . . . . . T T T T T T T T T T . . . . . . . . . T T T . T T T T T T T T T T T T T T T T T . . . . . . . T T T T T T T T . . . . . . . . . . . T . . T T T    
768	. . . . . . . . . . . . . . . . . . . . . G G . . . G G G G G G G G G G G . G . . G . G G G G G G . G G G G G G G . . . . . . . . . . G G G G G G G G G G . . . . . . . . . G G G G G G G G G G G G G G G G G G G G G . . . . . . . G G G G G G G G . . . . . . . . . . . G . . G G G    
769	. . . . . . . . . . . . . . . . . . . . . T T . . . T T T T T T T T T T T . T . . T . T T T T T T . T T T T T T T . . . . . . . . . . T T T T T T T T T T . . . . . . . . . T T T T T T T T T T T T T T T T T T T T T . . . . . . . T T T T T T T T . . . . . . . . . . . T . T T T T    
770	. . . . . . . . . . . . . . . . . . . . . A A . . . A A A A A A A A A A A . A . . A . A A A A A A . A A A A A A A . . . . . . . . . . A A A A A A A A A A . . . . . . . . A A A A A A A A A A A A A A A A A A A A A A . . . . . . . A A A A A A A A . . . . . . . . . . . A . A A A A    
771	. . . . . . . . . . . . . . . . . . . . . G G . . . G G G G G G G G G G G . G . . G . G G G G G G . G G G G G G G . . . . . . . . . . G G G G G G G G G G . . . . . . . . G G G G G G G G G G G G G G G G G G G G G G . . . . . . . G G G G G G G G . . . . . . . . . . . G . G G G G    
772	. . . . . . . . . . . . . . . . . . . . . T T . . . T T T T T T T T T T T . T . . T T T T T T T T . T T T T T T T . . . . . . . . . . T T T T T T T T T T . . . . . . . . T T T T T T T T T T T T T T T T T T T T T T . . . . . . . T T T T T T T T . . . . . . . . . . . T . T T T T    
773	. . . . . . . . . . . . . . . . . . . . . A A . . . A A A A A A A A A A A A A . A A A A A A A A A . A A A A A A A . . . . . . . . . . A A A A A A A A A A . . . . . . . . A A A A A A A A A A A A A A A A A A A A A A . . . . . . . A A A A A A A A . . . . . . . . . . . A . A A A A    
774	. . . . . . . . . . . . . . . . . . . . . C C . . . C C C C C C C C C C - C C . C C C C C C C C C . C C C C C C C . . . . . . . . . . C C C C C C C C C C . . . . . . . . C C C C C C C C C C C C C C C C C C C C C C . . . . . . . C C C C C C C C . . . . . . . . . . . C . C C C C    
775	. . . . . . . . . . . . . . . . . . . . . T T . . . C T T T T T T T T T - T T . T T T T T T T T T . T T T T T T T . . . . . . . . . . T T T T T T T T T T . . . . . . . . T T T T T T T T T T T T T T T T T T T T T T . . . . . . . T T T T T T T T . . . . . . . . . . . T . T T T T    
776	. . . . . . . . . . . . . . . . . . . . . A A . . . . A A A A A A A A A - A A . A A A A A A A A A . A A A A A A A . . . . . . . . . . A A A A A A A A A A . . . . . . . . A A A A A A A A A A A A A A A A A A A A A A . . . . . . . A A A A A A A A . . . . . . . . . . . A . A A A A    
777	. . . . . . . . . . . . . . . . . . . . . A A . . . . A A A A A A A A A A A A . A A A A A A A A A . A A A A A A A . . . . . . . . . . A A A A A A A A A A . . . . . . . . A A A A A A A A A A A A A A A A A A A A A A . . . . . . . A A A A A A A A . . . . . . . . . . . A . A A A A    
778	. . . . . . . . . . . . . . . . . . . . . A A . . . . G G G G G G G G G G G G . G G G G G G G G G . G G G G G G G . . . . . . . . . . G G G G G G G G G G . . . . . . . . G G G G G G G G G G G G G G G G G G G G G G . . . . . . . G G G G G G G G . . . . . . . . . . . G . G G G G    
779	. . . . . . . . . . . . . . . . . . . . . G G . . . . G G G G G G G G G G G G . G G G G G G G G G . G G G G G G G . . . . . . . . . . G G G G G G G G G G . . . . . . . . G G G G G G G G G G G G G G G G G G G G G G . . . . . . . G G G G G G G G . . . . . . . . . . . G . G G G G    
780	. . . . . . . . . . . . . . . . . . . . . C G . . . . C C C C C C C C C C C C . C C C C C C C C C . C C C C C C C . . . . . . . . . . C C C C C C C C C C . . . . . . . . C C C C C C C C C C C C C C C C C C C C C C . . . . . . . C C C C C C C C . . . . . . . . . . . C . C C C C    
781	. . . . . . . . . . . . . . . . . . . . . . . . . . . T T T T T T T T T T T T . T T T T T T T T T . T T T T T T T . . . . . . . . . . T T T T T T T T T T . . . . . . . . T T T T T T T T T T T T T T T T T T T T T T . . . . . . . T T T T T T T T . . . . . . . . . . . T . T T T T    
782	. . . . . . . . . . . . . . . . . . . . . . . . . . . T T T T T T T T T T T T . T T T T T T T T T . T T T T T T T . . . . . . . . . . T T T T T T T T T T . . . . . . . . T T T T T T T T T T T T T T T T T T T T T T . . . . . . . T T T T T T T T . . . . . . . . . . . T . T T T T    
783	. . . . . . . . . . . . . . . . . . . . . . . . . . . G G G G G G G G G G G G . G G G G G G G G G . G G G G G G G . . . . . . . . . . G G G G G G G G G G . . . . . . . . G G G G G G G G G G G G G G G G G G G G G G . . . . . . . G G G G G G G G . . . . . . . . . . . G . G G G G    
784	. . . . . . . . . . . . . . . . . . . . . . . . . . . A A A A A A A A A A A A . A A A A A A A A A . A A A A A A A . . . . . . . . . . A A A A A A A A A A . . . . . . . . A A A A A A A A A A A A A A A A A A A A A A . . . . . . . A A A A A A A A . . . . . . . . . . . A . A A A A    
785	. . . . . . . . . . . . . . . . . . . . . . . . . . . A A A A A A A A A A A A . A A A A A A A A A . A A A A A A A . . . . . . . . . . A A A A A A A A A A . . . . . . . . A A A A A A A A A A A A A A A A A A A A A A . . . . . . . A A A A A A A A . . . . . . . . . . . A . A A A A    
786	. . . . . . . . . . . . . . . . . . . . . . . . . . . G G G G G G G G G G G G . G G G G G G G G G . G G G G G G G . . . . . . . . . . G G G G G G G G G G . . . . . . . . G G G G G G G G G G G G G G G G G G G G G G . . . . . . . G G G G G G G G . . . . . . . . . G . G . G G G G    
787	. . . . . . . . . . . . . . . . . . . . . . . . . . . A A A A A A A A A A A A . A A A A A A A A A . A A A A A A A . . . . . . . . . . A A A A A A A A A A . . . . . . . . A A A A A A A A A A A A A A A A A A A A A A . . . . . . . A A A A A A A A . . . . . . . . . A . A . A A A A    
788	. . . . . . . . . . . . . . . . . . . . . . . . . . . C C C C C C C C C C C C . C C C C C C C C C . C C C C C C C . . . . . . . . . . C C C C C C C C C C . . . . . . . . C C C C C C C C C C C C C C C C C C C C C C . . . . . . . C C C C C C C C . . . . . . . . . C . C . C C C C    
789	. . . . . . . . . . . . . . . . . . . . . . . . . . . A A A A A A A A A A A A . A A A A A A A A A . A A A A A A A . . . . . . . . . . A A A A A A A A A A . . . . . . . . A A A A A A A A A A A A A A A A A A A A A A . . . . . . . A A A A A A A A . . . . . . . . . A . A . A A A A    
790	. . . . . . . . . . . . . . . . . . . . . . . . . . . C C C C C C C C C C C C . C C C C C C C C C . T C C C C C C . . . . . . . . . . C C C C C C C C C C . . . . . . . . C C C C C C C C C C C C C C C T T T C C C C . . . . . . . C C C C C C C C . . . . . . . . . C . C . C C C C    
791	. . . . . . . . . . . . . . . . . . . . . . . . . . . G G G G G G G G G G G G . G G G G G G G G G . G G G G G G G . . . . . . . . . . G G G G G G G G G G . . . . . . . . G G G G G G G G G G G G G G G G G G G G G G . . . . . . . G G G G G G G G . . . . . . . . . G G G . G G G G    
792	. . . . . . . . . . . . . . . . . . . . . . . . . . . T T T T T T T T T T T T . T T T T T T T T T . T T T T T T T . . . . . . . . . . T T T T T T T T T T . . . . . . . . T T T T T T T T T T T T T T T T T T T T T T . . . . . . . T T T T T T T T . . . . . . . . . T T T . T T T T    
793	. . . . . . . . . . . . . . . . . . . . . . . . . . . T T T T T T T T T T T T . T T T T T T T T T . T T T T T T T . . . . . . . . . . T T T T T T T T T T . . . . . . . . T T T T T T T T T T T T T T T T T T T T T T . . . . . . . T T T T T T T T . . . . . . . . . T T T . T T T T    
794	. . . . . . . . . . . . . . . . . . . . . . . . . . . G G G G G G G G G G G G . G G G G G G G G G . G G G G G G G . . . . . . . . . . G G G G G G G G G G . . . . . . . . G G G G G G G G G G G G G G G G G G G G G G . . . . . . . G G G G G G G G . . . . . . . . . G G G . G G G G    
795	. . . . . . . . . . . . . . . . . . . . . . . . . . . C C C C C C C C C C C C . C C C C C C C C C . C C C C C C C . . . . . . . . . . C C C C C C C C C C . . . . . . . . C C C C C C C C C C C C C C C C C C C C C C . . . . . . . C C C C C C C C . . . . . . . . . C C C . C C C C    
796	. . . . . . . . . . . . . . . . . . . . . . . . . . . T T T T T T T T T T T T . T T T T T T T T T . T T T T T T T . . . . . . . . . . T T T T T T T T T T . . . . . . . . T T T T T T T T T T T T T T T T T T T T T T . . . . . . . T T T T T T T T . . . . . . . . . T T T . T T T T    
797	. . . . . . . . . . . . . . . . . . . . . . . . . . . G G G G G G G G G G G G . G G G G G G G G G . G G G G G G G . . . . . . . . G G G G G G G G G G G G . . . . . . . . G G G G G G G G G G G G G G G G G G G G G G . . . . . . . G G G G G G G G . . . . . . . . . G G G . G G G G    
798	. . . . . . . . . . . . . . . . . . . . . . . . . . . A A A A A A A A A A A A . A A A A A A A A A . A A A A A A A . . . . . . . . A A A A A A A A A A A A . . . . . . . . A A A A A A A A A A A A A A A A A A A A A A . . . . . . . A A A A A A A A . . . . . . . . . A A A . A A A A    
799	. . . . . . . . . . . . . . . . . . . . . . . . . . . A A A A A A A A A A A A . A A A A A A A A A . A A A A A A A . . . . . . . . A A A A A A A A A A A A . . . . . . . . A A A A A A A A A A A A A A A A A A A A A A . . . . . . . A A A A A A A A . . . . . . . . . A A A . A A A A    
800	. . . . . . . . . . . . . . . . . . . . . . . . . . . T . T T T T T T T T T T . T T T T T T T T T . T T T T T T T . . . . . . . . T T T T T T T T T T T T . . . . . . . . T T T T T T T T T T T T T T T T T T T T T T . . . . . . . T T T T T T T T . . . . . . . . . T T T . T T T T    
801	. . . . . . . . . . . . . . . . . . . . . . . . . . . C . C C C C C C C C C C . C C C C C C C C C . C C C C C C C . . . . . . . . C C C C C C C C C C C C . . . . . . . . C C C C C C C C C C C C C C C C C C C C C C . . . . . . . C C C C C C C C . . . . . . . . . C C C . C C C C    
802	. . . . . . . . . . . . . . . . . . . . . . . . . . . T . T T T T T T T T T T . T T T T T T T T T . T T T T T T T . . . . . . . . T T T T T T T T T T T T . . . . . . . . T T T T T T T T T T T T T T T T T T T T T T . . . . . . . T T T T T T T T . . . . . . . . . T T T . T T T T    
803	. . . . . . . . . . . . . . . . . . . . . . . . . . . G . G G G G G G G G G G . G G G G G G G G G . G G G G G G G . . . . . . . . G G G G G G G G G G G G . . . . . . . G G G G G G G G G G G G G G G G G G G G G G G . . . . . . . G G G G G G G G . . . . . . . . . G G G . G G G G    
804	. . . . . . . . . . . . . . . . . . . . . . . . . . . C . C C C C C C C C C C . C C C C C C C C C . C C C C C C C . . . . . . . C C C C C C C C C C C C C . . . . . . . C C C C C C C C C C C C C C C C C C C C C C C . . . . . . . C C C C C C C C . . . . . . . . . C C C . C C C C    
805	. . . . . . . . . . . . . . . . . . . . . . . . . . . A . A A A A A A A A A A . A A A A A A A A A . A A A A A A A . . . . . . . A A A A A A A A A A A A A . . . . . . . A A A A A A A A A A A A A A A A A A A A A A A . . . . . . . A A A A A A A A . . . . . . . . . A A A . A A A A    
806	. . . . . . . . . . . . . . . . . . . . . . . . . . . G . G G G G G G G G G G . G G G G G G G G G G G G G G G G G . . . . . . . G G G G G G G G G G G G G . . . . . . . G G G G G G G G G G G G G G G G G G G G G G G . . . . . . . G G G G G G G G . . . . . . . . . G G G . G G G G    
807	. . . . . . . . . . . . . . . . . . . . . . . . . . . C . C C C C C C C C C C . C C C C C C C C C C C C C C C C C . . . . . . . C C C C C C C C C C C C C . . . . . . . C C C C C C C C C C C C C C C C C C C C C C C . . . . . . . C C C C C C C C . . . . . . . . . C C C . C C C C    
808	. . . . . . . . . . . . . . . . . . . . . . . . . . . . . A A A A A A A A A A . A A A A A A A A A A A A A A A A A . . . . . . . A A A A A A A A A A A A A . . . . . . . A A A A A A A A A A A A A A A A A A A A A A A . . . . . . . A A A A A A A A . . . . . . . . . A A A . A A A A    
809	. . . . . . . . . . . . . . . . . . . . . . . . . . . . . G G G G G G G G G G . G G G G G G G G G G G G G G G G G . . . . . . . G G G G G G G G G G G G G . . . . . . . G G G G G G G G G G G G G G G G G G G G G G G . . . . . . . G G G G G G G G . . . . . . . . . G G G . G G G G    
810	. . . . . . . . . . . . . . . . . . . . . . . . . . . . . A A A A A A A A A A . A A A A A A A A A A A A A A A A A . . . . . . . A A A A A A A A A A A A A . . . . . . . A A A A A A A A A A A A A A A A A A A A A A A . . . . . . . A A A A A A A A . . . . . . . . . A A A . A A A A    
811	. . . . . . . . . . . . . . . . . . . . . . . . . . . . . A A A A A A A A A A . A A A A A A A A A A A A A A A A A . . . . . . . A A A A A A A A A A A A A . . . . . . . A A A A A A A A A A A A A A A A A A A A A A A . . . . . . . A A A A A A A A . . . . . . . . . A A A . A A A A    
812	. . . . . . . . . . . . . . . . . . . . . . . . . . . . . A A A A A A A A A A . A A A A A A A A A A A A A A A A A . . . . . . . A A A A A A A A A A A A A . . . . . . . A A A A A A A A A A A A A A A A A A A A A A A . . . . . . . A A A A A A A A . . . . . . . . . A A A . A A A A    
813	. . . . . . . . . . . . . . . . . . . . . . . . . . . . . T T T T T T T T T T . T T T T T T T T T T T T C T T T T . . . . . . . T T T T T T T T T T T T T . . . . . . . T T T T T T T T T T T T T T T T T T T C T T T . . . . . . . T T T T T T T T . . . . . . . . . T T T . T T T T    
814	. . . . . . . . . . . . . . . . . . . . . . . . . . . . . G G G G G G G G G G . G G G G G G G G G G G G G G G G G . . . . . . . G G G G G G G G G G G G G . . . . . . . G G G G G G G G G G G G G G G G G G G G G G G . . . . . . . G G G G G G G G . . . . . . . . . G G G . G G G G    
815	. . . . . . . . . . . . . . . . . . . . . . . . . . . . . G G G G G G G G G G . G G G G G G G G G G G G G G G G G . . . . . . . G G G G G G G G G G G G G . . . . . . . G G G G G G G G G G G G G G G G G G G G G G G . . . . . . . G G G G G G G G . . . . . . . . . G G G . G G G G    
816	. . . . . . . . . . . . . . . . . . . . . . . . . . . . . - - - - G - - - - - G - - - - - - - - - - - - - - - - - . . . G . . . - - - - - - - - - - - - - . . G . . . . - - - - - - - - - - - - - - - - - - - - - - - . . . . . . . - - - - - - - - . . . . . . . . . - - - . - - - -    
817	. . . . . . . . . . . . . . . . . . . . . . . . . . . . . A A A A A A A A A A A A A A A A A A A A A A A A A A A A . . . A . . . A A A A A A A A A A A A A . . A . . . . A A A A A A A A A A A A A A A A A A A A A A A . . . . . . . A A A A A A A A . . . . . . . . . A A A . A A A A    
818	. . . . . . . . . . . . . . . . . . . . . . . . . . . . . T T T T T T T T T T T T T T T T T T T T T T T T T T T T . . . T . . . T T T T T T T T T T T T T . . T . . . . T T T T T T T T T T T T T T T T T T T T T T T . . . . . . . T T T T T T T T . . . . . . . . . T T T . T T T T    
819	. . . . . . . . . . . . . . . . . . . . . . . . . . . . . C C C C C C C C C C C C C C C C C C C C C C C C C C C C . . . C . . . C C C C C C C C C C C C C . . C . . . . C C C C C C C C C C C C C C C C C C C C C C C . . . . . . . C C C C C C C C . . . . . . . . . C C C . C C C C    
820	. . . . . . . . . . . . . . . . . . . . . . . . . . . . . C C C C C C C C C C C C C C C C C C C C C C C C C C C C . . . C . . . C C C C C C C C C C C C C . . C . . . . C C C C C C C C C C C C C C C C C C C C C C C . . . . . . . C C C C C C C C . . . . . . . . . C C C . C C C C    
821	. . . . . . . . . . . . . . . . . . . . . . . . . . . . . T T T T T T T T T T T T T T T T T T T T T T T T T T T T . . . T . . . T T T T T T T T T T T T T . . T . . . . T T T T T T T T T T T T T T T T T T T T T T T . . . . . . . T T T T T T T T . . . . . . . . . T T T . T T T T    
822	. . . . . . . . . . . . . . . . . . . . . . . . . . . . . A A A A A A A A A A A A A A A A A A A A A A A A A A A A . . . A . . . A A A A A A A A A A A A A . . A . . . . A A A A A A A A A A A A A A A A A A A A A A A . . . . . . . A A A A A A A A . . . . . . . . . A A A . A A A A    
823	. . . . . . . . . . . . . . . . . . . . . . . . . . . . . A A A A A A A A A A A A A A A A A A A A A A A A A A A A . . . A . . . A A A A A A A A A A A A A . . A . . . A A A A A A A A A A A A A A A A A A A A A A A A . . . . . . . A A A A A A A A . . . . . . . . . A A A . A A A A    
824	. . . . . . . . . . . . . . . . . . . . . . . . . . . . . A A A A A A A A A A A A A A A A A A A A A A A A A A A A . . . A . . . A A A A A A A A A A A A A . . A . . . A A A A A A A A A A A A A A A A A A A A A A A A . . . A . . . A A A A A A A A . . . . . . . . . A A A . A A A A    
825	. . . . . . . . . . . . . . . . . . . . . . . . . . . . . T T T T T T T T T T T T T T T T T T T T T T T T T T T T . . . T . . . T T T T T T T T T T T T T . . T . . . T T T T T T T T T T T T T T T T T T T T T T T T . . . T . . . T T T T T T T T . . . . . . . . . T T T T T T T T    
826	. . . . . . . . . . . . . . . . . . . . . . . . . . . . . G G G G G G G G G G G G G G G G G G G G G G G G G G G G . . . G . . . G G G G G G G G G G G G G . . G . . . G G G G G G G G G G G G G G G G G G G G G G G G . . G G . . G G G G G G G G G . . . . . . . . . G G G G G G G G    
827	. . . . . . . . . . . . . . . . . . . . . . . . . . . . . A A A A A A A A A A A A A A A A A A A A A A A A A A A A . . . A . . . A A A A A A A A A A A A A . . A . . . A A A A A A A A A A A A A A A A A A A A A A A A . . A A . . A A A A A A A A A . . . . . . . . . A A A A A A A A    
828	. . . . . . . . . . . . . . . . . . . . . . . . . . . . . G G G G G G G G G G G G G G G G G G G G G C G G G G G G . . . G . . . G G G G G G G G G G G G G . . G . . . G G G G G G G G G G G G G G G G G C C C G G G G . . G G . . G G G G G G G G G . . . . . . . . . G G G G G G G G    
829	. . . . . . . . . . . . . . . . . . . . . . . . . . . . . A A A A A A A A A A A A A A A A A A A A A A A A A A A A . . . A . . . A A A A A A A A A A A A A . . A . . . A A A A A A A A A A A A A A A A A A A A A A A A . . A A . . A A A A A A A A A . . . . . . . . . A A A A A A A A    
830	. . . . . . . . . . . . . . . . . . . . . . . . . . . . . T T T T T T T T T T T T T T T T T T T T T T T T T T T T . . . T . . . T T T T T T T T T T T T T . . T . . . T T T T T T T T T T T T T T T T T T T T T T T T . . T T . . T T T T T T T T T . . . . . . . . . T T T T T T T T    
831	. . . . . . . . . . . . . . . . . . . . . . . . . . . . . G G G G G G G G G G G G G G G G G G G G G G G G G G G G . . . G . . . G G G G G G G G G G G G G . . G . . . G G G G G G G G G G G G G G G G G G G G G G G G . . G G . . G G G G G G G G G . . . . . . . . . G G G G G G G G    
832	. . . . . . . . . . . . . . . . . . . . . . . . . . . . . T T T T T T T T T T T T T T T T T T T T T T T T T T T T . . . T . . . T T T T T T T T T T T T T . . T . . . T T T T T T T T T T T T T T T T T T T T T T T T . . T T . . T T T T T T T T T . . . . . . . . . T T T T T T T T    
833	. . . . . . . . . . . . . . . . . . . . . . . . . . . . . T T T T T T T T T T T T T T T T T T T T T T T T T T T T . . . T . . . T T T T T T T T T T T T T . . T . . . T T T T T T T T T T T T T T T T T T T T T T T T . . T T . . T T T T T T T T T . . . . . . . . . T T T T T T T T    
834	. . . . . . . . . . . . . . . . . . . . . . . . . . . . . C C C C C C C C C C C C C C C C C C C C C C C C C C C C . . . C . C C C C C C C C C C C C C C C . . C . . . C C C C C C C C C C C C C C C C C C C C C C C C . . C C . . C C C C C C C C C . . . . . . . . . C C C C C C C C    
835	. . . . . . . . . . . . . . . . . . . . . . . . . . . . . T T T T T T T T T T T T T T T T T T T T T T T T T T T T . . . T . T T T T T T T T T T T T T T T . . T . . . T T T T T T T T T T T T T T T T T T T T T T T T . . T T . . T T T T T T T T T . . . . . . . . . T T T T T T T T    
836	. . . . . . . . . . . . . . . . . . . . . . . . . . . . . C C C C C C C C C C C C C C C C C C C C C T C C C C C C . . . C C C C C C C C C C C C C C C C C . . C . C C C C C C C C C C C C C C C C C C C T T T C C C C . . C C . C C C C C C C C C C . . . . . . . . . C C C C C C C C    
837	. . . . . . . . . . . . . . . . . . . . . . . . . . . . . G G G G G G G G G G G G G G G G G G G G G G G G G G G G . . . G G G G G G G G G G G G G G G G G . . G . G G G G G G G G G G G G G G G G G G G G G G G G G G . . G G . G G G G G G G G G G . . . . . . . . . G G G G G G G G    
838	. . . . . . . . . . . . . . . . . . . . . . . . . . . . . C C C C C C C C C C C C C C C C C C C C C C C C C C C C . . . C C C C C C C C C C C C C C C C C . . C . C C C C C C C C C C C C C C C C C C C C C C C C C C . . C C . C C C C C C C C C C . . . . . . . . . C C C C C C C C    
839	. . . . . . . . . . . . . . . . . . . . . . . . . . . . . C C C C C C C C C C C C C C C C C C C C C C C C C C C C . . . C C C C C C C C C C C C C C C C C . . C C C C C C C C C C C C C C C C C C C C C C C C C C C C . . C C . C C C C C C C C C C . . . . . . . . . C C C C C C C C    
840	. . . . . . . . . . . . . . . . . . . . . . . . . . . . . T T T T T T T T T T T T T T T T T T T T T T T T T T T T . . . T T T T T T T T T T T T T T T T T . . T T T T T T T T T T T T T T T T T T T T T T T T T T T T . . T T . T T T T T T T T T T . . . . . . . . . T T T T T T T T    
841	. . . . . . . . . . . . . . . . . . . . . . . . . . . . . G G G G G G G G G G G G G G G G G G G G G G G G G G G G . . . G G G G G G G G G G G G G G G G G . . G G G G G G G G G G G G G G G G G G G G G G G G G G G G . . G G . G G G G G G G G G G . . . . . . . . . G G G G G G G G    
842	. . . . . . . . . . . . . . . . . . . . . . . . . . . . . T T T T T T T T T T T T T T T T T T T T T T T T T T T T . . . T T T T T T T T T T T T T T T T T . . T T T T T T T T T T T T T T T T T T T T T T T T T T T T . . T T . T T T T T T T T T T . . . . . . . . . T T T T T T T T    
843	. . . . . . . . . . . . . . . . . . . . . . . . . . . . . T T T T T T T T T T T T T T T T T T T T T T T T T T T T . . . T T T T T T T T T T T T T T T T T . . T T T T T T T T T T T T T T T T T T T T T T T T T T T T . . T T . T T T T T T T T T T . . . . . . . . . T T T T T T T T    
844	. . . . . . . . . . . . . . . . . . . . . . . . . . . . . A A A A A A A A A A A A A A A A A A A A A A A A A A A A . . . A A A A A A A A A A A A A A A A A . . A A A A A A A A A A A A A A A A A A A A A A A A A A A A . . A A . A A A A A A A A A A . . . . . . . . . A A A A A A A A    
845	. . . . . . . . . . . . . . . . . . . . . . . . . . . . . T T T T T T T T T T T T T T T T T T T T T T T T T T T T . . . T T T T T T T T T T T T T T T T T . . T T T T T T T T T T T T T T T T T T T T T T T T T T T T . . T T T T T T T T T T T T T . . . . . . . . . T T T T T T T T    
846	. . . . . . . . . . . . . . . . . . . . . . . . . . . . . G G G G G G G G G G G G G G G G G G G G G G G G G G G G . . . G G G G G G G G G G G G G G G G G . . G G G G G G G G G G G G G G G G G G G G G G G G G G G G . . G G G G G G G G G G G G G . . . . . . . . . G G G G G G G G    
847	. . . . . . . . . . . . . . . . . . . . . . . . . . . . . . A A A A A A A A A A A A A A A A A A A A A A A A A A A . . . A A A A A A A A A A A A A A A A A . . A A A A A A A A A A A A A A A A A A A A A A A A A A A A . . A A A A A A A A A A A A A . . . . . . . . . A A A A A A A A    
848	. . . . . . . . . . . . . . . . . . . . . . . . . . . . . . A A A A A A A A A A A A A A A A A A A A A A A A A A A . . . A A A A A A A A A A A A A A A A A . . A A A A A A A A A A A A A A A A A A A A A A A A A A A A . . A A A A A A A A A A A A A . . . . . . . . . A A A A A A A A    
849	. . . . . . . . . . . . . . . . . . . . . . . . . . . . . . T T T T T T T T T T T T T T T T T T T T T T T T T T T . . . T T T T T T T T T T T T T T T T T . . T T T T T T T T T T T T T T T T T T T T T T T T T T T T . . T T T T T T T T T T T T T . . . . . . . . . T T T T T T T T    
850	. . . . . . . . . . . . . . . . . . . . . . . . . . . . . . C C C C C C C C C C C C C C C C C C C C C C C C C C C . . . C C C C C C C C C C C C C C C C C . . C C C C C C C C C C C C C C C C C C C C C C C C C C C C . . C C C C C C C C C C C C C . . . . . . . . . C C C C C C C C    
851	. . . . . . . . . . . . . . . . . . . . . . . . . . . . . . A A A A A A A A A A A A A A A A A A A A A A A A A A A . . . A A A A A A A A A A A A A A A A A . . A A A A A A A A A A A A A A A A A A A A A A A A A A A A . . A A A A A A A A A A A A A . . . . . . . . . A A A A A A A A    
852	. . . . . . . . . . . . . . . . . . . . . . . . . . . . . . G G G G G G G G G G G G G G G G G G G G G G G G G G G . . . G G G G G G G G G G G G G G G G G . . G G G G G G G G G G G G G G G G G G G G G G G G G G G G . . G G G G G G G G G G G G G . . . . . . . . . G G G G G G G G    
853	. . . . . . . . . . . . . . . . . . . . . . . . . . . . . . T T T T T T T T T T T T T T T T T T T T T T T T T T T . . . T T T T T T T T T T T T T T T T T . . T T T T T T T T T T T T T T T T T T T T T T T T T T T T . . T T T T T T T T T T T T T . . . . . . . . . T T T T T T T T    
854	. . . . . . . . . . . . . . . . . . . . . . . . . . . . . . G G G G G G G G G G G G G G G G G G G G G G G G G G G . . . G G G G G G G G G G G G G G G G G . . G G G G G G G G G G G G G G G G G G G G G G G G G G G G . . G G G G G G G G G G G G G . . . . . . . . . G G G G G G G G    
855	. . . . . . . . . . . . . . . . . . . . . . . . . . . . . . T T T T T T T T T T T T T T T T T T T T T T T T T T T . . . T T T T T T T T T T T T T T T T T . . T T T T T T T T T T T T T T T T T T T T T T T T T T T T . . T T T T T T T T T T T T T . . . . . . . . T T T T T T T T T    
856	. . . . . . . . . . . . . . . . . . . . . . . . . . . . . . T T T T T T T T T T T T T T T T T T T T T T T T T T T . . . T T T T T T T T T T T T T T T T T . . T T T T T T T T T T T T T T T T T T T T T T T T T T T T . . T T T T T T T T T T T T T . . . . . . . . T T T T T T T T T    
857	. . . . . . . . . . . . . . . . . . . . . . . . . . . . . . C C C C C C C C C C C C C C C C C C C C C C C C C C C . . . C C C C C C C C C C C C C C C C C . . C C C C C C C C C C C C C C C C C C C C C C C C C C C C . . C C C C C C C C C C C C C . . . . . . . . C C C C C C C C C    
858	. . . . . . . . . . . . . . . . . . . . . . . . . . . . . . C C C C C C C C C C C C C C C C C C C C C C C C C C C . . . C C C C C C C C C C C C C C C C C . . C C C C C C C C C C C C C C C C C C C C C C C C C C C C . . C C C C C C C C C C C C C . . . . . . . . C C C C C C C C C    
859	. . . . . . . . . . . . . . . . . . . . . . . . . . . . . . T T T T T T T T T T T T T T T T T T T T T T T T T T T . . . T T T T T T T T T T T T T T T T T . . T T T T T T T T T T T T T T T T T T T T T T T T T T T T . . T T T T T T T T T T T T T . . . . . . . . T T T T T T T T T    
860	. . . . . . . . . . . . . . . . . . . . . . . . . . . . . . G G G G G G G G G G G G G G G G G G G G G G G G G G G . . . G G G G G G G G G G G G G G G G G . . G G G G G G G G G G G G G G G G G G G G G G G G G G G G . . G G G G G G G G G G G G G . . . . . . . . G G G G G G G G G    
861	. . . . . . . . . . . . . . . . . . . . . . . . . . . . . . A A A A A A A A A A A A A A A A A A A A A A A A A A A . . . A A A A A A A A A A A A A A A A A . . A A A A A A A A A A A A A A A A A A A A A A A A A A A A . . A A A A A A A A A A A A A . . . . . . . . A A A A A A A A A    
862	. . . . . . . . . . . . . . . . . . . . . . . . . . . . . . T T T T T T T T T T T T T T T T T T T T T T T T T T T . . . T T T T T T T T T T T T T T T T T . . T T T T T T T T T T T T T T T T T T T T T T T T T T T T . . T T T T T T T T T T T T T . . . . . . . . T T T T T T T T T    
863	. . . . . . . . . . . . . . . . . . . . . . . . . . . . . . T T T T T T T T T T T T T T T T T T T T T T T T T T T . . . T T T T T T T T T T T T T T T T T . . T T T T T T T T T T T T T T T T T T T T T T T T T T T T . . T T T T T T T T T T T T T . . . . . . . . T T T T T T T T T    
864	. . . . . . . . . . . . . . . . . . . . . . . . . . . . . . G G G G G G G G G G G G G G G G G G G G G G G G G G G . . . G G G G G G G G G G G G G G G G G . . G G G G G G G G G G G G G G G G G G G G G G G G G G G G . . G G G G G G G G G G G G G . . . . . . . . G G G G G G G G G    
865	. . . . . . . . . . . . . . . . . . . . . . . . . . . . . . G G G G G G G G G G G G G G G G G G G G G G G G G G G . . . G G G G G G G G G G G G G G G G G . . G G G G G G G G G G G G G G G G G G G G G G G G G G G G . . G G G G G G G G G G G G G . . . . . . . . G G G G G G G G G    
866	. . . . . . . . . . . . . . . . . . . . . . . . . . . . . . A A A A A A A A A A A A A A A A A A A A A A A A A A A . . . A A A A A A A A A A A A A A A A A . . A A A A A A A A A A A A A A A A A A A A A A A A A A A A . . A A A A A A A A A A A A A . . . . . . . . A A A A A A A A A    
867	. . . . . . . . . . . . . . . . . . . . . . . . . . . . . . C C C C C C C C C C C C C C C C C C C C C C C C C C C . . . C C C C C C C C C C C C C C C C C . . C C C C C C C C C C C C C C C C C C C C C C C C C C C C . . C C C C C C C C C C C C C . . . . . . . . C C C C C C C C C    
868	. . . . . . . . . . . . . . . . . . . . . . . . . . . . . . G G G G G G G G G G G G G G G G G G G G G G G G G G G . . . G G G G G G G G G G G G G G G G G . . G G G G G G G G G G G G G G G G G G G G G G G G G G G G . . G G G G G G G G G G G G G . . . . . . . . G G G G G G G G G    
869	. . . . . . . . . . . . . . . . . . . . . . . . . . . . . . T T T T T T T T T T T T T T T T T T T T T T T T T T T . . . T T T T T T T T T T T T T T T T T . . T T T T T T T T T T T T T T T T T T T T T T T T T T T T . . T T T T T T T T T T T T T . . . . . . . . T T T T T T T T T    
870	. . . . . . . . . . . . . . . . . . . . . . . . . . . . . . T T T T T T T T T T T T T T T T T T T T T T T T T T T . . . T T T T T T T T T T T T T T T T T . . T T T T T T T T T T T T T T T T T T T T T T T T T T T T . . T T T T T T T T T T T T T . . . . . . . . T T T T T T T T T    
871	. . . . . . . . . . . . . . . . . . . . . . . . . . . . . . A A A A A A A A A A A A A A A A A A A A A A A A A A A . . A A A A A A A A A A A A A A A A A A . . A A A A A A A A A A A A A A A A A A A A A A A A A A A A . . A A A A A A A A A A A A A . . . . . . . . A A A A A A A A A    
872	. . . . . . . . . . . . . . . . . . . . . . . . . . . . . . C C C C C C C C C C C C C C C C C C C C C C C C C C C . . C C C C C C C C C C C C C C C C C C . . C C C C C C C C C C C C C C C C C C C C C C C C C C C C . . C C C C C C C C C C C C C . . . . . . . . C C C C C C C C C    
873	. . . . . . . . . . . . . . . . . . . . . . . . . . . . . . T T T T T T T T T T T T T T T T T T T T T T T T T T T . . T T T T T T T T T T T T T T T T T T . . T T T T T T T T T T T T T T T T T T T T T T T T T T T T . . T T T T T T T T T T T T T . . . . . . . . T T T T T T T T T    
874	. . . . . . . . . . . . . . . . . . . . . . . . . . . . . . G G G G G G G G G G G G G G G G G G G G G G G G G G G . . G G G G G G G G G G G G G G G G G G . . G G G G G G G G G G G G G G G G G G G G G G G G G G G G . . G G G G G G G G G G G G G . . . . . . . . G G G G G G G G G    
875	. . . . . . . . . . . . . . . . . . . . . . . . . . . . . . C C C C C C C C C C C C C C C C C C C C C C C C C C C . . C C C C C C C C C C C C C C C C C C . . C C C C C C C C C C C C C C C C C C C C C C C C C C C C . . C C C C C C C C C C C C C . . . . . . . . C C C C C C C C C    
876	. . . . . . . . . . . . . . . . . . . . . . . . . . . . . . T T T T T T T T T T T T T T T T T T T T T T T T T T T . . T T T T T T T T T T T T T T T T T T . . T T T T T T T T T T T T T T T T T T T T T T T T T T T T . . T T T T T T T T T T T T T . . . . . . . . T T T T T T T T T    
877	. . . . . . . . . . . . . . . . . . . . . . . . . . . . . . G G G G G G G G G G G G G G G G G G G G G G G G G G G . . G G G G G G G G G G G G G G G G G G . . G G G G G G G G G G G G G G G G G G G G G G G G G G G G . . G G G G G G G G G G G G G . . . . . . . . G G G G G G G G G    
878	. . . . . . . . . . . . . . . . . . . . . . . . . . . . . . G G G G G G G G G G G G G G G G G G G G G G G G G G G . . G G G G G G G G G G G G G G G G G G . . G G G G G G G G G G G G G G G G G G G G G G G G G G G G . . G G G G G G G G G G G G G . . . . . . . . G G G G G G G G G    
879	. . . . . . . . . . . . . . . . . . . . . . . . . . . . . . A A A A A A A A A A A A A A A A A A A A A A A A A A A . . A A A A A A A A A A A A A A A A A A . . A A A A A A A A A A A A A A A A A A A A A A A A A A A A . . A A A A A A A A A A A A A . . . . . . . . A A A A A A A A A    
880	. . . . . . . . . . . . . . . . . . . . . . . . . . . . . . C C C C C C C C C C C C C C C C C C C C C C C C C C C . . C C C C C C C C C C C C C C C C C C . . C C C C C C C C C C C C C C C C C C C C C C C C C C C C . . C C C C C C C C C C C C C . . . . . . . . C C C C C C C C C    
881	. . . . . . . . . . . . . . . . . . . . . . . . . . . . . . T T T T T T T T T T T T T T T T T T T T T T T T T T T . . T T T T T T T T T T T T T T T T T T . . T T T T T T T T T T T T T T T T T T T T T T T T T T T T . . T T T T T T T T T T T T T . . . . . . . . T T T T T T T T T    
882	. . . . . . . . . . . . . . . . . . . . . . . . . . . . . . G G G G G G G G G G G G G G G G G G G G G G G G G G G . . G G G G G G G G G G G G G G G G G G . . G G G G G G G G G G G G G G G G G G G G G G G G G G G G . . G G G G G G G G G G G G G . . . . . . . . G G G G G G G G G    
883	. . . . . . . . . . . . . . . . . . . . . . . . . . . . . . T T T T T T T T T T T T T T T T T T T T T T T T T T T . . T T T T T T T T T T T T T T T T T T . . T T T T T T T T T T T T T T T T T T T T T T T T T T T T . . T T T T T T T T T T T T T . . . . . . . . T T T T T T T T T    
884	. . . . . . . . . . . . . . . . . . . . . . . . . . . . . . T T T T T T T T T T T T T T T T T T T T T T T T T T T . . T T T T T T T T T T T T T T T T T T . . T T T T T T T T T T T T T T T T T T T T T T T T T T T T . . T T T T T T T T T T T T T . . . . . . . . T T T T T T T T T    
885	. . . . . . . . . . . . . . . . . . . . . . . . . . . . . . T T T T T T T T T T T T T T T T T T T T T T T T T T T . . T T A T T T T T T T T T T T T T T T . . T T T T T T T T T T T T T T T T T T T T T T T T T T T T . . T T T T T T T T T T T T T . . . . . . . . T T T T T T T T T    
886	. . . . . . . . . . . . . . . . . . . . . . . . . . . . . . A A A A A A A A A A A A A A A A A A A A A A A A A A A . . A A A A A A A A A A A A A A A A A A . . A A A A A A A A A A A A A A A A A A A A A A A A A A A A . . A A A A A A A A A A A A A . . . . . . . . A A A A A A A A A    
887	. . . . . . . . . . . . . . . . . . . . . . . . . . . . . . A A A A A A A A A A A A A A A A A A A A A A A A A A A . . A A A A A A A A A A A A A A A A A A . . A A A A A A A A A A A A A A A A A A A A A A A A A A A A . . A A A A A A A A A A A A A . . . . . . . . A A A A A A A A A    
888	. . . . . . . . . . . . . . . . . . . . . . . . . . . . . . C C C C C C C C C C C C C C C C C C C C C C C C C C C . . C C C C C C C C C C C C C C C C C C . . C C C C C C C C C C C C C C C C C C C C C C C C C C C C . . C C C C C C C C C C C C C . . . . . . . . C C C C C C C C C    
889	. . . . . . . . . . . . . . . . . . . . . . . . . . . . . . T T T T T T T T T T T T T T T T T T T T T T T T T T T . . T T T T T T T T T T T T T T T T T T . . T T T T T T T T T T T T T T T T T T T T T T T T T T T T . . T T T T T T T T T T T T T . . . . . . . . T T T T T T T T T    
890	. . . . . . . . . . . . . . . . . . . . . . . . . . . . . . G G G G G G G G G G G G G G G G G G G G G G G G G G G . . G G G G G G G G G G G G G G G G G G . . G G G G G G G G G G G G G G G G G G G G G G G G G G G G . . G G G G G G G G G G G G G . . . . . . . . G G G G G G G G G    
891	. . . . . . . . . . . . . . . . . . . . . . . . . . . . . . G G G G G G G G G G G G G G G G G G G G G G G G G G G . . G G G G G G G G G G G G G G G G G G . . G G G G G G G G G G G G G G G G G G G G G G G G G G G G . . G G G G G G G G G G G G G . . . . . . . . G G G G G G G G G    
892	. . . . . . . . . . . . . . . . . . . . . . . . . . . . . . - - - G - - - - - - - - - - - - - - - - - - - - - - - . . - - - - - - - - - - - - - - - - - - . . - - - - - - - - - - - - - - - - - - - - - - - - - - - - . . - - - - - - - - - - - - - . . . . . . . . - - - - - - - - -    
893	. . . . . . . . . . . . . . . . . . . . . . . . . . . . . . A A A A A A A A A A A A A A A A A A A A A A A A A A A . . A A A A A A A A A A A A A A A A A A . . A A A A A A A A A A A A A A A A A A A A A A A A A A A A . . A A A A A A A A A A A A A . . . . . . . . A A A A A A A A A    
894	. . . . . . . . . . . . . . . . . . . . . . . . . . . . . . C C C C C C C C C C C C C C C C C C C C C C C C C C C . . C C C C C C C C C C C C C C C C C C . . C C C C C C C C C C C C C C C C C C C C C C C C C C C C . . C C C C C C C C C C C C C . . . . . . . C C C C C C C C C C    
895	. . . . . . . . . . . . . . . . . . . . . . . . . . . . . . A A A A A A A A A A A A A A A A A A A A G A A A A A A . . A A A A A A A A A A A A A A A A A A . . A A A A A A A A A A A A A A A A A A A A A G G G A A A A . . A A G A A A A A A A A A A . . . . . . . A A A A A A A A A A    
896	. . . . . . . . . . . . . . . . . . . . . . . . . . . . . . T T T T T T T T T T T T T T T T T T T T T T T T T T T . . T T T T T T T T T T T T T T T T T T . . T T T T T T T T T T T T T T T T T T T T T T T T T T T T . . T T T T T T T T T T T T T . . . . . . . T T T T T T T T T T    
897	. . . . . . . . . . . . . . . . . . . . . . . . . . . . . . G G G G G G G G G G G G G G G G G G G G G G G G G G G . . G G G G G G G G G G G G G G G G G G . . G G G G G G G G G G G G G G G G G G G G G G G G G G G G . . G G G G G G G G G G G G G . . . . . . . G G G G G G G G G G    
898	. . . . . . . . . . . . . . . . . . . . . . . . . . . . . . A A A A A A A A A A A A A A A A A A A A A A A A A A A . . A A A A A A A A A A A A A A A A A A . . A A A A A A A A A A A A A A A A A A A A A A A A A A A A . . A A A A A A A A A A A A A . . . . . . . A A A A A A A A A A    
899	. . . . . . . . . . . . . . . . . . . . . . . . . . . . . . A A A A A A A A A A A A A A A A A A A A A A A A A A A . . A A A A A A A A A A A A A A A A A A . . A A A A A A A A A A A A A A A A A A A A A A A A A A A A . . A A A A A A A A A A A A A . . . . . . . A A A A A A A A A A    
900	. . . . . . . . . . . . . . . . . . . . . . . . . . . . . . T T T T T T T T T T T T T T T T T T T T T T T T T T T . . T T T T T T T T T T T T T T T T T T . . T T T T T T T T T T T T T T T T T T T T T T T T T T T T . . T T T T T T T T T T T T T . . . . . . . T T T T T T T T T T    
901	. . . . . . . . . . . . . . . . . . . . . . . . . . . . . . C C C C C C C C C C C C C C C C C C C C C C C C C C C . . C C C C C C C C C C C C C C C C C C . . C C C C C C C C C C C C C C C C C C C C C C C C C C C C . . C C C C C C C C C C C C C . . . . . . . C C C C C C C C C C    
902	. . . . . . . . . . . . . . . . . . . . . . . . . . . . . . A A A A A A A A A A A A A A A A A A A A A A A A A A A . . A A A A A A A A A A A A A A A A A A . . A A A A A A A A A A A A A A A A A A A A A A A A A A A A . . A A A A A A A A A A A A A . . . . . . . A A A A A A A A A A    
903	. . . . . . . . . . . . . . . . . . . . . . . . . . . . . . A A A A A A A A A A A A A A A A A A A A A A A A A A A . . A A A A A A A A A A A A A A A A A A . . A A A A A A A A A A A A A A A A A A A A A A A A A A A A . . A A A A A A A A A A A A A . . . . . . . A A A A A A A A A A    
904	. . . . . . . . . . . . . . . . . . . . . . . . . . . . . . T T T T T T T T T T T T T T T T T T T T T T T T T T T . . T T T T T T T T T T T T T T T T T T . . T T T T T T T T T T T T T T T T T T T T T T T T T T T T . . T T T T T T T T T T T T T . . . . . . . T T T T T T T T T T    
905	. . . . . . . . . . . . . . . . . . . . . . . . . . . . . . A A A A A A A A A A A A A A A A A A A A A A A A A A A . . A A A A A A A A A A A A A A A A A A . . A A A A A A A A A A A A A A A A A A A A A A A A A A A A . . A A A A A A A A A A A A A . . . . . . . A A A A A A A A A A    
906	. . . . . . . . . . . . . . . . . . . . . . . . . . . . . . C C C C C C C C C C C C C C C C C C C C C C C C C C C . . C C C C C C C C C C C C C C C C C C . . C C C C C C C C C C C C C C C C C C C C C C C C C C C C . . C C C C C C C C C C C C C . . . . . . . C C C C C C C C C C    
907	. . . . . . . . . . . . . . . . . . . . . . . . . . . . . . T T T T T T T T T T T T T T T T T T T T T T T T T T T . . T T T T T T T T T T T T T T T T T T . . T T T T T T T T T T T T T T T T T T T T T T T T T T T T . . T T T T T T T T T T T T T . . . . . . . T T T T T T T T T T    
908	. . . . . . . . . . . . . . . . . . . . . . . . . . . . . . T T T T T T T T T T T T T T T T T T T T T T T T T T T . . T T T T T T T T T T T T T T T T T T . . T T T T T T T T T T T T T T T T T T T T T T T T T T T T . . T T T T T T T T T T T T T . . . . . . . T T T T T T T T T T    
909	. . . . . . . . . . . . . . . . . . . . . . . . . . . . . . T T T T T T T T T T T T T T T T T T T T T T T T T T T . . T T T T T T T T T T T T T T T T T T . . T T T T T T T T T T T T T T T T T T T T T T T T T T T T . . T T T T T T T T T T T T T . . . . . . . T T T T T T T T T T    
910	. . . . . . . . . . . . . . . . . . . . . . . . . . . . . . C C C C C C C C C C C C C C C C C C C T C C C C C C C . . C C C C C C C C C C C C C C C C C C . . C C C C C C C C C C C C C C C C C C C C C C C C C C C C . . C C C C C C C C C C C C C . . . . . . . C C C C C C C C C C    
911	. . . . . . . . . . . . . . . . . . . . . . . . . . . . . . T T T T T T T T T T T T T T T T T T T T T T T T T T T . T T T T T T T T T T T T T T T T T T T . . T T T T T T T T T T T T T T T T T T T T T T T T T T T T . . T T T T T T T T T T T T T . . . . . . . T T T T T T T T T T    
912	. . . . . . . . . . . . . . . . . . . . . . . . . . . . . . T T T T T T T T T T T T T T T T T T T T T T T T T T T . T T T T T T T T T T T T T T T T T T T . . T T T T T T T T T T T T T T T T T T T T T T T T T T T T T T T T T T T T T T T T T T T . . . . . . . T T T T T T T T T T    
913	. . . . . . . . . . . . . . . . . . . . . . . . . . . . . . A A A A A A A A A A A A A A A A A A A A A A A A A A A . A A A A A A A A A A A A A A A A A A A . . A A A A A A A A A A A A A A A A A A A A A A A A A A A A A A A A A A A A A A A A A A A . . . . . . . A A A A A A A A A A    
914	. . . . . . . . . . . . . . . . . . . . . . . . . . . . . . A A A A A A A A A A A A A A A A A A A A A A A A A A A . A A A A A A A A A A A A A A A A A A A . . A A A A A A A A A A A A A A A A A A A A A A A A A A A A A A A A A A A A A A A A A A A . . . . . . . A A A A A A A A A A    
915	. . . . . . . . . . . . . . . . . . . . . . . . . . . . . . T T T T T T T T T T T T T T T T T T T T T T T T T T T . T T T T T T T T T T T T T T T T T T T . . T T T T T T T T T T T T T T T T T T T T T T T T T T T T T T T T T T T T T T T T T T T . . . . . . . T T T T T T T T T T    
916	. . . . . . . . . . . . . . . . . . . . . . . . . . . . . . G G G G G G G G G G G G G G G G G G G G G G G G G G G . G G G G G G G G G G G G G G G G G G G . . G G G G G G G G G G G G G G G G G G G G G G G G G G G G G G G G G G G G G G G G G G G . . . . . . . G G G G G G G G G G    
917	. . . . . . . . . . . . . . . . . . . . . . . . . . . . . . A A A A A A A A A A A A A A A A A A A A A A A A A A A . A A A A A A A A A A A A A A A A A A A . . A A A A A A A A A A A A A A A A A A A A A A A A A A A A A A A A A A A A A A A A A A A . . . . . . . A A A A A A A A A A    
918	. . . . . . . . . . . . . . . . . . . . . . . . . . . . . . A A A A A A A A A A A A A A A A A A A A A A A A A A A . A A A A A A A A A A A A A A A A A A A . . A A A A A A A A A A A A A A A A A A A A A A A A A A A A A A A A A A A A A A A A A A A . . . . . . . A A A A A A A A A A    
919	. . . . . . . . . . . . . . . . . . . . . . . . . . . . . . G G G G G G G G G G G G G G G G G G G G G G G G G G G . G G G G G G G G G G G G G G G G G G G . . G G G G G G G G G G G G G G G G G G G G G G G G G G G G G G G G G G G G G G G G G G G . . . . . . . G G G G G G G G G G    
920	. . . . . . . . . . . . . . . . . . . . . . . . . . . . . . C C C C C C C C C C C C C C C C C C C C C C C C C C C . C C C C C C C C C C C C C C C C C C C . . C C C C C C C C C C C C C C C C C C C C C C C C C C C C C C C C C C C C C C C C C C C . . . . . . . C C C C C C C C C C    
921	. . . . . . . . . . . . . . . . . . . . . . . . . . . . . . A A A A A A A A A A A A A A A A A A A A A A A A A A A . A A A A A A A A A A A A A A A A A A A . . A A A A A A A A A A A A A A A A A A A A A A A A A A A A A A A A A A A A A A A A A A A . . . . . . . A A A A A A A A A A    
922	. . . . . . . . . . . . . . . . . . . . . . . . . . . . . . T T T T T T T T T T T T T T T T T T T T T T T T T T T . T T T T T T T T T T T T T T T T T T T . . T T T T T T T T T T T T T T T T T T T T T T T T T T T T T T T T T T T T T T T T T T T . . . . . . . T T T T T T T T T T    
923	. . . . . . . . . . . . . . . . . . . . . . . . . . . . . . A A A A A A A A A A A A A A A A A A A A A A A A A A A . A A A A A A A A A A A A A A A A A A A . . A A A A A A A A A A A A A A A A A A A A A A A A A A A A A A A A A A A A A A A A A A A . . . . . . . A A A A A A A A A A    
924	. . . . . . . . . . . . . . . . . . . . . . . . . . . . . . T T T T T T T T T T T T T T T T T T T T T T T T T T T . T T T T T T T T T T T T T T T T T T T . . T T T T T T T T T T T T T T T T T T T T T T T T T T T T T T T T T T T T T T T T T T T . . . . . . . T T T T T T T T T T    
925	. . . . . . . . . . . . . . . . . . . . . . . . . . . . . . T T T T T T T T T T T T T T T T T T T T T T T T T T T . T T T T T T T T T T T T T T T T T T T . . T T T T T T T T T T T T T T T T T T T T T T T T T T T T T T T T T T T T T T T T T T T . . . . . . . T T T T T T T T T T    
926	. . . . . . . . . . . . . . . . . . . . . . . . . . . . . . G G G G G G G G G G G G G G G G G G G G G G G G G G G . G G G G G G G G G G G G G G G G G G G . . G G G G G G G G G G G G G G G G G G G G G G G G G G G G G G G G G G G G G G G G G G G . . . . . . . G G G G G G G G G G    
927	. . . . . . . . . . . . . . . . . . . . . . . . . . . . . . . T T T T T T T T T T T T T T T T T T T T T T T T T T . T T T T T T T T T T T T T T T T T T T . . T T T T T T T T T T T T T T T T T T T T T T T T T T T T T T T T T T T T T T T T T T T . . . . . . . T T T T T T T T T T    
928	. . . . . . . . . . . . . . . . . . . . . . . . . . . . . . . T T T T T T T T T T T T T T T T T T T T T T T T T T . T T T T T T T T T T T T T T T T T T T . . T T T T T T T T T T T T T T T T T T T T T T T T T T T T T T T T T T T T T T T T T T T . . . . . . . T T T T T T T T T T    
929	. . . . . . . . . . . . . . . . . . . . . . . . . . . . . . . G G G G G G G G G G G G G G G G G G G G G G G G G G . G G G G G G G G G G G G G G G G G G G . . G G G G G G G G G G G G G G G G G G G G G G G G G G G G G G G G G G G G G G G G G G G . . . . . . . G G G G G G G G G G    
930	. . . . . . . . . . . . . . . . . . . . . . . . . . . . . . . G G G G G G G G G G G G G G G G G G G G G G G G G G . G G G G G G G G G G G G G G G G G G G . . G G G G G G G G G G G G G G G G G G G G G G G G G G G G G G G G G G G G G G G G G G G . . . . . . . G G G G G G G G G G    
931	. . . . . . . . . . . . . . . . . . . . . . . . . . . . . . . T T T T T T T T T T T T T T T T T T T T T T T T T T . T T T T T T T T T T T T T T T T T T T . . T T T T T T T T T T T T T T T T T T T T T T T T T T T T T T T T T T T T T T T T T T T . . . . . . . T T T T T T T T T T    
932	. . . . . . . . . . . . . . . . . . . . . . . . . . . . . . . A A A A A A A A A A A A A A A A A A A A A A A A A A . A A A A A A A A A A A A A A A A A A A . . A A A A A A A A A A A A A A A A A A A A A A A A A A A A A A A A A A A A A A A A A A A . . . . . . . A A A A A A A A A A    
933	. . . . . . . . . . . . . . . . . . . . . . . . . . . . . . . A A A A A A A A A A A A A A A A A A A A A A A A A A . A A A A A A A A A A A A A A A A A A A . . A A A A A A A A A A A A A A A A A A A A A A A A A A A A A A A A A A A A A A A A A A A . . . . . . . A A A A A A A A A A    
934	. . . . . . . . . . . . . . . . . . . . . . . . . . . . . . . C C C C C C C C C C C C C C C C C C C C C C C C C C . C C C C C C C C C C C C C C C C C C C . . C C C C C C C C C C C C C C C C C C C C C C C C C C C C C C C C C C C C C C C C C C C . . . . . . . C C C C C C C C C C    
935	. . . . . . . . . . . . . . . . . . . . . . . . . . . . . . . A A A A A A A A A A A A A A A A A A A A A A A A A A . A A A A A A A A A A A A A A A A A A A . . A A A A A A A A A A A A A A A A A A A A A A A A A A A A A A A A A A A A A A A A A A A . . . . . . . A A A A A A A A A A    
936	. . . . . . . . . . . . . . . . . . . . . . . . . . . . . . . T T T T T T T T T T T T T T T T T T T T T T T T T T . T T T T T T T T T T T T T T T T T T T . . T T T T T T T T T T T T T T T T T T T T T T T T T T T T T T T T T T T T T T T T T T T . . . . . . . T T T T T T T T T T    
937	. . . . . . . . . . . . . . . . . . . . . . . . . . . . . . . A A A A A A A A A A A A A A A A A A A A A A A A A A . A A A A A A A A A A A A A A A A A A A . . A A A A A A A A A A A A A A A A A A A A A A A A A A A A A A A A A A A A A A A A A A A . . . . . . . A A A A A A A A A A    
938	. . . . . . . . . . . . . . . . . . . . . . . . . . . . . . . T T T T T T T T T T T T T T T T T T T T T T T T T T . T T T T T T T T T T T T T T T T T T T . . T T T T T T T T T T T T T T T T T T T T T T T T T T T T T T T T T T T T T T T T T T T . . . . . . . T T T T T T T T T T    
939	. . . . . . . . . . . . . . . . . . . . . . . . . . . . . . . A A A A A A A A A A A A A A A A A A A A A A A A A A . A A A A A A A A A A A A A A A A A A A . . A A A A A A A A A A A A A A A A A A A A A A A A A A A A A A A A A A A A A A A A A A A . . . . . . . A A A A A A A A A A    
940	. . . . . . . . . . . . . . . . . . . . . . . . . . . . . . . T T T T T T T T T T T T T T T T T T T T T T T T T T . T T T T T T T T T T T T T T T T T T T . . T T T T T T T T T T T T T T T T T T T T T T T T T T T T T T T T T T T T T T T T T T T . . . . . . . T T T T T T T T T T    
941	. . . . . . . . . . . . . . . . . . . . . . . . . . . . . . . T T T T T T T T T T T T T T T T T T T T T T T T T T . T T T T T T T T T T T T T T T T T T T . . T T T T T T T T T T T T T T T T T T T T T T T T T T C T T T T T T T T T T T T T T T T . . . . . . . T T T T T T T T T T    
942	. . . . . . . . . . . . . . . . . . . . . . . . . . . . . . . A A A A A A A A A A A A A A A A A A A A A A A A A A . A A A A A A A A A A A A A A A A A A A . . A A A A A A A A A A A A A A A A A A A A A A A A A A A A A A A A A A A A A A A A A A A . . . . . . . A A A A A A A A A A    
943	. . . . . . . . . . . . . . . . . . . . . . . . . . . . . . . T T T T T T T T T T T T T T T T T T T T T T T T T T . T T T T T T T T T T T T T T T T T T T . . T T T T T T T T T T T T T T T T T T T T T T T T T T T T T T T T T T T T T T T T T T T . . . . . . . T T T T T T T T T T    
944	. . . . . . . . . . . . . . . . . . . . . . . . . . . . . . . C C C C C C C C C C C C C C C C C C C C C C C C C C . C C C C C C C C C C C C C C C C C C C . . C C C C C C C C C C C C C C C C C C C C C C C C C C C C C C C C C C C C C C C C C C C . . . . . . . C C C C C C C C C C    
945	. . . . . . . . . . . . . . . . . . . . . . . . . . . . . . . C C C C C C C C C C C C C C C C C C C C C C C C C C . C C C C C C C C C C C C C C C C C C C . . C C C C C C C C C C C C C C C C C C C C C C C C C C C C C C C C C C C C C C C C C C C . . . . . . . C C C C C C C C C C    
946	. . . . . . . . . . . . . . . . . . . . . . . . . . . . . . . T T T T T T T T T T T T T T T T T T T T T T T T T T . T T T T T T T T T T T T T T T T T T T . . T T T T T T T T T T T T T T T T T T T T T T T T T T T T T T T T T T T T T T T T T T T . . . . . . . T T T T T T T T T T    
947	. . . . . . . . . . . . . . . . . . . . . . . . . . . . . . . T T T T T T T T T T T T T T T T T T T T T T T T T T . T T T T T T T T T T T T T T T T T T T . . T T T T T T T T T T T T T T T T T T T T T T T T T T T T T T T T T T T T T T T T T T T . . . . . . . T T T T T T T T T T    
948	. . . . . . . . . . . . . . . . . . . . . . . . . . . . . . . T T T T T T T T T T T T T T T T T T T C T T T T T T . T T T T T T T T T T T T T T T T T T T . . T T T T T T T T T T T T T T T T T T T T C C C C T T T T T T T T C T T T T T T T T T T . . . . . . . T T T T T C C T T T  8	 transition  
949	. . . . . . . . . . . . . . . . . . . . . . . . . . . . . . . C C C C C C C C C C C C C C C C C C C C C C C C C C . C C C C C C C C C C C C C C C C C C C . . C C C C C C C C C C C C C C C C C C C C C C C C C C C C C C C C C C C C C C C C C C C . . . . . . . C C C C C C C C C C    
950	. . . . . . . . . . . . . . . . . . . . . . . . . . . . . . . A A A A A A A A A A A A A A A A A A A A A A A A A A . A A A A A A A A A A A A A A A A A A A . . A A A A A A A A A A A A A A A A A A A A A A A A A A A A A A A A A A A A A A A A A A A . . . . . . . A A A A A A A A A A    
951	. . . . . . . . . . . . . . . . . . . . . . . . . . . . . . . C C C C C C C C C C C C C C C C C C C C C C C C C C . C C C C C C C C C C C C C C C C C C C . . C C C C C C C C C C C C C C C C C C C C C C C C C C C C C C C C C C C C C C C C C C C . . . . . . . C C C C C C C C C C    
952	. . . . . . . . . . . . . . . . . . . . . . . . . . . . . . . C C C C C C C C C C C C C C C C C C C C C C C C C C . C C C C C C C C C C C C C C C C C C C . . C C C C C C C C C C C C C C C C C C C C C C C C C C C C C C C C C C C C C C C C C C C . . . . . . . C C C C C C C C C C    
953	. . . . . . . . . . . . . . . . . . . . . . . . . . . . . . . T T T T T T T T T T T T T T T T T T T T T T T T T T . T T T T T T T T T T T T T T T T T T T . . T T T T T T T T T T T T T T T T T T T T T T T T T T T T T T T T T T T T T T T T T T T . . . . . . . T T T T T T T T T T    
954	. . . . . . . . . . . . . . . . . . . . . . . . . . . . . . . T T T T T T T T T T T T T T T T T T T T T T T T T T . T T T T T T T T T T T T T T T T T T T . . T T T T T T T T T T T T T T T T T T T T T T T T T T T T T T T T T T T T T T T T T T T . . . . . . . T T T T T T T T T T    
955	. . . . . . . . . . . . . . . . . . . . . . . . . . . . . . . T T T T T T T T T T T T T T T T T T T T T T T T T T . T T T T T T T T T T T T T T T T T T T . . T T T T T T T T T T T T T T T T T T T T T T T T T T T T T T T T T T T T T T T T T T T . . . . . . . T T T T T T T T T T    
956	. . . . . . . . . . . . . . . . . . . . . . . . . . . . . . . C C C C C C C C C C C C C C C C C C C C C C C C C C . C C C C C C C C C C C C C C C C C C C . . C C C C C C C C C C C C C C C C C C C C C C C C C C C C C C C C C C C C C C C C C C C . . . . . . . C C C C C C C C C C    
957	. . . . . . . . . . . . . . . . . . . . . . . . . . . . . . . A A A A A A A A A A A A A A A A A A A A A A A A A A . A A A A A A A A A A A A A A A A A A A . . A A A A A A A A A A A A A A A A A A A A A A A A A A A A A A A A A A A A A A A A A A A . . . . . . . A A A A A A A A A A    
958	. . . . . . . . . . . . . . . . . . . . . . . . . . . . . . . . T T T T T T T T T T T T T T T T T T T T T T T T T . T T T T T T T T T T T T T T T T T T T . . T T T T T T T T T T T T T T T T T T T T T T T T T T T T T T T T T T T T T T T T T T T . . . . . . . T T T T T T T T T T    
959	. . . . . . . . . . . . . . . . . . . . . . . . . . . . . . . . A A A A A A A A A A A A A A A A A A A A A A A A A . A A A A A A A A A A A A A A A A A A A . . A A A A A A A A A A A A A A A A A A A A A A A A A A A A A A A A A A A G A A A A A A A . . . . . . . A A A A A A A A A A    
960	. . . . . . . . . . . . . . . . . . . . . . . . . . . . . . . . T T T T T T T T T T T T T T T T T T T T T T T T T . T T T T T T T T T T T T T T T T T T T . . T T T T T T T T T T T T T T T T T T T T T T T T T T T T T T T T T T T T T T T T T T T . . . . . . . T T T T T T T T T T    
961	. . . . . . . . . . . . . . . . . . . . . . . . . . . . . . . . T T T T T T T T T T T T T T T T T T T T T T T T T . T T T T T T T T T T T T T T T T T T T . . T T T T T T T T T T T T T T T T T T T T T T T T T T T T T T T T T T T T T T T T T T T . . . . . . . T T T T T T T T T T    
962	. . . . . . . . . . . . . . . . . . . . . . . . . . . . . . . . A A A A A A A A A A A A A A A A A A A A A A A A A . A A A A A A A A A A A A A A A A A A A A . A A A A A A A A A A A A A A A A A A A A A A A A A A A A A A A A A A A A A A A A A A A . . . . . . . A A A A A A A A A A    
963	. . . . . . . . . . . . . . . . . . . . . . . . . . . . . . . . A A A A A A A A A A A A A A A A A A A A A A A A A . A A A A A A A A A A A A A A A A A A A A . A A A A A A A A A A A A A A A A A A A A A A A A A A A A A A A A A A A A A A A A A A A . . . . . . . A A A A A A A A A A    
964	. . . . . . . . . . . . . . . . . . . . . . . . . . . . . . . . T T T T T T T T T T T T T T T T T T T T T T T T T . T T T T T T T T T T T T T T T T T T T T . T T T T T T T T T T T T T T T T T T T T T T T T T T T T T T T T T T T T T T T T T T T . . . . . . . T T T T T T T T T T    
965	. . . . . . . . . . . . . . . . . . . . . . . . . . . . . . . . C C C C C C C C C C C C C C C C C C C C C C C C C . C C C C C C C C C C C C C C C C C C C C . C C C C C C C C C C C C C C C C C C C C C C C C C C C C C C C C C C C C C C C C C C C . . . . . . . C C C C C C C C C C    
966	. . . . . . . . . . . . . . . . . . . . . . . . . . . . . . . . C C C C C C C C C C C C C C C C C C C C C C C C C . C C C C C C C C C C C C C C C C C C C C . C C C C C C C C C C C C C C C C C C C C C C C C C C C C C C C C C C C C C C C C C C C . . . . . . . C C C C C C C C C C    
967	. . . . . . . . . . . . . . . . . . . . . . . . . . . . . . . . A A A A A A A A A A A A A A A A A A A A A A A A A . A A A A A A A A A A A A A A A A A A A A . A A A A A A A A A A A A A A A A A A A A A A A A A A A A A A A A A A A A A A A A A A A . . . . . . . A A A A A A A A A A    
968	. . . . . . . . . . . . . . . . . . . . . . . . . . . . . . . . . T T T T T T T T T T T T T T T T T T T T T T T T . T T T T T T T T T T T T T T T T T T T T . T T T T T T T T T T T T T T T T T T T T T T T T T T T T T T T T T T T T T T T T T T T . . . . . . . T T T T T T T T T T    
969	. . . . . . . . . . . . . . . . . . . . . . . . . . . . . . . . . T T T T T T T T T T T T T T T T T T T T T T T T . T T T T T T T T T T T T T T T T T T T T . T T T T T T T T T T T T T T T T T T T T T T T T T T T T T T T T T T T T T T T T T T T . . . . . . . T T T T T T T T T T    
970	. . . . . . . . . . . . . . . . . . . . . . . . . . . . . . . . . A A A A A A A A A A A A A A A A A A A A A A A A . A A A A A A A A A A A A A A A A A A A A . A A A A A A A A A A A A A A A A A A A A A A A A A A A A A A A A A A A A A A A A A A A . . . . . . . A A A A A A A A A A    
971	. . . . . . . . . . . . . . . . . . . . . . . . . . . . . . . . . A A A A A A A A A A A A A A A A A A A A A A A A . A A A A A A A A A A A A A A A A A A A A . A A A A A A A A A A A A A A A A A A A A A A A A A A A A A A A A A A A A A G A A A A A . . . . . . . A A A A A A A A A A    
972	. . . . . . . . . . . . . . . . . . . . . . . . . . . . . . . . . C C C C C C C C C C C C C C C C C C C C C C C C . C C C C C C C C C C C C C C C C C C C C . C C C C C C C C C C C C C C C C C C C C C C C C C C C C C C C C C C C C C C C C C C C . . . . . . . C C C C C C C C C C    
973	. . . . . . . . . . . . . . . . . . . . . . . . . . . . . . . . . A A A A A A A A A A A A A A A A A A A A A A A A . A A A A A A A A A A A A A A A A A A A A . A A A A A A A A A A A A A A A A A A A A A A A A A A A A A A A A A A A A A A A A A A A . . . . . . . A A A A A A A A A A    
974	. . . . . . . . . . . . . . . . . . . . . . . . . . . . . . . . . A A A A A A A A A A A A A A A A A A A A A A A A . A A A A A A A A A A A A A A A A A A A A . A A A A A A A A A A A A A A A A A A A A A A A A A A A A A A A A A A A A A A A A A A A . . . . . . . A A A A A A A A A A    
975	. . . . . . . . . . . . . . . . . . . . . . . . . . . . . . . . . C C C C C C C C C C C C C C C C C C C C C C C C . C C C C C C C C C C C C C C C C C C C C . C C C C C C C C C C C C C C C C C C C C C C C C C C C C C C C C C C C C C C C C C C C . . . . . . . C C C C C C C C C C    
976	. . . . . . . . . . . . . . . . . . . . . . . . . . . . . . . . . T T T T T T T T T T T T T T T T T T T T T T T T . T T T T T T T T T T T T T T T T T T T T . T T T T T T T T T T T T T T T T T T T T T T T T T T T T T T T T T T T T T T T T T T T . . . . . . . T T T T T T T T T T    
977	. . . . . . . . . . . . . . . . . . . . . . . . . . . . . . . . . A A A A A A A A A A A A A A A A A A A A A A A A . A A A A A A A A A A A A A A A A A A A A . A A A A A A A A A A A A A A A A A A A A A A A A A A A A A A A A A A A A A A A A A A A . . . . . . . A A A A A A A A A A    
978	. . . . . . . . . . . . . . . . . . . . . . . . . . . . . . . . . T T T T T T T T T T T T T T T T T T T T T T T T . T T T T T T T T T T T T T T T T T T T T . T T T T T T T T T T T T T T T T T T T T T T T T T T T T T T T T T T T T T T T T T T T . . . . . . . T T T T T T T T T T    
979	. . . . . . . . . . . . . . . . . . . . . . . . . . . . . . . . . A A A A A A A A A A A A A A A A A A A A A A A A . A A A A A A A A A A A A A A A A A A A A . A A A A A A A A A A A A A A A A A A A A A A A A A A A A A A A A A A A A A A A A A A A . . . . . . . A A A A A A A A A A    
980	. . . . . . . . . . . . . . . . . . . . . . . . . . . . . . . . . G G G G G G G G G G G G G G G G G G G G G G G G . G G G G G G G G G G G G G G G G G G G G . G G G G G G G G G G G G G G G G G G G G G G G G G G G G G G G G G G G G G G G G G G G . . . . . . . G G G G G G G G G G    
981	. . . . . . . . . . . . . . . . . . . . . . . . . . . . . . . . . G G G G G G G G G G G G G G G G G G G G G G G G . G G G G G G G G G G G G G G G G G G G G . G G G G G G G G G G G G G G G G G G G G G G G G G G G G G G G G G G G G G G G G G G G . . . . . . . G G G G G G G G G G    
982	. . . . . . . . . . . . . . . . . . . . . . . . . . . . . . . . . C C C C C C C C C C C C C C C C C C C C C C C C . C C C C C C C C C C C C C C C C C C C C . C C C C C C C C C C C C C C C C C C C C C C C C C C C C C C C C C C C C C C C C C C C . . . . . . . C C C C C C C C C C    
983	. . . . . . . . . . . . . . . . . . . . . . . . . . . . . . . . . T T T T T T T T T T T T T T T T T T T T T T T T . T T T T T T T T T T T T T T T T T T T T . T T T T T T T T T T T T T T T T T T T T T T T T T T T T T T T T T T T T T T T T T T T . . . . . . . T T T T T T T T T T    
984	. . . . . . . . . . . . . . . . . . . . . . . . . . . . . . . . . T T T T T T T T T T T T T T T T T T T T T T T T . T T T T T T T T T T T T T T T T T T T T . T T T T T T T T T T T T T T T T T T T T T T T T T T T T T T T T T T T T T T T T T T T . . . . . . . T T T T T T T T T T    
985	. . . . . . . . . . . . . . . . . . . . . . . . . . . . . . . . . G G G G G G G G G G G G G G G G G G G G G G G G . G G G G G G G G G G G G G G G G G G G G . G G G G G G G G G G G G G G G G G G G G G G G G G G G G G G G G G G G G G G G G G G G . . . . . . . G G G G G G G G G G    
986	. . . . . . . . . . . . . . . . . . . . . . . . . . . . . . . . . A A A A A A A A A A A A A A A A A A A A A A A A . A A A A A A A A A A A A A A A A A A A A . A A A A A A A A A A A A A A A A A A A A A A A A A A A A A A A A A A A A A A A A A A A . . . . . . . A A A A A A A A A A    
987	. . . . . . . . . . . . . . . . . . . . . . . . . . . . . . . . . T T T T T T T T T T T T T T T T T T T T T T T T . T T T T T T T T T T T T T T T T T T T T . T T T T T T T T T T T T T T T T T T T T T T T T T T T T T T T T T T T T T T T T T T T . . . . . . . T T T T T T T T T T    
988	. . . . . . . . . . . . . . . . . . . . . . . . . . . . . . . . . A A A A A A A A A A A A A A A A A A A A A A A A . A A A A A A A A A A A A A A A A A A A A . A A A A A A A A A A A A A A A A A A A A A A A A A A A A A A A A A A A A A A A A A A A . . . . . . . A A A A A A A A A A    
989	. . . . . . . . . . . . . . . . . . . . . . . . . . . . . . . . . G G G G G G G G G G G G G G G G G G G G G G G G . G G G G G G G G G G G G G G G G G G G G . G G G G G G G G G G G G G G G G G G G G G G G G G G G G G G G G G G G G G G G G G G G . . . . . . . G G G G G G G G G G    
990	. . . . . . . . . . . . . . . . . . . . . . . . . . . . . . . . . T T C T T T T T T T T T T T T T T T T T T T T T . T T T T T T T T T T T T T T T T T T T T . T T T T T T T T T T T T T T T T T T T T T T T T T T T T T T T T T T T T T T T T T T T . . . . . . . T T T T T T T T T T    
991	. . . . . . . . . . . . . . . . . . . . . . . . . . . . . . . . . T A A A A A A A A A A A A A A A A A A A A A A A . A A A A A A A A A A A A A A A A A A A A . A A A A A A A A A A A A A A A A A A A A A A A A A A A A A A A A A A A A A A A A A A A . . . . . . . A A A A A A A A A A    
992	. . . . . . . . . . . . . . . . . . . . . . . . . . . . . . . . . . T T T T T T T T T T T T T T T T T T T T T T T . T T T T T T T T T T T T T T T T T T T T . T T T T T T T T T T T T T T T T T T T T T T T T T T T T T T T T T T T T T T T T T T T . . . . . . . T T T T T T T T T T    
993	. . . . . . . . . . . . . . . . . . . . . . . . . . . . . . . . . . T T T T T T T T T T T T T T T T T T T T T T T . T T T T T T T T T T T T T T T T T T T T T T T T T T T T T T T T T T T T T T T T T T T T T T T T T T T T T T T T T T T T T T T T . . . . . . . T T T T T T T T T T    
994	. . . . . . . . . . . . . . . . . . . . . . . . . . . . . . . . . . T T T T T T T T T T T T T T T T T T T T T T T . T T T T T T T T T T T T T T T T T T T T T T T T T T T T T T T T T T T T T T T T T T T T T T T T T T T T T T T T T T T T T T T T . . . . . . . T T T T T T T T T T    
995	. . . . . . . . . . . . . . . . . . . . . . . . . . . . . . . . . . A A A A A A A A A A A A A A A A A A A A A A A . A A A A A A A A A A A A A A A A A A A A A A A A A A A A A A A A A A A A A A A A A A A A A A A A A A A A A A A A A A A A A A A A . . . . . . . A A A A A A A A A A    
996	. . . . . . . . . . . . . . . . . . . . . . . . . . . . . . . . . . G G G G G G G G G G G G G G G G G G G G G G G . G G G G G G G G G G G G G G G G G G G G G G G G G G G G G G G G G G G G G G G G G G G G G G G G G G G G G G G G G G G G G G G G . . . . . . . G G G G G G G G G G    
997	. . . . . . . . . . . . . . . . . . . . . . . . . . . . . . . . . . A A A A A A A A A A A A A A A A A A A A A A A . A A A A A A A A A A A A A A A A A A A A A A A A A A A A A A A A A G A A A A A A A A A A A A A A A A A A A A A A A A A A A A A A . . . . . . . A A A A A A A A A A    
998	. . . . . . . . . . . . . . . . . . . . . . . . . . . . . . . . . . C C C C C C C C C C C C C C C C C C C C C C C . C C C C C C C C C C C C C C C C C C C C C C C C C C C C C C C C C C C C C C C C C C C C C C C C C C C C C C C C C C C C C C C C . . . . . . . C C C C C C C C C C    
999	. . . . . . . . . . . . . . . . . . . . . . . . . . . . . . . . . . A A A A A A A A A A A A A A A A A A A A A A A . A A A A A A A A A A A A A A A A A A A A A A A A A A A A A A A A A A A A A A A A A A A A A A A A A A A A A A A A A A A A A A A A . . . . . . . A A A A A A A A A A    
1000	. . . . . . . . . . . . . . . . . . . . . . . . . . . . . . . . . . G G G G G G G G G G G G G G G G G G G G G G G . G G G G G G G G G G G G G G G G G G G G G G G G G G G G G G G G G G G G G G G G G G G G G G G G G G G G G G G G G G G G G G G G . . . . . . . G G G G G G G G G G    
1001	. . . . . . . . . . . . . . . . . . . . . . . . . . . . . . . . . . T T T T T T T T T T T T T T T T T T T T T T T . T T T T T T T T T T T T T T T T T T T T T T T T T T T T T T T T T T T T T T T T T T T T T T T T T T T T T T T T T T T T T T T T . . . . . . . T T T T T T T T T T    
1002	. . . . . . . . . . . . . . . . . . . . . . . . . . . . . . . . . . T T T T T T T T T T T T T T T T T T T T T T T . T T T T T T T T T T T T T T T T T T T T T T T T T T T T T T T T T T T T T T T T T T T T T T T T T T T T T T T T T T T T T T T T . . . . . . . T T T T T T T T T T    
1003	. . . . . . . . . . . . . . . . . . . . . . . . . . . . . . . . . . G G G G G G G G G G G G G G G G G G G G G G G . G G G G G G G G G G G G G G G G G G G G G G G G G G G G G G G G G G G G G G G G G G G G G G G G G G G G G G G G G G G G G G G G . . . . . . . G G G G G G G G G G    
1004	. . . . . . . . . . . . . . . . . . . . . . . . . . . . . . . . . . T T T T T T T T T T T T T T T T T T T T T T T . T T T T T T T T T T T T T T T T T T T T T T T T T T T T T T T T T T T T T T T T T T T T T T T T T T T T T T T T T T T T T T T T . . . . . . . T T T T T T T T T T    
1005	. . . . . . . . . . . . . . . . . . . . . . . . . . . . . . . . . . T T T T T T T T T T T T T T T T T T T T T T T . T T T T T T T T T T T T T T T T T T T T T T T T T T T T T T T T T T T T T T T T T T T T T T T T T T T T T T T T T T T T T T T T . . . . . . . T T T T T T T T T T    
1006	. . . . . . . . . . . . . . . . . . . . . . . . . . . . . . . . . . T T T T T T T T T T T T T T T T T T T T T T T . T T T T T T T T T T T T T T T T T T T T T T T T T T T T T T T T T T T T T T T T T T T T T T T T T T T T T T T T T T T T T T T T . . . . . . . T T T T T T T T T T    
1007	. . . . . . . . . . . . . . . . . . . . . . . . . . . . . . . . . . A A A A A A A A A A A A A A A A A A A A A A A . A A A A A A A A A A A A A A A A A A A A A A A A A A A A A A A A A A A A A A A A A A A A A A A A A A A A A A A A A A A A A A A A . . . . . . . A A A A A A A A A A    
1008	. . . . . . . . . . . . . . . . . . . . . . . . . . . . . . . . . . T T T T T T T T T T T T T T T T T T T T T T T . T T T T T T T T T T T T T T T T T T T T T T T T T T T T T T T T T T T T T T T T T T T T T T T T T T T T T T T T T T T T T T T T . . . . . . . T T T T T T T T T T    
1009	. . . . . . . . . . . . . . . . . . . . . . . . . . . . . . . . . . - - T T - - T - - - - T - - - - - - - - - - T . - - - - - - - - - - - - - - T T - - - - - - - - - - - - - - - - - - - - - - - - - - - - - - - - - - - - - - - - - - - - - - T T . . . . . . . T T T T T T T T T T  19	 indel  
1010	. . . . . . . . . . . . . . . . . . . . . . . . . . . . . . . . . . - - A A - - A - - - - A - - - - - - - - - - A . - - - - - - - - - - - - - - A A - - - - - - - - - - - - - - - - - - - - - - - - - - - - - - - - - - - - - - - - - - - - - - A A . . . . . . . A A A A A A A A A A  19	 indel  
1011	. . . . . . . . . . . . . . . . . . . . . . . . . . . . . . . . . . - - C C - - C - - - - C - - - - - - - - - - C . - - - - - - - - - - - - - - C C - - - - - - - - - - - - - - - - - - - - - - - - - - - - - - - - - - - - - - - - - - - - - - C C . . . . . . . C C C C C C C C C C  19	 indel  
1012	. . . . . . . . . . . . . . . . . . . . . . . . . . . . . . . . . . - - C C - - C - - - - C - - - - - - - - - - C . - - - - - - - - - - - - - - C C - - - - - - - - - - - - - - - - - - - - - - - - - - - - - - - - - - - - - - - - - - - - - - C C . . . . . . . C C C C C C C C C C  19	 indel  
1013	. . . . . . . . . . . . . . . . . . . . . . . . . . . . . . . . . . - - T T - - T - - - - T - - - - - - - - - - T . - - - - - - - - - - - - - - T T - - - - - - - - - - - - - - - - - - - - - - - - - - - - - - - - - - - - - - - - - - - - - - T T . . . . . . . T T T T T T T T T T  19	 indel  
1014	. . . . . . . . . . . . . . . . . . . . . . . . . . . . . . . . . . T T T T T T T T T T T T T T T T T T T T T T T . T T T T T T T T T T T T T T T T T T T T T T T T T T T T T T T T T T T T T T T T T T T T T T T T T T T T T T T T T T T T T T T T . . . . . . . T T T T T T T T T T    
1015	. . . . . . . . . . . . . . . . . . . . . . . . . . . . . . . . . . A A A A A A A A A A A A A A A A A A A A A A A . A A A A A A A A A A A A A A A A A A A A A A A A A A A A A A A A A A A A A A A A A A A A A A A A A A A A A A A A A A A A A A A A . . . . . . . A A A A A A A A A A    
1016	. . . . . . . . . . . . . . . . . . . . . . . . . . . . . . . . . . A A A A A A A A A A A A A A A A A A A A A A A . A A A A A A A A A A A A A A A A A A A A A A A A A A A A A A A A A A A A A A A A A A A A A A A A A A A A A A A A A A A A A A A A . . . . . . . A A A A A A A A A A    
1017	. . . . . . . . . . . . . . . . . . . . . . . . . . . . . . . . . . T T T T T T T T T T T T T T T T T T T T T T T . T T T T T T T T T T T T T T T T T T T T T T T T T T T T T T T T T T T T T T T T T T T T T T T T T T T T T T T T T T T T T T T T . . . . . . . T T T T T T T T T T    
1018	. . . . . . . . . . . . . . . . . . . . . . . . . . . . . . . . . . A A A A A A A A A A A A A A A A A A A A A A A . A A A A A A A A A A A A A A A A A A A A A A A A A A A A A A A A A A A A A A A A A A A A A A A A A A A A A A A A A A A A A A A A . . . . . . . A A A A A A A A A A    
1019	. . . . . . . . . . . . . . . . . . . . . . . . . . . . . . . . . . G G G G G G G G G G G G G G G G G G G G G G G . G G G G G G G G G G G G G G G G G G G G G G G G G G G G G G G G G G G G G G G G G G G G G G G G G G G G G G G G G G G G G G G G . . . . . . . G G G G G G G G G G    
1020	. . . . . . . . . . . . . . . . . . . . . . . . . . . . . . . . . . A A A A A A A A A A A A A A A A A A A A A A A . A A A A A A A A A A A A A A A A A A A A A A A A A A A A A A A A A A A A A A A A A A A A A A A A A A A A A A A A A A A A A A A A . . . . . . . A A A A A A A A A A    
1021	. . . . . . . . . . . . . . . . . . . . . . . . . . . . . . . . . . T T T T T T T T T T T T T T T T T T T T T T T . T T T T T T T T T T T T T T T T T T T T T T T T T T T T T T T T T T T T T T T T T T T T T T T T T T T T T T T T T T T T T T T T . . . . . . . T T T T T T T T T T    
1022	. . . . . . . . . . . . . . . . . . . . . . . . . . . . . . . . . . G G G G G G G G G G G G G G G G G G G G G G G . G G G G G G G G G G G G G G G G G G G G G G G G G G G G G G G G G G G G G G G G G G G G G G G G G G G G G G G G G G G G G G G G . . . . . . . G G G G G G G G G G    
1023	. . . . . . . . . . . . . . . . . . . . . . . . . . . . . . . . . . T T T T T T T T T T T T T T T T T T T T T T T . T T T T T T T T T T T T T T T T T T T T T T T T T T T T T T T T T T T T T T T T T T T T T T T T T T T T T T T T T T T T T T T T . . . . . . . T T T T T T T T T T    
1024	. . . . . . . . . . . . . . . . . . . . . . . . . . . . . . . . . . G G G G G G G G G G G G G G G G G G G G G G G . G G G G G G G G G G G G G G G G G G G G G G G G G G G G G G G G G G G G G G G G G G G G G G G G G G G G G G G G G G G G G G G G . . . . . . . G G G G G G G G G G    
1025	. . . . . . . . . . . . . . . . . . . . . . . . . . . . . . . . . . G G G G G G G G G G G G G G G G G G G G G G G . G G G G G G G G G G G G G G G G G G G G G G G G G G G G G G G G G G G G G G G G G G G G G G G G G G G G G G G G G G G G G G G G . . . . . . . G G G G G G G G G G    
1026	. . . . . . . . . . . . . . . . . . . . . . . . . . . . . . . . . . T T T T T T T T T T T T T T T T T T T T T T T . T T T T T T T T T T T T T T T T T T T T T T T T T T T T T T T T T T T T T T T T T T T T T T T T T T T T T T T T T T T T T T T T . . . . . . . T T T T T T T T T T    
1027	. . . . . . . . . . . . . . . . . . . . . . . . . . . . . . . . . . A A A A A A A A A A A A A A A A A A A A A A A . A A A A A A A A A A A A A A A A A A A A A A A A A A A A A A A A A A A A A A A A A A A A A A A A A A A A A A A A A A A A A A A A . . . . . . . A A A A A A A A A A    
1028	. . . . . . . . . . . . . . . . . . . . . . . . . . . . . . . . . . G G G G G G G G G G G G G G G G G G G G G G G . G G G G G G G G G G G G G G G G G G G G G G G G G G G G G G G G G G G G G G G G G G G G G G G G G G G G G G G G G G G G G G G G . . . . . . . G G G G G G G G G G    
1029	. . . . . . . . . . . . . . . . . . . . . . . . . . . . . . . . . . A A A A A A A A A A A A A A A A A A A A A A A . A A A A A A A A A A A A A A A A A A A A A A A A A A A A A A A A A A A A A A A A A A A A A A A A A A A A A A A A A A A A A A A A . . . . . . . A A A A A A A A A A    
1030	. . . . . . . . . . . . . . . . . . . . . . . . . . . . . . . . . . G G G G G G G G G G G G G G G G G G G G G G G . G G G G G G G G G G G G G G G G G G G G G G G G G G G G G G G G G G G G G G G G G G G G G G G G G G G G G G G G G G G G G G G G . . . . . . . G G G G G G G G G G    
1031	. . . . . . . . . . . . . . . . . . . . . . . . . . . . . . . . . . A A A A A A A A A A A A A A A A A A A A A A A . A A A A A A A A A A A A A A A A A A A A A A A A A A A A A A A A A A A A A A A A A A A A A A A A A A A A A A A A A A A A A A A A . . . . . . . A A A A A A A A A A    
1032	. . . . . . . . . . . . . . . . . . . . . . . . . . . . . . . . . . T T T T T T T T T T T T T T T T T T T T T T T . T T T T T T T T T T T T T T T T T T T T T T T T T T T T T T T T T T T T T T T T T T T T T T T T T T T T T T T T T T T T T T T T . . . . . . . T T T T T T T T T T    
1033	. . . . . . . . . . . . . . . . . . . . . . . . . . . . . . . . . . C C C C C C C C C C C C C C C T C C C C C C C . C C C C C C C C C C C C C C C C C C C C C C C C C C C C C C C C C C C C C C C C C C C C C C C C C C C C C C C C C C C C C C C C . . . . . . . C C C C C C C C C C    
1034	. . . . . . . . . . . . . . . . . . . . . . . . . . . . . . . . . . T T T T T T T T T T T T T T T T T T T T T T T . T T T T T T T T T T T T T T T T T T T T T T T T T T T T T T T T T T T T T T T T T T T T T T T T T T T T T T T T T T T T T T T T . . . . . . . T T T T T T T T T T    
1035	. . . . . . . . . . . . . . . . . . . . . . . . . . . . . . . . . . G G G G G G G G G G G G G G G G G G G G G G G . G G G G G G G G G G G G G G G G G G G G G G G G G G G G G G G G G G G G G G G G G G G G G G G G G G G G G G G G G G G G G G G G . . . . . . . G G G G G G G G G G    
1036	. . . . . . . . . . . . . . . . . . . . . . . . . . . . . . . . . . C C C C C C C C C C C C C C C C C C C C C C C . C C C C C C C C C C C C C C C C C C C C C C C C C C C C C C C C C C C C C C C C C C C C C C C C C C C C C C C C C C C C C C C C . . . . . . . C C C C C C C C C C    
1037	. . . . . . . . . . . . . . . . . . . . . . . . . . . . . . . . . . T T T T T T T T T T T T T T T T T T T T T T T . T T T T T T T T T T T T T T T T T T T T T T T T T T T T T T T T T T T T T T T T T T T T T T T T T T T T T T T T T T T T T T T T . . . . . . . T T T T T T T T T T    
1038	. . . . . . . . . . . . . . . . . . . . . . . . . . . . . . . . . . A . T A A A A A T A A A A A A A T A A A A A A . A A A A A A A A A A A A A A A A A A A A A A A A A A A A A A A A A A A A A A A A A A A A A A A A A A A A A T A A A A A A A A A A . . . . . . . A A A A A A A A A A    
1039	. . . . . . . . . . . . . . . . . . . . . . . . . . . . . . . . . . T . . T T T T T T T T T T T T T T T T T T T T . T T T T T T T T T T T T T T T T T T T T T T T T T T T T T T T T T T T T T T T T T T T T T T T T T T T T T T T T T T T T T T T T . . . . . . . T T T T T T T T T T    
1040	. . . . . . . . . . . . . . . . . . . . . . . . . . . . . . . . . . A . . A A A A A A A A A A A A A A A A A A A A . A A A A A A A A A A A A A A A A A A A A A A A A A A A A A A A A A A A A A A A A A A A A A A A A A A A A A A A A A A A A A A A A . . . . . . . A A A A A A A A A A    
1041	. . . . . . . . . . . . . . . . . . . . . . . . . . . . . . . . . . A . . A A A A A A A A A A A A A A A A A A A A . A A A A A A A A A A A A A A A A A A A A A A A A A A A A A A A A A A A A A A A A A A A A A A A A A A A A A A A A A A A A A A A A . . . . . . . A A A A A A A A A A    
1042	. . . . . . . . . . . . . . . . . . . . . . . . . . . . . . . . . . C . . C C C C C C C C C C C C C C C C C C C C . C C C C C C C C C C C C C C C C C C C C C C C C C C C C C C C C C C C C C C C C C C C C C C C C C C C C C C C C C C C C C C C C . . . . . C C C C C C C C C C C C    
1043	. . . . . . . . . . . . . . . . . . . . . . . . . . . . . . . . . . A . . A A A A A A A A A A A A A A A A A A A A . A A A A A A A A A A A A A A A A A A A A A A A A A A A A A A A A A A A A A A A A A A A A A A A A A A A A A A A A A A A A A A A A . . . . . A A A A A A A A A A A A    
1044	. . . . . . . . . . . . . . . . . . . . . . . . . . . . . . . . . . C . . C C C C C C C C C C C C C C C C C C C C . C C C C C C C C C C C C C C C C C C C C C C C C C C C C C C C C C C C C C C C C C C C C C C C C C C C C C C C C C C C C C C C C . . . . . C C C C C C C C C C C C    
1045	. . . . . . . . . . . . . . . . . . . . . . . . . . . . . . . . . . A . . A A A A A A A A A A A A A A A A A A A A . A A A A A A A A A A A A A A A A A A A A A A A A A A A A A A A A A A A A A A A A A A A A A A A A A A A A A T A A A A A A A A A A . . . . . A A A A A A A A A A A A    
1046	. . . . . . . . . . . . . . . . . . . . . . . . . . . . . . . . . . G . . G G G G G G G G G G G G G G G G G G G G . G G G G G G G G G G G G G G G G G G G G G G G G G G G G G G G G G G G G G G G G G G G G G G G G G G G G G G G G G G G G G G G G . . . . . G G G G G G G G G G G G    
1047	. . . . . . . . . . . . . . . . . . . . . . . . . . . . . . . . . . T . . T T T T T T T T T T T T T T T T T T T T . T T T T T T T T T T T T T T T T T T T T T T T T T T T T T T T T T T T T T T T T T T T T T T T T T T T T T T T T T T T T T T T T . . . . . T T T T T T T T T T T T    
1048	. . . . . . . . . . . . . . . . . . . . . . . . . . . . . . . . . . A . . A A A A A A A A A A A A A A A A A A A A . A A A A A A A A A A A A A A A A A A A A A A A A A A A A A A A A A A A A A A A A A A A A A A A G A A A A A A A A A A A A A A A A . . . . . A A A A A A A A A A A A    
1049	. . . . . . . . . . . . . . . . . . . . . . . . . . . . . . . . . . A . . A A A A A A A A A A A A A A A A A A A A . A A A A A A A A A A A A A A A A A A A A A A A A A A A A A A A A A A A A A A A A A A A A A A A A A A A A A A A A A A A A A A A A . . . . . A A A A A A A A A A A A    
1050	. . . . . . . . . . . . . . . . . . . . . . . . . . . . . . . . . . G . . G G G G G G G G G G G G G G G G G G G G . G G G G G G G G G G G G G G G G G G G G G G G G G G G G G G G G G G G G G G G G G G G G G G G G G G G G G G G G G G G G G G G G . . . . . G G G G G G G G G G G G    
1051	. . . . . . . . . . . . . . . . . . . . . . . . . . . . . . . . . . T . . T T T T T T T T T T T T T T T T T T T T . T T T T T T T T T T T T T T T T T T T T T T T T T T T T T T T T T T T T T T T T T T T T T T T T T T T T T T T T T T T T T T T T . . . . . T T T T T T T T T T T T    
1052	. . . . . . . . . . . . . . . . . . . . . . . . . . . . . . . . . . T . . T T T T T T T T T T T T T T T T T T T T . T T T T T T T T T T T T T T T T T T T T T T T T T T T T T T T T T T T T T T T T T T T T T T T T T T T T T T T T T T T T T T T T . . . . . T T T T T T T T T T T T    
1053	. . . . . . . . . . . . . . . . . . . . . . . . . . . . . . . . . . A . . A A A A A A A A A A A A A A A A A A A A . A A A A A A A A A A A A A A A A A A A A A A A A A A A A A A A A A A A A A A A A A A A A A A A A A A A A A A A A A A A A A A A A . . . . . A A A A A A A A A A A A    
1054	. . . . . . . . . . . . . . . . . . . . . . . . . . . . . . . . . . T . . T T T T T T T T T T T T T T T T T T T T . T T T T T T T T T T T T T T T T T T T T T T T T T T T T T T T T T T T T T T T T T T T T T T T T T T T T T T T T T T T T T T T T . . . . . T T T T T T T T T T T T    
1055	. . . . . . . . . . . . . . . . . . . . . . . . . . . . . . . . . . T . . T T T T T T T T T T T T T T T T T T T T . T T T T T T T T T T T T T T T T T T T T T T T T T T T T T T T T T T T T T T T T T T T T T T T T T T T T T T T T T T T T T T T T . . . . . T T T T T T T T T T T T    
1056	. . . . . . . . . . . . . . . . . . . . . . . . . . . . . . . . . . C . . C C C C C C C C C C C C T C C C C C C C . C C C C C C C C C C C C C C C C C C C C C C C C C C C C C C C C C C C C C C C C C C C C C C C C C C C C C C C C C C C C C C C C . . . . . C C T T C C C C T C C C    
1057	. . . . . . . . . . . . . . . . . . . . . . . . . . . . . . . . . . T . . T T T T T T T T T T T T T T T T T T T T . T T T T T T T T T T T T T T T T T T T T T T T T T T T T T T T T T T T T T T T T T T T T T T T T T T T T T T T T T T T T T T T T . . . . . T T T T T T T T T T T T    
1058	. . . . . . . . . . . . . . . . . . . . . . . . . . . . . . . . . . A . . A A A A A A A A A A A A A A A A A A A A . A A A A A A A A A A A A A A A A A A A A A A A A A A A A A A A A A A A A A A A A A A A A A A A A A A A A A A A A A A A A A A A A . . . . . A A A A A A A A A A A A    
1059	. . . . . . . . . . . . . . . . . . . . . . . . . . . . . . . . . . G . . G G G G G G G G G G G G G G G G G G G G . G G G G G G G G G G G G G G G G G G G G G G G G G G G G G G G G G G G G G G G G G G G G G G G G G G G G G G G G G G G G G G G G . . . . . G G G G G G G G G G G G    
1060	. . . . . . . . . . . . . . . . . . . . . . . . . . . . . . . . . . G . . G G G G G G G G G G G G G G G G G G G G . G G G G G G G G G G G G G G G G G G G G G G G G G G G G G G G G G G G G G G G G G G G G G G G G G G G G G G G G G G G G G G G G . . . . . G G G G G G G G G G G G    
1061	. . . . . . . . . . . . . . . . . . . . . . . . . . . . . . . . . . G . . G G G G G G G G G G G G G G G G G G G G . G G G G G G G G G G G G G G G G G G G G G G G G G G G G G G G G G G G G G G G G G G G G G G G G G G G G G G G G G G G G G G G G . . . . . G G G G G G G G G G G G    
1062	. . . . . . . . . . . . . . . . . . . . . . . . . . . . . . . . . . G . . G G G G G G G G G G G G G G G G G G G G . G G G G G G G G G G G G G G G G G G G G G G G G G G G G G G G G G G G G G G G G G G G G G G G G G G G G G G G G G G G G G G G G . . . . . G G G G G G G G G G G G    
1063	. . . . . . . . . . . . . . . . . . . . . . . . . . . . . . . . . . T . . T T T T T T T T T T T T T T T T T T T T . T T T T T T T T T T T T T T T T T T T T T T T T T T T T T T T T T T T T T T T T T T T T T T T T T T T T T T T T T T T T T T T T . . . . . T T T T T T T T T T T T    
1064	. . . . . . . . . . . . . . . . . . . . . . . . . . . . . . . . . . T . . T T T T T T T T T T T T T T T T T T T T . T T T T T T T T T T T T T T T T T T T T T T T T T T T T T T T T T T T T T T T T T T T T T T T T T T T T T T T T T T T T T T T T . . . . . T T T T T T T T T T T T    
1065	. . . . . . . . . . . . . . . . . . . . . . . . . . . . . . . . . . A . . A A A A A A A A A A A A A A A A A A A A . A A A A A A A A A A A A A A A A A A A A A A A A A A A A A A A A A A A A A A A A A A A A A A A A A A A A A A A A A A A A A A A A . . . . . A A A A A A A A A A A A    
1066	. . . . . . . . . . . . . . . . . . . . . . . . . . . . . . . . . . T . . T T T T T T T T T T T T T T T T T T T T . T T T T T T T T T T T T T T T T T T T T T T T T T T T T T T T T T T T T T T T T T T T T T T T T T T T T T T T T T T T T T T T T . . . . . T T T T T T T T T T T T    
1067	. . . . . . . . . . . . . . . . . . . . . . . . . . . . . . . . . . T . . T T T T T T T T T T T T T T T T T T T T . T T T T T T T T T T T T T T T T T T T T T T T T T T T T T T T T T T T T T T T T T T T T T T T T T T T T T T T T T T T T T T T T . . . . . T T T T T T T T T T T T    
1068	. . . . . . . . . . . . . . . . . . . . . . . . . . . . . . . . . . T . . T T T T T T T T T T T T T T T T T T T T . T T T T T T T T T T T T T T T T T T T T T T T T T T T T T T T T T T T T T T T T T T T T T T T T T T T T T T T T T T T T T T T T . . . . . T T T T T T T T T T T T    
1069	. . . . . . . . . . . . . . . . . . . . . . . . . . . . . . . . . . A . . A A A A A A A A A A A A A A A A A A A A . A A A A A A A A A A A A A A A A A A A A A A A A A A A A A A A A A A A A A A A A A A A A A A A A A A A A A G A A A A A A A A A A . . . . . A A A A A A A A A A A A    
1070	. . . . . . . . . . . . . . . . . . . . . . . . . . . . . . . . . . C . . C C C C C C C C C C C C C C C C C C C C . C C C C C C C C C C C C C C C C C C C C C C C C C C C C C C C C C C C C C C C C C C C C C C C C C C C C C C C C C C C C C C C C . . . . . C C C C C C C C C C C C    
1071	. . . . . . . . . . . . . . . . . . . . . . . . . . . . . . . . . . C . . C C C C C C C C C C C C C C C C C C C C . C C C C C C C C C C C C C C C C C C C C C C C C C C C C C C C C C C C C C C C C C C C C C C C C C C C C C C C C C C C C C C C C . . . . . C C C C C C C C C C C C    
1072	. . . . . . . . . . . . . . . . . . . . . . . . . . . . . . . . . . T . . T T T T T T T T T T T T T T T T T T T T . T T T T T T T T T T T T T T T T T T T T T T T T T T T T T T T T T T T T T T T T T T T T T T T T T T T T T T T T T T T T T T T T . . . . . T T T T T T T T T T T T    
1073	. . . . . . . . . . . . . . . . . . . . . . . . . . . . . . . . . . G . . G G G G G G G G G G G G G G G G G G G G . G G G G G G G G G G G G G G G G G G G G G G G G G G G G G G G G G G G G G G G G G G G G G G G G G G G G G G G G G G G G G G G G . . . . . G G G G G G G G G G G G    
1074	. . . . . . . . . . . . . . . . . . . . . . . . . . . . . . . . . . C . . C C C C C C C C C C C C C C C C C C C C . C C C C C C C C C C C C C C C C C C C C C C C C C C C C C C C C C C C C C C C C C C C C C C C C C C C C C C C C C C C C C C C C . . . . . C C C C C C C C C C C C    
1075	. . . . . . . . . . . . . . . . . . . . . . . . . . . . . . . . . . T . . T T T T T T G T T T T T T T T T T T T T . T T T T T T T T T T T T T T T T T T T T T T T T T T T T T T T T T T T T T T T T T T T T T T T T G T T T T T T T T T T T T T T T . . . . . T T T T T T T T T T T T    
1076	. . . . . . . . . . . . . . . . . . . . . . . . . . . . . . . . . . G . . G G G G G G G G G G G G G G G G G G G G . G G G G G G G G G G G G G G G G G G G G G G G G G G G G G G G G G G G G G G G G G G G G G G G G G G G G G G G G G G G G G G G G . . . . . G G G G G G G G G G G G    
1077	. . . . . . . . . . . . . . . . . . . . . . . . . . . . . . . . . . G . . T T T A T A T T A T T T T A T T T T T A . T T T T T T T T T T T T T T A A T T T T T T T T T T T T T T T T T T T T T T T T T T A A A T T T T T T T T T T T T T T T T T A A . . . . . A A A A A A T T T A A A  1	 transition  
1078	. . . . . . . . . . . . . . . . . . . . . . . . . . . . . . . . . . . . . . A A A A A A A A A A A A A A A A A A A . A A A A A A A A A A A A A A A A A A A A A A A A A A A A A A A A A A A A A A A A A A A A A A A A A A A A A A A A A A A A A A A A . . . . . A A A A A A A A A A A A    
1079	. . . . . . . . . . . . . . . . . . . . . . . . . . . . . . . . . . . . . . C C C C C C C C C C C C C C C C C C C . C C C C C C C C C C C C C C C C C C C C C C C C C C C C C C C C C C C C C C C C C C C C C C C C C C C C C C C C C C C C C C C C . . . . . C C C C C C C C C C C C    
1080	. . . . . . . . . . . . . . . . . . . . . . . . . . . . . . . . . . . . . . T T T T T T T T T T T T T T T T T T T . T T T T T T T T T T T T T T T T T T T T T T T T T T T T T T T T T T T T T T T T T T T T T T T T T T T T T T T T T T T T T T T T . . . . . T T T T T T T T T T T T    
1081	. . . . . . . . . . . . . . . . . . . . . . . . . . . . . . . . . . . . . . G G G G G G G G G G G G G G G G G G G . G G G G G G G G G G G G G G G G G G G G G G G G G G G G G G G G G G G G G G G G G G G G G G G G G G G G G G G G G G G G G G G G . . . . . G G G G G G G G G G G G    
1082	. . . . . . . . . . . . . . . . . . . . . . . . . . . . . . . . . . . . . . T T T T T T T T T T T T T T T T T T T . T T T T T T T T T T T T T T T T T T T T T T T T T T T T T T T T T T T T T T T T T T T T T T T T T T T T T T T T T T T T T T T T . . . . . T T T T T T T T T T T T    
1083	. . . . . . . . . . . . . . . . . . . . . . . . . . . . . . . . . . . . . . T T T T T T T T T T T T T T T T T T T . T T T T T T T T T T T T T T T T T T T T T T T T T T T T T T T T T T T T T T T T T T T T T T T T T T T T T T T T T T T T T T T T . . . . . T T T T T T T T T T T T    
1084	. . . . . . . . . . . . . . . . . . . . . . . . . . . . . . . . . . . . . . G G G G G G G G G G G G G G G G G G G . G G G G G G G G G G G G G G G G G G G G G G G G G G G G G G G G G G G G G G G G G G G G G G G G G G G G G G G G G G G G G G G G . . . . . G G G G G G G G G G G G    
1085	. . . . . . . . . . . . . . . . . . . . . . . . . . . . . . . . . . . . . . T T T T T T T T T T T T T T T T T T T . T T T T T T T T T T T T T T T T T T T T T T T T T T T T T T T T T T T T T T T T T T T T T T T T T T T T T T T T T T T T T T T T . . . . . T T T T T T T T T T T T    
1086	. . . . . . . . . . . . . . . . . . . . . . . . . . . . . . . . . . . . . . C C C C C C C C C C C C C C C C C C C . C C C C C C C C C C C C C C C C C C C C C C C C C C C C C C C C C C C C C C C C C C C C C C C C C C C C C C C C C C C C C C C C . . . . . C C C C C C C C C C C C    
1087	. . . . . . . . . . . . . . . . . . . . . . . . . . . . . . . . . . . . . . T T T T T T T T T T T T T T T T T T T . T T T T T T T T T T T T T T T T T T T T T T T T T T T T T T T T T T T T T T T T T T T T T T T T T T T T T T T T T T T T T T T T . . . . . T T T T T T T T T T T T    
1088	. . . . . . . . . . . . . . . . . . . . . . . . . . . . . . . . . . . . . . T T T T T T T T T T T T T T T T T T T . T T T T T T T T T T T T T T T T T T T T T T T T T T T T T T T T T T T T T T T T T T T T T T T T T T T T T T T T T T T T T T T T . . . . . T T T T T T T T T T T T    
1089	. . . . . . . . . . . . . . . . . . . . . . . . . . . . . . . . . . . . . . T T T T T T T T T T T T T T T T T T T . T T T T T T T T T T T T T T T T T T T T T T T T T T T T T T T T T T T T T T T T T T T T T T T T T T T T T T T T T T T T T T T T . . . . . T T T T T T T T T T T T    
1090	. . . . . . . . . . . . . . . . . . . . . . . . . . . . . . . . . . . . . . T T T T T T T T T T T T T T T T T T T . T T T T T T T T T T T T T T T T T T T T T T T T T T T T T T T T T T T T T T T T T T T T T T T T T T T T T T T T T T T T T T T T . . . . . T T T T T T T T T T T T    
1091	. . . . . . . . . . . . . . . . . . . . . . . . . . . . . . . . . . . . . . C C C C C C C C C C C C C C C C C C C . C C C C C C C C C C C C C C C C C C C C C C C C C C C C C C C C C C C C C C C C C C C C C C C C C C C C C C C C C C C C C C C C . . . . . C C C C C C C C C C C C    
1092	. . . . . . . . . . . . . . . . . . . . . . . . . . . . . . . . . . . . . . A C A A A A A A A A A A A A A A A A A . A A A A A A A A A A A A A A A A A A A A A A A A A A A A A A A A A A A A A A A A A A A A A A A A A A A A A A A A A A A A A A A A . . . . . A A A A A A A A A A A A    
1093	. . . . . . . . . . . . . . . . . . . . . . . . . . . . . . . . . . . . . . C . C C C C C C C C C C C C C C C C C . C C C C C C C C C C C C C C C C C C C C C C C C C C C C C C C C C C C C C C C C C C C C C C C C C C C C C C C C C C C C C C C C . . . . . C C C C C C C C C C C C    
1094	. . . . . . . . . . . . . . . . . . . . . . . . . . . . . . . . . . . . . . T . T T T T T T T T T T T T T T T T T . T T T T T T T T T T T T T T T T T T T T T T T T T T T T T T T T T T T T T T T T T T T T T T T T T T T T T T T T T T T T T T T T . . . . . T T T T T T T T T T T T    
1095	. . . . . . . . . . . . . . . . . . . . . . . . . . . . . . . . . . . . . . A . A A A A A A A A A A A A A A A A A . A A A A A A A A A A A A A A A A A A A A A A A A A A A A A A A A A A A A A A A A A A A A A A A A A A A A A A A A A A A A A A A A . . . . . A A A A A A A A A A A A    
1096	. . . . . . . . . . . . . . . . . . . . . . . . . . . . . . . . . . . . . . A . A A A A A A A A A A A A A A A A A . A A A A A A A A A A A A A A A A A A A A A A A A A A A A A A A A A A A A A A A A A A A A A A A A A A A A A A A A A A A A A A A A . . . . . A A A A A A A A A A A A    
1097	. . . . . . . . . . . . . . . . . . . . . . . . . . . . . . . . . . . . . . C . C C C C C C C C C C C C C C C C C . C C C C C C C C C C C C C C C C C C C C C C C C C C C C C C C C C C C C C C C C C C C C C C C C C C C C C C C C C C C C C C C C . . . . . C C C C C C C C C C C C    
1098	. . . . . . . . . . . . . . . . . . . . . . . . . . . . . . . . . . . . . . A . A A A A A A A A A A A A A A A A A . A A A A A A A A A A A A A A A A A A A A A A A A A A A A A A A A A A A A A A A A A A A A A A A A A A A A A A A A A A A A A A A A . . . . . A A A A A A A A A A A A    
1099	. . . . . . . . . . . . . . . . . . . . . . . . . . . . . . . . . . . . . . A . A A A A A A A A A A A A A A A A A . A A A A A A A A A A A A A A A A A A A A A A A A A A A A A A A A A A A A A A A A A A A A A A A A A A A A A A A A A A A A A A A A . . . . . A A A A A A A A A A A A    
1100	. . . . . . . . . . . . . . . . . . . . . . . . . . . . . . . . . . . . . . A . A A A A A A A A A A A A A A A A A . A A A A A A A A A A A A A A A A A A A A A A A A A A A A A A A A A A A A A A A A A A A A A A A A A A A A A A A A A A A A A A A A . . . . . A A A A A A A A A A A A    
1101	. . . . . . . . . . . . . . . . . . . . . . . . . . . . . . . . . . . . . . T . T T T T T T T T T T T T T T T T T . T T T T T T T T T T T T T T T T T T T T T T T T T T T T T T T T T T T T T T T T T T T T T T T T T T T T T T T T T T T T T T T T . . . . . T T T T T T T T T T T T    
1102	. . . . . . . . . . . . . . . . . . . . . . . . . . . . . . . . . . . . . . A . A A A A A A A A A A A A A A A A A . A A A A A A A A A A A A A A A A A A A A A A A A A A A A A A A A A A A A A A A A A A A A A A A A A A A A A A A A A A A A A A A A . . . . . A A A A A A A A A A A A    
1103	. . . . . . . . . . . . . . . . . . . . . . . . . . . . . . . . . . . . . . A . A A A A A A A A A A A A A A A A A . A A A A A A A A A A A A A A A A A A A A A A A A A A A A A A A A A A A A A A A A A A A A A A A A A A A A A A A A A A A A A A A A . . . . . A A A A A A A A A A A A    
1104	. . . . . . . . . . . . . . . . . . . . . . . . . . . . . . . . . . . . . . C . C C C C C C C C C C C C C C C C C . C C C C C C C C C C C C C C C C C C C C C C C C C C C C C C C C C C C C C C C C C C C C C C C C C C C C C C C C C C C C C C C C . . . . . C C C C C C C C C C C C    
1105	. . . . . . . . . . . . . . . . . . . . . . . . . . . . . . . . . . . . . . A . A A A A A A A A A A A A A A A A A . A A A A A A A A A A A A A A A A A A A A A A A A A A A A A A A A A A A A A A A A A A A A A A A A A A A A A A A A A A A A A A A A . . . . . A A A A A A A A A A A A    
1106	. . . . . . . . . . . . . . . . . . . . . . . . . . . . . . . . . . . . . . C . C C C C C C C C C C C C C C C C C . C C C C C C C C C C C C C C C C C C C C C C C C C C C C C C C C C C C C C C C C C C C C C C C C C C C C C C C C C C C C C C C C . . . . . C C C C C C C C C C C C    
1107	. . . . . . . . . . . . . . . . . . . . . . . . . . . . . . . . . . . . . . A . A A A A A A A A A A A A A A A A A . A A A A A A A A A A A A A A A A A A A A A A A A A A A A A A A A A A A A A A A A A A A A A A A A A A A A A A A A A A A A A A A A . . . . . A A A A A A A A C A A A    
1108	. . . . . . . . . . . . . . . . . . . . . . . . . . . . . . . . . . . . . . C . C C C C C C C C C C C C C C C C C . C C C C C C C C C C C C C C C C C C C C C C C C C C C C C C C C C C C C C C C C C C C C C C C C C C C C C C C C C C C C C C C C . . . . . C C C C C C C C C C C C    
1109	. . . . . . . . . . . . . . . . . . . . . . . . . . . . . . . . . . . . . . C . C C C C C C C C C C C C C C C C C . C C C C C C C C C C C C C C C C C C C C C C C C C C C C C C C C C C C C C C C C C C C C C C C C C C C C C C C C C C C C C C C C . . . . . C C C C C C C C C C C C    
1110	. . . . . . . . . . . . . . . . . . . . . . . . . . . . . . . . . . . . . . T . T T T T T T T T T T T T T T T T T . T T T T T T T T T T T T T T T T T T T T T T T T T T T T T T T T T T T T T T T T T T T T T T T T T T T T T T T T T T T T T T T T . . . . . T T T T T T T T T T T T    
1111	. . . . . . . . . . . . . . . . . . . . . . . . . . . . . . . . . . . . . . G . G G G G G G G G G G G G G G G G G . G G G G G G G G G G G G G G G G G G G G G G G G G G G G G G G G G G G G G G G G G G G G G G G G G G G G G G G G G G G G G G G G . . . . . G G G G G G G G G G G G    
1112	. . . . . . . . . . . . . . . . . . . . . . . . . . . . . . . . . . . . . . T . T T T T T T T T T T T T T T T T T . T T T T T T T T T T T T T T T T T T T T T T T T T T T T T T T T T T T T T T T T T T T T T T T T T T T T T T T T T T T T T T T T . . . . . T T T T T T T T T T T T    
1113	. . . . . . . . . . . . . . . . . . . . . . . . . . . . . . . . . . . . . . T . T T T T T T T T T T T T T T T T T . T T T T T T T T T T T T T T T T T T T T T T T T T T T T T T T T T T T T T T T T T T T T T T T T T T T T T T T T T T T T T T T T . . . . . T T T T T T T T T T T T    
1114	. . . . . . . . . . . . . . . . . . . . . . . . . . . . . . . . . . . . . . C . C C C C C C C C C T C C C C C C C . C C C C C C C C C C C C C C C C C C C C C C C C C C C C C C C C C C C C C C C C C C C C C C C C C C C C C C C C C C C C C C C C . . . . . C C T C C C C C C C C C    
1115	. . . . . . . . . . . . . . . . . . . . . . . . . . . . . . . . . . . . . . T . T T T T T T T T T T T T T T T T T . T T T T T T T T T T T T T T T T T T T T T T T T T T T T T T T T T T T T T T T T T T T T T T T T T T T T T T T T T T T T T T T T . . . . . T T T T T T T T T T T T    
1116	. . . . . . . . . . . . . . . . . . . . . . . . . . . . . . . . . . . . . . T . T T T T T T T T T T T T T T T T T T T T T T T T T T T T T T T T T T T T T T T T T T T T T T T T T T T T T T T T T T T T T T T T T T T T T T T T T T T T T T T T T T . . . T . T T T T T T T T T T T T    
1117	. . . . . . . . . . . . . . . . . . . . . . . . . . . . . . . . . . . . . . A . A A A A A A A A A A A A A A A A A A A A A A A A A A A A A A A A A A A A A A A A A A A A A A A A A A A A A A A A A A A A A A A A A A A A A A A A A A A A A A A A A A . . . A . A A A A A A A A A A A A    
1118	. . . . . . . . . . . . . . . . . . . . . . . . . . . . . . . . . . . . . . A . A A A A A A A A A A A A A A A A A A A A A A A A A A A A A A A A A A A A A A A A A A A A A A A A A A A A A A A A A A A A A A A A A A A A A A A A A A A A A A A A A A . . . A . A A A A A A A A A A A A    
1119	. . . . . . . . . . . . . . . . . . . . . . . . . . . . . . . . . . . . . . A . A A A A A A A A A A A A A A A A A A A A A A A A A A A A A A A A A A A A A A A A A A A A A A A A A A A A A A A A A A A A A A A A A A A A A A A A A A A A A A A A A A . . . A . A A A A A A A A A A A A    
1120	. . . . . . . . . . . . . . . . . . . . . . . . . . . . . . . . . . . . . . G . G G G G G G G G G G G G G G G G G G G G G G G G G G G G G G G G G G G G G G G G G G G G G G G G G G G G G G G G G G G G G G G G G G G G G G G G G G G G G G G G G G . . . G . G G G G G G G G G G G G    
1121	. . . . . . . . . . . . . . . . . . . . . . . . . . . . . . . . . . . . . . G . G G G G G G G G G G G G G G G G G G G G G G G G G G G G G G G G G G G G G G G G G G G G G G G G G G G G G G G G G G G G G G G G G G G G G G G G G G G G G G G G G G . . . G . G G G G G G G G G G G G    
1122	. . . . . . . . . . . . . . . . . . . . . . . . . . . . . . . . . . . . . . A . A A A A A A A A A A A A A A A A A A A A A A A A A A A A A A A A A A A A A A A A A A A A A A A A A A A A A A A A A A A A A A A A A A A A A A A A A A A A A A A A A A . . . A . A A A A A A A A A A A A    
1123	. . . . . . . . . . . . . . . . . . . . . . . . . . . . . . . . . . . . . . . . A A A A A A A A A A A A A A A A A A A A A A A A A A A A A A A A A A A A A A A A A A A A A A A A A A A A A A A A A A A A A A A A A A A A A A A A A A A A A A A A A A . . . A . A A A A A A A A A A A A    
1124	. . . . . . . . . . . . . . . . . . . . . . . . . . . . . . . . . . . . . . . . C C C C C C C C C C C C C C C C C C C C C C C C C C C C C C C C C C C C C C C C C C C C C C C C C C C C C C C C C C C C C C C C C C C C C C C C C C C C C C C C C C . . . C . C C C C C C C C C C C C    
1125	. . . . . . . . . . . . . . . . . . . . . . . . . . . . . . . . . . . . . . . . A A A A A A A A A A A A A A A A A A A A A A A A A A A A A A A A A A A A A A A A A A A A A A A A A A A A A A A A A A A A A A A A A A A A A A A A A A A A A A A A A A . . . A . A A A A A A A A A A A A    
1126	. . . . . . . . . . . . . . . . . . . . . . . . . . . . . . . . . . . . . . . . G G G G G G G G G G G G G G G G G G G G G G G G G G G G G G G G G G G G G G G G G G G G G G G G G G G G G G G G G G G G G G G G G G G G G G G G G G G G G G G G G G . . . G . G G G G G G G G G G G G    
1127	. . . . . . . . . . . . . . . . . . . . . . . . . . . . . . . . . . . . . . . . C C C C C C C C C C C C C C C C C C C C C C C C C C C C C C C C C C C C C C C C C C C C C C C C C C C C C C C C C C C C C C C C C C C C C C C C C C C C C C C C C C . . . C C C C C C C C C C C C C C    
1128	. . . . . . . . . . . . . . . . . . . . . . . . . . . . . . . . . . . . . . . . G G G G G G G G G G G G G G G G G G G G G G G G G G G G G G G G G G G G G G G G G G G G G G G G G G G G G G G G G G G G G G G G G G G G G G G G G G G G G G G G G G . . . G G G G G G G G G G G G G G    
1129	. . . . . . . . . . . . . . . . . . . . . . . . . . . . . . . . . . . . . . . . G G G G G G G G G G G G G G G G G G G G G G G G G G G G G G G G G G G G G G G G G G G G G G G G G G G G G G G G G G G G G G G G G G G G G G G G G G G G G G G G G G . . . G G G G G G G G G G G G G G    
1130	. . . . . . . . . . . . . . . . . . . . . . . . . . . . . . . . . . . . . . . . A A A A A A A A A A A A A A A A A A A A A A A A A A A A A A A A A A A A A A A A A A A A A A A A A A A A A A A A A A A A A A A A A A A A A A A A A A A A A A A A A A . . . A A A A A A A A A A A A A A    
1131	. . . . . . . . . . . . . . . . . . . . . . . . . . . . . . . . . . . . . . . . A A A A A A A A A A A A A A A A A A A A A A A A A A A A A A A A A A A A A A A A A A A A A A A A A A A A A A A A A A A A A A A A A A A A A A A A A A A A A A A A A A . . . A A A A A A A A A A A A A A    
1132	. . . . . . . . . . . . . . . . . . . . . . . . . . . . . . . . . . . . . . . . T T T T T T T T T T T T T T T T T T T T T T T T T T T T T T T T T T T T T T T T T T T T T T T T T T T T T T T T T T T T T T T T T T T T T T T T T T T T T T T T T T . . . T T T T T T T T T T T T T T    
1133	. . . . . . . . . . . . . . . . . . . . . . . . . . . . . . . . . . . . . . . . A A A A A A A A A A A A A A A A A A A A A A A A A A A A A A A A A A A A A A A A A A A A A A A A A A A A A A A A A A A A A A A A A A A A A A A A A A A A A A A A A A . . . A A A A A A A A A A A A A A    
1134	. . . . . . . . . . . . . . . . . . . . . . . . . . . . . . . . . . . . . . . . T T T T T T T T T T T T T T T T T T T T T T T T T T T T T T T T T T T T T T T T T T T T T T T T T T T T T T T T T T T T T T T T T T T T T T T T T T T T T T T T T T . . . T T T T T T T T T T T T T T    
1135	. . . . . . . . . . . . . . . . . . . . . . . . . . . . . . . . . . . . . . . . G G G G G G G G G G G G G G G G G G G G G G G G G G G G G G G G G G G G G G G G G G G G G G G G G G G G G G G G G G G G G G G G G G G G G G G G G G G G G G G G G G . . . G G G G G G G G G G G G G G    
1136	. . . . . . . . . . . . . . . . . . . . . . . . . . . . . . . . . . . . . . . . T T T T T T T T T T T T T T T T T T T T T T T T T T T T T T T T T T T T T T T T T T T T T T T T T T T T T T T T T T T T T T T T T T T T T T T T T T T T T T T T T T . . . T T T T T T T T T T T T T T    
1137	. . . . . . . . . . . . . . . . . . . . . . . . . . . . . . . . . . . . . . . . T T T T T T T T T T T T T T T T T T T T T T T T T T T T T T T T T T T T T T T T T T T T T T T T T T T T T T T T T T T T T T T T T T T T T T T T T T T T T T T T T T . . . T T T T T T T T T T T T T T    
1138	. . . . . . . . . . . . . . . . . . . . . . . . . . . . . . . . . . . . . . . . C C C C C C C C C C C C C C C C C C C C C C C C C C C C C C C C C C C C C C C C C C C C C C C C C C C C C C C C C C C C C C C C C C C C C C C C C C C C C C C C C C . . . C C C C C C C C C C C C C C    
1139	. . . . . . . . . . . . . . . . . . . . . . . . . . . . . . . . . . . . . . . . A A A A A A A A A A A A A A A A A A A A A A A A A A A A A A A A A A A A A A A A A A A A A A A A A A A A A A A A A A A A A A A A A A A A A A A A A A A A A A A A A A . . . A A A A A A A A A A A A A A    
1140	. . . . . . . . . . . . . . . . . . . . . . . . . . . . . . . . . . . . . . . . G G G G G G G G G G G G G G G G G G G G G G G G G G G G G G G G G G G G G G G G G G G G G G G G G G G G G G G G G G G G G G G G G G G G G G G G G G G G G G G G G G . . . G G G G G G G G G G G G G G    
1141	. . . . . . . . . . . . . . . . . . . . . . . . . . . . . . . . . . . . . . . . T T T T T T T T T T T T T T T T T T T T T T T T T T T T T T T T T T T T T T T T T T T T T T T T T T T T T T T T T T T T T T T T T T T T T T T T T T T T T T T T T T . . . T T T T T T T T T T T T T T    
1142	. . . . . . . . . . . . . . . . . . . . . . . . . . . . . . . . . . . . . . . . A A A A A A A A A A A A A A A A A A A A A A A A A A A A A A A A A A A A A A A A A A A A A A A A A A A A A A A A A A A A A A A A A A A A A A A A A A A A A A A A A A . . . A A A A A A A A A A A A A A    
1143	. . . . . . . . . . . . . . . . . . . . . . . . . . . . . . . . . . . . . . . . A A A A A A A A A A A A A A A A A A A A A A A A A A A A A A A A A A A A A A A A A A A A A A A A A A A A A A A A A A A A A A A A A A A A A A A A A A A A A A A A A A . . . A A A A A A A A A A A A A A    
1144	. . . . . . . . . . . . . . . . . . . . . . . . . . . . . . . . . . . . . . . . A A A A A A A A A A A A A A A A A A A A A A A A A A A A A A A A A A A A A A A A A A A A A A A A A A A A A A A A A A A A A A A A A A A A A A A A A A A A A A A A A A . . . A A A A A A A A A A A A A A    
1145	. . . . . . . . . . . . . . . . . . . . . . . . . . . . . . . . . . . . . . . . T T T T T T T T T T T T T T T T T T T T T T T T T T T T T T T T T T T T T T T T T T T T T T T T T T T T T T T T T T T T T T T T T T T T T T T T T T T T T T T T T T . . . T T T T T T T T T T T T T T    
1146	. . . . . . . . . . . . . . . . . . . . . . . . . . . . . . . . . . . . . . . . A G G G G G G G G G G G G G G G G G G G G G G G G G G G G G G G G G G G G G G G G G G G G G G G G G G G G G G G G G G G G G G G G G G G G G G G G G G G G G G G G G . . . G G G G G G G G G G G G G G    
1147	. . . . . . . . . . . . . . . . . . . . . . . . . . . . . . . . . . . . . . . . . G G G G G G G G G G G G G G G G G G G G G G G G G G G G G G G G G G G G G G G G G G G G G G G G G G G G G G G G G G G G G G G G G G G G G G G G G G G G G G G G G . . . G G G G G G G G G G G G G G    
1148	. . . . . . . . . . . . . . . . . . . . . . . . . . . . . . . . . . . . . . . . . T T T T T T T T T T T T T T T T T T T T T T T T T T T T T T T T T T T T T T T T T T T T T T T T T T T T T T T T T T T T T T T T T T T T T T T T T T T T T T T T T . . . T T T T T T T T T T T T T T    
1149	. . . . . . . . . . . . . . . . . . . . . . . . . . . . . . . . . . . . . . . . . G G G G G G G G G G G G G G G G G G G G G G G G G G G G G G G G G G G G G G G G G G G G G G G G G G G G G G G G G G G G G G G G G G G G G G G G G G G G G G G G G . . . G G G G G G G G G G G G G G    
1150	. . . . . . . . . . . . . . . . . . . . . . . . . . . . . . . . . . . . . . . . . A A A A A A A A A A A A A A A A A A A A A A A A A A A A A A A A A A A A A A A A A A A A A A A A A A A A A A A A A A A A A A A A A A A A A A A A A A A A A A A A A . . . A A A A A A A A A A A A A A    
1151	. . . . . . . . . . . . . . . . . . . . . . . . . . . . . . . . . . . . . . . . . A A A A A A A A A A A A A A A A A A A A A A A A A A A A A A A A A A A A A A A A A A A A A A A A A A A A A A A A A A A A A A A A A A A A A A A A A A A A A A A A A . . . A A A A A A A A A A A A A A    
1152	. . . . . . . . . . . . . . . . . . . . . . . . . . . . . . . . . . . . . . . . . A A A A A A A A A A A A A A A A A A A A A A A A A A A A A A A A A A A A A A A A A A A A A A A A A A A A A A A A A A A A A A A A A A A A A A A A A A A A A A A A A . . . A A A A A A A A A A A A A A    
1153	. . . . . . . . . . . . . . . . . . . . . . . . . . . . . . . . . . . . . . . . . A A A A A A A A A A A A A A A A A A A A A A A A A A A A A A A A A A A A A A A A A A A A A A A A A A A A A A A A A A A A A A A A A A A A A A A A A A A A A A A A A . . . A A A A A A A A A A A A A A    
1154	. . . . . . . . . . . . . . . . . . . . . . . . . . . . . . . . . . . . . . . . . T T T T T T T T T T T T T T T T T T T T T T T T T T T T T T T T T T T T T T T T T T T T T T T T T T T T T T T T T T T T T T T T T T T T T T T T T T T T T T T T T . . . T T T T T T T T T T T T T T    
1155	. . . . . . . . . . . . . . . . . . . . . . . . . . . . . . . . . . . . . . . . . A A A A A A A A A A A A A A A A A A A A A A A A A A A A A A A A A A A A A A A A A A A A A A A A A A A A A A A A A A A A A A A A A A A A A A C A A A A A A A A A A . . . A A A A A A A A A A A A A A    
1156	. . . . . . . . . . . . . . . . . . . . . . . . . . . . . . . . . . . . . . . . . C C C C C C C C C C C C C C C C C C C C C C C C C C C C C C C C C C C C C C C C C C C C C C C C C C C C C C C C C C C C C C C C C C C C C C C C C C C C C C C C C . . . C C C C C C C C C C C C C C    
1157	. . . . . . . . . . . . . . . . . . . . . . . . . . . . . . . . . . . . . . . . . T T T T T T T T T T T T T T T T T T T T T T T T T T T T T T T T T T T T T T T T T T T T T T T T T T T T T T T T T T T T T T T T T T T T T T T T T T T T T T T T T . . . T T T T T T T T T T T T T T    
1158	. . . . . . . . . . . . . . . . . . . . . . . . . . . . . . . . . . . . . . . . . G G G G G G G G G G G G G G G G G G G G G G G G G G G G G G G G G G G G G G G G G G G G G G G G G G G G G G G G G G G G G G G G G G G G G G G G G G G G G G G G G . . . G G G G G G G G G G G G G G    
1159	. . . . . . . . . . . . . . . . . . . . . . . . . . . . . . . . . . . . . . . . . T T T T T T T T T T T T T T T T T T T T T T T T T T T T T T T T T T T T T T T T T T T T T T T T T T T T T T T T T T T T T T T T T T T T T T T T T T T T T T T T T . . . T T T T T T T T T T T T T T    
1160	. . . . . . . . . . . . . . . . . . . . . . . . . . . . . . . . . . . . . . . . . G G G G G G G G G G G G G G G G G G G G G G G G G G G G G G G G G G G G G G G G G G G G G G G G G G G G G G G G G G G G G G G G G G G G G G G G G G G G G G G G G . . . G G G G G G G G G G G G G G    
1161	. . . . . . . . . . . . . . . . . . . . . . . . . . . . . . . . . . . . . . . . . G G G G G G G G G G G G G G G G G G G G G G G G G G G G G G G G G G G G G G G G G G G G G G G G G G G G G G G G G G G G G G G G G G G G G G G G G G G G G G G G G . . . G G G G G G G G G G G G G G    
1162	. . . . . . . . . . . . . . . . . . . . . . . . . . . . . . . . . . . . . . . . . T T T T T T T T T T T T T T T T T T T T T T T T T T T T T T T T T T T T T T T T T T T T T T T T T T T T T T T T T T T T T T T T T T T T T T T T T T T T T T T T T . . . T T T T T T T T T T T T T T    
1163	. . . . . . . . . . . . . . . . . . . . . . . . . . . . . . . . . . . . . . . . . A A A A A A A A A A A A A A A A A A A A A A A A A A A A A A A A A A A A A A A A A A A A A A A A A A A A A A A A A A A A A A A A A A A A A A A A A A A A A A A A A . . . A A A A A A A A A A A A A A    
1164	. . . . . . . . . . . . . . . . . . . . . . . . . . . . . . . . . . . . . . . . . A A A A A A A A A A A A A A A A A A A A A A A A A A A A A A A A A A A A A A A A A A A A A A A A A A A A A A A A A A A A A A A A A A A A A A A A A A A A A A A A A . . . A A A A A A A A A A A A A A    
1165	. . . . . . . . . . . . . . . . . . . . . . . . . . . . . . . . . . . . . . . . . A A A A A A A A A A A A A A A A A A A A A A A A A A A A A A A A A A A A A A A A A A A A A A A A A A A A A A A A A A A A A A A A A A A A A A A A A A A A A A A A A . . . A A A A A A A A A A A A A A    
1166	. . . . . . . . . . . . . . . . . . . . . . . . . . . . . . . . . . . . . . . . . A A A A A A A A A A A A A A A A A A A A A A A A A A A A A A A A A A A A A A A A A A A A A A A A A A A A A A A A A A A A A A A A A A A A A A A A A A A A A A A A A . . . A A A A A A A A A A A A A A    
1167	. . . . . . . . . . . . . . . . . . . . . . . . . . . . . . . . . . . . . . . . . C C C C C C C C C C C C C C C C C C C C C C C C C C C C C C C C C C C C C C C C C C C C C C C C C C C C C C C C C C C C C C C C C C C C C C C C C C C C C C C C C . . . C C C C C C C C C C C C C C    
1168	. . . . . . . . . . . . . . . . . . . . . . . . . . . . . . . . . . . . . . . . . A A A A A A A A A A A A A A A A A A A A A A A A A A A A A A A A A A A A A A A A A A A A A A A A A A A A A A A A A A A A A A A A A A A A A A A A A A A A A A A A A . . . A A A A A A A A A A A A A A    
1169	. . . . . . . . . . . . . . . . . . . . . . . . . . . . . . . . . . . . . . . . . G G G G G G G G G G G G G G G G G G G G G G G G G G G G G G G G G G G G G G G G G G G G G G G G G G G G G G G G G G G G G G G G G G G G G G G G G G G G G G G G G . . . G G G G G G G G G G G G G G    
1170	. . . . . . . . . . . . . . . . . . . . . . . . . . . . . . . . . . . . . . . . . G G G G G G G G G G G G G G G G G G G G G G G G G G G G G G G G G G G G G G G G G G G G G G G G G G G G G G G G G G G G G G G G G G G G G G G G G G G G G G G G G . . . G G G G G G G G G G G G G G    
1171	. . . . . . . . . . . . . . . . . . . . . . . . . . . . . . . . . . . . . . . . . T T T T T T T T T T T T T T T T T T T T T T T T T T T T T T T T T T T T T T T T T T T T T T T T T T T T T T T T T T T T T T T T T T T T T T T T T T T T T T T T T . . . T T T T T T T T T T T T T T    
1172	. . . . . . . . . . . . . . . . . . . . . . . . . . . . . . . . . . . . . . . . . T T T T T T T T T T T T T T T T T T T T T T T T T T T T T T T T T T T T T T T T T T T T T T T T T T T T T T T T T T T T T T T T T T T T T T T T T T T T T T T T T . . . T T T T T T T T T T T T T T    
1173	. . . . . . . . . . . . . . . . . . . . . . . . . . . . . . . . . . . . . . . . . T T T T T T T T T T T T T T T T T T T T T T T T T T T T T T T T T T T T T T T T T T T T T T T T T T T T T T T T T T T T T T T T T T T T T T T T T T T T T T T T T . . . T T T T T T T T T T T T T T    
1174	. . . . . . . . . . . . . . . . . . . . . . . . . . . . . . . . . . . . . . . . . T T T T T T T T T T T T T T T T T T T T T T T T T T T T T T T T T T T T T T T T T T T T T T T T T T T T T T T T T T T T T T T T T T T T T T T T T T T T T T T T T . . . T T T T T T T T T T T T T T    
1175	. . . . . . . . . . . . . . . . . . . . . . . . . . . . . . . . . . . . . . . . . G G G G G G G G G G G G G G G G G G G G G G G G G G G G G G G G G G G G G G G G G G G G G G G G G G G G G G G G G G G G G G G G G G G G G G G G G G G G G G G G G . . . G G G G G G G G G G G G G G    
1176	. . . . . . . . . . . . . . . . . . . . . . . . . . . . . . . . . . . . . . . . . A A A A A A A A A A A A A A A A A A A A A A A A A A A A A A A A A A A A A A A A A A A A A A A A A A A A A A A A A A A A A A A A A A A A A A A A A A A A A A A A A . . . A A A A A A A A A A A A A A    
1177	. . . . . . . . . . . . . . . . . . . . . . . . . . . . . . . . . . . . . . . . . G G G G G G G G G G G G G G G G G G G G G G G G G G G G G G G G G G G G G G G G G G G G G G G G G G G G G G G G G G G G G G G G G G G G G G G G G G G G G G G G G . . . G G G G G G G G G G G G G G    
1178	. . . . . . . . . . . . . . . . . . . . . . . . . . . . . . . . . . . . . . . . . A A A A A A A A A A A A A A A A A A A A A A A A A A A A A A A A A A A A A A A A A A A A A A A A A A A A A A A A A A A A A A A A A A A A A A A A A A A A A A A A A . . . A A A A A A A A A A A A A A    
1179	. . . . . . . . . . . . . . . . . . . . . . . . . . . . . . . . . . . . . . . . . G G G G G G G G G G G G G G G G G G G G G G G G G G G G G G G G G G G G G G G G G G G G G G G G G G G G G G G G G G G G G G G G G G G G G G G G G G G G G G G G G . . . G G G G G G G G G G G G G G    
1180	. . . . . . . . . . . . . . . . . . . . . . . . . . . . . . . . . . . . . . . . . A A A A A A A A A A A A A A A A A A A A A A A A A A A A A A A A A A A A A A A A A A A A A A A A A A A A A A A A A A A A A A A A A A A A A A A A A A A A A A A A A . . . A A A A A A A A A A A A A A    
1181	. . . . . . . . . . . . . . . . . . . . . . . . . . . . . . . . . . . . . . . . . C C C C C C C C C C C C C C C C C C C C C C C C C C C C C C C C C C C C C C C C C C C C C C C C C C C C C C C C C C C C C C C C C C C C C C C C C C C C C C C C C . . . C C C C C C C C C C C C C C    
1182	. . . . . . . . . . . . . . . . . . . . . . . . . . . . . . . . . . . . . . . . . G G G G G G G G G G G G G G G G G G G G G G G G G G G G G G G G G G G G G G G G G G G G G G G G G G G G G G G G G G G G G G G G G G G G G G G G G G G G G G G G G . . . G G G G G G G G G G G G G G    
1183	. . . . . . . . . . . . . . . . . . . . . . . . . . . . . . . . . . . . . . . . . C C C C C C C C C C C C C C C C C C C C C C C C C C C C C C C C C C C C C C C C C C C C C C C C C C C C C C C C C C C C C C C C C C C C C C C C C C C C C C C C C . . . C C C C C C C C C C C C C C    
1184	. . . . . . . . . . . . . . . . . . . . . . . . . . . . . . . . . . . . . . . . . T T T T T T T T T T T T T T T T T T T T T T T T T T T T T T T T T T T T T T T T T T T T T T T T T T T T T T T T T T T T T T T T T T T T T T T T T T T T T T T T T . . . T T T T T T T T T T T T T T    
1185	. . . . . . . . . . . . . . . . . . . . . . . . . . . . . . . . . . . . . . . . . G G G G G G G G G G G G G G G G G G G G G G G G G G G G G G G G G G G G G G G G G G G G G G G G G G G G G G G G G G G G G G G G G G G G G G G G G G G G G G G G G . . . G G G G G G G G G G G G G G    
1186	. . . . . . . . . . . . . . . . . . . . . . . . . . . . . . . . . . . . . . . . . G G G G G G G G G G G G G G G G G G G G G G G G G G G G G G G G G G G G G G G G G G G G G G G G G G G G G G G G G G G G G G G G G G G G G G G G G G G G G G G G G . . . G G G G G G G G G G G G G G    
1187	. . . . . . . . . . . . . . . . . . . . . . . . . . . . . . . . . . . . . . . . . T T T T T T T T T T T T T T T T T T T T T T T T T T T T T T T T T T T T T T T T T T T T T T T T T T T T T T T T T T T T T T T T T T T T T T T T T T T T T T T T T . . . T T T T T T T T T T T T T T    
1188	. . . . . . . . . . . . . . . . . . . . . . . . . . . . . . . . . . . . . . . . . T T T T T T T T T T T T T T T T T T T T T T T T T T T T T T T T T T T T T T T T T T T T T T T T T T T T T T T T T T T T T T T T T T T T T T T T T T T T T T T T T . . . T T T T T T T T T T T T T T    
1189	. . . . . . . . . . . . . . . . . . . . . . . . . . . . . . . . . . . . . . . . . A A A A A A A A A A A A A A A A A A A A A A A A A A A A A A A A A A A A A A A A A A A A A A A A A A A A A A A A A A A A A A A A A A A A A A A A A A A A A A A A A . . . A A A A A A A A A A A A A A    
1190	. . . . . . . . . . . . . . . . . . . . . . . . . . . . . . . . . . . . . . . . . A A A A A A A A A A A A A A A A A A A A A A A A A A A A A A A A A A A A A A A A A A A A A A A A A A A A A A A A A A A A A A A A A A A A A A A A A A A A A A A A A . . . A A A A A A A A A A A A A A    
1191	. . . . . . . . . . . . . . . . . . . . . . . . . . . . . . . . . . . . . . . . . T T T T T T T T T T T T T T T T T T T T T T T T T T T T T T T T T T T T T T T T T T T T T T T T T T T T T T T T T T T T T T T T T T T T T T T T T T T T T T T T T . . . T T T T T T T T T T T T T T    
1192	. . . . . . . . . . . . . . . . . . . . . . . . . . . . . . . . . . . . . . . . . G G G G G G G G G G G G G G G G G G G G G G G G G G G G G G G G G G G G G G G G G G G G G G G G G G G G G G G G G G G G G G G G G G G G G G G G G G G G G G G G G . . . G G G G G G G G G G G G G G    
1193	. . . . . . . . . . . . . . . . . . . . . . . . . . . . . . . . . . . . . . . . . A A A A A A A A A A A A A A A A A A A A A A A A A A A A A A A A A A A A A A A A A A A A A A A A A A G A A A A A A A A A A A A A A A A A A A A A A A A A A A A A A . . . A A A A A A A A A A A A A A    
1194	. . . . . . . . . . . . . . . . . . . . . . . . . . . . . . . . . . . . . . . . . G G G G G G G G G G G G G G G G G G G G G G G G G G G G G G G G G G G G G G G G G G G G G G G G G G G G G G G G G G G G G G G G G G G G G G G G G G G G G G G G G . . . G G G G G G G G G G G G G G    
1195	. . . . . . . . . . . . . . . . . . . . . . . . . . . . . . . . . . . . . . . . . A A A A A A A A A A A A A A A A A A A A A A A A A A A A A A A A A A A A A A A A A A A A A A A A A A A A A A A A A A A A A A A A A A A A A A A A A A A A A A A A A . . . A A A A A A A A A A A A A A    
1196	. . . . . . . . . . . . . . . . . . . . . . . . . . . . . . . . . . . . . . . . . A A A A A A A A A A A A A A A A A A A A A A A A A A A A A A A A A A A A A A A A A A A A A A A A A A A A A A A A A A A A A A A A A A A A A A A A A A A A A A A A A . . . A A A A A A A A A A A A A A    
1197	. . . . . . . . . . . . . . . . . . . . . . . . . . . . . . . . . . . . . . . . . T T T T T T T T T T T T T T T T T T T T T T T T T T T T T T T T T T T T T T T T T T T T T T T T T T T T T T T T T T T T T T T T T T T T T T T T T T T T T T T T T . . . T T T T T T T T T T T T T T    
1198	. . . . . . . . . . . . . . . . . . . . . . . . . . . . . . . . . . . . . . . . . A A A A A A A A A A A A A A A A A A A A A A A A A A A A A A A A A A A A A A A A A A A A A A A A A A A A A A A A A A A A A A A A A A A A A A A A A A A A A A A A A . . . A A A A A A A A A A A A A A    
1199	. . . . . . . . . . . . . . . . . . . . . . . . . . . . . . . . . . . . . . . . . G G G G G G G G G G G G G G G G G G G G G G G G G G G G G G G G G G G G G G G G G G G G G G G G G G G G G G G G G G G G G G G G G G G G G G G G G G G G G G G G G . . . G G G G G G G G G G G G G G    
1200	. . . . . . . . . . . . . . . . . . . . . . . . . . . . . . . . . . . . . . . . . A A A A A A A A A A A A A A A A A A A A A A A A A A A A A A A A A A A A A A A A A A A A A A A A A A A A A A A A A A A A A A A A A A A A A A A A A A A A A A A A A . . . A A A A A A A A A A A A A A    
1201	. . . . . . . . . . . . . . . . . . . . . . . . . . . . . . . . . . . . . . . . . T T T T T T T T T T T T T T T T T T T T T T T T T T T T T T T T T T T T T T T T T T T T T T T T T T T T T T T T T T T T T T T T T T T T T T T T T T T T T T T T T . . . T T T T T T T T T T T T T T    
1202	. . . . . . . . . . . . . . . . . . . . . . . . . . . . . . . . . . . . . . . . . T T T T T T T T T T T T T T T T T T T T T T T T T T T T T T T T T T T T T T T T T T T T T T T T T T T T T T T T T T T T T T T T T T T T T T T T T T T T T T T T T . . . T T T T T T T T T T T T T T    
1203	. . . . . . . . . . . . . . . . . . . . . . . . . . . . . . . . . . . . . . . . . C C C C C C C C C C C C C C C C C C C C C C C C C C C C C C C C C C C C C C C C C C C C C C C C C C C C C C C C C C C C C C C C C C C C C C C C C C C C C C C C C . . . C C C C C C C C C C C C C C    
1204	. . . . . . . . . . . . . . . . . . . . . . . . . . . . . . . . . . . . . . . . . A A A A A A A A A A A A A A A A A A A A A A A A A A A A A A A A A A A A A A A A A A A A A A A A A A A A A A A A A A A A A A A A A A A A A A A A A A A A A A A A A . . . A A A A A A A A A A A A A A    
1205	. . . . . . . . . . . . . . . . . . . . . . . . . . . . . . . . . . . . . . . . . G G G G G G G G G G G G G G G G G G G G G G G G G G G G G G G G G G G G G G G G G G G G G G G G G G G G G G G G G G G G G G G G G G G G G G G G G G G G G G G G G . . . G G G G G G G G G G G G G G    
1206	. . . . . . . . . . . . . . . . . . . . . . . . . . . . . . . . . . . . . . . . . T T T T T T T T T T T T T T T T T T T T T T T T T T T T T T T T T T T T T T T T T T T T T T T T T T T T T T T T T T T T T T T T T T T T T T T T T T T T T T T T T . . . T T T T T T T T T T T T T T    
1207	. . . . . . . . . . . . . . . . . . . . . . . . . . . . . . . . . . . . . . . . . C C C C C C C C C C C C C C C C C C C C C C C C C C C C C C C C C C C C C C C C C C C C C C C C C C C C C C C C C C C C C C C C C C C C C C C C C C C C C C C C C . . . C C C C C C C C C C C C C C    
1208	. . . . . . . . . . . . . . . . . . . . . . . . . . . . . . . . . . . . . . . . . T T T T T T T T T T T T T T T T T T T T T T T T T T T T T T T T T T T T T T T T T T T T T T T T T T T T T T T T T T T T T T T T T T T T T T T T T T T T T T T T T . . . T T T T T T T T T T T T T T    
1209	. . . . . . . . . . . . . . . . . . . . . . . . . . . . . . . . . . . . . . . . . T T T T T T T T T T T T T T T T T T T T T T T T T T T T T T T T T T T T T T T T T T T T T T T T T T T T T T T T T T T T T T T T T T T T T T T T T T T T T T T T T . . . T T T T T T T T T T T T T T    
1210	. . . . . . . . . . . . . . . . . . . . . . . . . . . . . . . . . . . . . . . . . T T T T T T T T T T T T T T T T T T T T T T T T T T T T T T T T T T T T T T T T T T T T T T T T T T T T T T T T T T T T T T T T T T T T T T T T T T T T T T T T T . . . T T T T T T T T T T T T T T    
1211	. . . . . . . . . . . . . . . . . . . . . . . . . . . . . . . . . . . . . . . . . G G G G G G G G G G G G G G G G G G G G G G G G G G G G G G G G G G G G G G G G G G G G G G G G G G G G G G G G G G G G G G G G G G G G G G G G G G G G G G G G G . . . G G G G G G G G G A A G G G    
1212	. . . . . . . . . . . . . . . . . . . . . . . . . . . . . . . . . . . . . . . . . A A A A A A A A A A A A A A A A A A A A A A A A A A A A A A A A A A A A A A A A A A A A A A A A A A A A A A A A A A A A A A A A A A A A A A A A A A A A A A A A A . . . A A A A A A A A A A A A A A    
1213	. . . . . . . . . . . . . . . . . . . . . . . . . . . . . . . . . . . . . . . . . T T T T T T T T T T T T T T T T T T T T T T T T T T T T T T T T T T T T T T T T T T T T T T T T T T T T T T T T T T T T T T T T T T T T T T T T T T T T T T T T T . . . T T T T T T T T T T T T T T    
1214	. . . . . . . . . . . . . . . . . . . . . . . . . . . . . . . . . . . . . . . . . T T T T T T T T T T T T T T T T T T T T T T T T T T T T T T T T T T T T T T T T T T T T T T T T T T T T T T T T T T T T T T T T T T T T T T T T T T T T T T T T T . . . T T T T T T T T T T T T T T    
1215	. . . . . . . . . . . . . . . . . . . . . . . . . . . . . . . . . . . . . . . . . T T T T T T T T T T T T T T T T T T T T T T T T T T T T T T T T T T T T T T T T T T T T T T T T T T T T T T T T T T T T T T T T T T T T T T T T T T T T T T T T T . . . T T T T T T T T T T T T T T    
1216	. . . . . . . . . . . . . . . . . . . . . . . . . . . . . . . . . . . . . . . . . T C C C C C C C C C C C C C C C C C C C C C C C C C C C C C C C C C C C C C C C C C C C C C C C C C C C C C C C C C C C C C C C C C C C C C C C C C C C C C C C C . . . C C C C C C C C C C C C C C    
1217	. . . . . . . . . . . . . . . . . . . . . . . . . . . . . . . . . . . . . . . . . . A A A A A A A A A A A A A A A A A A A A A A A A A A A A A A A A A A A A A A A A A A A A A A A A A A A A A A A A A A A A A A A A A A A A A A A A A A A A A A A A . . . A A A A A A A A A A A A A A    
1218	. . . . . . . . . . . . . . . . . . . . . . . . . . . . . . . . . . . . . . . . . . T T T T T T T T T T T T T T T T T T T T T T T T T T T T T T T T T T T T T T T T T T T T T T T T T T T T T T T T T T T T T T T T T T T T T T T T T T T T T T T T . . . T T T T T T T T T T T T T T    
1219	. . . . . . . . . . . . . . . . . . . . . . . . . . . . . . . . . . . . . . . . . . T T T T T T T T T T T T T T T T T T T T T T T T T T T T T T T T T T T T T T T T T T T T T T T T T T T T T T T T T T T T T T T T T T T T T T T T T T T T T T T T . . . T T T T T T T T T T T T T T    
1220	. . . . . . . . . . . . . . . . . . . . . . . . . . . . . . . . . . . . . . . . . . T T T T T T T T T T T T T T T T T T T T T T T T T T T T T T T T T T T T T T T T T T T T T T T T T T T T T T T T T T T T T T T T T T T T T T T T T T T T T T T T . . . T T T T T T T T T T T T T T    
1221	. . . . . . . . . . . . . . . . . . . . . . . . . . . . . . . . . . . . . . . . . . C C C C C C C T C C C C C C C T C C C C C C C C C C C C C C C C C C C C C C C C C C C C C C C C C C C C C C C C C C C C C C C C C C C C C C C C C C C C C C C C . . . C C C C C C C C C C C C C C    
1222	. . . . . . . . . . . . . . . . . . . . . . . . . . . . . . . . . . . . . . . . . . T T T T T T T T T T T T T T T T T T T T T T T T T T T T T T T T T T T T T T T T T T T T T T T T T T T T T T T T T T T T T T T T T T T T T T T T T T T T T T T T . . . T T T T T T T T T T T T T T    
1223	. . . . . . . . . . . . . . . . . . . . . . . . . . . . . . . . . . . . . . . . . . T T T T T T T T T T T T T T T T T T T T T T T T T T T T T T T T T T T T T T T T T T T T T T T T T T T T T T T T T T T T T T T T T T T T T T T T T T T T T T T T . . . T T T T T T T T T T T T T T    
1224	. . . . . . . . . . . . . . . . . . . . . . . . . . . . . . . . . . . . . . . . . . C C C C C C C C C C C C C C C C C C C C C C C C C C C C C C C C C C C C C C C C C C C C C C C C C C C C C C C C C C C C C C C C C C C C C C C C C C C C C C C C . . . C C C C C C C C C C C C C C    
1225	. . . . . . . . . . . . . . . . . . . . . . . . . . . . . . . . . . . . . . . . . . A A A A A A A A A A A A A A A A A A A A A A A A A A A A A A A A A A A A A A A A A A A A A A A A A A A A A A A A A A A A A A A A A A A A A A A A A A A A A A A A . . . A A A A A A A A A A A A A A    
1226	. . . . . . . . . . . . . . . . . . . . . . . . . . . . . . . . . . . . . . . . . . T T T T T T T T T T T T T T T T T T T T T T T T T T T T T T T T T T T T T T T T T T T T T T T T T T T T T T T T T T T T T T T T T T T T T T T T T T T T T T T T . . . T T T T T T T T T T T T T T    
1227	. . . . . . . . . . . . . . . . . . . . . . . . . . . . . . . . . . . . . . . . . . G G G G G G G G G G G G G G G G G G G G G G G G G G G G G G G G G G G G G G G G G G G G G G G G G G G G G G G G G G G G G G G G G G G G G G G G G G G G G G G G . . . G G G G G G G G G G G G G G    
1228	. . . . . . . . . . . . . . . . . . . . . . . . . . . . . . . . . . . . . . . . . . T T T T T T T T T T T T T T T T T T T T T T T T T T T T T T T T T T T T T T T T T T T T T T T T T T T T T T T T T T T T T T T T T T T T T T T T T T T T T T T T . . . T T T T T T T T T T T T T T    
1229	. . . . . . . . . . . . . . . . . . . . . . . . . . . . . . . . . . . . . . . . . . T T T T T T T T T T T T T T T T T T T T T T T T T T T T T T T T T T T T T T T T T T T T T T T T T T T T T T T T T T T T T T T T T T T T T T T T T T T T T T T T . . . T T T T T T T T T T T T T T    
1230	. . . . . . . . . . . . . . . . . . . . . . . . . . . . . . . . . . . . . . . . . . T T T T T T T T T T T T T T T T T T T T T T T T T T T T T T T T T T T T T T T T T T T T T T T T T T T T T T T T T T T T T T T T T T T T T T T T T T T T T T T T . . . T T T T T T T T T T T T T T    
1231	. . . . . . . . . . . . . . . . . . . . . . . . . . . . . . . . . . . . . . . . . . T T T T T T T T T T T T T T T T T T T T T T T T T T T T T T T T T T T T T T T T T T T T T T T T T T T T T T T T T T T T T T T T T T T T T T T T T T T T T T T T . . . T T T T T T T T T T T T T T    
1232	. . . . . . . . . . . . . . . . . . . . . . . . . . . . . . . . . . . . . . . . . . A A A A A A A A A A A A A A A A A A A A A A A A A A A A A A A A A A A A A A A A A A A A A A A A A A A A A A A A A A A A A A A A A A A A A A A A A A A A A A A A . . . A A A A A A A A A A A A A A    
1233	. . . . . . . . . . . . . . . . . . . . . . . . . . . . . . . . . . . . . . . . . . G G G G G G G G G G G G G G G G G G G G G G G G G G G G G G G G G G G G G G G G G G G G G G G G G G G G G G G G G G G G G G G G G G G G G G G G G G G G G G G G . . . G G G G G G G G G G G G G G    
1234	. . . . . . . . . . . . . . . . . . . . . . . . . . . . . . . . . . . . . . . . . . A A A A A A A A A A A A A A A A A A A A A A A A A A A A A A A A A A A A A A A A A A A A A A A A A A A A A A A A A A A A A A A A A A A A A A A A A A A A A A A A . . . A A A A A A A A A A A A A A    
1235	. . . . . . . . . . . . . . . . . . . . . . . . . . . . . . . . . . . . . . . . . . C C C C C C C C C C C C C C C C C C C C C C C C C C C C C C C C C C C C C C C C C C C C C C C C C C C C C C C C C C C C C C C C C C C C C C C C C C C C C C C C . . . C C C C C C C C C C C C C C    
1236	. . . . . . . . . . . . . . . . . . . . . . . . . . . . . . . . . . . . . . . . . . T T T T T T T T T T T T T T T T T T T T T T T T T T T T T T T T T T T T T T T T T T T T T T T T T T T T T T T T T T T T T T T T T T T T T T T T T T T T T T T T . . . T T T T T T T T T T T T T T    
1237	. . . . . . . . . . . . . . . . . . . . . . . . . . . . . . . . . . . . . . . . . . C T T T T T T T T T T T T T T T T T T T T T T T T T T T T T T T T T T T T T T T T T T T T T T T T T T T T T T T T T T T T T T T T T T T T T T T T T T T T T T T . . . T T T T T T T T T T T T T T    
1238	. . . . . . . . . . . . . . . . . . . . . . . . . . . . . . . . . . . . . . . . . . . T T T T T T T T T T T T T T T T T T T T T T T T T T T T T T T T T T T T T T T T T T T T T T T T T T T T T T T T T T T T T T T T T T T T T T T T T T T T T T T . . . T T T T T T T T T T T T T T    
1239	. . . . . . . . . . . . . . . . . . . . . . . . . . . . . . . . . . . . . . . . . . . A A A A A A A A A A A A A A A A A A A A A A A A A A A A A A A A A A A A A A A A A A A A A A A A A A A A A A A A A A A A A A A A A A A A A A A A A A A A A A A . . . A A A A A A A A A A A A A A    
1240	. . . . . . . . . . . . . . . . . . . . . . . . . . . . . . . . . . . . . . . . . . . A A A A A A A A A A A A A A A A A A A A A A A A A A A A A A A A A A A A A A A A A A A A A A A A A A A A A A A A A A A A A A A A A A A A A A A A A G A A A A A . . . A A A A A A A A A A A A A A    
1241	. . . . . . . . . . . . . . . . . . . . . . . . . . . . . . . . . . . . . . . . . . . A A A A A A A A A A A A A A A A A A A A A A A A A A A A A A A A A A A A A A A A A A A A A A A A A A A A A A A A A A A A A A A A A A A A A A A A A A A A A A A . . . A A A A A A A A A A A A A A    
1242	. . . . . . . . . . . . . . . . . . . . . . . . . . . . . . . . . . . . . . . . . . . A A A A A A A A A A A A A A A A A A A A A A A A A A A A A A A A A A A A A A A A A A A A A A A A A A A A A A A A A A A A A A A A A A A A A A A A A A A A A A A . . . A A A A A A A A A A A A A A    
1243	. . . . . . . . . . . . . . . . . . . . . . . . . . . . . . . . . . . . . . . . . . . G G G G G G G G G G G G G G G G G G G G G G G G G G G G G G G G G G G G G G G G G G G G G G G G G G G G G G G G G G G G G G G G G G G G G G G G G G G G G G G . . . G G G G G G G G G G G G G G    
1244	. . . . . . . . . . . . . . . . . . . . . . . . . . . . . . . . . . . . . . . . . . . T T T T T T T T T T T T T T T T T T T T T T T T T T T T T T T T T T T T T T T T T T T T T T T T T T T T T T T T T T T T T T T T T T T T T T T T T T T T T T T . . . T T T T T T T T T T T T T T    
1245	. . . . . . . . . . . . . . . . . . . . . . . . . . . . . . . . . . . . . . . . . . . C C C C C C C C C C C C C C C C C C C C C C C C C C C C C C C C C C C C C C C C C C C C C C C C C C C C C C C C C C C C C C C C C C C C C C C C C C C C C C C . . . C C C C C C C C C C C C C C    
1246	. . . . . . . . . . . . . . . . . . . . . . . . . . . . . . . . . . . . . . . . . . . C C C C C C C C C C C C C C C C C C C C C C C C C C C C C C C C C C C C C C C C C C C C C C C C C C C C C C C C C C C C C C C C C C C C C C C C C C C C C C C . . . C C C C C C C C C C C C C C    
1247	. . . . . . . . . . . . . . . . . . . . . . . . . . . . . . . . . . . . . . . . . . . C C C C C C C C C C C C C C C C C C C C C C C C C C C C C C C C C C C C C C C C C C C C C C C C C C C C C C C C C C C C C C C C C C C C C C C C C C C C C C C . . . C C C C C C C C C C C C C C    
1248	. . . . . . . . . . . . . . . . . . . . . . . . . . . . . . . . . . . . . . . . . . . C C C C C C C C C C C C C C C C C C C C C C C C C C C C C C C C C C C C C C C C C C C C C C C C C C C C C C C C C C C C C C C C C C C C C C C C C C C C C C C . . . C C C C C C C C C C C C C C    
1249	. . . . . . . . . . . . . . . . . . . . . . . . . . . . . . . . . . . . . . . . . . . T T T T T T T T T T T T T T T T T T T T T T T T T T T T T T T T T T T T T T T T T T T T T T T T T T T T T T T T T T T T T T T T T T T T T T T T T T T T T T T . . . T T T T T T T T T T T T T T    
1250	. . . . . . . . . . . . . . . . . . . . . . . . . . . . . . . . . . . . . . . . . . . G G G G G G G G G G G G G G G G G G G G G G G G G G G G G G G G G G G G G G G G G G G G G G G G G G G G G G G G G G G G G G G G G G G G G G G G G G G G G G G . . . G G G G G G G G G G G G G G    
1251	. . . . . . . . . . . . . . . . . . . . . . . . . . . . . . . . . . . . . . . . . . . T T T T T T T T T T T T T T T T T T T T T T T T T T T T T T T T T T T T T T T T T T T T T T T T T T T T T T T T T T T T T T T T T T T T T T T T T T T T T T T . . . T T T T T T T T T T T T T T    
1252	. . . . . . . . . . . . . . . . . . . . . . . . . . . . . . . . . . . . . . . . . . . A A A A A A A A A A A A A A A A A A A A A A A A A A A A A A A A A A A A A A A A A A A A A A A A A A A A A A A A A A A A A A A A A A A A A A A A A A A A A A A . A . A A A A A A A A A A A A A A    
1253	. . . . . . . . . . . . . . . . . . . . . . . . . . . . . . . . . . . . . . . . . . . C C C C C C C C C C C C C C C C C C C C C C C C C C C C C C C C C C C C C C C C C C C C C C C C C C C C C C C C C C C C C C C C C C C C C C C C C C C C C C C . C C C C C C C C C C C C C C C C    
1254	. . . . . . . . . . . . . . . . . . . . . . . . . . . . . . . . . . . . . . . . . . . T T T T T T T T T T T T T T T T T T T T T T T T T T T T T T T T T T T T T T T T T T T T T T T T T T T T T T T T T T T T T T T T T T T T T T T T T T T T T T T . T T T T T T T T T T T T T T T T    
1255	. . . . . . . . . . . . . . . . . . . . . . . . . . . . . . . . . . . . . . . . . . . G G G G G G G G G G G G G G G G G G G G G G G G G G G G G G G G G G G G G G G G G G G G G G G G G G G G G G G G G G G G G G G G G G G G G G G G G G G G G G G . G G G G G G G G G G G G G G G G    
1256	. . . . . . . . . . . . . . . . . . . . . . . . . . . . . . . . . . . . . . . . . . . C C C C C C C C C C C C C C C C C C C C C C C C C C C C C C C C C C C C C C C C C C C C C C C C C C C C C C C C C A A A C C C C C C C C C C C C C C C C C C C . C C C C C C C C C C C C C C C C    
1257	. . . . . . . . . . . . . . . . . . . . . . . . . . . . . . . . . . . . . . . . . . . C C C C C C C C C C C C C C C C C C C C C C C C C C C C C C C C C C C C C C C C C C C C C C C C C C C C C C C C C C C C C C C C C C C C C C C C C C C C C C C . C C C C C C C C C C C C C C C C    
1258	. . . . . . . . . . . . . . . . . . . . . . . . . . . . . . . . . . . . . . . . . . . A A A A A A A A A A A A A A A A A A A A A A A A A A A A A A A A A A A A A A A A A A A A A A A A A A A A A A A A A A A A A A A A A A A A A A A A A A A A A A A . A A A A A A A A A A A A A A A A    
1259	. . . . . . . . . . . . . . . . . . . . . . . . . . . . . . . . . . . . . . . . . . . T T T T T T T T T T T T T T T T T T T T T T T T T T T T T T T T T T T T T T T T T T T T T T T T T T T T T T T T T T T T T T T T T T T T T T T T T T T T T T T . T T T T T T T T T T T T T T T T    
1260	. . . . . . . . . . . . . . . . . . . . . . . . . . . . . . . . . . . . . . . . . . . C C C C C C C C C C C C C C C C C C C C C C C C C C C C C C C C C C C C C C C C C C C C C C C C C C C C C C C C C C C C C C C C C C C C C C C C C C C C C C C . C C C C C C C C C C C C C C C C    
1261	. . . . . . . . . . . . . . . . . . . . . . . . . . . . . . . . . . . . . . . . . . . A A A A A A A A A A A A A A A A A A A A A A A A A A A A A A A A A A A A A A A A A A A A A A A A A A A A A A A A A A A A A A A A A A A A A A A A A A A A A A A . A A A A A A A A A A A A A A A A    
1262	. . . . . . . . . . . . . . . . . . . . . . . . . . . . . . . . . . . . . . . . . . . C C C C C C C C C C C C C C C C C C C C C C C C C C C C C C C C C C C C C C C C C C C C C C C C C C C C C C C C C C C C C C C C C C C C C C C C C C C C C C C . C C C C C C C C C C C C C C C C    
1263	. . . . . . . . . . . . . . . . . . . . . . . . . . . . . . . . . . . . . . . . . . . C C C C C C C C C C C C C C C C C C C C C C C C C C C C C C C C C C C C C C C C C C C C C C C C C C C C C C C C C C C C C C C C C C C C C C C C C C C C C C C . C C C C C C C C C C C C C C C C    
1264	. . . . . . . . . . . . . . . . . . . . . . . . . . . . . . . . . . . . . . . . . . . - - - - - - - - - - - - - - - - - - - - - - - - - - - - - - - - - - - - - - - - - - - - - - - - - - - - - - - - - C C C - - - - - - - - - - - - - - - - - - - . - - - - - - - - - - - - - - - -    
1265	. . . . . . . . . . . . . . . . . . . . . . . . . . . . . . . . . . . . . . . . . . . - - - - - - - - - - - - - - - - - - - - - - - - - - - - - - - - - - - - - - - - - - - - - - - - - - - - - - - - - T T T - - - - - - - - - - - - - - - - - - - . - - - - - - - - - - - - - - - -    
1266	. . . . . . . . . . . . . . . . . . . . . . . . . . . . . . . . . . . . . . . . . . . T T T T T T T T T C T T T T T T T T T T T T T T T T T T T T T T T T T T T T T T T T T T T T T T T T T T T T T T T C C C C T T T T T T T T T T T T T T T T T T . T T T T T T T T T T T T T T T T    
1267	. . . . . . . . . . . . . . . . . . . . . . . . . . . . . . . . . . . . . . . . . . . C C C C C C C C C C C C C C C C C C C C C C C C C C C C C C C C C C C C C C C C C C C C C C C C C C C C C C C C C C C C C C C C C C C C C C C C C C C C C C C . C C C C C C C C C C C C C C C C    
1268	. . . . . . . . . . . . . . . . . . . . . . . . . . . . . . . . . . . . . . . . . . . T T T T T T T T T T T T T T T T T T T T T T T T T T T T T T T T T T T T T T T T T T T T T T T T T T T T T T T T T T T T T T T T T T T T T T T T T T T T T T T . T T T T T T T T T T T T T T T T    
1269	. . . . . . . . . . . . . . . . . . . . . . . . . . . . . . . . . . . . . . . . . . . G G G G G G G G G G G G G G G G G G G G G G G G G G G G G G G G G G G G G G G G G G G G G G G G G G G G G G G G G G G G G G G G G G G G G G G G G G G G G G G . G G G G G G G G G G G G G G G G    
1270	. . . . . . . . . . . . . . . . . . . . . . . . . . . . . . . . . . . . . . . . . . . G G G G G G G G G G G G G G G G G G G G G G G G G G G G G G G G G G G G G G G G G G G G G G G G G G G G G G G G G G G G G G G G G G G G G G G G G G G G G G G . G G G G G G G G G G G G G G G G    
1271	. . . . . . . . . . . . . . . . . . . . . . . . . . . . . . . . . . . . . . . . . . . A A A A A A A A A A A A A A A A A A A A A A A A A A A A A A A A A A A A A A A A A A A A A A A A A A A A A A A A A A A A A A A A A A A A A A A A A A A A A A A . A A A A A A A A A A A A A A A A    
1272	. . . . . . . . . . . . . . . . . . . . . . . . . . . . . . . . . . . . . . . . . . . A A . A A A A A A A A A A A A A A A A A A A A A A A A A A A A A A A A A A A A A A A A A A A A A A A A A A A A A A A A A A A A A A A A A A A A A A A A A A A A . A A A A A A A A A A A A A A A A    
1273	. . . . . . . . . . . . . . . . . . . . . . . . . . . . . . . . . . . . . . . . . . . A G . G G G G G G G G G G G G G G G G G G G G G G G G G G G G G G G G G G G G G G G G G G G G G G G G G G G G G G G G G G G G G G G G G G G G G G G G G G G G . G G G G G G G G G G G G G G G G    
1274	. . . . . . . . . . . . . . . . . . . . . . . . . . . . . . . . . . . . . . . . . . . . T . T T T T T T T T T T T T T T T T T T T T T T T T T T T T T T T T T T T T T T T T T T T T T T T T T T T T T T T T T T T T T T T T T T T T T T T T T T T T . T T T T T T T T T T T T T T T T    
1275	. . . . . . . . . . . . . . . . . . . . . . . . . . . . . . . . . . . . . . . . . . . . A . A A A A A A A A A A A A A A A A A A A A A A A A A A A A A A A A A A A A A A A A A A A A A A A A A A A A A A A A A A A A A A A A A A A A A A A A A A A A . A A A A A A A A A A A A A A A A    
1276	. . . . . . . . . . . . . . . . . . . . . . . . . . . . . . . . . . . . . . . . . . . . A . A A A A C A A A A A A A A A A A A A A A A A A A A A A A A A A A A A A A A A A A A A A A A A A A A A A A A A A A A A A A A A A A A A A A A A A A A A A A . A A A A A A A A A A A A A A A A    
1277	. . . . . . . . . . . . . . . . . . . . . . . . . . . . . . . . . . . . . . . . . . . . C . C C C C . C C C C C C C C C C C C C C C C C C C C C C C C C C C C C C C C C C C C C C C C C C C C C C C C C C C C C C C C C C C C C C C C C C C C C C C . C C C C C C C C C C C C C C C C    
1278	. . . . . . . . . . . . . . . . . . . . . . . . . . . . . . . . . . . . . . . . . . . . C . C C C C . C C C C C C C C C C C C C C C C C C C C C C C C C C C C C C C C C C C C C C C C C C C C C C C C C C C C C C C C C C C C C C C C C C C C C C C . C C C C C C C C C C C C C C C C    
1279	. . . . . . . . . . . . . . . . . . . . . . . . . . . . . . . . . . . . . . . . . . . . T . T T T T . T T T T T T T T T T T T T T T T T T T T T T T T T T T T T T T T T T T T T T T T T T T T T T T T T T T T T T T T T T T T T T T T T T T T T T T . T T T T T T T T T T T T T T T T    
1280	. . . . . . . . . . . . . . . . . . . . . . . . . . . . . . . . . . . . . . . . . . . . C . C C C C . C C C C C C C C C C C C C C C C C C C C C C C C C C C C C C C C C C C C C C C C C C C C C C C C C C C C C C C C C C C C C C C C C C C C C C C . C C C C C C C C C C C C C C C C    
1281	. . . . . . . . . . . . . . . . . . . . . . . . . . . . . . . . . . . . . . . . . . . . C . C C C C . C C C C C C C C C C C C C C C C C C C C C C C C C C C C C C C C C C C C C C C C C C C C C C C C C C C C C C C C C C C C C C C C C C C C C C C . C C C C C C C C C C C C C C C C    
1282	. . . . . . . . . . . . . . . . . . . . . . . . . . . . . . . . . . . . . . . . . . . . T . T T T T . T T T T T T T T T T T T T T T T T T T T T T T T T T T T T T T T T T T T T T T T T T T T T T T T T T T T T T T T T T T T T T T T T T T T T T T . T T T T T T T T T T T T T T T T    
1283	. . . . . . . . . . . . . . . . . . . . . . . . . . . . . . . . . . . . . . . . . . . . T . T T T T . T T T T T T T T T T T T T T T T T T T T T T T T T T T T T T T T T T T T T T T T T T T T T T T T T T T T T T T T T T T T T T T T T T T T T T T . T T T T T T T T T T T T T T T T    
1284	. . . . . . . . . . . . . . . . . . . . . . . . . . . . . . . . . . . . . . . . . . . . C . C C C C . C C C C C C C C C C C C C C C C C C C C C C C C C C C C C C C C C C C C C C C C C C C C C C C C C C C C C C C C C C C C C C C C C C C C C C C . C C C C C C C C C C C C C C C C    
1285	. . . . . . . . . . . . . . . . . . . . . . . . . . . . . . . . . . . . . . . . . . . . A . A A A A . A A A A A A A A A A A A A A A A A A A A A A A A A A A A A A A A A A A A A A A A A A A A A A A A A A A A A A A A A A A A A A A A A A A A A A A . A A A A A A A A A A A A A A A A    
1286	. . . . . . . . . . . . . . . . . . . . . . . . . . . . . . . . . . . . . . . . . . . . T . T T T T . T T T T T T T T T T T T T T T T T T T T T T T T T T T T T T T T T T T T T T T T T T T T T T T T T T T T T T T T T T T T T T T T T T T T T T T . T T T T T T T T T T T T T T T T    
1287	. . . . . . . . . . . . . . . . . . . . . . . . . . . . . . . . . . . . . . . . . . . . G . G G G G . G G G G G G G G G G G G G G G G G G G G G G G G G G G G G G G G G G G G G G G G G G G G G G G G G G G G G G G G G G G G G G G G G G G G G G G . G G G G G G G G G G G G G G G G    
1288	. . . . . . . . . . . . . . . . . . . . . . . . . . . . . . . . . . . . . . . . . . . . T . T T T T . T T T T T T T T T T T T T T T T T T T T T T T T T T T T T T T T T T T T T T T T T T T T T T T T T T T T T T T T T T T T T T T T T T T T T T T . T T T T T T T T T T T T T T T T    
1289	. . . . . . . . . . . . . . . . . . . . . . . . . . . . . . . . . . . . . . . . . . . . C . C C C T . C C C C C C C C C C C C C C C C C C C C C C C C C C C C C C C C C C C C C C C C C C C C C C C C C C C C C C C C C C C C C C C C C C C C C C C . C C C C C C C C C C C C C C C C    
1290	. . . . . . . . . . . . . . . . . . . . . . . . . . . . . . . . . . . . . . . . . . . . T . T T T . . T T T T T T T T T T T T T T T T T T T T T T T T T T T T T T T T T T T T T T T T T T T T T T T T T T T T T T T T T T T T T T T T T T T T T T T . T T T T T T T T T T T T T T T T    
1291	. . . . . . . . . . . . . . . . . . . . . . . . . . . . . . . . . . . . . . . . . . . . C . C C C . . C C C C C C C C C C C C C C C C C C C C C C C C C C C C C C C C C C C C C C C C C C C C C C C C C C C C C C C C C C C C C C C C C C C C C C C . C C C C C C C C C C C C C C C C    
1292	. . . . . . . . . . . . . . . . . . . . . . . . . . . . . . . . . . . . . . . . . . . . A . A A A . . A A A A A A A A A A A A A A A A A A A A A A A A A A A A A A A A A A A A A A A A A A A A A A A A A A A A A A A A A A A A A A A A A A A A A A A . A A A A A A A A A A A A A A A A    
1293	. . . . . . . . . . . . . . . . . . . . . . . . . . . . . . . . . . . . . . . . . . . . T . T T T . . T T T T T T T T T T T T T T T T T T T T T T T T T T T T T T T T T T T T T T T T T T T T T T T T T T T T T T T T T T T T T T T T T T T T T T T . T T T T T T T T T T T T T T T T    
1294	. . . . . . . . . . . . . . . . . . . . . . . . . . . . . . . . . . . . . . . . . . . . T . T T T . . T T T T T T T T T T T T T T T T T T T T T T T T T T T T T T T T T T T T T T T T T T T T T T T T T T T T T T T T T T T T T T T T T T T T T T T . T T T T T T T T T T T T T T T T    
1295	. . . . . . . . . . . . . . . . . . . . . . . . . . . . . . . . . . . . . . . . . . . . C . C T C . . C C C C C C C C C C C C C C C C C C C C C C C C C C C C C C C C C C C C C C C C C C C C C C C C C C C C C C C C C C C C C C C C C C C C C C C . C C C C C C C C C C C C C C C C    
1296	. . . . . . . . . . . . . . . . . . . . . . . . . . . . . . . . . . . . . . . . . . . . A . A . A . . A A A A A A A A A A A A A A A A A A A A A A A A A A A A A A A A A A A A A A A A A A A A A A A A A A A A A A A A A A A A A A A A A A A A A A A . A A A A A A A A A A A A A A A A    
1297	. . . . . . . . . . . . . . . . . . . . . . . . . . . . . . . . . . . . . . . . . . . . T . T . T . . T T T T T T T T T T T T T T T T T T T T T T T T T T T T T T T T T T T T T T T T T T T T T T T T T T T T T T T T T T T T T T T T T T T T T T T . T T T T T T T T T T T T T T T T    
1298	. . . . . . . . . . . . . . . . . . . . . . . . . . . . . . . . . . . . . . . . . . . . A . T . A . . A A A A A A A A A A A A A A A A A A A A A A A A A A A A A A A A A A A A A A A A A A A A A A A A A A A A A A A A A A A A A A A A A A A A A A A . A A A A A A A A A A A A A A A A    
1299	. . . . . . . . . . . . . . . . . . . . . . . . . . . . . . . . . . . . . . . . . . . . . . . . A . . A A A A A A A A A A A A A A A A A A A A A A A A A A A A A A A A A A A A A A A A A A A A A A A A A A A A A A A A A A A A A A A A A A A A A A A . A A A A A A A A A A A A A A A A    
1300	. . . . . . . . . . . . . . . . . . . . . . . . . . . . . . . . . . . . . . . . . . . . . . . . T . . T T T T T T T T T T T T T T T T T T T T T T T T T T T T T T T T T T T T T T T T T T T T T T T T T T T T T T T T T T T T T T T T T T T T T T T . T T T T T T T T T T T T T T T T    
1301	. . . . . . . . . . . . . . . . . . . . . . . . . . . . . . . . . . . . . . . . . . . . . . . . G . . G G G G G G G G G G G G G G G G G G G G G G G G G G G G G G G G G G G G G G G G G G G G G G G G G G G G G G G G G G G G G G G G G G G G G G G . G G G G G G G G G G G G G G G G    
1302	. . . . . . . . . . . . . . . . . . . . . . . . . . . . . . . . . . . . . . . . . . . . . . . . T . . T T T T T T T T T T T T T T T T T T T T T T T T T T A T T T T T T T T T T T T T T T T T T T T T T T T T T T T T T T T T T T T T T T T T T T T . T T T T T T T T T T T T T T T T    
1303	. . . . . . . . . . . . . . . . . . . . . . . . . . . . . . . . . . . . . . . . . . . . . . . . C . . C C C C C C C C C C C C C C C C C C C C C C C C C C C C C C C C C C C C C C C C C C C C C C C C C C C C C C C C C C C C C C C C C C C C C C C . C C C C C C C C C C C C C C C C    
1304	. . . . . . . . . . . . . . . . . . . . . . . . . . . . . . . . . . . . . . . . . . . . . . . . T . . T T T T T T T T T T T T T T T T T T T T T T T T T T T T T T T T T T T T T T T T T T T T T T T T T T T T T T T T T T T T T T T T T T T T T T T . T T T T T T T T T T T T T T T T    
1305	. . . . . . . . . . . . . . . . . . . . . . . . . . . . . . . . . . . . . . . . . . . . . . . . A . . A A A A A A A A A A A A A A A A A A A A A A A A A A A A A A A A A A A A A A A A A A A A A A A A A A A A A A A A A A A A A A A A A A A A A A A . A A A A A A A A A A A A A A A A    
1306	. . . . . . . . . . . . . . . . . . . . . . . . . . . . . . . . . . . . . . . . . . . . . . . . T . . T T T T T T T T T T T T T T T T T T T T T T T T T T T T T T T T T T T T T T T T T T T T T T T T T T T T T T T T T T T T T T T T T T T T T T T . T T T T T T T T T T T T T T T T    
1307	. . . . . . . . . . . . . . . . . . . . . . . . . . . . . . . . . . . . . . . . . . . . . . . . A . . A A A A A A A A A A A A A A A A A A A A A A A A A A A A A A A A A A A A A A A A A A A A A A A A A A A A A A A A A A A A A A A A A A A A A A A . A A A A A A A A A A A A A A A A    
1308	. . . . . . . . . . . . . . . . . . . . . . . . . . . . . . . . . . . . . . . . . . . . . . . . T . . T T T T T T T T T T T T T T T T T T T T T T T T T T T T T T T T T T T T T T T T T T T T T T T T T T T T T T T T T T T T T T T T T T T T T T T . T T T T T T T T T T T T T T T T    
1309	. . . . . . . . . . . . . . . . . . . . . . . . . . . . . . . . . . . . . . . . . . . . . . . . G . . G G G G G G G G G G G G G G G G G G G G G G G G G G G G G G G G G G G G G G G G G G G G G G G G G G G G G G G G G G G G G G G G G G G G G G G . G G G G G G G G G G G G G G G G    
1310	. . . . . . . . . . . . . . . . . . . . . . . . . . . . . . . . . . . . . . . . . . . . . . . . G . . G G G G G G G G G G G G G G G G G G G G G G G G G G G G G G G G G G G G G G G G G G G G G G G G G G G G G G G G G G G G G G G G G G G G G G G . G G G G G G G G G G G G G G G G    
1311	. . . . . . . . . . . . . . . . . . . . . . . . . . . . . . . . . . . . . . . . . . . . . . . . T . . T T T T T T T T T T T T T T T T T T T T T T T T T T T T T T T T T T T T T T T T T T T T T T T T T T T T T T C T T T T T T T T T T T T T T T T . T T T T T T T T T T T T T T T T    
1312	. . . . . . . . . . . . . . . . . . . . . . . . . . . . . . . . . . . . . . . . . . . . . . . . . . . T T T T T T T T T T T T T T T T T T T T T T T T T T T T T T T T T T T T T T T T T T T T T T T T T T T T T T T T T T T T T T T T T T T T T T T . T T T T T T T T T T T T T T T T    
1313	. . . . . . . . . . . . . . . . . . . . . . . . . . . . . . . . . . . . . . . . . . . . . . . . . . . T T T T T T T T T T T T T T T T T T T T T T T T T T T T T T T T T T T T T T T T T T T T T T T T T T T T T T T T T T T T T T T T T T T T T T T . T T T T T T T T T T T T T T T T    
1314	. . . . . . . . . . . . . . . . . . . . . . . . . . . . . . . . . . . . . . . . . . . . . . . . . . . C C C C C C C C C C C C C C C C C C C C C C C C C C C C C C C C C C C C C C C C C C C C C C C C C C C C C C C C C C C C C C C C C C C C C C C . C C C C C C C C C C C C C C C C    
1315	. . . . . . . . . . . . . . . . . . . . . . . . . . . . . . . . . . . . . . . . . . . . . . . . . . . A A A A A A A A A A A A A A A A A A A A A A A A A A A A A A A A A A A A A A A A A A A A A A A A A A A A A A A A A A A A A A A A A A A A A A A . A A A A A A A A A A A A A A A A    
1316	. . . . . . . . . . . . . . . . . . . . . . . . . . . . . . . . . . . . . . . . . . . . . . . . . . . G G G G G G G G G G G G G G G G G G G G G G G G G G G G G G G G G G G G G G G G G G G G G G G G G G G G G G G G G G G G G G G G G G G G G G G . G G G G G G G G G G G G G G G G    
1317	. . . . . . . . . . . . . . . . . . . . . . . . . . . . . . . . . . . . . . . . . . . . . . . . . . . C C C C C C C C C C C C C C C C C C C C C C C C C C C C C C C C C C C C C C C C C C C C C C C C C C C C C C C C C C C C C C C C C C C C C C C . C C C C C C C C C C C C C C C C    
1318	. . . . . . . . . . . . . . . . . . . . . . . . . . . . . . . . . . . . . . . . . . . . . . . . . . . A A G G A A A A A A A A A A A A A A A A A A A A A A A A A A A A A A A A A A A A A A A A A A A A A A A A A A A A A A A A G A A A A A A A A A A . A A A A A A A A A A G G G A A A  6	 transition  
1319	. . . . . . . . . . . . . . . . . . . . . . . . . . . . . . . . . . . . . . . . . . . . . . . . . . . C C C C C C C C C C C C C C C C C C C C C C C C C C C C C C C C C C C C C C C C C C C C C C C C C C C C C C C C C C C C C C C C C C C C C C C . C C C C C C C C C C C C C C C C    
1320	. . . . . . . . . . . . . . . . . . . . . . . . . . . . . . . . . . . . . . . . . . . . . . . . . . . C C C C C C C C C C C C C C C C C C C C C C C C C C C C C C C C C C C C C C C C C C C C C C C C C C C C C C C C C C C C C C C C C C C C C C C . C C C C C C C C C C C C C C C C    
1321	. . . . . . . . . . . . . . . . . . . . . . . . . . . . . . . . . . . . . . . . . . . . . . . . . . . C C C C C C C C C C C C C C C C C C C C C C C C C C C C C C C C C C C C C C C C C C C C C C C C C C C C C C C C C C C C C C C C C C C C C C C . C C C C C C C C C C C C C C C C    
1322	. . . . . . . . . . . . . . . . . . . . . . . . . . . . . . . . . . . . . . . . . . . . . . . . . . . T T T T T T T T T T T T T T T T T T T T T T T T T T T T T T T T T T T T T T T T T T T T T T T T T T T T T T T T T T T T T T T T T T T T T T T T T T T T T T T T T T T T T T T T    
1323	. . . . . . . . . . . . . . . . . . . . . . . . . . . . . . . . . . . . . . . . . . . . . . . . . . . T T T T T T T T T T T T T T T T T T T T T T T T T T T T T T T T T T T T T T T T T T T T T T T T T T T T T T T T T T T T T T T T T T T T T T T T T T T T T T T T T T T T T T T T    
1324	. . . . . . . . . . . . . . . . . . . . . . . . . . . . . . . . . . . . . . . . . . . . . . . . . . . C C C C C C C C C C C C C C C C C C C C C C C C C C C C C C C C C C C C C C C C C C C C C C C C C C C C C C C C C C C C C C C C C C C C C C C C C C C C C C C C C C C C C C C C    
1325	. . . . . . . . . . . . . . . . . . . . . . . . . . . . . . . . . . . . . . . . . . . . . . . . . . . A A A A A A A A A A A A A A A A A A A A A A A A A A A A A A A A A A A A A A A A A A A A A A A A A A A A A A A A A A A A A A A A A A A A A A A A A A A A A A A A A A A A A A A A    
1326	. . . . . . . . . . . . . . . . . . . . . . . . . . . . . . . . . . . . . . . . . . . . . . . . . . . T T T T T T T T T T T T T T T T T T T T T T T T T T T T T T T T T T T T T T T T T T T T T T T T T T T T T T T T T T T T T T T T T T T T T T T T T T T T T T T T T T T T T T T T    
1327	. . . . . . . . . . . . . . . . . . . . . . . . . . . . . . . . . . . . . . . . . . . . . . . . . . . T T T T T T T T T T T T T T T T T T T T T T T T T T T T T T T T T T T T T T T T T T T T T T T T T T T T T T T T T T T T T T T T T T T T T T T T T T T T T T T T T T T T T T T T    
1328	. . . . . . . . . . . . . . . . . . . . . . . . . . . . . . . . . . . . . . . . . . . . . . . . . . . T T T T T T T T T T T T T T T T T T T T T T T T T T T T T T T T T T T T T T T T T T T T T T T T T T T T T T T T T T T T T T T T T C T T T T T T T T T T T T T T T T T T T T T T    
1329	. . . . . . . . . . . . . . . . . . . . . . . . . . . . . . . . . . . . . . . . . . . . . . . . . . . T T T T T T T T T T T T T T T T T T T T T T T T T T T T T T T T T T T T T T T T T T T T T T T T T T T T T T T T T T T T T T T T T T T T T T T T T T T T T T T T T T T T T T T T    
1330	. . . . . . . . . . . . . . . . . . . . . . . . . . . . . . . . . . . . . . . . . . . . . . . . . . . C C C C C C C C C C C C C C C C C C C C C C C C C C C C C C C C C C C C C C C C C C C C C C C C C C C C C C C C C C C C C C C C C C C C C C C C C C C C C C C C C C C C C C C C    
1331	. . . . . . . . . . . . . . . . . . . . . . . . . . . . . . . . . . . . . . . . . . . . . . . . . . . T T T T T T T T T T T T T T T T T T T T T T T T T T T T T T T T T T T T T T T T T T T T T T T T T T T T T T T T T T T T T T T T T T T T T T T T T T T T T T T T T T T T T T T T    
1332	. . . . . . . . . . . . . . . . . . . . . . . . . . . . . . . . . . . . . . . . . . . . . . . . . . . G G G G G G G G G G G G G G G G G G G G G G G G G G G G G G G G G G G G G G G G G G G G G G G G G G G G G G G G G G G G G G G G G G G G G G G G G G G G G G G G G G G G G G G G    
1333	. . . . . . . . . . . . . . . . . . . . . . . . . . . . . . . . . . . . . . . . . . . . . . . . . . . C C C C C C C C C C C C C C C C C C C C C C C C C C C C C C C C C C C C C C C C C C C C C C C C C C C C C C C C C C C C C C C C C C C C C C C C C C C C C C C C C C C C C C C C    
1334	. . . . . . . . . . . . . . . . . . . . . . . . . . . . . . . . . . . . . . . . . . . . . . . . . . . T T T T T T T T T T T T T T T T T T T T T T T T T T T T T T T T T T T T T T T T T T T T T T T T T T T T T T T T T T T T T T T T T T T T T T T T T T T T T T T T T T T T T T T T    
1335	. . . . . . . . . . . . . . . . . . . . . . . . . . . . . . . . . . . . . . . . . . . . . . . . . . . C C C C C C C C C C C C C C C C C C C C C C C C C C C C C C C C C C C C C C C C C C C C C C C C C C C C C C C C C C C C C C C C C C C C C C C C C C C C C C C C C C C C C C C C    
1336	. . . . . . . . . . . . . . . . . . . . . . . . . . . . . . . . . . . . . . . . . . . . . . . . . . . . T T T T T T T T T T T T T T T T T T T T T T T T T T T T T T T T T T T T T T T T T T T T T T T T T T T T T T T T T T T T T T T T T T T T T T T T T T T T T T T T T T T T T T T    
1337	. . . . . . . . . . . . . . . . . . . . . . . . . . . . . . . . . . . . . . . . . . . . . . . . . . . . G G G G G G G G G G G G G G G G G G G G G G G G G G G G G G G G G G G G G G G G G G G G G G G G G G G G G G G G G G G G G G G G G G G G G G G G G G G G G G G G G G G G G G G    
1338	. . . . . . . . . . . . . . . . . . . . . . . . . . . . . . . . . . . . . . . . . . . . . . . . . . . . T T T T T T T T T T T T T T T T T T T T T T T T T T T T T T T T T T T T T T T T T T T T T T T T T T T T T T T T T T T T T T T T T T T T T T T T T T T T T T T T T T T T T T T    
1339	. . . . . . . . . . . . . . . . . . . . . . . . . . . . . . . . . . . . . . . . . . . . . . . . . . . . A A A A A A A A A A A A A A A A A A A A A A A A A A A A A A A A A A A A A A A A A A A A A A A A A A A A A A A A A A A A A A A A A A A A A A A A A A A A A A A A A A A A A A A    
1340	. . . . . . . . . . . . . . . . . . . . . . . . . . . . . . . . . . . . . . . . . . . . . . . . . . . . A A A A A A A A A A A A A A A A A A A A A A A A A A A A A A A A A A A A A A A A A A A A A A A A A A A A A A A A A A A A A A A A A A A A A A A A A A A A A A A A A A A A A A A    
1341	. . . . . . . . . . . . . . . . . . . . . . . . . . . . . . . . . . . . . . . . . . . . . . . . . . . . T T T T T T T T T T T T T T T T T T T T T T T T T T T T T T T T T T T T T T T T T T T T T T T T T T T T T T T T T T T T T T T T T T T T T T T T T T T T T T T T T T T T T T T    
1342	. . . . . . . . . . . . . . . . . . . . . . . . . . . . . . . . . . . . . . . . . . . . . . . . . . . . G G G G G G G G G G G G G G G G G G G G G G G G G G G G G G G G G G G G G G G G G G G G G G G G G G G G G G G G G G G G G G G G G G G G G G G G G G G G G G G G G G G G G G G    
1343	. . . . . . . . . . . . . . . . . . . . . . . . . . . . . . . . . . . . . . . . . . . . . . . . . . . . G G G G G G G G G G G G G G G G G G G G G G G G G G G G G G G G G G G G G G G G G G G G G G G G G G G G G G G G G G G G G G G G G G G G G G G G G G G G G G G G G G G G G G G    
1344	. . . . . . . . . . . . . . . . . . . . . . . . . . . . . . . . . . . . . . . . . . . . . . . . . . . . T T T T T T T T T T T T T T T T T T T T T T T T T T T T T T T T T T T T T T T T T T T T T T T T T T T T T T T T T T T T T T T T T T T T T T T T T T T T T T T T T T T T T T T    
1345	. . . . . . . . . . . . . . . . . . . . . . . . . . . . . . . . . . . . . . . . . . . . . . . . . . . . T T T T T T T T T T T T T T T T T T T T T T T T T T T T T T T T T T T T T T T T T T T T T T T T T T T T T T T T T T T T T T T T T T T T T T T T T T T T T T T T T T T T T T T    
1346	. . . . . . . . . . . . . . . . . . . . . . . . . . . . . . . . . . . . . . . . . . . . . . . . . . . . G G G G G G G G G G G G G G G G G G G G G G G G G G G G G G G G G G G G G G G G G G G G G G G G G G G G G G G G G G G G G G G G G G G G G G G G G G G G G G G G G G G G G G G    
1347	. . . . . . . . . . . . . . . . . . . . . . . . . . . . . . . . . . . . . . . . . . . . . . . . . . . . T T T T T T T T T T T T T T T T T T T T T T T T T T T T T T T T T T T T T T T T T T T T T T T T T T T T T T T T T T T T T T T T T T T T T T T T T T T T T T T T T T T T T T T    
1348	. . . . . . . . . . . . . . . . . . . . . . . . . . . . . . . . . . . . . . . . . . . . . . . . . . . . C C C C C C C C C C C C C C C C C C C C C C C C C C C C C C C C C C C C C C C C C C C C C C C C C C C C C C C C C C C C C C C C C C C C C C C C C C C C C C C C C C C C C C C    
1349	. . . . . . . . . . . . . . . . . . . . . . . . . . . . . . . . . . . . . . . . . . . . . . . . . . . . T T T T T T T T T T T T T T T T T T T T T T T T T T T T T T T T T T T T T T T T T T T T T T T T T T T T T T T T T T T T T T T T T T T T T T T T T T T T T T T T T T T T T T T    
1350	. . . . . . . . . . . . . . . . . . . . . . . . . . . . . . . . . . . . . . . . . . . . . . . . . . . . T T T T T T T T T T T T T T T T T T T T T T T T T T T T T T T T T T T T T T T T T T T T T T T T T T T T T T T T T T T T T T T T T T T T T T T T T T T T T T T T T T T T T T T    
1351	. . . . . . . . . . . . . . . . . . . . . . . . . . . . . . . . . . . . . . . . . . . . . . . . . . . . G G G G G G G G G G G G G G G G G G G G G G G G G G G G G G G G G G G G G G G G G G G G G G G G G G G G G G G G G G G G G G G G G G G G G G G G G G G G G G G G G G G G G G G    
1352	. . . . . . . . . . . . . . . . . . . . . . . . . . . . . . . . . . . . . . . . . . . . . . . . . . . . T T T T T T T T T T T T T T T T T T T T T T T T T T T T T T T T T T T T T T T T T T T T T T T T T T T T T T T T T T T T T T T T T T T T T T T T T T T T T T T T T T T T T T T    
1353	. . . . . . . . . . . . . . . . . . . . . . . . . . . . . . . . . . . . . . . . . . . . . . . . . . . . C C C C C C C C C C C C C C C C C C C C C C T T C C C C C C C C C C C C C C C C C C C C C C C C C C C C C C C C C C C C C C C C C C C C C C C C C C C C C C C C C C C C C C C    
1354	. . . . . . . . . . . . . . . . . . . . . . . . . . . . . . . . . . . . . . . . . . . . . . . . . . . . T T T T T T T T T T T T T T T T T T T T T T T T T T T T T T T T T T T T T T T T T T T T T T T T T T T T T T T T T T T T T T T T T T T T T T T T T T T T T T T T T T T T T T T    
1355	. . . . . . . . . . . . . . . . . . . . . . . . . . . . . . . . . . . . . . . . . . . . . . . . . . . . T T T T T T T T T T T T T T T T T T T T T T T T T T T T T T T T T T T T T T T T T T T T T T T T T T T T T T T T T T T T T T T T T T T T T T T T T T T T T T T T T T T T T T T    
1356	. . . . . . . . . . . . . . . . . . . . . . . . . . . . . . . . . . . . . . . . . . . . . . . . . . . . C C C C C C C C C C C C C C C C C C C C C C C C C C C C C C C C C C C C C C C C C C C C C C C C C C C C C C C C C C C C C C C C C C C C C C C C C C C C C T C C C C C C C C C    
1357	. . . . . . . . . . . . . . . . . . . . . . . . . . . . . . . . . . . . . . . . . . . . . . . . . . . . T T T T T T T T T T T T T T T T T T T T T T T T T T T T T T T T T T T T T T T T T T T T T T T T T T T T T T T T T T T T T T T T T T T T T T T T T T T T T T T T T T T T T T T    
1358	. . . . . . . . . . . . . . . . . . . . . . . . . . . . . . . . . . . . . . . . . . . . . . . . . . . . A A A A A A A A A A A A A A A A A A A A A A A A A A A A A A A A A A A A A A A A A A A A A A A A A A A A A A A A A A A A A A A A A A A A A A A A A A A A A A A A A A A A A A A    
1359	. . . . . . . . . . . . . . . . . . . . . . . . . . . . . . . . . . . . . . . . . . . . . . . . . . . . T T T T T T T T T T T T T T T T T T T T T T T T T T T T T T T T T T T T T T T T T T T T T T T T T T T T T T T T T T T T T T T T T T T T T T T T T T T T T T T T T T T T T T T    
1360	. . . . . . . . . . . . . . . . . . . . . . . . . . . . . . . . . . . . . . . . . . . . . . . . . . . . A A A A A A A A A A A A A A A A A A A A A A A A A A A A A A A A A A A A A A A A A A A A A A A A A A A A A A A A A A A A A A A A A A A A A A A A A A A A A A A A A A A A A A A    
1361	. . . . . . . . . . . . . . . . . . . . . . . . . . . . . . . . . . . . . . . . . . . . . . . . . . . . T T T T T T T T T T T T T T T T T T T T T T T T T T T T T T T T T T T T T T T T T T T T T T T T T T T T T T T T T T T T T T T T T T T T T T T T T T T T T T T T T T T T T T T    
1362	. . . . . . . . . . . . . . . . . . . . . . . . . . . . . . . . . . . . . . . . . . . . . . . . . . . . A A A A A A A A A A A A A A A A A A A A A A A A A A A A A A A A A A A A A A A A A A A A A A A A A A A A A A A A A A A A A A A A A A A A A A A A A A A A A A A A A A A A A A A    
1363	. . . . . . . . . . . . . . . . . . . . . . . . . . . . . . . . . . . . . . . . . . . . . . . . . . . . A A A A A A A A A A A A A A A A A A A A A A A A A A A A A A A A A A A A A A A A A A A A A A A A A A A A A A A A A A A A A A A A A A A A A A A A A A A A A A A A A A A A A A A    
1364	. . . . . . . . . . . . . . . . . . . . . . . . . . . . . . . . . . . . . . . . . . . . . . . . . . . . C C C C C C C C C C C C C C C C C C C C C C C C C C C C C C C C C C C C C C C C C C C C C C C C C C C C C C C C C C C C C C C C C C C C C C C C C C C C C C C C C C C C C C C    
1365	. . . . . . . . . . . . . . . . . . . . . . . . . . . . . . . . . . . . . . . . . . . . . . . . . . . . T T T T T T T T T T T T T T T T T T T T T T T T T T T T T T T T T T T T T T T T T T T T T T T T T T T T T T T T T T T T T T T T T T T T T T T T T T T T T T T T T T T T T T T    
1366	. . . . . . . . . . . . . . . . . . . . . . . . . . . . . . . . . . . . . . . . . . . . . . . . . . . . G G G G G G G G G G G G G G G G G G G G G G G G G G G G G G G G G G G G G G G G G G G G G G G G G G G G G G G G G G G G G G G G G G G G G G G G G G G G G G G G G G G G G G G    
1367	. . . . . . . . . . . . . . . . . . . . . . . . . . . . . . . . . . . . . . . . . . . . . . . . . . . . G G G G G G G G G G G G G G G G G G G G G G G G G G G G G G G G G G G G G G G G G G G G G G G G G G G G G G G G G G G G G G G G G G G G G G G G G G G G G G G G G G G G G G G    
1368	. . . . . . . . . . . . . . . . . . . . . . . . . . . . . . . . . . . . . . . . . . . . . . . . . . . . G G G G G G G G G G G G G G G G G G G G G G G G G G G G G G G G G G G G G G G G G G G G G G G G G G G G G G G G G G G G G G G G G G G G G G G G G G G G G G G G G G G G G G G    
1369	. . . . . . . . . . . . . . . . . . . . . . . . . . . . . . . . . . . . . . . . . . . . . . . . . . . . A A A A A A A A A A A A A A A A A A A A A A A A A A A A A A A A A A A A A A A A A A A A A A A A A A A A A A A A A A A A A A A A A A A A A A A A A A A A A A A A A A A A A A A    
1370	. . . . . . . . . . . . . . . . . . . . . . . . . . . . . . . . . . . . . . . . . . . . . . . . . . . . C C C C C C C C C C C C C C C C C C C C C C C C C C C C C C C C C C C C C C C C C C C C C C C C C C C C C C C C C C C C C C C C C C C C C C C C C C C C C C C C C C C C C C C    
1371	. . . . . . . . . . . . . . . . . . . . . . . . . . . . . . . . . . . . . . . . . . . . . . . . . . . . . A A A A A A A A A A A A A A A A A A A A A A A A A A A A A A A A A A A A A A A A A A A A A A A A A A A A A A A A A A A A A A A A A A A A A A A A A A A A A A A A A A A A A A    
1372	. . . . . . . . . . . . . . . . . . . . . . . . . . . . . . . . . . . . . . . . . . . . . . . . . . . . . T T T T T T T T T T T T T T T T T T T T T T T T T T T T T T T T T T T T T T T T T T T T T T T T T T T T T T T T T T T T T T T T T T T T T T T T T T T T T T T T T T T T T T    
1373	. . . . . . . . . . . . . . . . . . . . . . . . . . . . . . . . . . . . . . . . . . . . . . . . . . . . . A A A A A A A A A A A A A A A A A A A A A A A A A A A A A A A A A A A A A A A A A A A A A A A A A A A A A A A A A A A A A A A A A A A A A A A A A A A A A A A A A A A A A A    
1374	. . . . . . . . . . . . . . . . . . . . . . . . . . . . . . . . . . . . . . . . . . . . . . . . . . . . . T T T T T T T T T T T T T T T T T T T T T T T T T T T T T T T T T T T T T T T T T T T T T T T T T T T T T T T T T T T T T T T T T T T T T T T T T T T T T T T T T T T T T T    
1375	. . . . . . . . . . . . . . . . . . . . . . . . . . . . . . . . . . . . . . . . . . . . . . . . . . . . . T T T T T T T T T T T T T T T T T T T T T T T T T T T T T T T T T T T T T T T T T T T T T T T T T T T T T T T T T T T T T T T T T T T T T T T T T T T T T T T T T T T T T T    
1376	. . . . . . . . . . . . . . . . . . . . . . . . . . . . . . . . . . . . . . . . . . . . . . . . . . . . . T T T T T T T T T T T T T T T T T T T T T T T T T T T T T T T T T T T T T T T T T T T T T T T T T T T T T T T T T T T T T T T T T T T T T T T T T T T T T T T T T T T T T T    
1377	. . . . . . . . . . . . . . . . . . . . . . . . . . . . . . . . . . . . . . . . . . . . . . . . . . . . . T T T T T T T T T T T T T T T T T T T T T T T T T T T T T T T T T T T T T T T T T T T T T T T T T T T T T T T T T T T T T T T T T T T T T T T T T T T T T T T T T T T T T T    
1378	. . . . . . . . . . . . . . . . . . . . . . . . . . . . . . . . . . . . . . . . . . . . . . . . . . . . . A A A A A A A A A A A A A A A A A A A A A A A A A A A A A A A A A A A A A A A A A A A A A A A A A A A A A A A A A A A A A A A A A A A A A A A A A A A A A A A A A A A A A A    
1379	. . . . . . . . . . . . . . . . . . . . . . . . . . . . . . . . . . . . . . . . . . . . . . . . . . . . . G G G G G G G G G G G G G G G G G G G G G G G G G G G G G G G G G G G G G G G G G G G G G G G G G G G G G G G G G G G G G G G G G G G G G G G G G G G G G G G G G G G G G G    
1380	. . . . . . . . . . . . . . . . . . . . . . . . . . . . . . . . . . . . . . . . . . . . . . . . . . . . . A A A A A A A A A A A A A A A A A A A A A A A A A A A A A A A A A A A A A A A A A A A A A A A A A A A A A A A A A A A A A A A A A A A A A A A A A A A A A A A A A A A A A A    
1381	. . . . . . . . . . . . . . . . . . . . . . . . . . . . . . . . . . . . . . . . . . . . . . . . . . . . . C C C C C C C C C C C C C C C C C C C C C C C C C C C C C C C C C C C C C C C C C C C C C C C C C C C C C C C C C C C C C C C C C C C C C C C C C C C C C C C C C C C C C C    
1382	. . . . . . . . . . . . . . . . . . . . . . . . . . . . . . . . . . . . . . . . . . . . . . . . . . . . . G G G G G G G G G G G G G G G G G G G G G G G G G G G G G G G G G G G G G G G G G G G G G G G G G G G G G G G G G G G G G G G G G G G G G G G G G G G G G G G G G G G G G G    
1383	. . . . . . . . . . . . . . . . . . . . . . . . . . . . . . . . . . . . . . . . . . . . . . . . . . . . . T T T T T T T T T T T T T T T T T T T T T T T T T T T T T T T T T T T T T T T T T T T T T T T T T T T T T T T T T T T T T T T T T T T T T T T T T T T T T T T T T T T T T T    
1384	. . . . . . . . . . . . . . . . . . . . . . . . . . . . . . . . . . . . . . . . . . . . . . . . . . . . . T T T T T T T T T T T T T T T T T T T T T T T T T T T T T T T T T T T T T T T T T T T T T T T T T T T T T T T T T T T T T T T T T C T T T T T T T T T T T T T T T T T T T T    
1385	. . . . . . . . . . . . . . . . . . . . . . . . . . . . . . . . . . . . . . . . . . . . . . . . . . . . . C C C C C C C C C C C C C C C C C C C C C C C C C C C C C C C C C C C C C C C C C C C C C C C C C C C C C C C C C C C C C C C C C C C C C C C C C C C C C C C C C C C C C C    
1386	. . . . . . . . . . . . . . . . . . . . . . . . . . . . . . . . . . . . . . . . . . . . . . . . . . . . . C C C C C C C C C C C C C C C C C C C C C C C C C C C C C C C C C C C C C C C C C C C C C C C C C C C C C C C C C C C C C C C C C C C C C C C C . C C C C C C C C C C C C C    
1387	. . . . . . . . . . . . . . . . . . . . . . . . . . . . . . . . . . . . . . . . . . . . . . . . . . . . . T T T T T T T T T T T T T T T T T T T T T T T T T T T T T T T T T T T T T T T T T T T T T T T T T T T T T T C C T T T T T T T T T T T T T T T T . T T T T T T T T T T T T T    
1388	. . . . . . . . . . . . . . . . . . . . . . . . . . . . . . . . . . . . . . . . . . . . . . . . . . . . . C C C C C C C C C C C C C C C C C C C C C C C C C C C C C C C C C C C C C C C C C C C C C C C C C C C C C C . . C C C C C C C C C C C C C C C C . C C C C C . C C C C C C C    
1389	. . . . . . . . . . . . . . . . . . . . . . . . . . . . . . . . . . . . . . . . . . . . . . . . . . . . . T T T T T T T T T T T T T T T T T T T T T T T T T T T T T T T T T T T T T T T T T T T T T T T T T T T T T T . . T T T T T T T T T T T T T T T T . T T T T T . T T T T T T T    
1390	. . . . . . . . . . . . . . . . . . . . . . . . . . . . . . . . . . . . . . . . . . . . . . . . . . . . . G G G G G G G G G G G G G G G G G G G G G G G G G G G G G G G G G G G G G G G G G G G G G G G G G G G G G G . . G G G G G G G G G G G G G G G G . G G G G G . G G G G G G G    
1391	. . . . . . . . . . . . . . . . . . . . . . . . . . . . . . . . . . . . . . . . . . . . . . . . . . . . . A A A A A A A A A A A A A A A A A A A A A A A A A A A A A A A A A A A A A A A A A A A A A A A A A A A A A A . . A A A A A A A A A A A A A A A A . A A A A A . A A A A A A A    
1392	. . . . . . . . . . . . . . . . . . . . . . . . . . . . . . . . . . . . . . . . . . . . . . . . . . . . . T T T T T T T T T T T T T T T T T T T T T T T T T T T T T T T T T T T T T T T T T T T T T T T T T T T T T T . . T T T T T T T T T T T T T T T T . T T T T T . T T T T T T T    
1393	. . . . . . . . . . . . . . . . . . . . . . . . . . . . . . . . . . . . . . . . . . . . . . . . . . . . . . T T T T T T T T T T T T T T T T T T . T T T T T T T T T T T T T T T T T T T T T T T T T T T T T . T T T T . . T T T T T T T T T T T T T T T T . T T T T T . T T T T T T T    
1394	. . . . . . . . . . . . . . . . . . . . . . . . . . . . . . . . . . . . . . . . . . . . . . . . . . . . . . T T T T T T T T T T T T T T T T T T . T T T T T T T T T T T T T T T T T T T T T T T T T T T T T . T T T T . . T T T T T T T T T T T T T T T T . T T T T T . T T T T T T T    
1395	. . . . . . . . . . . . . . . . . . . . . . . . . . . . . . . . . . . . . . . . . . . . . . . . . . . . . . G G G G G G G G G G G G G G G G G G . G G G G G G G G G G G G G G G G G G G G G G G G G G G G G . G G G G . . G G G G G G G G G G G G G G G G . G G G G G . G G G G G G G    
1396	. . . . . . . . . . . . . . . . . . . . . . . . . . . . . . . . . . . . . . . . . . . . . . . . . . . . . . G G G G G G G G G G G G G G G G G G . G G G G G G G G G G G G G G G G G G G G G G G G G G G G G . G G G G . . G G G G G G G G G G G G G G G G . G G G G G . G G G G G G G    
1397	. . . . . . . . . . . . . . . . . . . . . . . . . . . . . . . . . . . . . . . . . . . . . . . . . . . . . . T T T T T T T T T T T T T T T T T T . T T T T T T T T T T T T T T T T T T T T T T T T T T T T T . T T T T . . T T T T T T T T T T T T T T . T . T T T T T . T T T T T T T    
1398	. . . . . . . . . . . . . . . . . . . . . . . . . . . . . . . . . . . . . . . . . . . . . . . . . . . . . . A A A A A A A A A A A A A A A A A A . A A A A A A A A A A A A A A A A A A A A A A A A A A A A A . A A A A . . A A A A A A A A A A A A A A . . . A A A A A . A A A A A A A    
1399	. . . . . . . . . . . . . . . . . . . . . . . . . . . . . . . . . . . . . . . . . . . . . . . . . . . . . . C C C C C C C C C C C C C C C C C C . C C C C C C C C C C C C C C C C C C C C C C C C C C C C C . C C C C . . C C C C C C C C C C C C C C . . . C C C C C . C C C C C C C    
1400	. . . . . . . . . . . . . . . . . . . . . . . . . . . . . . . . . . . . . . . . . . . . . . . . . . . . . . A A A A A A A A A A A A A A A A A A . A A A A A A A A A A A A A A A A A A A A A A A A A A A A A . A A A A . . A A A A A A A A A A A A A A . . . A A A A A . A A A A A A A    
1401	. . . . . . . . . . . . . . . . . . . . . . . . . . . . . . . . . . . . . . . . . . . . . . . . . . . . . . A A A A A A A A A A A A A A A A A A . A A A A A A A A A A A A A A A A A A A A A A A A A A A A A . A A A A . . A A A A A A A A A A A A A A . . . A A A A A . A A A A A A A    
1402	. . . . . . . . . . . . . . . . . . . . . . . . . . . . . . . . . . . . . . . . . . . . . . . . . . . . . . G G G G G G G G G G G G G G G G G G . G G G G G G G G G G G G G G G G G G G G G G G G G G G G G . G G G G . . G G G G G G G G G G G G G G . . . G G G G G . G G G G G G G    
1403	. . . . . . . . . . . . . . . . . . . . . . . . . . . . . . . . . . . . . . . . . . . . . . . . . . . . . . C C C C C C . C C C C C C C C C C C . G C C C C C . C C C C C C C C C C C C C C C C C C C C C C . C C C C . . C C C C C C C C C C C C C C . . . C C C C C . C C C C C C C    
1404	. . . . . . . . . . . . . . . . . . . . . . . . . . . . . . . . . . . . . . . . . . . . . . . . . . . . . . T T T T T T . T T T T T T T T T T T . . T T T T . . T T T T T T T T T T T T T T T T T T T T T T . T T T T . . T T T T T T T T T T T T T T . . . T T T T T . T T T T T T T    
1405	. . . . . . . . . . . . . . . . . . . . . . . . . . . . . . . . . . . . . . . . . . . . . . . . . . . . . . T T T T T T . T T T T T T T T T T T . . T T T T . . T T T T T T T T T T T T T T T T T T T T T T . T T T T . . T T T T T T T T T T T T T T . . . T T T T T . T T T T T T T    
1406	. . . . . . . . . . . . . . . . . . . . . . . . . . . . . . . . . . . . . . . . . . . . . . . . . . . . . . T T T T T T . T T T T T T T T T T T . . T T T T . . T T T T T T T T T T T T T T T T T T T T T T . T T T T . . T T T T T T T T T T T T T T . . . T T T T T . T T T T T T T    
1407	. . . . . . . . . . . . . . . . . . . . . . . . . . . . . . . . . . . . . . . . . . . . . . . . . . . . . . A A A A A A . A A A A A A A A A A A . . A A A A . . A A A A A A A A A A A A A A A A A A A A T T . A A A A . . A A A A A A A A A A A A A A . . . A A A A A . A A A A A A A    
1408	. . . . . . . . . . . . . . . . . . . . . . . . . . . . . . . . . . . . . . . . . . . . . . . . . . . . . . C C C C C C . C C C C C C C C C C C . . C C C C . . C C C C C C C C C C C C C C C C C C C C . . . C C C C . . C C C C C C C C C C C C C C . . . C C C C C . C C C C C C C    
1409	. . . . . . . . . . . . . . . . . . . . . . . . . . . . . . . . . . . . . . . . . . . . . . . . . . . . . . A A A A A A . A A A A A A A A A A A . . A A A A . . A A A A A A A A A A A A A A A A A A A A . . . A A A A . . A A A A A A A A A A A A A A . . . A A A A A . A A A A A A A    
1410	. . . . . . . . . . . . . . . . . . . . . . . . . . . . . . . . . . . . . . . . . . . . . . . . . . . . . . A A A A A A . A A A A A A A A A A A . . A A A A . . A A A A A A A A A A A A A A A A A A A A . . . A A A A . . A A A A A A A A A A A A A A . . . A A A A A . A A A A A A A    
1411	. . . . . . . . . . . . . . . . . . . . . . . . . . . . . . . . . . . . . . . . . . . . . . . . . . . . . . T T T T T T . T T T T T T T T T T T . . T T T T . . T T T T T T T T T T T T T T T T T T T T . . . T T T T . . T T T T T T T T T T T T T T . . . T T T T T . T T T T T T T    
1412	. . . . . . . . . . . . . . . . . . . . . . . . . . . . . . . . . . . . . . . . . . . . . . . . . . . . . . A A A A A A . A A A A A A A A A A A . . A A A A . . A A A A A A A A A A A A A A A A A A A A . . . A A A A . . A A A A A A A A A A A A A A . . . A A A A A . A A A A A A A    
1413	. . . . . . . . . . . . . . . . . . . . . . . . . . . . . . . . . . . . . . . . . . . . . . . . . . . . . . A A A A A A . A A A A A A A A A A A . . A A A A . . A A A A A A A A A A A A A A A A A A A A . . . A A A A . . A A A A A A A A A A A A A A . . . A A A A A . A A A A A A A    
1414	. . . . . . . . . . . . . . . . . . . . . . . . . . . . . . . . . . . . . . . . . . . . . . . . . . . . . . A A A A A A . A A A A A A A A A A A . . A A A A . . A A A A A A A A A A A A A A A A A A A A . . . A A A A . . A A A A A A A A A A A A A A . . . A A A A A . A A A A A A A    
1415	. . . . . . . . . . . . . . . . . . . . . . . . . . . . . . . . . . . . . . . . . . . . . . . . . . . . . . C C C C C C . C C C C C C C C C C C . . C C C C . . C C C C C C C C C C C C C C C C C C C C . . . C C C C . . C C C C C C C C C C C C C C . . . C C C C C . C C C C C C C    
1416	. . . . . . . . . . . . . . . . . . . . . . . . . . . . . . . . . . . . . . . . . . . . . . . . . . . . . . C C C C C C . C A C C C C C C C C C . . C C C . . . C C C C C C C C C C C C C C C C C C C C . . . C C C C . . C C C C C C C C C C C C C C . . . C C C C C . C C C C C C C    
1417	. . . . . . . . . . . . . . . . . . . . . . . . . . . . . . . . . . . . . . . . . . . . . . . . . . . . . . A A A A A A . A . A A A A A A A A A . . A A A . . . A A A A A A A A A A A A A A A A A A A A . . . A A A A . . A A . A A A A A A A A A A A . . . A A A A A . A A A A A A A    
1418	. . . . . . . . . . . . . . . . . . . . . . . . . . . . . . . . . . . . . . . . . . . . . . . . . . . . . . T T T T T T . T . T T T T T T T T T . . T T T . . . T T T T T T T T T T T T T T T T T T T T . . . T T T T . . T T . T T T T T T T T T T T . . . T T T T T . T T T T T T T    
1419	. . . . . . . . . . . . . . . . . . . . . . . . . . . . . . . . . . . . . . . . . . . . . . . . . . . . . . T T T T T T . T . T T T T T T T T T . . T T T . . . T T T T T T T T T T T T A T T T T T T T . . . T T T T . . T T . T T T T T T T T T T T . . . T T T T T . T T T T T T T    
1420	. . . . . . . . . . . . . . . . . . . . . . . . . . . . . . . . . . . . . . . . . . . . . . . . . . . . . . A A A A A A . A . A A A A A A A A A . . A A A . . . A A A A A A A A A A A A . A A A A A A A . . . A A A A . . A A . A A A A A A A A A A A . . . A A A A A . A A A A A . A    
1421	. . . . . . . . . . . . . . . . . . . . . . . . . . . . . . . . . . . . . . . . . . . . . . . . . . . . . . A A A A A A . A . A A A A A A A A A . . A A A . . . A A A A A A A A A A A A . A A A A A A A . . . A A A A . . A A . A A A A A A A A A A A . . . A A A A A . A A A A A . A    
1422	. . . . . . . . . . . . . . . . . . . . . . . . . . . . . . . . . . . . . . . . . . . . . . . . . . . . . . A A A A A A . A . A A A A A A A A A . . A A A . . . A A A A A A A A A A A A . A A A A A A A . . . A A A A . . A A . A A A A A A A A A A A . . . A A A A A . A A A C A . A    
1423	. . . . . . . . . . . . . . . . . . . . . . . . . . . . . . . . . . . . . . . . . . . . . . . . . . . . . . C C C C C C . C . C C C C C C C C C . . C C C . . . C C C C C C C C C C C C . C C C C C C C . . . C C C C . . C C . C C C C C C C C C C C . . . C C C C C . C C C . C . C    
1424	. . . . . . . . . . . . . . . . . . . . . . . . . . . . . . . . . . . . . . . . . . . . . . . . . . . . . . A A A A A A . A . A A A A A A A A A . . A A A . . . A A A A A A A A A A A A . A A C A A A A . . . A A A A . . A A . A A A A A A A A A A A . . . A A A A A . A A A . A . A    
1425	. . . . . . . . . . . . . . . . . . . . . . . . . . . . . . . . . . . . . . . . . . . . . . . . . . . . . . T T T T T T . T . T T T T T T T T T . . T T T . . . T T T T T T T T T T T T . T T . T T T T . . . T T T T . . T T . T T T T T T T T T T T . . . T T T T T . T T T . T . T    
1426	. . . . . . . . . . . . . . . . . . . . . . . . . . . . . . . . . . . . . . . . . . . . . . . . . . . . . . G G G G G G . G . G T G G G G G G G . . G G G . . . G G G G G G G G G G G G . G A . G G G G . . . G G G G . . G G . G G G G G G G G G G G . . . G G G G G . G G G . G . G    
1427	. . . . . . . . . . . . . . . . . . . . . . . . . . . . . . . . . . . . . . . . . . . . . . . . . . . . . . T T T T T T . T . T . T T T T T T T . . C C T . . . T . T T T T T T T T T T . T . . T T T T . . . . G G T . . T T . T T T T T T T T T T T . . . T T T T T . T T T . T . T    
1428	. . . . . . . . . . . . . . . . . . . . . . . . . . . . . . . . . . . . . . . . . . . . . . . . . . . . . . . . . . . . . . . . . . . . . . . . . . . . . . . . . . . . . . . . . . . . . . . . . . . . . . . . . . C . . . C . C C C C A C C G C C . . . . . . . . . . . C . . . . C    
1429	. . . . . . . . . . . . . . . . . . . . . . . . . . . . . . . . . . . . . . . . . . . . . . . . . . . . . . . . . . . . . . . . . . . . . . . . . . . . . . . . . . . . . . . . . . . . . . . . . . . . . . . . . . G . . . G . G G G G . G G . G G . . . . . . . . . . . A . . . . G    
1430	. . . . . . . . . . . . . . . . . . . . . . . . . . . . . . . . . . . . . . . . . . . . . . . . . . . . . . . . . . . . . . . . . . . . . . . . . . . . . . . . . . . . . . . . . . . . . . . . . . . . . . . . . . T . . . T . T T T T . T T . T T . . . . . . . . . . . . . . . . T    
1431	. . . . . . . . . . . . . . . . . . . . . . . . . . . . . . . . . . . . . . . . . . . . . . . . . . . . . . . . . . . . . . . . . . . . . . . . . . . . . . . . . . . . . . . . . . . . . . . . . . . . . . . . . . G . . . G . G G G G . . G . G G . . . . . . . . . . . . . . . . G    
1432	. . . . . . . . . . . . . . . . . . . . . . . . . . . . . . . . . . . . . . . . . . . . . . . . . . . . . . . . . . . . . . . . . . . . . . . . . . . . . . . . . . . . . . . . . . . . . . . . . . . . . . . . . . A . . . A . A A A A . . A . A A . . . . . . . . . . . . . . . . A    
1433	. . . . . . . . . . . . . . . . . . . . . . . . . . . . . . . . . . . . . . . . . . . . . . . . . . . . . . . . . . . . . . . . . . . . . . . . . . . . . . . . . . . . . . . . . . . . . . . . . . . . . . . . . . T . . . T . T T T T . . T . T T . . . . . . . . . . . . . . . . T    
1434	. . . . . . . . . . . . . . . . . . . . . . . . . . . . . . . . . . . . . . . . . . . . . . . . . . . . . . . . . . . . . . . . . . . . . . . . . . . . . . . . . . . . . . . . . . . . . . . . . . . . . . . . . . G . . . G . G G G G . . G . G G . . . . . . . . . . . . . . . . G    
1435	. . . . . . . . . . . . . . . . . . . . . . . . . . . . . . . . . . . . . . . . . . . . . . . . . . . . . . . . . . . . . . . . . . . . . . . . . . . . . . . . . . . . . . . . . . . . . . . . . . . . . . . . . . C . . . C . C C C C . . C . C C . . . . . . . . . . . . . . . . C    
1436	. . . . . . . . . . . . . . . . . . . . . . . . . . . . . . . . . . . . . . . . . . . . . . . . . . . . . . . . . . . . . . . . . . . . . . . . . . . . . . . . . . . . . . . . . . . . . . . . . . . . . . . . . . . . . . . . . . . . . . . . . . . . . . . . . . . . . . . . . . A    
1437	. . . . . . . . . . . . . . . . . . . . . . . . . . . . . . . . . . . . . . . . . . . . . . . . . . . . . . . . . . . . . . . . . . . . . . . . . . . . . . . . . . . . . . . . . . . . . . . . . . . . . . . . . . . . . . . . . . . . . . . . . . . . . . . . . . . . . . . . . . A    
1438	. . . . . . . . . . . . . . . . . . . . . . . . . . . . . . . . . . . . . . . . . . . . . . . . . . . . . . . . . . . . . . . . . . . . . . . . . . . . . . . . . . . . . . . . . . . . . . . . . . . . . . . . . . . . . . . . . . . . . . . . . . . . . . . . . . . . . . . . . . A    
1439	. . . . . . . . . . . . . . . . . . . . . . . . . . . . . . . . . . . . . . . . . . . . . . . . . . . . . . . . . . . . . . . . . . . . . . . . . . . . . . . . . . . . . . . . . . . . . . . . . . . . . . . . . . . . . . . . . . . . . . . . . . . . . . . . . . . . . . . . . . A    
1440	. . . . . . . . . . . . . . . . . . . . . . . . . . . . . . . . . . . . . . . . . . . . . . . . . . . . . . . . . . . . . . . . . . . . . . . . . . . . . . . . . . . . . . . . . . . . . . . . . . . . . . . . . . . . . . . . . . . . . . . . . . . . . . . . . . . . . . . . . . A    
1441	. . . . . . . . . . . . . . . . . . . . . . . . . . . . . . . . . . . . . . . . . . . . . . . . . . . . . . . . . . . . . . . . . . . . . . . . . . . . . . . . . . . . . . . . . . . . . . . . . . . . . . . . . . . . . . . . . . . . . . . . . . . . . . . . . . . . . . . . . . A    
1442	. . . . . . . . . . . . . . . . . . . . . . . . . . . . . . . . . . . . . . . . . . . . . . . . . . . . . . . . . . . . . . . . . . . . . . . . . . . . . . . . . . . . . . . . . . . . . . . . . . . . . . . . . . . . . . . . . . . . . . . . . . . . . . . . . . . . . . . . . . A    
1443	. . . . . . . . . . . . . . . . . . . . . . . . . . . . . . . . . . . . . . . . . . . . . . . . . . . . . . . . . . . . . . . . . . . . . . . . . . . . . . . . . . . . . . . . . . . . . . . . . . . . . . . . . . . . . . . . . . . . . . . . . . . . . . . . . . . . . . . . . . A    
1444	. . . . . . . . . . . . . . . . . . . . . . . . . . . . . . . . . . . . . . . . . . . . . . . . . . . . . . . . . . . . . . . . . . . . . . . . . . . . . . . . . . . . . . . . . . . . . . . . . . . . . . . . . . . . . . . . . . . . . . . . . . . . . . . . . . . . . . . . . . A    
1445	. . . . . . . . . . . . . . . . . . . . . . . . . . . . . . . . . . . . . . . . . . . . . . . . . . . . . . . . . . . . . . . . . . . . . . . . . . . . . . . . . . . . . . . . . . . . . . . . . . . . . . . . . . . . . . . . . . . . . . . . . . . . . . . . . . . . . . . . . . G    
1446	. . . . . . . . . . . . . . . . . . . . . . . . . . . . . . . . . . . . . . . . . . . . . . . . . . . . . . . . . . . . . . . . . . . . . . . . . . . . . . . . . . . . . . . . . . . . . . . . . . . . . . . . . . . . . . . . . . . . . . . . . . . . . . . . . . . . . . . . . . G    
1447	. . . . . . . . . . . . . . . . . . . . . . . . . . . . . . . . . . . . . . . . . . . . . . . . . . . . . . . . . . . . . . . . . . . . . . . . . . . . . . . . . . . . . . . . . . . . . . . . . . . . . . . . . . . . . . . . . . . . . . . . . . . . . . . . . . . . . . . . . . G    
1448	. . . . . . . . . . . . . . . . . . . . . . . . . . . . . . . . . . . . . . . . . . . . . . . . . . . . . . . . . . . . . . . . . . . . . . . . . . . . . . . . . . . . . . . . . . . . . . . . . . . . . . . . . . . . . . . . . . . . . . . . . . . . . . . . . . . . . . . . . . G    
1449	. . . . . . . . . . . . . . . . . . . . . . . . . . . . . . . . . . . . . . . . . . . . . . . . . . . . . . . . . . . . . . . . . . . . . . . . . . . . . . . . . . . . . . . . . . . . . . . . . . . . . . . . . . . . . . . . . . . . . . . . . . . . . . . . . . . . . . . . . . G    
1450	. . . . . . . . . . . . . . . . . . . . . . . . . . . . . . . . . . . . . . . . . . . . . . . . . . . . . . . . . . . . . . . . . . . . . . . . . . . . . . . . . . . . . . . . . . . . . . . . . . . . . . . . . . . . . . . . . . . . . . . . . . . . . . . . . . . . . . . . . . G    
1451	. . . . . . . . . . . . . . . . . . . . . . . . . . . . . . . . . . . . . . . . . . . . . . . . . . . . . . . . . . . . . . . . . . . . . . . . . . . . . . . . . . . . . . . . . . . . . . . . . . . . . . . . . . . . . . . . . . . . . . . . . . . . . . . . . . . . . . . . . . G    
1452	. . . . . . . . . . . . . . . . . . . . . . . . . . . . . . . . . . . . . . . . . . . . . . . . . . . . . . . . . . . . . . . . . . . . . . . . . . . . . . . . . . . . . . . . . . . . . . . . . . . . . . . . . . . . . . . . . . . . . . . . . . . . . . . . . . . . . . . . . . C    
1453	. . . . . . . . . . . . . . . . . . . . . . . . . . . . . . . . . . . . . . . . . . . . . . . . . . . . . . . . . . . . . . . . . . . . . . . . . . . . . . . . . . . . . . . . . . . . . . . . . . . . . . . . . . . . . . . . . . . . . . . . . . . . . . . . . . . . . . . . . . C    
1454	. . . . . . . . . . . . . . . . . . . . . . . . . . . . . . . . . . . . . . . . . . . . . . . . . . . . . . . . . . . . . . . . . . . . . . . . . . . . . . . . . . . . . . . . . . . . . . . . . . . . . . . . . . . . . . . . . . . . . . . . . . . . . . . . . . . . . . . . . . C
```
